# Combination of Isothermal Recombinase-Aided Amplification and CRISPR-Cas12a-Mediated Assay for Rapid Detection of Major Severe Acute Respiratory Syndrome Coronavirus 2 Variants of Concern

**DOI:** 10.3389/fmicb.2022.945133

**Published:** 2022-06-28

**Authors:** Hongqing Lin, Yuanhao Liang, Lirong Zou, Baisheng Li, Jianhui Zhao, Haiying Wang, Jiufeng Sun, Xiaoling Deng, Shixing Tang

**Affiliations:** ^1^Department of Epidemiology, School of Public Health, Southern Medical University, Guangzhou, China; ^2^Institute of Pathogenic Microbiology, Guangdong Provincial Center for Disease Control and Prevention, Guangdong Workstation for Emerging Infectious Disease Control and Prevention, Chinese Academy of Medical Sciences, Guangzhou, China; ^3^Wenzhou Institute, University of Chinese Academy of Sciences, Wenzhou, China; ^4^Department of Infectious Diseases, Nanfang Hospital, Southern Medical University, Guangzhou, China

**Keywords:** SARS-CoV-2, isothermal amplification, CRISPR-Cas12a, variants of concern, variant genotyping

## Abstract

Coronavirus disease 2019 (COVID-19) pandemic caused by SARS-CoV-2 variants is a new and unsolved threat; therefore, it is an urgent and unmet need to develop a simple and rapid method for detecting and tracking SARS-CoV-2 variants. The spike gene of SARS-CoV-2 was amplified by isothermal recombinase-aided amplification (RAA) followed by the cleavage of CRISPR-Cas12a in which five allele-specific crRNAs and two Omicron-specific crRNAs were designed to detect and distinguish major SARS-CoV-2 variants of concerns (VOCs), including alpha, beta, delta variants, and Omicron sublineages BA.1 and BA.2. The whole reaction can be carried out in one tube at 39°C within 1.5–2 h, and the results can be read out by a fluorescence meter or naked eyes. Our results show that the RAA/CRISPR-Cas12a-based assay could readily distinguish the signature mutations, i.e., K417N, T478K, E484K, N501Y, and D614G, with a sensitivity of 100.0% and a specificity of 94.9–100.0%, respectively. The assay had a low limit of detection (LOD) of 10^4^ copies/reaction and a concordance of 92.59% with Sanger sequencing results when detecting 54 SARS-CoV-2 positive clinical samples. The two Omicron-specific crRNAs can readily and correctly distinguish Omicron BA.1 and BA.2 sublineages with a LOD of as low as 20 copies/reaction. Furthermore, no cross-reaction was observed for all crRNAs analyzed when detecting clinical samples infected with 11 common respiratory pathogens. The combination of isothermal amplification and CRISPR-Cas12a-mediated assay is suitable for rapid detection of major SARS-CoV-2 variants in point-of-care testing and in resource-limiting settings. This simple assay could be quickly updated for emerging variants and implemented to routinely monitor and track the spread of SARS-CoV-2 variants.

## Introduction

The ongoing pandemic of coronavirus disease 2019 (COVID-19) caused by the emerging variants of severe acute respiratory syndrome coronavirus 2 (SARS-CoV-2) is a great challenge for the prevention and control of the COVID-19 epidemic (Tao et al., 2021; Dong et al., 2022). According to the relevant biological properties and public health concerns, these emerged variants have been classified by the World Health Organization (WHO) into variants of concern (VOCs, including alpha, beta, gamma, delta, and recently identified Omicron), variants of interest (VOIs, including lambda and mu), or variants under monitoring (VUMs, including kappa, iota, and eta; World Health Organization [WHO], 2022). The SARS-CoV-2 variants are mainly characterized by the signature mutations in the spike protein, which are proved to be associated with higher transmissibility and virulence (Dong et al., 2021; Khandia et al., 2022), and compromise the efficacy of COVID-19 vaccines (Brown et al., 2021; Hacisuleyman et al., 2021; Kroidl et al., 2021; Rovida et al., 2021; Vignier et al., 2021). Therefore, the ability to rapidly screen and monitor the spread of SARS-CoV-2 variants is essential to control the COVID-19 pandemic and to timely adjust vaccination strategy.

There are several methods to identify and detect SARS-CoV-2 mutations and variants, such as viral whole-genome sequencing, although the cost and complexity may limit its accessibility (Chiara et al., 2021), reverse transcription-polymerase chain reactions (RT-PCR)-based nucleic acid tests, which are the gold-standard technology for diagnosis of SARS-CoV-2 infection and have been developed to distinguish SARS-CoV-2 variants (Vega-Magaña et al., 2021; Vogels et al., 2021; Wang et al., 2021; Zelyas et al., 2021). However, the above two methods are time-consuming and labor-intensive and are difficult to be widely implemented. At present, only a few methods for rapidly detecting SARS-CoV-2 variants have been reported (de Puig et al., 2021; Welch et al., 2022).

In the past decades, the CRISPR-Cas-based detection platform has emerged as the next-generation of molecular diagnostics and has become a powerful tool for pathogen detection or genotyping by using specific CRISPR RNAs (crRNAs). The Cas13a-based SHERLOCK (specific high-sensitivity enzymatic reporter unlocking) platform was able to identify subtypes of Zika virus and dengue virus (Gootenberg et al., 2017, 2018), whereas the Cas12a-based DETECTR (DNA endonuclease-targeted CRISPR *trans* reporter) platform could discriminate between genotypes 16 and 18 of HPV (Chen et al., 2018). The detection sensitivity can be further enhanced by combining it with a pre-amplification step such as isothermal enzymatic reaction to fulfill clinical requirements. CRISPR-Cas-based assays have been developed for detecting SARS-CoV-2 (Broughton et al., 2020; Joung et al., 2020).

For the purpose of characterizing and differentiating the major VOCs of SARS-CoV-2 by using CRISPR-based assays, it is required to carefully design and select specific crRNAs that can discriminate single-nucleotide mutations in the target sequences. We have previously reported a system to combine RT-PCR and CRISPR-Cas12a-mediated assay to detect major VOCs of SARS-CoV-2 with high sensitivity and specificity (Liang et al., 2021), because mismatches between the crRNAs and the target sequences would inhibit the cleavage activity of Cas12a proteins and could be adapted to distinguish SARS-CoV-2 variants. Although the aforementioned RT-PCR/CRISPR-Cas12a-based approach is affordable, simple, and rapid, it is still not feasible for point-of-care testing (POCT) because qPCR equipment and facilities are required. Multiple reactions increase the complexity of testing and the risk of contamination by PCR products. Herein, we refined the system by integrating isothermal recombinase-aided amplification (RAA) technology with a CRISPR-Cas12a-mediated assay to develop a one-tube genotyping assay for major SARS-CoV-2 VOCs, including Omicron sub-linages BA.1 and BA.2. In our RAA/CRISPR-Cas12a-mediated assay, nucleic acid amplification and CRISPR-Cas12a-mediated cleavage could be processed in one tube at 39°C within 1.5–2 h without the need of high-end facilities or trained technicians. The results can be read out by fluorescence meter or judged by naked eyes.

## Materials and Methods

### Clinical Samples

A total of 54 SARS-CoV-2 positive samples, including 4 nucleic acid samples and 50 oropharyngeal specimens, were included in this study. A total of 50 oropharyngeal specimens were confirmed by real-time quantitative reverse transcription PCR assay (Easydiagnosis Biomedicine Co., Ltd., Wuhan, China) targeting both ORF1a/b and nucleocapsid (NP) genes of SARS-CoV-2 and by Sanger sequencing in the Guangdong Provincial Center for Diseases Prevention and Control between March 2020 and December 2021. Demographic data including sampling date, age, gender, infection sources, and disease stages, but no patient identification information, were collected (Supplementary Table 1). In addition, a total of 19 SARS-CoV-2 negative clinical samples infected with various respiratory pathogens collected before the COVID-19 pandemic were used as negative controls and for the evaluation of assay specificity. These respiratory pathogens include common human coronavirus (HCoV) 229E, OC43, and HKU1 as well as rhinovirus (HRV), adenovirus (ADV), respiratory syncytial virus (RSV) A and B, human bocavirus (HBoV), human metapneumovirus (HMPV), and human parainfluenza virus one (HPIV-1) and four (HPIV-4). Written informed consent was obtained from all subjects enrolled in this study. Research protocols were in accordance with the Declaration of Helsinki.

### Cas12a Proteins

The LbCas12a gene of the Lachnospiraceae bacterium (Addgene #69988) and AsCas12a of *Acidaminococcus* sp. (Addgene #114073) were cloned into expression vector pET-28a (+) and transformed into DE3 competent cells (TransGen Biotech, Beijing, China), respectively, to express LbCas12a and AsCas12a proteins in our laboratory. Expressed proteins were purified on HisTrap HP columns (Marlborough, MA, United States), and eluted proteins were dialyzed in storage buffer (600 mM NaCl, 5% glycerol, 2 mM DTT, 50 mM Tris–HCl, pH 7.5; Supplementary Figure 1). The concentration of purified proteins was further quantitated using the BCA Protein Assay Kit (Thermo Fisher Scientific, Waltham, MA, United States). Aliquots of purified proteins were stored at −80°C until use. In addition, we also purchased LbCas12a (New England Biolabs, Ipswich, MA, United States) and LbCas12a (Bio-lifesci, Guangzhou, China) to develop a CRISPR-Cas12a assay.

### Construction of the Plasmids of Severe Acute Respiratory Syndrome Coronavirus 2 Spike Gene

The full-length genomic fragment (nt21,563–25,384) of the spike (S) protein of the SARS-CoV-2 wild-type strain (GenBank accession no. MN908947) and the mutant S gene containing mutations of L5F, D80A, D215G, R246I, K417N, L452R, Y453F, T478K, E484Q, N501Y, A570D, D614G, P681H, A701V, T716I, S982A, D1118H, P1263L, and the gene fragment of Omicron sublineages BA.1 and BA.2 spike proteins were synthesized and inserted into the vector pUC57 (Sangon Biotech Co., Ltd., Shanghai, China) to be used as templates for developing the CRISPR-Cas12a assay. The detailed information on the plasmids used in this study is available in Supplementary Table 2.

### Design and Preparation of the Primers and crRNAs

The primers used for isothermal amplification were designed to target the conserved sequences of the SARS-CoV-2 spike gene according to the manufacturer’s instructions of the recombinase-aided amplification nucleic acid amplification kit (Qitian, Jiangsu, China). The length of the forward and reverse primers was 32–37 nucleotides (nt), and the melting temperatures were around 54–67°C. The expected amplicon size was 209–523 bp. Since a T-rich protospacer adjacent motif (PAM, 5′-TTTN-3′, where N refers to A/G/C) sequence at the 5′ terminus of the target sequence is necessary for the activation of Cas12a protein (Zetsche et al., 2015), an artificial PAM sequence was inserted into the primers to produce amplified products with a PAM motif when necessary.

We downloaded from the GenBank database, the sequences of spike protein of wild-type and major SARS-CoV-2 variants were collected from different countries or regions and conducted alignment analysis (Supplementary Figure 2), and 5 signature mutations in the spike protein (K417N, T478K, E484K, N501Y, and D614G) were identified and selected for developing a CRISPR-Cas12a-based assay (Supplementary Table 3). A total of 5 allele-specific crRNAs targeting the aforementioned 5 signature mutations were designed according to the working principle of the CRISPR-Cas12a system (Zetsche et al., 2015). In addition, two Omicron-specific crRNAs were designed, i.e., an Omicron sublineage BA.1-specific crRNA (crRNA-S-49X) covering Q493R, G496S, and Q498R mutations and an Omicron-specific crRNA (crRNA-S-50X) covering Q498R and N501Y mutations. For the preparation of crRNAs, DNA oligonucleotides containing T7 promoter, conserved stem-loop sequences, and guide sequences and the completely complementary single-stranded DNAs were synthesized and denatured at 95°C for 10 min and annealed from 95 to 25°C with a temperature reduction of 2°C every minute. Afterward, 1 μg purified dsDNA was transcribed at 37°C for 4 h using HiScribe T7 High Yield RNA Synthesis Kit (New England Biolabs, Ipswich, MA, United States). The transcription product was treated with 4 units of DNase I (New England Biolabs, Ipswich, MA, United States) at 37°C for 40 min and then purified using the miRNeasy Serum/Plasma Kit (Qiagen, Hilden, Germany). The concentration of crRNAs was quantified using the NanoDrop 2000 spectrophotometer (Thermo Fisher Scientific, Waltham, MA, United States). All the primer and crRNA sequences are listed in Supplementary Table 4, and all the oligonucleotides were synthesized using the Ruiboxingke Biotechnology (Beijing, China).

### Recombinase-Aided Amplification/CRISPR-Cas12a-Mediated Assay

**FIGURE 1:**
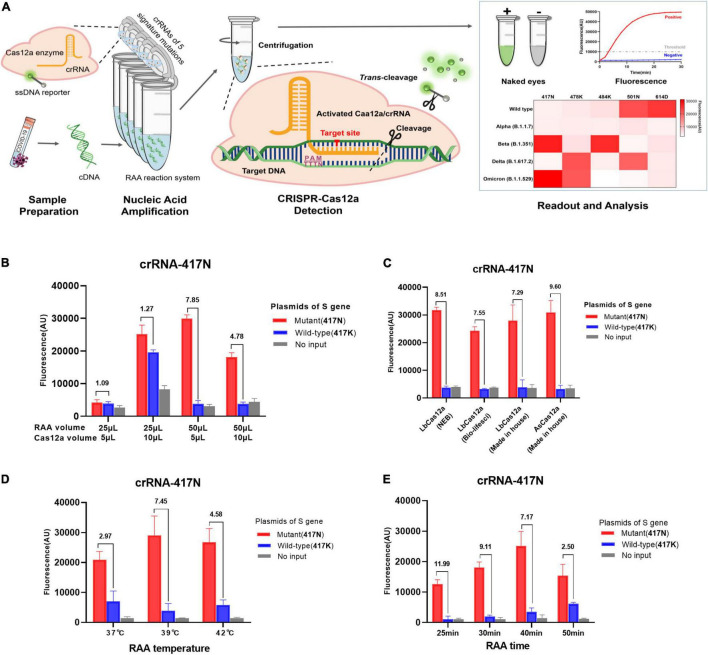
RAA/CRISPR-Cas12a-mediated direct detection of SARS-CoV-2 mutations. **(A)** Workflow of RAA/CRISPR-Cas12a-mediated assay for SARS-CoV-2 variant detection. In brief, viral RNA is extracted, reverse transcripted into cDNA and amplified by recombinase-based isothermal amplification (left panel). Then, the CRISPR-Cas12a reagents are centrifuged and mixed with amplification products to initiate CRISPR-Cas12a-mediated *cis*-cleavage of the amplified products and *trans*-cleavage of reporter DNA (middle panel). Finally, the detection results are measured by a fluorescent detector or read directly by the naked eye under a blue light imager and presented in a heat map (right panel). **(B–E)** Optimization of RAA/CRISPR-Cas12a-mediated assay by using the plasmid DNA of K417N mutant (1 × 10^4^ copies/μl) and wild-type spike gene (1 × 10^6^ copies/μl) as template. The detection efficiency was evaluated according to the volume of RAA and CRISPR-Cas12a reaction mixture **(B)**, different sources and types of Cas12a proteins **(C)**, RAA reaction temperature **(D)**, or reaction time **(E)**. Fluorescence values are represented as mean ± standard deviation (SD) of three replicates. The fluorescence ratio of sample over control is presented at the top of each panel. The amino acid is indicated in the brackets. No input refers to no DNA template.

Viral RNA was extracted from oropharyngeal swab samples of confirmed COVID-19 patients by using the QIAamp Viral RNA Mini Kit (Qiagen, Hilden, Germany) and reverse-transcribed to cDNA using Oligo(dT) or random primer (Roche Diagnostics, Indianapolis, IN, United States). The recombinase-aided amplification reaction (RAA) was performed according to the instructions of the RAA basic kit (Qitian, Jiangsu, China) with slight modifications. Briefly, 25 μl of rehydration buffer, 2 μl of each primer (10 μM), 2 μl of target DNA template, and 16.5 μl of nuclease-free water were added into the tube containing a dried enzyme pellet (including recombinase, single-stranded DNA binding protein, and strand-displacing DNA polymerase) and 2.5 μl of magnesium acetate (280 mM). Subsequently, 5 μl of CRISPR reaction mixture [6 μM of crRNA, 0.8 μM of AsCas12a, 3 μl of NEB buffer 2.1 and 2 μM probe reporter (5′-6-FAM-TTATT-BHQ-1-3′)] were transferred to the lid of the RAA reaction tube and incubated at 39°C for amplification (25 min for crRNA-S-49X and 50X while 40 min for other 5 specific crRNAs). After that, the tube was centrifuged to move the CRISPR-Cas12a reagents to the bottom of tube and incubated at 39°C for 30–40 min for detection (Figure 1A). The fluorescence signal was measured by a fluorescent detector (Qitian, Jiangsu, China) in real-time or judged by the naked eye under a portable blue light imager (Sangon Biotech, Shanghai, China).

### Statistical Analysis

Data analysis was conducted using the IBM SPSS software, version 22 (IBM Corporation, Armonk, NY, United States). The two-tailed Mann–Whitney *U* test and Fisher’s exact test were used to analyze the difference detected by the CRISPR-Cas12a-mediated assay. The receiver-operating characteristic curve (ROC) and the area under the ROC curve (AUC) were calculated to assess the performance of the CRISPR-Cas12a-mediated assay, while the cutoff value was estimated according to the Youden index. Positive predictive value (PPV) and negative predictive value (NPV), as well as 95% binomial confidence intervals, were calculated according to Clopper–Pearson score. The concordance between the CRISPR-Cas12a-based assay and Sanger sequencing was calculated according to the kappa value. *P*-value < 0.05 was considered statistically significant. Data plotting was performed using the GraphPad Prism software (Version 8.0, La Jolla, CA, United States).

## Results

### Optimization of Recombinase-Aided Amplification/CRISPR-Cas12a-Mediated Assay

The purpose of this study was to develop a rapid and simple system by integrating RAA and CRISPR-Cas12a reaction in one tube as shown in Figure 1A. We adapted a strategy of two separate reactions of RAA and CRISPR-Cas12a cleavage in the same tube to avoid invalid amplification caused by the early cleavage of the target template by activated Cas12a when RAA and Cas12a-mediated digestion reacted simultaneously. We optimized the reaction conditions by detecting K417N mutation and found that the volume of RAA and CRISPR-Cas12a reaction as well as the ratio of the two mixtures significantly affected the amplification efficiency of RAA and the *trans*-cleavage efficiency of CRISPR-Cas12a for ssDNA reporter (Figure 1B). As shown in Figure 1B, the combination of 50 μl RAA and 5 μl CRISPR-Cas12a reaction mixture exhibited the greatest fluorescence ratio of 7.85 for the positive (417N) over the negative control (417K), suggesting that the final concentration of the reagents and templates are critical for the efficiency and specificity of both RAA and CRISPR-Cas12a reactions. However, the types and sources of Cas12a proteins did not significantly affect the cleavage activity since the fluorescence ratio for detecting 417N over 417K was quite similar when using the in-house-made AsCas12a and LbCas12a or commercially available LbCas12a (Figure 1C).

Furthermore, the highest efficiency of isothermal amplification was obtained when the RAA reaction was conducted at 39°C, where the signal ratio was 7.45, greater than at 37°C (ratio = 2.97) or 42°C (4.58), respectively (Figure 1D). Interestingly, we found that the extended isothermal amplification could increase the strength of fluorescence signal but decrease the fluorescence ratio since the signal ratio was 11.99, 9.11, 7.17, and 2.50 when the RAA lasted for 25, 30, 40, and 50 min, respectively (Figure 1E). Considering the relatively low fluorescence signal at 25 and 30 min, we decided that the optimized condition for RAA was at 39°C for 40 min.

Next, we tested the efficiency of the CRISPR reaction at 37°C and 39°C using the optimized conditions as above since the Cas detection reaction had only been tested at 37°C so far, although a recent study reported that AsCas12a was robust to temperature (Ooi et al., 2021). We observed that the fluorescence readout even increased slightly at 39°C (Supplementary Figure 3). Therefore, we conclude that our RAA/CRISPR-Cas12a assay could be performed at the same temperature of 39°C.

### Design and Evaluation of Recombinase-Aided Amplification Primers and crRNAs

**FIGURE 2:**
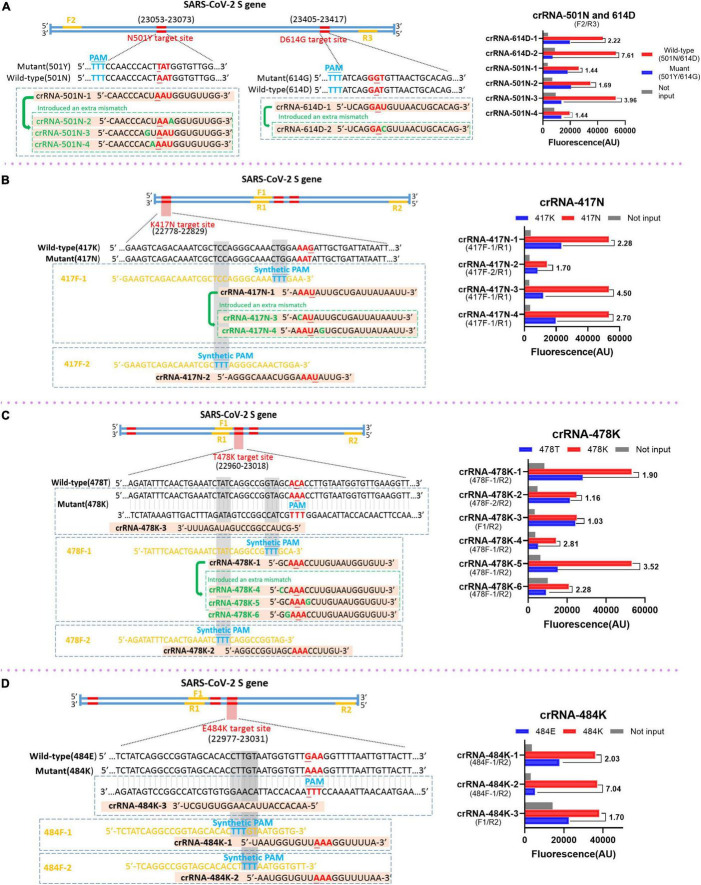
Design and selection of RAA primers and crRNAs. The schematic of the sequences and positions of RAA primers (yellow), the crRNAs with the specific mutations (red), the protospacer adjacent motif PAM (blue) for the mutations of SARS-CoV-2 spike gene (left panel), and the performance of the crRNAs (right panel) for the mutation of N501Y and D614G **(A)**, K417N **(B)**, T478K **(C)**, and E484K **(D)**, respectively. In the right panels, the wild-type and mutant template were labeled in red and blue, respectively. No input means negative control and was labeled in gray. The RAA primer sets for the corresponding crRNAs are presented within the parenthesis.

Different strategies were applied in this study to design and select the RAA primers and crRNAs. For the mutations N501Y and D614G in which the target sequences already have the PAM motif that is required for the recognition and cleavage of Cas12a protein, the principles for designing and selecting RAA primers just follow the criteria of RAA reaction. Our results indicated that the primer sets F2 and R3 could efficiently amplify the templates with N501Y and D614G mutations (data not shown) and were used in the subsequent analysis (Figure 2A). Interestingly, we found that the original crRNAs crRNA-614D-1 and crRNA-501N-1 that are specific for 614D and 501N mutations did not distinguish between D614G and N501Y very well, respectively (Figure 2A). We then designed a crRNA-614D-2 and another three crRNAs, i.e., crRNA-501N-2, -3, and -4 by introducing extra mutations around the 614D or 501N mutation. Our results indicated that the crRNA-614D-2 and crRNA-501N-3 distinguished D614G and N501Y more efficiently and specifically than other crRNAs (Figure 2A). A more complicated strategy was adopted for K417N, T478K, and E484K mutations, which do not contain suitable PAM motifs around these mutations. We first designed the RAA forward primers by inserting an artificial PAM motif (5′-TTTN-3′) into the 3′ end of the forward RAA primers in order to introduce the PAM sequences into the amplified fragments (Figures 2B–D). A series of crRNAs with extra mutations were designed to evaluate their performance in distinguishing K417N, T478K, and E484K mutations, respectively. Our results identified the best combination of the appropriate RAA primers and crRNAs, i.e., the primer set of 417F-1/R1 and crRNA-417N-3 for K417N (Figure 2B); 478F-1/R2 and crRNA-478K-5 for T478K (Figure 2C); and 484F-1/R2 and crRNA-484K-2 for E484K (Figure 2D). Therefore, the above optimized RAA primer sets and crRNAs were included for the following evaluation of our RAA-CRISPR-Cas12a-mediated genotyping assay.

### Detection Limit and Specificity of Recombinase-Aided Amplification/CRISPR-Cas12a Assay

**FIGURE 3:**
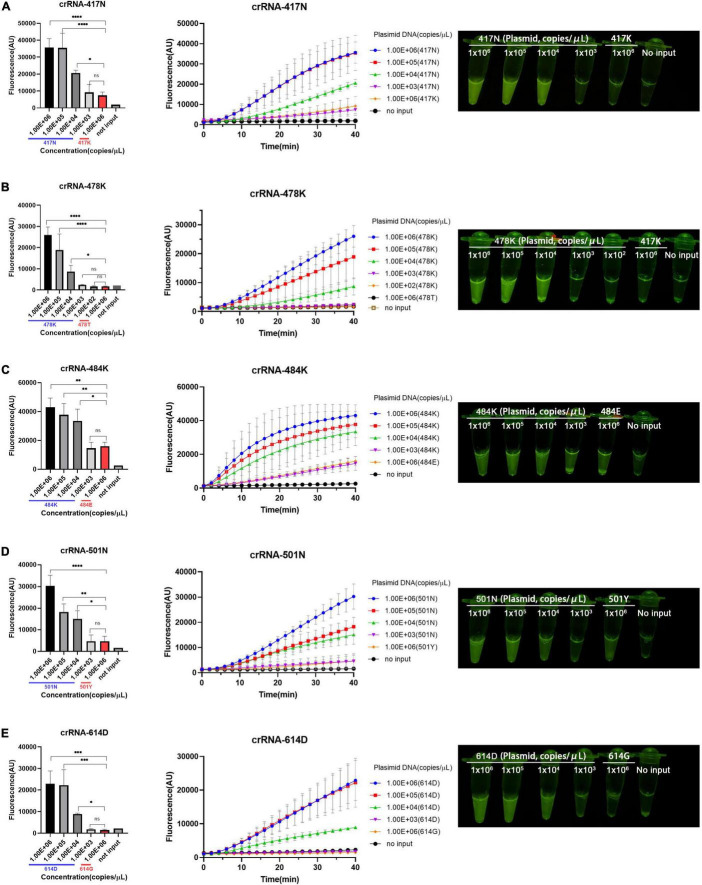
Limit of detection (LOD) of RAA/CRISPR-Cas12a-mediated detection. The LOD was determined and presented for the 5 crRNAs specific for the amino acid 417N **(A)**, 478K **(B)**, 484K **(C)**, 501N **(D)**, and 614D **(E)** of SARS-CoV-2 spike protein, respectively. A series of 10-fold diluted synthetic SARS-CoV-2 plasmid DNAs of wild-type (10^2^–10^6^ copies/μl) and mutant S gene (10^6^ copies/μl) were used as the templates for RAA followed by the detection of CRISPR-Cas12a-mediated assay. Fluorescence intensity are represented as mean ± standard deviation (SD) of three replicates or read out directly by the naked eye under a blue light imager. No input refers to no DNA template. Two-tailed Mann–Whitney *U* test was used to analyze the fluorescence difference between on-target and off-target template detected by the RAA/Cas12a-mediated assay. ns, *P* > 0.05; *P < 0.05; ^**^P < 0.01; ^***^P < 0.001; ^****^P < 0.0001.

According to the aforementioned optimized conditions as well as the RAA primers and crRNAs, we determined the low limit of detection (LOD) of the RAA/CRISPR-Cas12a-mediated assay using 10-fold serial dilutions of the target DNA templates, which ranged from 10^3^ to 10^6^ copies/μl. We found that there was a very good correlation between the reaction time and fluorescence intensity, and a linear relationship was observed in the presence of 10^4^ copies/μl of the target templates (Figure 3). Our results indicated that the 5 signature mutations could be readily detected by fluorescent detector or by naked eyes under blue light by using crRNA-417N-3, crRNA-478K-5, crRNA-484K-3, crRNA-501N-3, and crRNA-614D-2 when the templates were as low as 10^4^ copies/μl (Figures 3A–E), indicating the LOD of 10^4^ copies for our RAA/CRISPR-Cas12a-mediated assay.

**FIGURE 4:**
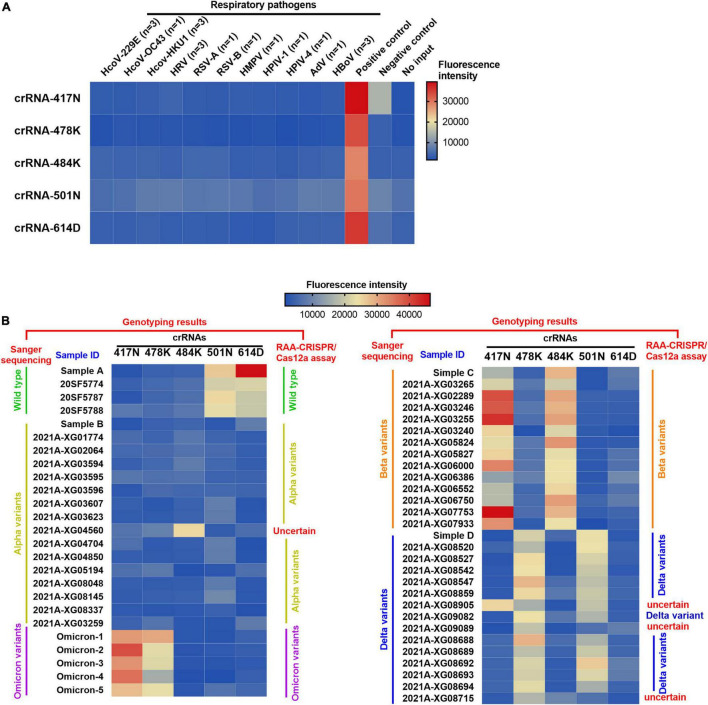
Heat map for the testing results of SARS-CoV-2 positive clinical samples and negative controls detected by the RAA/CRISPR-Cas12a-mediated assay. **(A)** The DNA plasmid of SARS-CoV-2 S gene was used as positive control, while SARS-CoV-2 negative clinical samples infected with common human coronavirus (HKU1, 229E, and OC43), respiratory syncytial virus (RSV) A and B, parainfluenza virus (HPIV) 1 and 4, rhinovirus (HRV), adenovirus (AdV), human bocavirus (HBoV), and human metapneumovirus (HMPV) were used as negative controls to validate the specificity of our assay. No input refers to negative control. **(B)** A total of 54 SARS-CoV-2 positive clinical samples in a panel of 4 wild-type strains, 16 alpha variants, 14 beta variants, 15 delta variants, and 5 Omicron variants were detected by the RAA/CRISPR-Cas12a-mediated assay using a set of crRNAs including crRNA-417N, crRNA-478K, crRNA-484K, crRNA-501N, and crRNA-614D, which are indicated at the top of the panel. The sample ID is presented at the top of the panel. The genotyping results of Sanger sequencing and the RAA/CRISPR-Cas12a-mediated assay are presented at the left and the right of the panel, respectively. Uncertain means that the genotype could not be determined based on our RAA/CRISPR-Cas12a-mediated assay. The corresponding fluorescence values were displayed in colors. The scale bar shows the range of fluorescence values while the color change from blue to red represented the increased strength of signals.

Moreover, the specificity of the RAA/CRISPR-Cas12a assay was validated with clinical samples infected with 11 common respiratory viruses, including common human coronavirus (HKU1, 229E, and OC43), respiratory syncytial virus (RSV) A and B, parainfluenza virus (HPIV) 1 and 4, rhinovirus (HRV), adenovirus (AdV), human bocavirus (HBoV), and human metapneumovirus (HMPV). As shown in Figure 4A, robust fluorescence signal was observed when the plasmid of the SARS-CoV-2 S gene was included as positive control, but no cross-reaction was found when detecting SARS-CoV-2 negative samples.

### Performance of Recombinase-Aided Amplification/CRISPR-Cas12a Assay in Detecting Severe Acute Respiratory Syndrome Coronavirus 2 Variants of Concerns

We examined 54 SARS-CoV-2 positive clinical samples, including 4 samples infected with wild-type strains, 16 with alpha variant, 14 with beta variant, 15 with delta variant, and 5 with Omicron variant, and compared them with Sanger sequencing results (Figure 4B). There was no significant difference as to baseline characteristics between the samples infected with wild-type or different SARS-CoV-2 variants (Supplementary Table 5). In general, all the allele-specific crRNAs could specifically identify the corresponding signature mutations, and the comprehensive results of all the allele-specific crRNAs could accurately distinguish SARS-CoV-2 strains with or without the corresponding mutations (Figure 4B). For example, a strong fluorescence signal was observed in the clinical samples infected with the wild-type strain when using 614D-specific crRNA-614D since only the wild-type strain contains the original 614D amino acid, while a very weak signal was detected in the clinical samples infected with alpha, delta, and Omicron variant since they all carry the D614G substitution (Figure 4B). Similar results were obtained for other specific crRNAs (Figure 4B). According to the cutoff values for each crRNA determined by the ROC curves (Supplementary Figure 4), a sensitivity of 100.0% and a specificity of 94.9–100% were obtained for the crRNAs tested when compared with Sanger sequencing results (Table 1). Furthermore, our RAA/CRISPR-Cas12a-mediated assay showed a concordance of 92.59% (50/54) with Sanger sequencing. The positive and negative predicative values were 100% and 92.9–100.0%, respectively (Table 2). Of note, our RAA/CRISPR-Cas12a-mediated assay characterized the virus in one sample 2021A-XG08905 as delta plus variant (AY.1), which is a delta variant with an extra mutation of K417N (Kannan et al., 2021). The results are consistent with Sanger sequencing data. However, our CRISPR-Cas12a-mediated assay also showed a false positive signal of an extra 484K mutation in one sample (2021A-XG04560) infected with the alpha variant and failed to detect the N501Y mutation in 2 samples (2021A-XG09089 and 2021A-XG08715) that were infected with the delta variant (Figure 4B). All the testing results, as measured by fluorescence meter and judged by naked eyes under blue light, were consistent and presented in Supplementary Figure 5.

**TABLE 1:** Performance of allele-specific crRNAs in RAA/CRISPR-Cas12a-mediated assay compared with Sanger sequencing.

RAA/CRISPR testing results	Sequencing results		ROC curve area	*P* value	cut-off value	Sensitivity (%, 95% CI)	Specificity (%, 95% CI)	Positive predictive value (%, 95% CI)	Negative predictive value (%, 95% CI)	Kappa value
	
	Detected	Not detected								
crRNA-417N			1	<0.0001	>10437	100.0 (78.0–100.0)	100.0 (87.4–100.0)	100.0 (78.0–100.0)	100.0 (87.4–100.0)	1.000
Detected	20	0								
Not detected	0	34								
crRNA-478K			1	<0.0001	>11758	100.0 (78.0–100.0)	100.0 (87.4–100.0)	100.0 (78.0–100.0)	100.0 (87.4–100.0)	1.000
Detected	20	0								
Not detected	0	34								
crRNA-484K			99.33	<0.0001	>14935	100.0 (73.2–100.0)	97.50 (85.3–99.87)	93.33 (66.0–99.7)	100.0 (88.8–100.0)	0.953
Detected	14	1								
Not detected	0	39								
crRNA-501N			97.87	<0.0001	>12792	100.0 (74.7–100.0)	94.87 (81.4–99.11)	88.23 (62.3–97.9)	100.0 (88.3–100.0)	0.911
Detected	15	2								
Not detected	0	37								
crRNA-614D			1	<0.001	>14238	100.0 (39.6–100.0)	100.0 (91.1–100.0)	100.0 (39.6–100.0)	100.0 (91.1–100.0)	1.000
Detected	4	0								
Not detected	0	50								

**TABLE 2:** Concordance between RAA/CRISPR-Cas12a-based assay and Sanger sequencing.

RAA/CRISPR testing results	Sequencing results		Sensitivity (%, 95% CI)	Specificity (%, 95% CI)	Positive predictive value (%, 95% CI)	Negative predictive value (%, 95% CI)	Kappa value
	
	Detected	Not detected					
Wild-type strain			100.0 (39.6–100.0)	100.0 (91.1–100.0)	100.0 (39.6–100.0)	100.0 (91.1–100.0)	1.000
Detected	4	0					
Not detected	0	50					
Alpha variant			93.8 (67.7–99.7)	100.0 (88.6–100.0)	100.0 (74.7–100.0)	97.4 (82.9–99.9)	0.955
Detected	15	0					
Not detected	1	38					
Beta variant			100.0 (73.2–100.0)	100.0 (89.1–100.0)	100.0 (73.2–100.0)	100.0 (89.1–100.0)	1.000
Detected	14	0					
Not detected	0	40					
Delta variant			80.0 (51.4–94.7)	100.0 (88.8–100.0)	100.0 (69.9–100.0)	92.9 (79.4–98.1)	0.852
Detected	12	0					
Not detected	3	39					
Omicron variant			100.0 (46.3–100.0)	100.0 (90.9–100.0)	100.0 (46.3–100.0)	100.0 (90.9–100.0)	1.000
Detected	5	0					
Not detected	0	49					

### Detection of Omicron Sublineages Using Single Omicron-Specific crRNA

**FIGURE 5:**
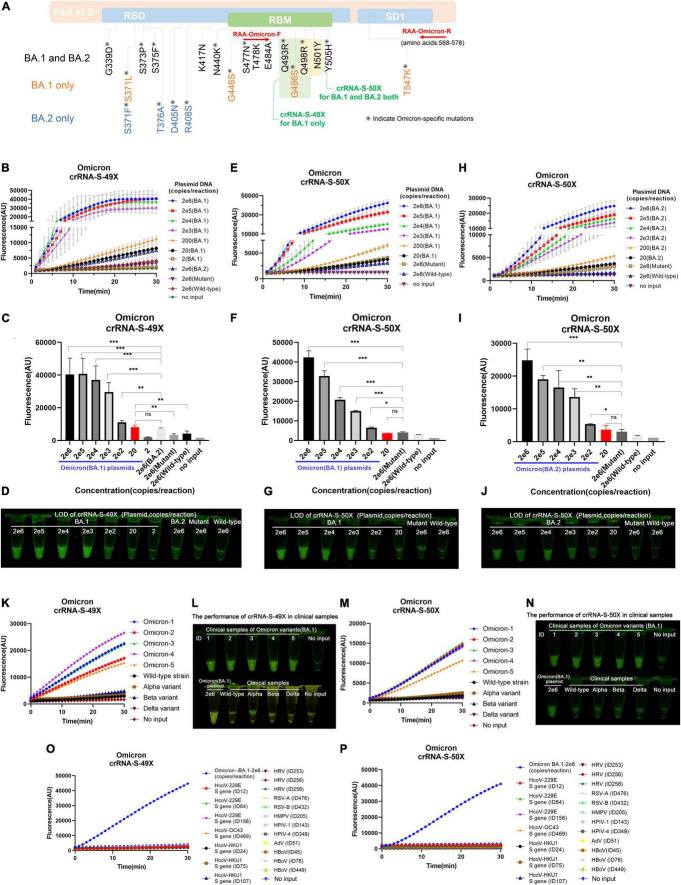
Detection of Omicron BA.1 and BA.2 sublineages *via* optimized RAA/CRISPR-Cas12a-mediated assay by using crRNA-S-49X and crRNA-S-50X. **(A)** The schematic of the specific mutations and the RAA primers. The shared mutations of BA.1 and BA.2 (black) and the mutations specific for BA.1 (yellow) or BA.2 (blue) were presented, while the RAA primers were labeled in red. A series of 10-fold diluted synthetic SARS-CoV-2 plasmid DNAs of wild-type, SARS-CoV-2 mutant, and Omicron BA.1 and BA.2 sublineages were used as the templates for RAA/CRISPR-Cas12a-mediated assay. The low limit of detection was determined and quantitatively analyzed for BA.1 template by using crRNA-S-49X **(B,C)** and crRNA-S-50X **(E,F)**, whereas the LOD of crRNA-S-50X was analyzed using BA.2 template **(H,I)**. Testing results were visualized by the naked eyes under blue light at 30 min post-reaction **(D,G,J)**. Five clinical samples infected with BA.1 sublineage could be specifically distinguish from other SARS-CoV-2 VOCs-infected samples by using crRNA-S-49X **(K,L)** and crRNA-S-50X **(M,N)**. Both crRNA-S-49X **(K)** and crRNA-S-50X **(M)** could specifically detect Omicron variant, but not wild-type strain and variant alpha, beta, and delta. The testing results were visualized by the naked eye under blue light **(L,N)**. The DNA plasmid of Omicron variant were used as positive control, while SARS-CoV-2 negative clinical samples infected with common human coronavirus (HCoV) 229E, HCoV OC43, and HCoV HKU1 as well as various other respiratory pathogens, including rhinovirus (HRV), respiratory syncytial virus (RSV) A and B, human metapneumovirus (HMPV), human parainfluenza virus (HPIV-1 and HPIV-4), human adenovirus (HAdV), and human bocavirus (HBoV) were used as negative controls to validate the specificity of our assay **(O,P)**. In all panels, error bars represent the mean ± standard deviation (SD) from three replicates of experiments. A two-tailed Mann–Whitney *U* test was used to analyze the fluorescence difference between on-target and off-target templates detected by CRISPR-Cas12a-based assay. ^***^P < 0.001; ^**^P < 0.01; *P < 0.05; ns, *P* > 0.05.

Unlike other SARS-CoV-2 VOCs, the Omicron variant carries multiple mutations at the S protein and RBD regions due to its high rate of mutation (Yu et al., 2022). We noticed that Omicron sublineages BA.1 and BA.2 have 37 and 31 mutations in the spike protein, respectively, and share multiple common mutations except for their unique mutations, which makes it possible to design Omicron-specific crRNAs to specifically diagnose Omicron infection and Omicron sublineage-specific crRNA to differentiate Omicron sublineages. After careful alignment analysis of SARS-CoV-2 Omicron sequences, we identified and designed two Omicron-specific crRNAs, i.e., crRNA-S-49X to cover Q493R, G496S, and Q498R mutations, and crRNA-S-50X to cover Q498R and N501Y mutations, respectively (Figure 5A). We predicted that the crRNA-S-49X can specifically detect the BA.1 variant because the G496S mutation is unique to Omicron sublineage BA.1, whereas the crRNA-S-50X can specifically diagnose Omicron infection. We also designed an RAA forward primer to cover the Omicron unique mutation S477N based on the target sequences to further increase the assay specificity.

By using crRNA-S-49X, our assay could specifically detect as low as 20 copies of Omicron BA.1 plasmid DNA per reaction without cross-reaction with 2 × 10^6^ copies of wild-type (Wuhan, China) or other SARS-CoV-2 VOCs plasmids (Figure 5B). Furthermore, the crRNA-S-49X could distinguish 200 copies of BA.1 plasmid DNA per reaction from 2 × 10^6^ copies of BA.2 plasmid DNA (Figure 5B). Both the quantitative results (Figure 5C) and the testing results judged by the naked eye (Figure 5D) proved the super specificity of crRNA-S-49X for detecting the Omicron BA.1 template and for distinguishing it from Omicron BA.2, other SARS-CoV-2 VOC plasmids or wild-type plasmid templates.

As expected, the crRNA-S-50X could readily detect both Omicron BA.1 and BA.2 plasmids with a LOD of 200 copies per reaction based on the reaction curves (Figures 5E,H), the quantitative results (Figures 5F,I), and the results judged by the naked eye (Figures 5G,J). Of note, a stronger fluorescence signal was obtained for detecting BA.1 plasmid than BA.2 by using the crRNA-S-50X (Figures 5E,H), probably due to the two unique extra mutations (G496S and T547K) of BA.2 in the amplification products, especially the G496S mutation located at the PAM motifs of crRNA-S-50X, which may affect the PAM identification and the efficiency of crRNA-S-50X to trigger collateral cleavage capability of Cas proteins (Tang et al., 2019).

Furthermore, both crRNA-S-49X and crRNA-S-50X could specifically detect Omicron variant in 5 clinical samples infected with BA.1 sublineage and verified by NGS and distinguish Omicron variant from other SARS-CoV-2 strains including wild-type strain and the variants of alpha, beta, and delta isolated from COVID-19 patients according to the reaction curves (Figures 5K,M) or the results visualized by eyes (Figures 5L,N). No cross-reaction was found when detecting SARS-CoV-2 negative clinical samples infected with common respiratory pathogens (Figures 5O,P).

## Discussion

The continuous emergence and spread of SARS-CoV-2 variants manifest the importance of simple and rapid SARS-CoV-2 genotyping methods. We have previously reported a PCR/CRISPR-Cas12a-based approach to distinguish between SARS-CoV-2 wild-type and major VOCs (Liang et al., 2021). In this study, we further refined the genotyping platform by replacing PCR with isothermal amplification, optimizing the crRNAs and primer sequences for detecting major SARS-CoV-2 VOCs, including the Omicron variant and its two major sublineages BA.1 and BA.2, and integrating all reactions in one tube. The refined assay is more feasible for rapid detection and tracking of SARS-CoV-2 variants.

Compared to other isothermal amplification methods such as loop-mediated isothermal amplification (LAMP), RAA appears to be an appropriate technology for rapid detection (20-40 min) with relatively simple primer design and selection (Supplementary Table 6). The most important fact is that the low-amplification temperature (37–42°C) makes it possible to integrate RAA with CRISPR-Cas12a-based detection in one tube to simplify the assay operation and to avoid the inactivation of Cas protein during thermal cycling or potential contamination caused by amplification products. In addition, the results can be read out directly by the naked eye under a portable blue light imager. These new features of our RAA/CRISPR-Cas12a-based assay make it more feasible to be implemented as point-of-care testing, which is more suitable for use in resource-limited settings. Compared to previously reported RAA or RPA/CRISPR-based nucleic acid detection methods (Ai et al., 2019; Bai et al., 2019; Jiao et al., 2021), our assay showed a comparable detection time and a single-base specificity.

Different from the PCR/CRISPR-Cas12a-mediated assay, our RAA/CRISPR-Cas12a-mediated assay shows a wide range of LOD from 10 to 10^4^ copies/μl of plasmid DNA according to the crRNAs. Our results indicated that relatively high LOD was observed for the 5 crRNAs with a single signature mutation, whereas low LOD was obtained for the two Omicron-specific crRNAs in which multiple mutations are included, suggesting that crRNA sequences and the number of mismatches between crRNAs and the target sequences play an important role in determining the detection sensitivity. In addition, artificial PAM motifs may affect the efficiency of RAA amplification, which in turn decreases the detection sensitivity of RAA/CRISPR-Cas12a-mediated assay. This may explain the relatively lower LOD of the RAA/CRISPR-Cas12a-mediated assay than the PCR/CRISPR-Cas12a-mediated assay. However, our results indicated that the relatively high LOD of our RAA/CRISPR-Cas12a-mediated assay did not affect its sensitivity when detecting major SARS-CoV-2 variants (Table 1). Previous studies showed that virus titers ranged from 10^4^ to 10^8^ copies/μl for the majority of SARS-CoV-2 positive samples (Jones et al., 2020; Pan et al., 2020; Pujadas et al., 2020), suggesting that our assay is suitable for detecting most of the clinical samples.

Sequencing is still the gold standard technology to identify mutations and to determine genotypes. In this study, we further evaluated our assay performance by comparing the results with Sanger sequencing data, and observed a positive predictive value of 100.0% and a negative predictive value of 92.9–100.0% (Table 2). The preliminary data showed a concordance of 92.59% with the Sanger sequencing method. Meanwhile, 100.0% specificity was achieved by our assay since no cross-reaction was found when detecting other common respiratory pathogens (Figure 4B). Of note, when detecting the same panels of SARS-CoV-2 positive and negative clinical samples, the RAA-CRISPR-Cas12a-mediated assay is slightly better than our PCR-CRISPR-Cas12a-mediated assay (Liang et al., 2021). That could be due to the further optimization of crRNAs used in this study to improve their performance (see below).

Genotyping based on CRISPR-Cas technology is due to the specific binding of crRNAs and the target sequences to activate Cas enzymes for both sequence-specific cutting (in cis) and non-specific sequence cleavage (in trans). In other words, mismatches between the crRNAs and the target sequences will affect the *trans*-cleavage efficiency and the strength of detection signals. In our study, we noticed the relatively high background and inefficiency of some crRNAs in differentiating single point mutations when using crRNAs that only contain one mismatched nucleotide (Figure 2). Previous studies indicate that the efficiency of crRNAs to trigger the collateral cleavage capability of CRISPR-Cas proteins could be affected by the extra substitutions in crRNAs (Creutzburg et al., 2020; Huang et al., 2021), especially the mismatches adjacent to the target mutations or in the PAM proximal regions (Kang et al., 2020). Therefore, we designed a series of crRNAs with one extra additional mutation at the upstream or downstream of the original signature mutation of SARS-CoV-2 variants, and confirmed their capability to enhance the specific detection signal and decrease the non-specific reaction, which in turn improves the detection sensitivity and specificity. The selection of the primer sets and crRNAs will depend on the performance evaluation. This new strategy to design the primers and crRNAs makes our system more feasible to be improved for the detection of SARS-CoV-2 variants.

## Conclusion

We successfully developed an RAA/CRISPR-Cas12a-mediated assay to specifically distinguish major SARS-CoV-2 variants, including the prevalent delta and Omicron sublineages BA.1 and BA.2. All the reactions were conducted in one sealed tube without the need for complex equipment and facilities. The simple and rapid assay could be set up and implemented routinely in resource-limited settings. In the future, this assay can be further simplified and used for high-throughput multiplex screening combined with sophisticated microfluidic devices.

## Data Availability Statement

The original contributions presented in this study are included in the article/Supplementary Material, further inquiries can be directed to the corresponding authors.

## Author Contributions

ST, XD, HL, and YL contributed to conception and design of the study, participated in interpretation of the data, and critical revisions of the manuscript. LZ, BL, and JS collected and sorted the materials. JZ and HW contributed with literature support. HL and YL performed the statistical analysis. All authors contributed to manuscript revision, read, and approved the submitted version.

## Conflict of Interest

The authors declare that the research was conducted in the absence of any commercial or financial relationships that could be construed as a potential conflict of interest.

## Publisher’s Note

All claims expressed in this article are solely those of the authors and do not necessarily represent those of their affiliated organizations, or those of the publisher, the editors and the reviewers. Any product that may be evaluated in this article, or claim that may be made by its manufacturer, is not guaranteed or endorsed by the publisher.
