# Supplementary material for: Combination of Isothermal Recombinase-Aided Amplification and CRISPR-Cas12a-Mediated Assay for Rapid Detection of Major Severe Acute Respiratory Syndrome Coronavirus 2 Variants of Concern
Source: Front Microbiol. 2022 Jun 28;13:945133. doi: 10.3389/fmicb.2022.945133 (PMC9274097; doi:10.3389/fmicb.2022.945133)

Supplementary Material

Supplementary Table S1. Characteristics and genotyping results of 54 SARS-CoV-2 positive samples

Sample ID[‡]	Ct value (ORF1ab/N)	Gender	Age (years)	Infection stage[§]	Infection sources	Detection results*					Genotyping	
						417N	478K	484K	501N	614D	RAA-CRISPR detection[†]	Sanger Sequencing
**Sample A**	NA	NA	NA	NA	NA	N	N	N	P	P	Wild-type	Wild-type
**Sample B**	NA	NA	NA	NA	NA	N	N	N	N	N	Alpha variant	Alpha variant
**Sample C**	NA	NA	NA	NA	NA	P	N	P	N	N	Beta variant	Beta variant
**Sample D**	NA	NA	NA	NA	NA	N	P	N	P	N	Delta variant	Delta variant
**20SF5774**	21/21	Male	50	Mild	Philippines	N	N	N	P	P	Wild-type	Wild-type
**20SF5787**	21/22	Female	56	Moderate	Philippines	N	N	N	P	P	Wild-type	Wild-type
**20SF5788**	21/22	Male	48	Moderate	Philippines	N	N	N	P	P	Wild-type	Wild-type
**2021A-XG01774**	21/20	Male	46	Moderate	Iraq	N	N	N	N	N	Alpha variant	Alpha variant
**2021A-XG02064**	24/23	Male	27	Moderate	UAE	N	N	N	N	N	Alpha variant	Alpha variant
**2021A-XG03265**	16/16	Male	46	Asymptomatic	Djibouti	P	N	P	N	N	Beta variant	Beta variant
**2021A-XG03594**	17/16	Female	55	Moderate	France	N	N	N	N	N	Alpha variant	Alpha variant
**2021A-XG03595**	26/26	Female	34	Moderate	Zambia	N	N	N	N	N	Alpha variant	Alpha variant
**2021A-XG03596**	15/15	Male	34	Moderate	Zambia	N	N	N	N	N	Alpha variant	Alpha variant
**2021A-XG03607**	18/16	Female	49	Moderate	Morocco	N	N	N	N	N	Alpha variant	Alpha variant

**2021A-XG03623**	16/15	Female	71	Moderate	Panama	N	N	N	N	N	Alpha variant	Alpha variant
**2021A-XG04560**	**18/16**	**Male**	**44**	Moderate	Iraq	**N**	**N**	**P**	**N**	**N**	Uncertain	**Alpha variant**
**2021A-XG04704**	18/17	Female	23	Moderate	Oman	N	N	N	N	N	Alpha variant	Alpha variant
**2021A-XG04850**	21/22	Male	31	Moderate	Cambodia	N	N	N	N	N	Alpha variant	Alpha variant
**2021A-XG05194**	19/18	Male	44	Asymptomatic	China	N	N	N	N	N	Alpha variant	Alpha variant
**2021A-XG08048**	16/16	Male	46	Moderate	Turkey	N	N	N	N	N	Alpha variant	Alpha variant
**2021A-XG08145**	24/22	Male	40	Asymptomatic	Philippines	N	N	N	N	N	Alpha variant	Alpha variant
**2021A-XG08337**	17/16	Female	29	Asymptomatic	China	N	N	N	N	N	Alpha variant	Alpha variant
**2021A-XG02289**	23/23	Male	35	Moderate	UAE	P	N	P	N	N	Beta variant	Beta variant
**2021A-XG03246**	18/18	Male	45	Mild	Djibouti	P	N	P	N	N	Beta variant	Beta variant
**2021A-XG03255**	19/19	Male	29	Mild	Djibouti	P	N	P	N	N	Beta variant	Beta variant
**2021A-XG03259**	24/23	Male	34	Mild	Turkey	N	N	N	N	N	Alpha variant	Alpha variant
**2021A-XG03240**	14/13	Male	41	Mild	Djibouti	P	N	P	N	N	Beta variant	Beta variant
**2021A-XG05824**	18/17	Female	17	Moderate	United States	P	N	P	N	N	Beta variant	Beta variant
**2021A-XG05827**	24/21	Female	17	Mild	United States	P	N	P	N	N	Beta variant	Beta variant
**2021A-XG06000**	23/23	Male	29	Moderate	Philippines	P	N	P	N	N	Beta variant	Beta variant
**2021A-XG06386**	15/16	Male	34	Moderate	Tanzania	P	N	P	N	N	Beta variant	Beta variant
**2021A-XG06552**	27/28	Female	42	Moderate	South Africa	P	N	P	N	N	Beta variant	Beta variant
**2021A-XG06750**	26/26	Female	38	Asymptomatic	Malaysia	P	N	P	N	N	Beta variant	Beta variant
**2021A-**	27/26	Female	27	Asymptomatic	Philippines	P	N	P	N	N	Beta variant	Beta variant

**XG07753**												
**2021A-XG07933**	22/22	Male	35	Asymptomatic	Philippines	P	N	P	N	N	Beta variant	Beta variant
**2021A-XG08520**	15/13	Male	31	Moderate	Iraq	N	P	N	P	N	Delta variant	Delta variant
**2021A-XG08527**	19/18	Male	50	Moderate	Malaysia	N	P	N	P	N	Delta variant	Delta variant
**2021A-XG08542**	23/22	Female	37	Asymptomatic	Malaysia	N	P	N	P	N	Delta variant	Delta variant
**2021A-XG08547**	20/19	Male	30	Mild	Turkey	N	P	N	P	N	Delta variant	Delta variant
**2021A-XG08859**	18/19	Male	32	Moderate	Iran	N	P	N	P	N	Delta variant	Delta variant
**2021A-XG08905**	**24/22**	**Female**	**34**	Moderate	Philippines	**P**	**P**	**N**	**P**	**N**	Uncertain	**Delta plus variant**
**2021A-XG09082**	16/17	Male	29	Moderate	UAE	N	P	N	P	N	Delta variant	Delta variant
**2021A-XG09089**	**30/31**	**Female**	**45**	Moderate	Kenya	**N**	**P**	**N**	**N**	**N**	Uncertain	**Delta variant**
**2021A-XG08688**	21/21	Female	49	Moderate	Malaysia	N	P	N	P	N	Delta variant	Delta variant
**2021A-XG08689**	15/15	Female	32	Moderate	Kenya	N	P	N	P	N	Delta variant	Delta variant
**2021A-XG08692**	16/16	Male	50	Moderate	DRC	N	P	N	P	N	Delta variant	Delta variant
**2021A-XG08693**	16/16	Male	28	Moderate	Kenya	N	P	N	P	N	Delta variant	Delta variant
**2021A-XG08694**	18/16	Female	31	Moderate	United States	N	P	N	P	N	Delta variant	Delta variant
**2021A-XG08715**	**30/30**	**Male**	**29**	Asymptomatic	Kenya	**N**	**P**	**N**	**N**	**N**	Uncertain	**Delta variant**
**Omicron-1**	23/25	Male	67	NA	Canada	P	P	N	N	N	Omicron variant	Omicron variant
**Omicron-2**	26/23	Female	64	NA	United States	P	P	N	N	N	Omicron variant	Omicron variant
**Omicron-3**	20/17	Male	28	NA	Ethiopia	P	P	N	N	N	Omicron variant	Omicron variant
**Omicron-4**	20/19	Female	70	NA	China	P	P	N	N	N	Omicron variant	Omicron variant

**Omicron-5**	25/25	Female	41	NA	China	P	P	N	N	N	Omicron variant	Omicron variant

Notes: NA, not available.

‡Sample A-D are SARS-CoV-2 isolates including wild-type strain (19A), variant Alpha (B1.1.7), Beta (B.1.351) and Delta (B1.617.2) grown in Vero cells. The other 50 SARS-CoV-2 positive clinical samples were oropharyngeal swab specimen collected from confirmed COVID-19 patients.

§The severity of illness was assessed according to the guideline for COVID-19 (version 6.0) published by the National Health Commission of China. Asymptomatic carriers present with no clinical symptoms but with a positive result of the pathogens tests of SARS-CoV-2 in respiratory tract specimens and so on; Mild patients have mild clinical symptoms and no pneumonia on chest imaging; Moderate patients have clinical symptoms (i.e. fever and respiratory tract symptoms) and pneumonia on chest imaging.

*N means negative result while P indicates positive result.

✝Uncertain means that genotype could not be determined based on our variant-typing algorithm.

Supplementary Table S2. Summary of DNA target sequences used for plasmid construction

Gene fragments	Sequence (5'→3')
**Wild-type S gene**	ATGTTTGTTTTTTTTGTTTATTGCCACTAGTCTCTAGTCAGTGTGTTAATCTTACAACCAGAACTCAATTACCCCCTGCATAC<br>ACTAATTCTTTCACACGTGGTGTTTATTACCCTGACAAAGTTTTCAGATCCTCAGTTTTACATTCAACTCAGGACTTGTTCTT<br>ACCTTTCTTTTCCAATGTTACTTGGTTCCATGCTATACATGTCTCTGGGACCAATGGTACTAAGAGGTTTGCTAACCCTGTCC<br>TACCATTTAATGATGGTGTTTATTTTGCTTCCACTGAGAAGTCTAACATAATAAGAGGCTGGATTTTTGGTACTACTTTAGATT<br>CGAAGACCCAGTCCCTACTTATTGTTAATAACGCTACTAATGTTGTTATTAAAGTCTGTGAATTTCAATTTTGTAATGATCCAT<br>TTTTGGGTGTTTATTACCACAAAAACAACAAAAGTTGGATGGAAAGTGAGTTCAGAGTTTATTCTAGTGCGAATAATTGCAC<br>TTTTGAATATGTCTCTCAGCCTTTTCTTATGGACCTTGAAGGAAAACAGGGTAATTTCAAAAATCTTAGGGAATTTGTGTTTA<br>AGAATATTGATGGTTATTTTAAAATATATTCTAAGCACACGCCTATTAATTTAGTGCGTGGTCTCCCTCAGGGTTTTTCGGCTT<br>TAGAACCATTGGTAGATTTGCCAATAGGTATTAACATCACTAGGTTTCAAACTTTACTTGCTTTACATATAAGTTATTTGACTC<br>CTGGTGATTCTTCTTCAGGTTGGACAGCTGGTGCTGCAGCTTATTATGTGGGTTATCTTCAACCTAGGACTTTTCTATTAAAA<br>TATAATGAAAATGGAACCATTACAGATGCTGTAGACTGTGCACTTGACCCTCTCTCAGAAACAAAGTGTACGTTGAAATCCT<br>TCACTGTAGAAAAAGGAATCTATCAAACTTCTAACTTTAGAGTCCAACCAACAGAATCTATTGTTAGATTTCCTAATATTACA<br>AACTTGTGCCCTTTTGGTGAAGTTTTTAACGCCACCAGATTTGCATCTGTTTATGCTTGGAACAGGAAGAGAATCAGCAACT<br>GTGTTGCTGATTATTCTGTCCTATATAATTCCGCATCATTTTCCACTTTTAAGTGTTATGGAGTGTCTCCTACTAAATTAAATGA<br>TCTCTGCTTTACTAATGTCTATGCAGATTCATTTGTAATTAGAGGTGATGAAGTCAGACAAATCGCTCCAGGGCAAACTGGA<br>AACATTGCTGATTATAATTATAAATTACCAGATGATTTTACAGGCTGCGTTATAGCTTGGAATTCTAACAATCTTGATTCTAAG<br>GTTGGTGGTAATTATAATTACCTGTTTAGATTGTTTAGGAAGTCTAATCTCAAACCTTTTGAGAGAGATATTTCAACTGAAAT<br>CTATCAGGCCGGTAGCACACCTTGTAATGGTGTTAAAGGTTTTAATTGTTACTTTCCTTTACAATCATATGGTTTCCAACCCAC<br>TTATGGTGTTGGTTACCAACCATACAGAGTAGTAGTACTTTCTTTTGAACTTCTACATGCACCAGCAACTGTTTGTGGACCTA<br>AAAAGTCTACTAATTTGGTTAAAAACAAATGTGTCAATTTCAACTTCAATGGTTTAACAGGCACAGGTGTTCTTACTGAGTC<br>TAACAAAAAGTTTCTGCCTTTCCAACAATTTGGCAGAGACATTGATGACACTACTGATGCTGTCCGTGATCCACAGACACTT<br>GAGATTCTTGACATTACACCATGTTCTTTTGGTGGTGTCAGTGTTATAACACCAGGAACAAATACTTCTAACCAGGTTGCTGT<br>TCTTTATCAGGGTGTTAACTGCACAGAAGTCCCTGTTGCTATTCATGCAGATCAACTTACTCCTACTTGGCGTGTTTATTCTAC<br>AGGTTCTAATGTTTTTCAAACACGTGCAGGCTGTTTAATAGGGGCTGAACATGTCAACAACTCATATGAGTGTGACATACCC<br>ATTGGTGCAGGTATATGCGCTAGTTATCAGACTCAGACTAATTCTCATCGGCGGGCACGTAGTGTAGCTAGTCAATCCATCAT<br>TGCCTACACTATGTCACTTGGTGTAGAAAATTCAGTTGCTTACTCTAATAACTCTATTGCCATACCCATAAATTTTACTATTAGT<br>GTTACCACAGAAATTCTACCAGTGTCTATGACCAAGACATCAGTAGATTGTACAATGTACATTTGTGGTGATTCAACTGAATG<br>CAGCAATCTTTTGTTGCAATATGGCAGTTTTTGTACACAATTAAACCGTGCTTTAACTGGAATAGCTGTTGAACAAGACAAA<br>AACACCCAAGAAGTTTTTGCACAAGTCAAACAAATTTACAAAACACCACCAATTAAAGATTTTGGTGGTTTTAATTTTTCAC<br>AAATATTACCAGATCCATCAAAACCAAGCAAGAGGTCATTTATTGAAGATCTACTTTTCAACAAAGTGACACTTGCAGATGC<br>TGGCTTCATCAAACAATATGGTGATTGCCTTGGTGATATTGCTGCTAGAGACCTCATTTGTGCACAAAAGTTTAACGGCCTTA<br>CTGTTTTGCCACCTTTGCTCACAGATGAAATGATTGCTCAATACACTTCTGCACTGTTAGCGGGTACAATCACTTCTGGTTGG<br>ACCTTTGGTGCAGGTGCTGCATTACAAATACCATTTGCTATGCAAATGGCTTATAGGTTTAATGGTATTGGAGTTACACAGAA<br>TGTTCTCTATGAGAACCAAAAATTGATTGCCAACCAATTTAATAGTGCTATTGGCAAAATTCAAGACTCACTTTCTTCCACAG

	CAAGTGCACTTGGAAAACTTCAAGATGTGGTCAACCAAAATGCACAAGCTTTAAACACGCTTGTTAAACAACTTAGCTCCA<br>ATTTTGGTGCAATTTCAAGTGTTTAAATGATATCCTTGCACGTCTTGACAAAGTTGAGGCTGAAGTGCAAATTGATAGGTT<br>GATCACAGGCAGACTTCAAAGTTTGCAGACATATGTGACTCAACAATTAATTAGAGCTGCAGAAATCAGAGCTTCTGCTAAT<br>CTTGCTGCTACTAAAATGTCAGAGTGTGTACTTGGACAATCAAAAAGAGTTGATTTTTGTGGAAAGGGCTATCATCTTATGT<br>CCTTCCCTCAGTCAGCACCTCATGGTGTAGTCTTCTTGCATGTGACTTATGTCCCTGCACAAGAAAAGAACTTCACAACTGC<br>TCCTGCCATTTGTCATGATGGAAAAGCACACTTTCCTCGTGAAGGTGTCTTTGTTTCAAATGGCACACACTGGTTTGTAACA<br>CAAAGGAATTTTTATGAACCACAAATCATTACTACACACAACACATTTGTGTCTGGTAACTGTGATGTTGTAATAGGAATTGT<br>CAACAACACAGTTTATGATCCTTTGCAACCTGAATTAGACTCATTCAAGGAGGAGTTAGATAAATATTTTAAGAATCATACAT<br>CACCAGATGTTGATTTAGGTGACATCTCTGGCATTAATGCTTCAGTTGTAAACATTCAAAAAGAAATTGACCGCCTCAATGA<br>GGTTGCCAAGAATTTAAATGAATCTCTCATCGATCTCCAAGAACTTGGAAAGTATGAGCAGTATATAAAATGGCCATGGTAC<br>ATTTGGCTAGGTTTTATAGCTGGCTTGATTGCCATAGTAATGGTGACAATTATGCTTTGCTGTATGACCAGTTGCTGTAGTTGT<br>CTCAAGGGCTGTTGTTCTTGTGGATCCTGCTGCAAATTTGATGAAGACGACTCTGAGCTAGTGCTCAAAGGAGTCAAATTA<br>CATTACACATAA
**Mutant S gene**	ATGTTTGTTTTTTTTGTTTATTGCCACTAGTCTCTAGTCAGTGTGTTAATCTTACAACCAGAACTCAATTACCCCCTGCATAC<br>ACTAATTCTTTCACACGTGGTGTTTATTACCCTGACAAAGTTTTCAGATCCTCAGTTTTACATTCAACTCAGGACTTGTTCTT<br>ACCTTTCTTTTCCAATGTTACTTGGTTCCATGCTATACATGTCTCTGGGACCAATGGTACTAAGAGGTTTGCTAACCCTGTCC<br>TACCATTTAATGATGGTGTTTATTTTGCTTCCACTGAGAAGTCTAACATAATAAGAGGCTGGATTTTTGGTACTACTTTAGATT<br>CGAAGACCCAGTCCCTACTTATTGTTAATAACGCTACTAATGTTGTTATTAAAGTCTGTGAATTTCAATTTTGTAATGATCCAT<br>TTTTGGGTGTTTATTACCACAAAAACAACAAAAGTTGGATGGAAAGTGAGTTCAGAGTTTATTCTAGTGCGAATAATTGCAC<br>TTTTGAATATGTCTCTCAGCCTTTTCTTATGGACCTTGAAGGAAAACAGGGTAATTTCAAAAATCTTAGGGAATTTGTGTTTA<br>AGAATATTGATGGTTATTTTAAAATATATTCTAAGCACACGCCTATTAATTTAGTGCGTGGTCTCCCTCAGGGTTTTTCGGCTT<br>TAGAACCATTGGTAGATTTGCCAATAGGTATTAACATCACTAGGTTTCAAACTTTACTTGCTTTACATATAAGTTATTTGACTC<br>CTGGTGATTCTTCTTCAGGTTGGACAGCTGGTGCTGCAGCTTATTATGTGGGTTATCTTCAACCTAGGACTTTTCTATTAAAA<br>TATAATGAAAATGGAACCATTACAGATGCTGTAGACTGTGCACTTGACCCTCTCTCAGAAACAAAGTGTACGTTGAAATCCT<br>TCACTGTAGAAAAAGGAATCTATCAAACTTCTAACTTTAGAGTCCAACCAACAGAATCTATTGTTAGATTTCCTAATATTACA<br>AACTTGTGCCCTTTTGGTGAAGTTTTTAACGCCACCAGATTTGCATCTGTTTATGCTTGGAACAGGAAGAGAATCAGCAACT<br>GTGTTGCTGATTATTCTGTCCTATATAATTCCGCATCATTTTCCACTTTTAAGTGTTATGGAGTGTCTCCTACTAAATTAAATGA<br>TCTCTGCTTTACTAATGTCTATGCAGATTCATTTGTAATTAGAGGTGATGAAGTCAGACAAATCGCTCCAGGGCAAACTGGA<br>AATATTGCTGATTATAATTATAAATTACCAGATGATTTTACAGGCTGCGTTATAGCTTGGAATTCTAACAATCTTGATTCTAAGG<br>TTGGTGGTAATTATAATTACCGGTTTAGATTGTTTAGGAAGTCTAATCTCAAACCTTTTGAGAGAGATATTTCAACTGAAATC<br>TATCAGGCCGGTAGCAAACCTTGTAATGGTGTTCAAGGTTTTAATTGTTACTTTCCTTTACAATCATATGGTTTCCAACCCACT<br>TATGGTGTTGGTTACCAACCATACAGAGTAGTAGTACTTTCTTTTGAACTTCTACATGCACCAGCAACTGTTTGTGGACCTAA<br>AAAGTCTACTAATTTGGTTAAAAACAAATGTGTCAATTTCAACTTCAATGGTTTAACAGGCACAGGTGTTCTTACTGAGTCT<br>AACAAAAAGTTTCTGCCTTTCCAACAATTTGGCAGAGACATTGATGACACTACTGATGCTGTCCGTGATCCACAGACACTT<br>GAGATTCTTGACATTACACCATGTTCTTTTGGTGGTGTCAGTGTTATAACACCAGGAACAAATACTTCTAACCAGGTTGCTGT<br>TCTTTATCAGGGTGTTAACTGCACAGAAGTCCCTGTTGCTATTCATGCAGATCAACTTACTCCTACTTGGCGTGTTTATTCTAC<br>AGGTTCTAATGTTTTTCAAACACGTGCAGGCTGTTTAATAGGGGCTGAACATGTCAACAACTCATATGAGTGTGACATACCC<br>ATTGGTGCAGGTATATGCGCTAGTTATCAGACTCAGACTAATTCTCATCGGCGGGCACGTAGTGTAGCTAGTCAATCCATCAT<br>TGCCTACACTATGTCACTTGGTGTAGAAAATTCAGTTGCTTACTCTAATAACTCTATTGCCATACCCATAAATTTTACTATTAGT

	GTTACCACAGAAATTCTACCAGTGTCTATGACCAAGACATCAGTAGATTGTACAATGTACATTTGTGGTGATTCAACTGAATG<br>CAGCAATCTTTTGTTGCAATATGGCAGTTTTTGTACACAATTAAACCGTGCTTTAACTGGAATAGCTGTTGAACAAGACAAA<br>AACACCCAAGAAGTTTTTGCACAAGTCAAACAAATTTACAAAACACCACCAATTAAAGATTTTGGTGGTTTTAATTTTTCAC<br>AAATATTACCAGATCCATCAAAACCAAGCAAGAGGTCATTTATTGAAGATCTACTTTTCAACAAAGTGACACTTGCAGATGC<br>TGGCTTCATCAAACAATATGGTGATTGCCTTGGTGATATTGCTGCTAGAGACCTCATTTGTGCACAAAAGTTTAACGGCCTTA<br>CTGTTTTGCCACCTTTGCTCACAGATGAAATGATTGCTCAATACACTTCTGCACTGTTAGCGGGTACAATCACTTCTGGTTGG<br>ACCTTTGGTGCAGGTGCTGCATTACAAATACCATTTGCTATGCAAATGGCTTATAGGTTTAATGGTATTGGAGTTACACAGAA<br>TGTTCTCTATGAGAACCAAAAATTGATTGCCAACCAATTTAATAGTGCTATTGGCAAAATTCAAGACTCACTTTCTTCCACAG<br>CAAGTGCACTTGGAAAACTTCAAGATGTGGTCAACCAAAATGCACAAGCTTTAAACACGCTTGTTAAACAACTTAGCTCCA<br>ATTTTGGTGCAATTTCAAGTGTTTTAAATGATATCCTTGCACGTCTTGACAAAGTTGAGGCTGAAGTGCAAATTGATAGGTT<br>GATCACAGGCAGACTTCAAAGTTTGCAGACATATGTGACTCAACAATTAATTAGAGCTGCAGAAATCAGAGCTTCTGCTAAT<br>CTTGCTGCTACTAAAATGTCAGAGTGTGTACTTGGACAATCAAAAAGAGTTGATTTTTGTGGAAAGGGCTATCATCTTATGT<br>CCTTCCCTCAGTCAGCACCTCATGGTGTAGTCTTCTTGCATGTGACTTATGTCCCTGCACAAGAAAAGAACTTCACAACTGC<br>TCCTGCCATTTGTCATGATGGAAAAGCACACTTTCCTCGTGAAGGTGTCTTTGTTTCAAATGGCACACACTGGTTTGTAACA<br>CAAAGGAATTTTTATGAACCACAAATCATTACTACACACAACACATTTGTGTCTGGTAACTGTGATGTTGTAATAGGAATTGT<br>CAACAACACAGTTTATGATCCTTTGCAACCTGAATTAGACTCATTCAAGGAGGAGTTAGATAAATATTTTAAGAATCATACAT<br>CACCAGATGTTGATTTAGGTGACATCTCTGGCATTAATGCTTCAGTTGTAAACATTCAAAAAGAAATTGACCGCCTCAATGA<br>GGTTGCCAAGAATTTAAATGAATCTCTCATCGATCTCCAAGAACTTGGAAAGTATGAGCAGTATATAAAATGGCCATGGTAC<br>ATTTGGCTAGGTTTTATAGCTGGCTTGATTGCCATAGTAATGGTGACAATTATGCTTTGCTGTATGACCAGTTGCTGTAGTTGT<br>CTCAAGGGCTGTTGTTCTTGTGGATCCTGCTGCAAATTTGATGAAGACGACTCTGAGCTAGTGCTCAAAGGAGTCAAATTA<br>CATTACACATAA
**Omicron BA.1 S gene**	ATGTTTGTTTTTCTTGTTTTATTGCCACTAGTCTCTAGTCAGTGTGTTAATCTTACAACCAGAACTCAATTACCCCCTGC<br>ATACACTAATTCTTTCACACGTGGTGTTTATTACCCTGACAAAGTTTTCAGATCCTCAGTTTTACATTCAACTCAGGACT<br>TGTTCTTACCTTTCTTTTCCAATGTTACTTGGTTCCATGTTATACATGTCTCTGGGACCAATGGTACTAAGAGGTTTGAT<br>AACCCTGTCCTACCATTTAATGATGGTGTTTATTTTGCTTCCATTGAGAAGTCTAACATAATAAGAGGCTGGATTTTTGG<br>TACTACTTTAGATTCGAAGACCCAGTCCCTACTTATTGTTAATAACGCTACTAATGTTGTTATTAAAGTCTGTGAATTTC<br>AATTTTGTAATGATCCATTTTTGGGTGTTTATGACCACAAAAACAACAAAAGTTGGATGGAAAGTGAGTTCAGAGTTTAT<br>TCTAGTGCGAATAATTGCACTTTTGAATATGTCTCTCAGCCTTTTCTTATGGACCTTGAAGGAAAACAGGGTAATTTCAA<br>AAATCTTAGGGAATTTGTGTTTAAGAATATTGATGGTTATTTTAAAATATATTCTAAGCACACGCCTATTAATATAGTGC<br>GTGATCTCCCTCAGGGTTTTTCGGCTTTAGAACCATTGGTAGATTTGCCAATAGGTATTAACATCACTAGGTTTCAAACT<br>TTACTTGCTTTACATAGAAGTTATTTGACTCCTGGTGATTCTTCTTCAGGTTGGACAGCTGGTGCTGCAGCTTATTATGT<br>GGGTTATCTTCAACCTAGGACTTTTCTATTAAAATATAATGAAAATGGAACCATTACAGATGCTGTAGACTGTGCACTTG<br>ACCCTCTCTCAGAAACAAAGTGTACGTTGAAATCCTTCACTGTAGAAAAAGGAATCTATCAAACTTCTAACTTTAGAGTC<br>CAACCAACAGAATCTATTGTTAGATTTCCTAATATTACAAACTTGTGCCCTTTTGATGAAGTTTTTAACGCCACCAGATT<br>TGCATCTGTTTATGCTTGGAACAGGAAGAGAATCAGCAACTGTGTTGCTGATTATTCTGTCCTATATAATCTCGCACCAT<br>TTTTCACTTTTAAGTGTTATGGAGTGTCTCCTACTAAATTAAATGATCTCTGCTTTACTAATGTCTATGCAGATTCATTT<br>GTAATTAGAGGTGATGAAGTCAGACAAATCGCTCCAGGGCAAACTGGAAATATTGCTGATTATAATTATAAATTACCAGA<br>TGATTTTACAGGCTGCGTTATAGCTTGGAATTCTAACAAACTTGATTCTAAGGTTAGTGGTAATTATAATTACCTGTATA<br>GATTGTTTAGGAAGTCTAATCTCAAACCTTTTGAGAGAGATATTTCAACTGAAATCTATCAGGCCGGTAACAAACCTTGT

	AATGGTGTTGCAGGTTTAATTGTTACTTTCCTTTACGATCATATAGTTTCCGACCCACTTATGGTGTTGGTCACCAACC<br>ATACAGAGTAGTAGTACTTTCTTTTGAACTTCTACATGCACCAGCAACTGTTTGTGGACCTAAAAAGTCTACTAATTTGG<br>TTAAAAACAAATGTGTCAATTTCAACTTCAATGGTTTAAAAGGCACAGGTGTTCTTACTGAGTCTAACAAAAAGTTTCTG<br>CCTTTCCAACAATTTGGCAGAGACATTGCTGACACTACTGATGCTGTCCGTGATCCACAGACACTTGAGATTCTTGACAT<br>TACACCATGTTCTTTTGGTGGTGTCAGTGTTATAACACCAGGAACAAATACTTCTAACCAGGTTGCTGTTCTTTATCAGG<br>GTGTTAACTGCACAGAAGTCCCTGTTGCTATTCATGCAGATCAACTTACTCCTACTTGGCGTGTTTATTCTACAGGTTCT<br>AATGTTTTTCAAACACGTGCAGGCTGTTTAATAGGGGCTGAATATGTCAACAACTCATATGAGTGTGACATACCCATTGG<br>TGCAGGTATATGCGCTAGTTATCAGACTCAGACTAAATCTCATCGGCGGGCACGTAGTGTAGCTAGTCAATCCATCATTG<br>CCTACACTATGTCACTTGGTGCAGAAAATTCAGTTGCTTACTCTAATAACTCTATTGCCATACCCACAAATTTTACTATT<br>AGTGTTACCACAGAAATTCTACCAGTGTCTATGACCAAG
**Omicron BA.2 S gene**	ATGTTTGTTTTTCTTGTTTTATTGCCACTAGTCTCTAGTCAGTGTGTTAATCTTATAACCAGAACTCAATCATACACTAA<br>TTCTTTCACACGTGGTGTTTATTACCCTGACAAAGTTTTCAGATCCTCAGTTTTACATTCAACTCAGGACTTGTTCTTAC<br>CTTTCTTTTCCAATGTTACTTGGTTCCATGCTATACATGTCTCTGGGACCAATGGTACTAAGAGGTTTGATAACCCTGTC<br>CTACCATTTAATGATGGTGTTTATTTTGCTTCCACTGAGAAGTCTAACATAATAAGAGGCTGGATTTTTGGTACTACTTT<br>AGATTCGAAGACCCAGTCCCTACTTATTGTTAATAACGCTACTAATGTTGTTATTAAAGTCTGTGAATTTCAATTTTGTA<br>ATGATCCATTTTTGGATGTTTATTACCACAAAAACAACAAAAGTTGGATGGAAAGTGAGTTCAGAGTTTATTCTAGTGCG<br>AATAATTGCACTTTTGAATATGTCTCTCAGCCTTTTCTTATGGACCTTGAAGGAAAACAGGGTAATTTCAAAAATCTTAG<br>GGAATTTGTGTTTAAGAATATTGATGGTTATTTTAAAATATATTCTAAGCACACGCCTATTAATTTAGGGCGTGATCTCC<br>CTCAGGGTTTTTCGGCTTTAGAACCATTGGTAGATTTGCCAATAGGTATTAACATCACTAGGTTTCAAACTTTACTTGCT<br>TTACATAGAAGTTATTTGACTCCTGGTGATTCTTCTTCAGGTTGGACAGCTGGTGCTGCAGCTTATTATGTGGGTTATCT<br>TCAACCTAGGACTTTTCTATTAAAATATAATGAAAATGGAACCATTACAGATGCTGTAGACTGTGCACTTGACCCTCTCT<br>CAGAAACAAAGTGTACGTTGAAATCCTTCACTGTAGAAAAAGGAATCTATCAAACTTCTAACTTTAGAGTCCAACCAACAG<br>AATCTATTGTTAGATTTCCTAATATTACAAACTTGTGCCCTTTTGATGAAGTTTTTAACGCCACCAGATTTGCATCTGT<br>TTATGCTTGGAACAGGAAGAGAATCAGCAACTGTGTTGCTGATTATTCTGTCCTATATAATTTCGCACCATTTTTCGCTT<br>TTAAGTGTTATGGAGTGTCTCCTACTAAATTAAATGATCTCTGCTTTACTAATGTCTATGCAGATTCATTTGTAATTAGA<br>GGTAATGAAGTCAGCCAAATCGCTCCAGGGCAAACTGGAAATATTGCTGATTATAATTATAAATTACCAGATGATTTTAC<br>AGGCTGCGTTATAGCTTGGAATTCTAACAAGCTTGATTCTAAGGTTGGTGGTAATTATAATTACCTGTATAGATTGTTTA<br>GGAAGTCTAATCTCAAACCTTTTGAGAGAGATATTTCAACTGAAATCTATCAGGCCGGTAACAAACCTTGTAATGGTGTT<br>GCAGGTTTAATTGTTACTTTCCTTTACGATCATATGGTTTCCGACCCACTTATGGTGTTGGTCACCAACCATACAGAGT<br>AGTAGTACTTTCTTTTGAACTTCTACATGCACCAGCAACTGTTTGTGGACCTAAAAAGTCTACTAATTTGGTTAAAAACA<br>AATGTGTCAATTTCAACTTCAATGGTTTAACAGGCACAGGTGTTCTTACTGAGTCTAACAAAAAGTTTCTGCCTTTCCAA<br>CAATTTGGCAGAGACATTGCTGACACTACTGATGCTGTCCGTGATCCACAGACACTTGAGATTCTTGACATTACACCATG<br>TTCTTTTGGTGGTGTCAGTGTTATAACACCAGGAACAAATACTTCTAACCAGGTTGCTGTTCTTTATCAGGGTGTTAACT<br>GCACAGAAGTCCCTGTTGCTATTCATGCAGATCAACTTACTCCTACTTGGCGTGTTTATTCTACAGGTTCTAATGTTTTT<br>CAAACACGTGCAGGCTGTTTAATAGGGGCTGAATATGTCAACAACTCATATGAGTGTGACATACCCATTGGTGCAGGTAT<br>ATGCGCTAGTTATCAGACTCAGACTAAGTCTCATCGGCGGGCACGTAGTGTAGCTAGTCAATCCATCATTGCCTACACTA<br>TGTCACTTGGTGCAGAAAATTCAGTTGCTTACTCTAATAACTCTATTGCCATACCCACAAATTTTACTATTAGTGTTACC<br>ACAGAAATTCTACCAGTGTCTATGACCAAG

Note: Wild-type S gene, gene fragment of SARS-CoV-2 spike protein (nt 21,563-25,384, NCBI accession number MN908947); Mutant S

gene, gene fragments of SARS-CoV-2 spike protein including mutations: L5F, D80A, D215G, R246I, K417N, L452R, Y453F, T478K, E484Q, N501Y, A570D, D614G, P681H, A701V, T716I, S982A, D1118H, P1263L. Omicron S gene, gene fragment of SARS-CoV-2 spike protein including mutations:A67V, 69/70del, T95I, 142/144del, Y145D, 211del, L212I, G339D, S371L, S373P, S375F, K417N, N440K, G446S, S477N, T478K, E484A, Q493R, G496S, Q498R, N501Y, Y505H, T547K, D614G, H655Y, N679K, P681H, N764K, D796Y, N856K, Q954H, N969K, L981F.

Supplementary Table S3. Detection of major SARS-CoV-2 variants of concern based on the RAA/CRISPR-Cas12a-medicated assay.

SARS-CoV-2	Location	Signature mutations in spike protein	CRISPR-Cas12a-based assay using crRNAs for				
			417N	478K	484K	501N	614D
**Wild type**	China	NA*	-	-	-	+	+
**Alpha (B.1.1.7)**	United Kingdom	N501Y, A570D, D614G	-	-	-	-	-
**Beta (B.1.351)**	South Africa	K417N, E484K, N501Y, D614G	+	-	+	-	-
**Delta (B.1.617.2)**	India	L452R, T478K, D614G	-	+	-	+	-
**Omicron (B.1.1.529)**	South Africa	K417N, T478K, E484A, N501Y, D614G, Q493R, G496S, Q498R	+	+	-	-	-

NA* , not applicable; +, robust fluorescence; -, mild or no fluorescence intensity

Supplementary Table S4. Summary of the crRNA and RAA primers sequences based on the target sites of SARS-COV-2 S gene in our study.

Mutations of Spike protein	RAA primers	Sequences (5'→3')	[a]Primer location(nt)
**K417N**	**417F-1**	**GAAGTCAGACAAATCGCTCCAGGGCAAATTTGAA**	**22778-22811**
	417F-2	AAGTCAGACAAATCGCTTTAGGGCAAACTGGA	22779-22810
	**R1**	**GGCCTGATAGATTTCAGTTGAAATATCTCTCT**	**22956-22987**
**T478K**	**478F-1**	**TATTTCAACTGAAATCTATCAGGCCGTTTGCA**	**22963-22994**
	478F-2	AGATATTTCAACTGAAATCTTTCAGGCCGGTAG	22960-22992
**E484K**	484F-1	TCTATCAGGCCGGTAGCACACTTTGTAATGGTG	22977-23009
	**484F-2**	**TCAGGCCGGTAGCACACCTTTTAATGGTGTT**	**22981-23011**
**T478K/ E484K**	F1	TTGAGAGAGATATTTCAACTGAAATCTATCAG	22953-22984
	**R2**	**TTGTTAGACTCAGTAAGAACACCTGTGCCTGT**	**23201-23232**
**N501Y/D614G**	**F2**	**TAGGAAGTCTAATCTCAAACCTTTTGAGAGAG**	**22930-22961**
	**R3**	**AGTAAGTTGATCTGCATGAATAGCAACAGGGA**	**23421-23452**
**Q493R, G496S, Q498R, N501Y**	**Omicron-F**	**CCGGTAACAAACCTTGTAATGGTGTTGCAGGT**	**22986-23017**
	**Omicron-R**	**ATCACGGACAGCATCAGTAGTGTCAGCAATGTC**	**23264-23296**

Mutations of Spike protein	crRNA	Sequences (5'→3')	The corresponding Primer set(F/R)
**K417N**	crRNA-417N-1	UAAUUUCUACUAAGUGUAGAAAAUAUUGCUGAUUAUAAUU	417F-1/R1
	crRNA-417N-2	UAAUUUCUACUAAGUGUAGAAGGGCAAACUGGAAAUAUUG	417F-2/R1
	**crRNA-417N-3**	**UAAUUUCUACUAAGUGUAGAACAUAUUGCUGAUUAUAAUU**	417F-1/R1
	crRNA-417N-4	UAAUUUCUACUAAGUGUAGA AAAUAGUGCUGAUUAUAAUU	
**T478K**	crRNA-478K-1	UAAUUUCUACUAAGUGUAGAGCAAACCUUGUAAUGGUGUU	478F-1/R2
	crRNA-478K-2	UAAUUUCUACUAAGUGUAGAAGGCCGGUAGCAAACCUUGU	478F-2/R2
	crRNA-478K-3	UAAUUUCUACUAAGUGUAGAGCUACCGGCCUGAUAGAUUU	F1/R2
	crRNA-478K-4	UAAUUUCUACUAAGUGUAGACCAAACCUUGUAAUGGUGUU	
	**crRNA-478K-5**	**UAAUUUCUACUAAGUGUAGAGCAAAGCUUGUAAUGGUGUU**	478F-1/R2
	crRNA-478K-6	UAAUUUCUACUAAGUGUAGAGGAAACCUUGUAAUGGUGUU	
**E484K**	crRNA-484K-1	UAAUUUCUACUAAGUGUAGAUAAUGGUGUUAAAGGUUUUA	484F-1/R2
	**crRNA-484K-2**	**UAAUUUCUACUAAGUGUAGAAAUGGUGUUAAAGGUUUUAA**	484F-2/R2
	crRNA-484K-3	UAAUUUCUACUAAGUGUAGAAACACCAUUACAAGGUGUGCU	F1/R2
**N501Y**	crRNA-501N-1	UAAUUUCUACUAAGUGUAGACAACCCACUAAUGGUGUUGG	F2/R3
	crRNA-501N-2	UAAUUUCUACUAAGUGUAGACAACCCACUAAAGGUGUUGG	

	**crRNA-501N-3**	**UAAUUUCUACUAAGUGUAGACAACCCAGUAAUGGUGUUGG**	
	crRNA-501N-4	UAAUUUCUACUAAGUGUAGACAACCCACAAAUGGUGUUGG	
**D614G**	crRNA-614D-1	UAAUUUCUACUAAGUGUAGAUCAGGAUGUUAACUGCACAG	F2/R3
	**crRNA-614D-2**	**UAAUUUCUACUAAGUGUAGAUCAGGACGUUAACUGCACAG**	
**Q493R, G496S, Q498R**	**crRNA-S-49X**	**UAAUUUCUACUAAGUGUAGACGAUCAUAUAGUUUCCGACC**	Omicron F/R
**Q498R，N501Y**	**crRNA-S-50X**	**UAAUUUCUACUAAGUGUAGAUCGAACCACUUAUGGUGUUG**	Omicron F/R

Note: The protospacer adjacent motif (PAM) (blue highlight), the target positions (red font), and the introduced additional mismatch in crRNA (yellow highlight) were indicated. The optimal set of crRNAs and RAA primers were selected in RAA-CRISPR/Cas12a medicated assay are in bold.

[a]The location of the primers refers to the position in the genome of severe acute respiratory syndrome coronavirus 2 isolate Wuhan-Hu-1 (Genbank accession number MN908947).

Supplementary Table S5. Comparison of baseline characteristics for 50 SARS-CoV-2 infected patients by genotypes.

Characteristics	SARS-CoV-2 genotypes						Test statistic	*P* value*
	Wild-type (N=3)	Alpha variant (N=15)	Beta variant (N=13)	Delta variant (N=14)	Omicron variant (N=5)	Total (N=50)		
**Age, years (Median, IQR)**	50.0 (48.0-56.0)	40.0 (31.0-46.0)	35.0 (28.0-41.5)	32.0 (29.8-46.0)	64.0 (34.5-68.5)	36.0 (29.8-46.5)	9.560	0.049
**Gender**								
**Male**	2 (66.7%)	9 (60.0%)	8 (61.5%)	8 (57.1%)	2 (40.0%)	29 (58.0%)	1.097	0.958
**Female**	1 (33.3%)	6 (40.0%)	5 (38.5%)	6 (42.9%)	3 (60.0%)	21 (42.0%)		
**Infection stage**								
**Mild**	1 (33.3%)	1 (6.7%)	4 (30.8%)	1 (7.1%)	NA	7 (15.6%)		
**Moderate**	2 (66.7%)	11 (73.3%)	5 (38.5%)	11 (78.6%)	NA	29 (64.4%)	7.157	0.266
**Asymptomatic**	0 (0.0%)	3 (20.0%)	4 (30.8%)	2 (14.3%)	NA	9 (20.0%)		
**Ct value (Median, IQR)**								
**ORF 1a/b gene**	21.0 (21.0-21.0)	18.0 (17.0-24.0)	22.0 (17.0-25.0)	18.5 (16.0-23.3)	23.0 (20.0-25.5)	20.0 (17.0-24.0)	3.441	0.487
**N gene**	22.0 (21.0-22.0)	17.0 (16.0-22.0)	21.0 (16.0-24.0)	18.50 (16.0-22.0)	23.0 (18.0-25.0)	19.0 (16.0-23.0)	4.509	0.341

Notes: IQR, interquartile range. NA, not available.

* *P* value for noncategorical variables was calculated by Kruskal-Wallis test while the Fisher's exact test was used for categorical variables.

Supplementary Table S6. Comparison of different CRISPR-Cas12a-based approach in detecting SARS-CoV-2

Characteristics	PCR/CRISPR-Cas12	RPA/CRISPR-Cas12	LAMP/CRISPR-Cas12
**Limits of detection**	1-10 copies per μL	10-100 copies per μL	10-100 copies per μL
**Amplification plus Cas12 cleavage time**	1.5h-2h	40min-60min	1-1.5h
**Components**	dNTPs, DNA polymerase, and designated primer	Recombinase, single stranded binding protein, DNA polymerase, forward primer, reverse primer	Bst DNA polymerase, dNTPs, magnesium ions, betaine, and two pairs of primers
**Primer designs**	Specifically designed primer	Recombinase primer complex	Require 4 to 6 specific primers
**Estimated cost**	Around \$3	Around \$5	Around \$9
**Amplification temperature**	52-95°C,thermal cycles	37-42°C	60-65°C
**Benefits**	More sensitive and high specificity	Fast and sensitive, feasible for POCT	High specificity, feasible for POCT
**Challenges**	Time-consuming, equipment required	Non-specific/high background signal	Complicated design of primers

Figure Legends

Supplementary Figure S1. The expression and preparation of AsCas12a and LbCas12a protein. **(A)** Coomassie blue stained polyacrylamide gel of AsCas12a and LbCas12a proteins before purification. Lane 1, LbCas12a in E. coli (bacterium solution) before induction by IPTG. After induction by IPTG, LbCas12a in E. coli bacterium solution (Lane 2), supernatant (Lane 3) or precipitate (Lane 4). Lane 5, AsCas12a in E. coli (bacterium solution) before induction by IPTG. After induction by IPTG, AsCas12a in E. coli bacterium solution (Lane 6), supernatant (Lane 7) or precipitate (Lane 8). The molecular marker lanes (M) are shown on the right sides of the gel. **(B)** Western blots of AsCas12a and LbCas12a proteins. Lane 1, LbCas12a in E. coli precipitate. Lane 2, LbCas12a in E. coli supernatant. Lane 3, LbCas12a in E. coli bacterium solution. Lane 4, LbCas12a in E. coli bacterium solution without induction by IPTG. Lane 5, AsCas12a in E. coli precipitate. Lane 6, AsCas12a in E. coli supernatant. Lane 7, AsCas12a in E. coli bacterium solution. Lane 8, AsCas12a in E. coli bacterium solution without induction by IPTG. The molecular marker lanes are shown on the left sides. **(C)** Coomassie blue stained polyacrylamide gel of AsCas12a and LbCas12a proteins after purification. Lane 1, LbCas12a protein in storage buffer. Lane 2, AsCas12a protein in storage buffer. The molecular marker lanes (M) are shown on the left sides of the lane 1 and 2, respectively.

Supplemental Figure S2. Alignment of amino acid sequences of SARS-CoV-2 spike protein of major variants of concern. The spike protein sequences of major SARS-CoV-2 variants from different geographic locations were aligned and compared with the wild-type SARS-CoV-2 strain isolated from Wuhan, China (Genbank accession number MN908947). SARS-CoV-2 genomes used for alignment were download from NCBI Virus database. Multiple alignment was performed and visualized using MAFFT version 7 and ESPript 3.0 respectively. The accession number, pangolin lineage and WHO classification are shown on the left side of the sequences. The red boxes represent identical residues while similar residues are indicated by red letters.

Supplemental Figure S3. Demonstration of RAA/CRISPR-Cas12a mediated assay which the RAA and Cas12a detection step could be performed with similar temperature. The CRISPR reaction temperature were set at 37°C and 39°C for 40min respectively to test the collateral activity of AsCas12a complexed with 5 crRNAs specific for the amino acid 417N **(A)**, 478K **(B)**, 484K **(C)**, 501N **(D)**, and 614D **(E)** of SARS-CoV-2 spike protein, respectively. The fluorescence signal at 39°C were comparable to the signals obtained at 37°C in all panels. Fluorescence values are represented as mean ± standard deviation (SD) of three replicates. The fluorescence ratio of sample over control is presented at the top of each panel. The amino acid is indicated in the brackets. No input refers to no DNA template.

Supplemental Figure S4. Evaluation of the performance of optimal mutation-specific crRNAs in detecting the signature mutations in SARS-CoV-2 spike protein in clinical samples by RAA/CRISPR-Cas12a-mediated assay. A total of 54 SARS-CoV-2 positive clinical samples including 4 wild-type strains, 16 Alpha variants, 14 Beta variants, 15 Delta variants and 5 Omicron variants were detected using a set of crRNAs including crRNA-417N **(A)**, crRNA-478K **(B)**, crRNA-484K **(C)**, crRNA-501N **(D)** and crRNA-614D **(E)** specific for the corresponding mutations, respectively. In the Left panel, the crRNAs used and the corresponding amino acid residues detected are presented at the top of each panel while the number of samples tested was indicated at the bottom of each panel. Y axis shows the fluorescence value detected. X axis indicates the wild-type strain and 4 major variants of concern and the corresponding amino acids. Each solid circle represents the average fluorescence value of three independent experiments for each clinical sample. Solid lines represent the mean values and standard deviations (SD) of all samples analyzed. Dash line means the cut-off value, which was derived from the maximization of Youden index. Two-tailed Mann-Whitney U test was used to analyze the fluorescence difference between on-target and off-target template detected by the RAA/Cas12a-mediated assay. ns, $P>0.05$; *, $P<0.05$; **, $P<0.01$; ***, $P<0.001$; ****, $P<0.0001$. In the Right panel, receiver operating characteristic curve (ROC) was used for assessing diagnostic performance of crRNAs in RAA/CRISPR-Cas12a mediated assay including the sensitivity (Y axis) and specificity (X axis), and to calculate the area under the ROC curve (AUC) based on the presence or absence of the specific mutations. The most suitable threshold and the corresponding sensitivity and specificity based on the maximization of Youden index for each crRNA were indicated in the bottom of each panel.

Supplemental Figure S5. The testing results were visualized by naked eyes under blue light at 40 min after the RAA/CRISPR-Cas12a-mediated reaction. A total of 54 SARS-CoV-2 positive clinical samples in a panel of 4 wild-type strains, 16 Alpha variants, 14 Beta variants, 15 Delta variants and 5 Omicron variants were detected in RAA/CRISPR-Cas12a assay using a set of crRNAs including crRNA-417N, crRNA-478K, crRNA-484K, crRNA-501N and crRNA-614D, which are indicated in each tube. The genotyping result based on Sanger sequencing are labeled in the left of pictures and the Sample ID are indicated in the top left of each picture.

Supplemental Figure S1

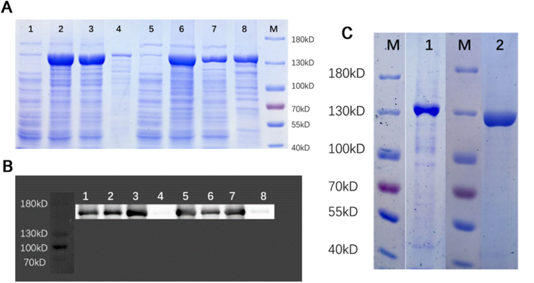

Supplemental Figure S2

```
                               1        10        20        30        40        50        60
MN908947/Wuhan-Hu-1            MFVFLVLLPLVSSQCVNLTTRTQLPPAYTNSFTRGVYYPDKVFRSSVLHSTQDLFLPFFS
BS001137/B.1.1.7/Alpha         MFVFLVLLPLVSSQCVNLTTRTQLPPAYTNSFTRGVYYPDKVFRSSVLHSTQDLFLPFFS
BS001138/B.1.1.7/Alpha         MFVFLVLLPLVSSQCVNLTTRTQLPPAYTNSFTRGVYYPDKVFRSSVLHSTQDLFLPFFS
BS001139/B.1.1.7/Alpha         MFVFLVLLPLVSSQCVNLTTRTQLPPAYTNSFTRGVYYPDKVFRSSVLHSTQDLFLPFFS
BS001140/B.1.1.7/Alpha         MFVFLVLLPLVSSQCVNLTTRTQLPPAYTNSFTRGVYYPDKVFRSSVLHSTQDLFLPFFS
MW913362/B.1.1.7/Alpha         MFVFLVLLPLVSSQCVNLTTRTQLPPAYTNSFTRGVYYPDKVFRSSVLHSTQDLFLPFFS
MZ266365/B.1.1.7/Alpha         MFVFLVLLPLVSSQCVNLTTRTQLPPAYTNSFTRGVYYPDKVFRSSVLHSTQDLFLPFFS
MZ350109/B.1.1.7/Alpha         MFVFLVLLPLVSSQCVNLTTRTQLPPAYTNSFTRGVYYPDKVFRSSVLHSTQDLFLPFFS
MZ888516/B.1.1.7/Alpha         MFVFLVLLPLVSSQCVNLTTRTQLPPAYTNSFTRGVYYPDKVFRSSVLHSTQDLFLPFFS
OU562607/B.1.1.7/Alpha         MFVFLVLLPLVSSQCVNLTTRTQLPPAYTNSFTRGVYYPDKVFRSSVLHSTQDLFLPFFS
OU514166/B.1.1.7/Alpha         MFVFLVLLPLVSSQCVNLTTRTQLPPAYTNSFTRGVYYPDKVFRSSVLHSTQDLFLPFFS
OU538849/B.1.1.7/Alpha         MFVFLVLLPLVSSQCVNLTTRTQLPPAYTNSFTRGVYYPDKVFRSSVLHSTQDLFLPFFS
MW981442/B.1.351/Beta          MFVFLVLLPLVSSQCVNFTTRTQLPPAYTNSFTRGVYYPDKVFRSSVLHSTQDLFLPFFS
MZ298839/B.1.351/Beta          MFVFLVLLPLVSSQCVNLTTRTQLPPSYTNSFTRGVYYPDKVFRSSVLHSTQDLFLPFFS
MZ913100/B.1.351/Beta          MFVFLVLLPLVSSQCVNFTTRTQLPPAYTNSFTRGVYYPDKVFRSSVLHSTQDLFLPFFS
MZ879365/B.1.351/Beta          MFVFLVLLPLVSSQCVNFTTRTQLLPAYTNSFTRGVYYPDKVFRSSVLHSTQDLFLPFFS
OU539352/B.1.351/Beta          MFVFLVLLPLVSSQCVNLTTRTQLPPAYTNSFTRGVYYPDKVFRSSVLHSTQDLFLPFFS
MZ202314/B.1.351/Beta          MFVFLVLLPLVSSQCVNFTTRTQLPPAYTNSFTRGVYYPDKVFRSSVLHSTQDLFLPFFS
MZ298840/B.1.351/Beta          MFVFLVLLPLVSSQCVNLTTRTQLPPSYTNSFTRGVYYPDKVFRSSVLHSTQDLFLPFFS
LC643036/B.1.617.2/Delta       MFVFLVLLPLVSSQCVNLRTRTQLPPAYTNSFTRGVYYPDKVFRSSVLHSTQDLFLPFFS
LC643044/B.1.617.2/Delta       MFVFLVLLPLVSSQCVNLRTRTQLPPAYTNSFTRGVYYPDKVFRSSVLHSTQDLFLPFFS
MZ853946/B.1.617.2/Delta       MFVFLVLLPLVSSQCVNLRTRTQLPPAYTNSFTRGVYYPDKVFRSSVLHSTQDLFLPFFS
MZ854386/B.1.617.2/Delta       MFVFLVLLPLVSSQCVNLRTRTQLPPAYTNSFTRGVYYPDKVFRSSVLHSTQDLFLPFFS
MZ888532/B.1.617.2/Delta       MFVFLVLLPLVSSQCVNLRTRTQLPPAYTNSFTRGVYYPDKVFRSSVLHSTQDLFLPFFS
MZ888533/B.1.617.2/Delta       MFVFLVLLPLVSSQCVNLRTRTQLPPAYTNSFTRGVYYPDKVFRSSVLHSTQDLFLPFFS
MZ896136/B.1.617.2/Delta       MFVFLVLLPLVSSQCVNLRTRTQLPPAYTNSFTRGVYYPDKVFRSSVLHSTQDLFLPFFS
OU539261/B.1.617.2/Delta       MFVFLVLLPLVSSQCVNLRTRTQLPPAYTNSFTRGVYYPDKVFRSSVLHSTQDLFLPFFS
MZ854389/B.1.617.2/Delta       MFVFLVLLPLVSSQCVNLRTRTQLPPAYTNSFTRGVYYPDKVFRSSVLHSTQDLFLPFFS
OU539784/P.1/Gamma             MFVFLVLLPLVSSQCVNFTNRTQLPSAYTNSFTRGVYYPDKVFRSSVLHSTQDLFLPFFS
OU539830/P.1/Gamma             MFVFLVLLPLVSSQCVNFTNRTQLPSAYTNSFTRGVYYPDKVFRSSVLHSTQDLFLPFFS
OU539894/P.1/Gamma             MFVFLVLLPLVSSQCVNFTNRTQLPSAYTNSFTRGVYYPDKVFRSSVLHSTQDLFLPFFS
MZ477748/P.1/Gamma             MFVFLVLLPLVSSQCVNFTNRTQLPSAYTNSFTRGVYYPDKVFRSSVLHSTQDLFLPFFS
MZ477746/P.1/Gamma             MFVFLVLLPLVSSQCVNFTNRTQLPSAYTNSFTRGVYYPDKVFRSSVLHSTQDLFLPFFS
MW938104/P.1/Gamma             MFVFLVLLPLVSSQCVNFTNRTQLPSAYTNSFTRGVYYPDKVFRSSVLHSTQDLFLPFFS
MZ896179/P.1/Gamma             MFVFLVLLPLVSSQCVNFTNRTQLPSAYTNSFTRGVYYPDKVFRSSVLHSTQDLFLPFFS
MZ896359/P.1/Gamma             MFVFLVLLPLVSSQCVNFTNRTQLPSAYTNSFTRGVYYPDKVFRSSVLHSTQDLFLPFFS
OL677199/BA.1/Omicron          MFVFLVLLPLVSSQCVNLTTRTQLPPAYTNSFTRGVYYPDKVFRSSVLHSTQDLFLPFFS
OL672836/BA.1/Omicron          MFVFLVLLPLVSSQCVNLTTRTQLPPAYTNSFTRGVYYPDKVFRSSVLHSTQDLFLPFFS
OW714547.1/BA.1/Omicron        MFVFLVLLPLVSSQCVNLTTRTQLPPAYTNSFTRGVYYPDKVFRSSVLHSTQDLFLPFFS
OW714198.1/BA.1/Omicron        MFVFLVLLPLVSSQCVNLTTRTQLPPAYTNSFTRGVYYPDKVFRSSVLHSTQDLFLPFFS
OW303180.1/BA.1/Omicron        MFVFLVLLPLVSSQCVNLTTRTQLPPAYTNSFTRGVYYPDKVFRSSVLHSTQDLFLPFFS
EPI_ISL_7747552/BA.2/Omicron   MFVFLVLLPLVSSQCVNLITRTQ....YTNSFTRGVYYPDKVFRSSVLHSTQDLFLPFFS
EPI_ISL_7747545/BA.2/Omicron   MFVFLVLLPLVSSQCVNLITRTQ....YTNSFTRGVYYPDKVFRSSVLHSTQDLFLPFFS
EPI_ISL_7747536/BA.2/Omicron   MFVFLVLLPLVSSQCVNLITRTQ....YTNSFTRGVYYPDKVFRSSVLHSTQDLFLPFFS
EPI_ISL_7747535/BA.2/Omicron   MFVFLVLLPLVSSQCVNLITRTQ....YTNSFTRGVYYPDKVFRSSVLHSTQDLFLPFFS
EPI_ISL_6795834/BA.2/Omicron   MFVFLVLLPLVSSQCVNLITRTQ....YTNSFTRGVYYPDKVFRSSVLHSTQDLFLPFFS
EPI_ISL_7718520/BA.2/Omicron   MFVFLVLLPLVSSQCVNLITRTQ....YTNSFTRGVYYPDKVFRSSVLHSTQDLFLPFFS
EPI_ISL_7580387/BA.2/Omicron   MFVFLVLLPLVSSQCVNLITRTQ....YTNSFTRGVYYPDKVFRSSVLHSTQDLFLPFFS
EPI_ISL_7701122/BA.2/Omicron   MFVFLVLLPLVSSQCVNLITRTQ....YTNSFTRGVYYPDKVFRSSVLHSTQDLFLPFFS
EPI_ISL_7190366/BA.2/Omicron   MFVFLVLLPLVSSQCVNLITRTQ....YTNSFTRGVYYPDKVFRSSVLHSTQDLFLPFFS
EPI_ISL_7644798/BA.2/Omicron   MFVFLVLLPLVSSQCVNLITRTQ....YTNSFTRGVYYPDKVFRSSVLHSTQDLFLPFFS
EPI_ISL_7740766/BA.3/Omicron   MFVFLVLLPLVSSQCVNLTTRTQLPPAYTNSFTRGVYYPDKVFRSSVLHSTQDLFLPFFS
EPI_ISL_7605589/BA.3/Omicron   MFVFLVLLPLVSSQCVNLTTRTQLPPAYTNSFTRGVYYPDKVFRSSVLHSTQDLFLPFFS
ON296657.1/BA.4/Omicron        MFGFLVLLPLVSSQCVNLITRTQ....YTNSFTRGVYYPDKVFRSSVLHSTQDLFLPFFS
ON331287.1/BA.4/Omicron        MFGFLVLLPLVSSQCVNLITRTQ....YTNSFTRGVYYPDKVFRSSVLHSTQDLFLPFFS
ON321245.1/BA.4/Omicron        MFGFLVLLPLVSSQCVNLITRTQ....YTNSFTRGVYYPDKVFRSSVLHSTQDLFLPFFS
ON337003.1/BA.4/Omicron        MFGFLVLLPLVSSQCVNLITRTQ....YTNSFTRGVYYPDKVFRSSVLHSTQDLFLPFFS
ON270681.1/BA.4/Omicron        MFGFLVLLPLVSSQCVNLITRTQ....YTNSFTRGVYYPDKVFRSSVLHSTQDLFLPFFS
ON373214.1/BA.4/Omicron        MFGFLVLLPLVSSQCVNLITRTQ....YTNSFTRGVYYPDKVFRSSVLHSTQDLFLPFFS
ON393428.1/BA.5/Omicron        MFVFLVLLPLVSSQCVNLITRTQ....YTNSFTRGVYYPDKVFRSSVLHSTQDLFLPFFS
ON393156.1/BA.5/Omicron        MFVFLVLLPLVSSQCVNLITRTQ....YTNSFTRGVYYPDKVFRSSVLHSTQDLFLPFFS
ON348053.1/BA.5/Omicron        MFVFLVLLPLVSSQCVNLITRTQ....YTNSFTRGVYYPDKVFRSSVLHSTQDLFLPFFS
ON323731.1/BA.5/Omicron        MFVFLVLLPLVSSQCVNLITRTQ....YTNSFTRGVYYPDKVFRSSVLHSTQDLFLPFFS
ON251467.1/BA.5/Omicron        MFVFLVLLPLVSSQCVNLITRTQ....YTNSFTRGVYYPDKVFRSSVLHSTQDLFLPFFS
ON250431.1/BA.5/Omicron        MFVFLVLLPLVSSQCVNLITRTQ....YTNSFTRGVYYPDKVFRSSVLHSTQDLFLPFFS
MZ911221/B.1.427/Epsilon       MFVFLVLLPLVSIQCVNLTTRTQLPPAYTNSFTRGVYYPDKVFRSSVLHSTQDLFLPFFS
MZ375858/B.1.427/Epsilon       MFVFLVLLPLVSIQCVNLTTRTQLPPAYTNSFTRGVYYPDKVFRSSVLHSTQDLFLPFFS
MZ375854/B.1.427/Epsilon       MFVFLVLLPLVSIQCVNLTTRTQLPPAYTNSFTRGVYYPDKVFRSSVLHSTQDLFLPFFS
MZ943834/B.1.429/Epsilon       MFVFLVLLPLVSIQCVNLTTRTQLPPAYTNSFTRGVYYPDKVFRSSVLHSTQDLFLPFFS
MZ277390/B.1.429/Epsilon       MFVFLVLLPLVSIQCVNLTTRTQLPSAYTNSFTRGVYYPDKVFRSSVLHSTQDLFLPFFS
OU282717/B.1.429/Epsilon       MFVFLVLLPLVSIQCVNLTTRTQLPPAYTNSFTRGVYYPDKVFRSSVLHSTQDLFLPFFS
OU235436/B.1.429/Epsilon       MFVFLVLLPLVSIQCVNLTTRTQLPPAYTNSFTRGVYYPDKVFRSSVLHSTQDLFLPFFS
MZ687447/B.1.429/Epsilon       MFVFLVLLPLVSIQCVNLTTRTQLPPAYTNSFTRGVYYPDKVFRSSVLHSTQDLFLPFFS
MZ363839/B.1.617.1/Kappa       MFVFLVLLPLVSSQCVNLTTRTQLPPAYTNSFTRGVYYPDKVFRSSVLHSTQDLFLPFFS
MZ538882/B.1.617.1/Kappa       MFVFLVLLPLVSSQCVNLTTRTQLPPAYTNSFTRGVYYPDKVFRSSVLHSTQDLFLPFFS
MZ724425/B.1.617.1/Kappa       MFVFLVLLPLVSSQCVNLTTRTQLPPAYTNSFTRGVYYPDKVFRSSVLHSTQDLFLPFFS
MZ332524/B.1.617.1/Kappa       MFVFLVLLPLVSSQCVNLTTRTQLPPAYTNSFTRGVYYPDKVFRSSVLHSTQDLFLPFFS
MZ332527/B.1.617.1/Kappa       MFVFLVLLPLVSSQCVNLTTRTQLPPAYTNSFTRGVYYPDKVFRSSVLHSTQDLFLPFFS
LC633761/B.1.617.1/Kappa       MFVFLVLLPLVSSQCVNLTTRTQLPPAYTNSFTRGVYYPDKVFRSSVLHSTQDLFLPFFS
MZ571142/B.1.617.1/Kappa       MFVFLVLLPLVSSQCVNLTTRTQLPPAYTNSFTRGVYYPDKVFRSSVLHSTQDLFLPFFS
MZ562746/B.1.617.1/Kappa       MFVFLVLLPLVSSQCVNLTTRTQLPPAYTNSFTRGVYYPDKVFRSSVLHSTQDLFLPFFS
OU053018/C.37/Lambda           MFVFLVLLPLVSSQCVNLTTRTQLPPAYTNSFTRGVYYPDKVFRSSVLHSTQDLFLPFFS
OU268528/C.37/Lambda           MFVFLVLLPLVSSQCVNLTTRTQLPPAYTNSFTRGVYYPDKVFRSSVLHSTQDLFLPFFS
OU100999/C.37/Lambda           MFVFLVLLPLVSSQCVNLTTRTQLPPAYTNSFTRGVYYPDKVFRSSVLHSTQDLFLPFFS
MZ275295/C.37/Lambda           MFVFLVLLPLVSSQCVNLTTRTQLPPAYTNSFTRGVYYPDKVFRSSVLHSTQDLFLPFFS
MZ275301/C.37/Lambda           MFVFLVLLPLVSSQCVNLTTRTQLPPAYTNSFTRGVYYPDKVFRSSVLHSTQDLFLPFFS
MZ908711/C.37/Lambda           MFVFLVLLPLVSSQCVNLTTRTQLPPAYTNSFTRGVYYPDKVFRSSVLHSTQDLFLPFFS
OU517038/C.37/Lambda           MFVFLVLLPLVSSQCVNLTTRTQLPPAYTNSFTRGVYYPDKVFRSSVLHSTQDLFLPFFS
MZ496613/C.37/Lambda           MFVFLVLLPLVSSQCVNLTTRTQLPPAYTNSFTRGVYYPDKVFRSSVLHSTQDLFLPFFS
OU466149/B.1.621/Mu            MFVFLVLLPLVSSQCVNLTTRTQLPPAYTNSFTRGVYYPDKVFRSSVLHSTQDLFLPFFS
OU571573/B.1.621/Mu            MFVFLVLLPLVSSQCVNLTTRTQLPPAYTNSFTRGVYYPDKVFRSSVLHSTQDLFLPFFS
OK025270/B.1.621/Mu            MFVFLVLLPLVSSQCVNLTTRTQLPPAYTNSFTRGVYYPDKVFRSSVLHSTQDLFLPFFS
```

```
                                            70        80        90       100       110       120
MN908947/Wuhan-Hu-1                 NVTWFHAIHVSGTNGTKRFDNPVLPFNDGVYFASTEKSNIIRGWIFGTTLDSKTQSLLIV
BS001137/B.1.1.7/Alpha              NVTWFHAI..SGTNGTKRFDNPVLPFNDGVYFASTEKSNIIRGWIFGTTLDSKTQSLLIV
BS001138/B.1.1.7/Alpha              NVTWFHAI..SGTNGTKRFDNPVLPFNDGVYFASTEKSNIIRGWIFGTTLDSKTQSLLIV
BS001139/B.1.1.7/Alpha              NVTWFHAI..SGTNGTKRFDNPVLPFNDGVYFASTEKSNIIRGWIFGTTLDSKTQSLLIV
BS001140/B.1.1.7/Alpha              NVTWFHAI..SGTNGTKRFDNPVLPFNDGVYFASTEKSNIIRGWIFGTTLDSKTQSLLIV
MW913362/B.1.1.7/Alpha              NVTWFHAI..SGTNGTKRFDNPVLPFNDGVYFASTEKSNIIRGWIFGTTLDSKTQSLLIV
MZ266365/B.1.1.7/Alpha              NVTWFHAI..SGTNGTKRFDNPVLPFNDGVYFASTEKSNIIRGWIFGTTLDSKTQSLLIV
MZ350109/B.1.1.7/Alpha              NVTWFHAI..SGTNGTKRFDNPVLPFNDGVYFASTEKSNIIRGWIFGTTLDSKTQSLLIV
MZ888516/B.1.1.7/Alpha              NVTWFHAI..SGTNGTKRFDNPVLPFNDGVYFASTEKSNIIRGWIFGTTLDSKTQSLLIV
OU562607/B.1.1.7/Alpha              NVTWFHAI..SGTNGTKRFDNPVLPFNDGVYFASTEKSNIIRGWIFGTTLDSKTQSLLIV
OU514166/B.1.1.7/Alpha              NVTWFHAI..SGTNGTKRFDNPVLPFNDGVYFASTEKSNIIRGWIFGTTLDSKTQSLLIV
OU538849/B.1.1.7/Alpha              NVTWFHAI..SGTNGTKRFDNPVLPFNDGVYFAFTEKSNIIRGWIFGTTLDSKTQSLLIV
MW981442/B.1.351/Beta               NVTWFHAIHVSGTNGTKRFANPVLPFNDGVYFASTEKSNIIRGWIFGTTLDSKTQSLLIV
MZ298839/B.1.351/Beta               NVTWFHAIHVSGTNGTKRFANPVLPFNDGVYFASTEKSNIIRGWIFGTTLDSKTQSLLIV
MZ913100/B.1.351/Beta               NVTWFHAIHVSGTNGTKRFANPVLPFNDGVYFASTEKSNIIRGWIFGTTLDSKTQSLLIV
MZ879365/B.1.351/Beta               NVTWFHAIHVSGTNGTKRFANPVLPFNDGVYFASTEKSNIIRGWIFGTTLDSKTQSLLIV
OU539352/B.1.351/Beta               NVTWFHAIHVSGTNGTKRFANPVLPFNDGVYFASTEKSNIIRGWIFGTTLDSKTQSLLIV
MZ202314/B.1.351/Beta               NVTWFHAIHVSGTNGTKRFANPVLPFNDGVYFASTEKSNIIRGWIFGTTLDSKTQSLLIV
MZ298840/B.1.351/Beta               NVTWFHAIHVSGTNGTKRFANPVLPFNDGVYFASTEKSNIIRGWIFGTTLDSKTQSLLIV
LC643036/B.1.617.2/Delta            NVTWFHAIHVSGTNGTKRFDNPVLPFNDGVYFASIEKSNIIRGWIFGTTLDSKTQSLLIV
LC643044/B.1.617.2/Delta            NVTWFHAIHVSGTNGTKRFDNPVLPFNDGVYFASIEKSNIIRGWIFGTTLDSKTQSLLIV
MZ853946/B.1.617.2/Delta            NVTWFHAIHVSGTNGTKRFDNPVLPFNDGVYFASTEKSNIIRGWIFGTTLDSKTQSLLIV
MZ854386/B.1.617.2/Delta            NVTWFHAIHVSGTNGTKRFDNPVLPFNDGVYFASTEKSNIIRGWIFGTTLDSKTQSLLIV
MZ888532/B.1.617.2/Delta            NVTWFHAIHVSGTNGTKRFDNPVLPFNDGVYFASTEKSNIIRGWIFGTTLDSKTQSLLIV
MZ888533/B.1.617.2/Delta            NVTWFHAIHVSGTNGTKRFDNPVLPFNDGVYFASTEKSNIIRGWIFGTTLDSKTQSLLIV
MZ896136/B.1.617.2/Delta            NVTWFHAIHVSGTNGTKRFDNPVLPFNDGVYFASTEKSNIIRGWIFGTTLDSKTQSLLIV
OU539261/B.1.617.2/Delta            NVTWFHAIHVSGTNGTTRFDNPVLPFNDGVYFASTEKSNIIRGWIFGTTLDSKTQSLLIV
MZ854389/B.1.617.2/Delta            NVTWFHAIHVSGTNGTKRFDNPVLPFNDGVYFASTEKSNIIRGWIFGTTLDSKTQSLLIV
OU539784/P.1/Gamma                  NVTWFHAIHVSGTNVTKRFDNPVLPFNDGVYFASTEKSNIIRGWIFGTTLDSKTQSLLIV
OU539830/P.1/Gamma                  NVTWFHAIHVSGTNGTKRFDNPVLPFNDGVYFASTEKSNIIRGWIFGTTLDSKTQSLLIV
OU539894/P.1/Gamma                  NVTWFHAIHVSGTNGTKRFDNPVLPFNDGVYFASTEKSNIIRGWIFGTTLDSKTQSLLIV
MZ477748/P.1/Gamma                  NVTWFHAIHVSGTNGTKRFDNPVLPFNDGVYFASTEKSNIIRGWIFGTTLDSKTQSLLIV
MZ477746/P.1/Gamma                  NVTWFHAIHVSGTNGTKRFDNPVLPFNDGVYFASTEKSNIIRGWIFGTTLDSKTQSLLIV
MW938104/P.1/Gamma                  NVTWFHAIHVSGTNGTKRFDNPVLPFNDGVYFASTEKSNIIRGWIFGTTLDSKTQSLLIV
MZ896179/P.1/Gamma                  NVTWFHAIHVSGTNGTKRFDNPVLPFNDGVYFASTEKSNIIRGWIFGTTLDSKTQSLLIV
MZ896359/P.1/Gamma                  NVTWFHAIHVSGTNGTKRFDNPVLPFNDGVYFASTEKSNIIRGWIFGTTLDSKTQSLLIV
OL677199/BA.1/Omicron               NVTWFHV...SGTNGTKRFDNPVLPFNDGVYFASIEKSNIIRGWIFGTTLDSKTQSLLIV
OL672836/BA.1/Omicron               NVTWFHV...SGTNGTKRFDNPVLPFNDGVYFASIEKSNIIRGWIFGTTLDSKTQSLLIV
OW714547.1/BA.1/Omicron             NVTWFHV..ISGTNGTKRFDNPVLPFNDGVYFASIEKSNIIRGWIFGTTLDSKTQSLLIV
OW714198.1/BA.1/Omicron             NVTWFHV..ISGTNGTKRFDNPVLPFNDGVYFASIEKSNIIRGWIFGTTLDSKTQSLLIV
OW303180.1/BA.1/Omicron             NVTWFHV..ISGTNGTKRFDNPVLPFNDGVYFASIEKSNIIRGWIFGTTLDSKTQSLLIV
EPI_ISL_7747552/BA.2/Omicron        NVTWFHAIHVSGTNGTKRFDNPVLPFNDGVYFASTEKSNIIRGWIFGTTLDSKTQSLLIV
EPI_ISL_7747545/BA.2/Omicron        NVTWFHAIHVSGTNGTKRFDNPVLPFNDGVYFASTEKSNIIRGWIFGTTLDSKTQSLLIV
EPI_ISL_7747536/BA.2/Omicron        NVTWFHAIHVSGTNGTKRFDNPVLPFNDGVYFASTEKSNIIRGWIFGTTLDSKTQSLLIV
EPI_ISL_7747535/BA.2/Omicron        NVTWFHAIHVSGTNGTKRFDNPVLPFNDGVYFASTEKSNIIRGWIFGTTLDSKTQSLLIV
EPI_ISL_6795834/BA.2/Omicron        NVTWFHAIHVSGTNGTKRFDNPVLPFNDGVYFASTEKSNIIRGWIFGTTLDSKTQSLLIV
EPI_ISL_7718520/BA.2/Omicron        NVTWFHAIHVSGTNGTKRFDNPVLPFNDGVYFASTEKSNIIRGWIFGTTLDSKTQSLLIV
EPI_ISL_7580387/BA.2/Omicron        NVTWFHAIHVSGTNGTKRFDNPVLPFNDGVYFASTEKSNIIRGWIFGTTLDSKTQSLLIV
EPI_ISL_7701122/BA.2/Omicron        NVTWFHAIHVSGTNGTKRFDNPVLPFNDGVYFASTEKSNIIRGWIFGTTLDSKTQSLLIV
EPI_ISL_7190366/BA.2/Omicron        NVTWFHAIHVSGTNGTKRFDNPVLPFNDGVYFASTEKSNIIRGWIFGTTLDSKTQSLLIV
EPI_ISL_7644798/BA.2/Omicron        NVTWFHAIHVSGTNGTKRFDNPVLPFNDGVYFASTEKSNIIRGWIFGTTLDSKTQSLLIV
EPI_ISL_7740766/BA.3/Omicron        NVTWFHV..ISGTNGTKRFDNPVLPFNDGVYFASIEKSNIIRGWIFGTTLDSKTQSLLIV
EPI_ISL_7605589/BA.3/Omicron        NVTWFHV..ISGTNGTKRFDNPVLPFNDGVYFASIEKSNIIRGWIFGTTLDSKTQSLLIV
ON296657.1/BA.4/Omicron             NVTWFHA...SGTNGTKRFDNPVLPFNDGVYFASTEKSNIIRGWIFGTTLDSKTQSLLIV
ON331287.1/BA.4/Omicron             NVTWFHA...SGTNGTKRFDNPVLPFNDGVYFASTEKSNIIRGWIFGTTLDSKTQSLLIV
ON321245.1/BA.4/Omicron             NVTWFHA...SGTNGTKRFDNPVLPFNDGVYFASTEKSNIIRGWIFGTTLDSKTQSLLIV
ON337003.1/BA.4/Omicron             NVTWFHA...SGTNGTKRFDNPVLPFNDGVYFASTEKSNIIRGWIFGTTLDSKTQSLLIV
ON270681.1/BA.4/Omicron             NVTWFHA...SGTNGTKRFDNPVLPFNDGVYFASTEKSNIIRGWIFGTTLDSKTQSLLIV
ON373214.1/BA.4/Omicron             NVTWFHA...SGTNGTKRFDNPVLPFNDGVYFASTEKSNIIRGWIFGTTLDSKTQSLLIV
ON393428.1/BA.5/Omicron             NVTWFHA...SGTNGTKRFDNPVLPFNDGVYFASTEKSNIIRGWIFGTTLDSKTQSLLIV
ON393156.1/BA.5/Omicron             NVTWFHA...SGTNGTKRFDNPVLPFNDGVYFASTEKSNIIRGWIFGTTLDSKTQSLLIV
ON348053.1/BA.5/Omicron             NVTWFHA...SGTNGTKRFDNPVLPFNDGVYFASTEKSNIIRGWIFGTTLDSKTQSLLIV
ON323731.1/BA.5/Omicron             NVTWFHA...SGTNGIKRFDNPVLPFNDGVYFASTEKSNIIRGWIFGTTLDSKTQSLLIV
ON251467.1/BA.5/Omicron             NVTWFHA...SGTNGIKRFDNPVLPFNDGVYFASTEKSNIIRGWIFGTTLDSKTQSLLIV
ON250431.1/BA.5/Omicron             NVTWFHA...SGTNGIKRFDNPVLPFNDGVYFASTEKSNIIRGWIFGTTLDSKTQSLLIV
MZ911221/B.1.427/Epsilon            NVTWFHAIHVSGTNGTKRFDNPVLPFNDGVYFASTEKSNIIRGWIFGTTLDSKTQSLLIV
MZ375858/B.1.427/Epsilon            NVTWFHAIHVSGTNGTKRFDNPVLPFNDGVYFASTEKSNIIRGWIFGTTLDSKTQSLLIV
MZ375854/B.1.427/Epsilon            NVTWFHAIHVSGTNGTKRFDNPVLPFNDGVYFASTEKSNIIRGWIFGTTLDSKTQSLLIV
MZ943834/B.1.429/Epsilon            NVTWFHAIHVSGTNGTKRFDNPVLPFNDGVYFASTEKSNIIRGWIFGTTLDSKTQSLLIV
MZ277390/B.1.429/Epsilon            NVTWFHAIHVSGTNGTKRFDNPVLPFNDGVYFASTEKSNIIRGWIFGTTLDSKTQSLLIV
OU282717/B.1.429/Epsilon            NVTWFHAIHVSGTNGTKRFDNPVLPFNDGVYFASTEKSNIIRGWIFGTTLDSKTQSLLIV
OU235436/B.1.429/Epsilon            NVTWFHAIHVSGTNGTKRFDNPVLPFNDGVYFASTEKSNIIRGWIFGTTLDSKTQSLLIV
MZ687447/B.1.429/Epsilon            NVTWFHAIHVSGTNGTKRFDNPVLPFNDGVYFASTEKSNIIRGWIFGTTLDSKTQSLLIV
MZ363839/B.1.617.1/Kappa            NVTWFHAIHVSGTNGTKRFDNPVLPFNDGVYFASIEKSNIIRGWIFGTTLDSKTQSLLIV
MZ538882/B.1.617.1/Kappa            NVTWFHAIHVSGTNGTKRFDNPVLPFNDGVYFASIEKSNIIRGWIFGTTLDSKTQSLLIV
MZ724425/B.1.617.1/Kappa            NVTWFHAIHVSGTNGTKRFDNPVLPFNDGVYFASIEKSNIIRGWIFGTTLDSKTQSLLIV
MZ332524/B.1.617.1/Kappa            NVTWFHAIHVSGTNGTKRFDNPVLPFNDGVYFASIEKSNIIRGWIFGTTLDSKTQSLLIV
MZ332527/B.1.617.1/Kappa            NVTWFHAIHVSGTNGTKRFDNPVLPFNDGVYFASTEKSNIIRGWIFGTTLDSKTQSLLIV
LC633761/B.1.617.1/Kappa            NVTWFHAIHVSGTNGTKRFDNPVLPFNDGVYFASIEKSNIIRGWIFGTTLDSKTQSLLIV
MZ571142/B.1.617.1/Kappa            NVTWFHAIHVSGTNGTKRFDNPVLPFNDGVYFASIEKSNIIRGWIFGTTLDSKTQSLLIV
MZ562746/B.1.617.1/Kappa            NVTWFHAIHVSGTNGTKRFDNPVLPFNDGVYFASIEKSNIIRGWIFGTTLDSKTQSLLIV
OU053018/C.37/Lambda                NVTWFHAIHVSGTNVIKRFDNPVLPFNDGVYFASTEKSNIIRGWIFGTTLDSKTQSLLIV
OU268528/C.37/Lambda                NVTWFHAIHVSGTNVIKRFDNPVLPFNDGVYFASTEKSNIIRGWIFGTTLDSKTQSLLIV
OU100999/C.37/Lambda                NVTWFHAIHVSGTNVIKRFDNPVLPFNDGVYFASTEKSNIIRGWIFGTTLDSKTQSLLIV
MZ275295/C.37/Lambda                NVTWFHAIHVSGTNVIKRFDNPVLPFNDGVYFASTEKSNIIRGWIFGTTLDSKTQSLLIV
MZ275301/C.37/Lambda                NVTWFHAIHVSGTNVIKRFDNPVLPFNDGVYFASTEKSNIIRGWIFGTTLDSKTQSLLIV
MZ908711/C.37/Lambda                NVTWFHAIHVSGTNVIKRFDNPVLPFNDGVYFASTEKSNIIRGWIFGTTLDSKTQSLLIV
OU517038/C.37/Lambda                NVTWFHAIHVSGTNVIKRFDNPVLPFNDGVYFASTEKSNIIRGWIFGTTLDSKTQSLLIV
MZ496613/C.37/Lambda                NVTWFHAIHVSGTNVIKRFDNPVLPFNDGVYFASTEKSNIIRGWIFGTTLDSKTQSLLIV
OU466149/B.1.621/Mu                 NVTWFHAIHVSGTNGTKRFDNPVLPFNDGVYFASIEKSNIIRGWIFGTTLDSKTQSLLIV
OU571573/B.1.621/Mu                 NVTWFHAIHVSGTNGTKRFDNPVLPFNDGVYFASIEKSNIIRGWIFGTTLDSKTQSLLIV
OK025270/B.1.621/Mu                 NVTWFHAIHVSGTNGTKRFDNPVLPFNDGVYFASIEKSNIIRGWIFGTTLDSKTQSLLIV
```

```
                                       130       140       150       160       170
                                         .         .         .         .         .
MN908947/Wuhan-Hu-1                 NNATNVVIKVCEFQFCNDPFLGVY.YHKNNKSWMESEFRVYSSANNCTFEYVSQPFLMDL
BS001137/B.1.1.7/Alpha              NNATNVVIKVCEFQFCNDPFLG...YHKNNKSWMESEFRVYSSANNCTFEYVSQPFLMDL
BS001138/B.1.1.7/Alpha              NNATNVVIKVCEFQFCNDPFLG...YHKNNKSWMESEFRVYSSANNCTFEYVSQPFLMDL
BS001139/B.1.1.7/Alpha              NNATNVVIKVCEFQFCNDPFLG...YHKNNKSWMESEFRVYSSANNCTFEYVSQPFLMDL
BS001140/B.1.1.7/Alpha              NNATNVVIKVCEFQFCNDPFLG...YYKNNKSWMESEFRVYSSANNCTFEYVSQPFLMDL
MW913362/B.1.1.7/Alpha              NNATNVVIKVCEFQFCNDPFLG...YHKNNKSWMESEFRVYSSANNCTFEYVSQPFLMDL
MZ266365/B.1.1.7/Alpha              NNATNVVIKVCEFQFCNDPFLG...YHKNNKSWMESEFRVYSSANNCTFEYVSQPFLMDL
MZ350109/B.1.1.7/Alpha              NNATNVVIKVCEFQFCNDPFLG...YHKNNKSWMESEFRVYSSANNCTFEYVSQPFLMDL
MZ888516/B.1.1.7/Alpha              NNATNVVIKVCEFQFCNDPFLG...YHKNNKSWMESEFRVYSSANNCTFEYVSQPFLMDL
OU562607/B.1.1.7/Alpha              NNATNVVIKVCEFQFCNDPFLG...YHKNNKSWMESEFRVYSSANNCTFEYVSQPFFMDL
OU514166/B.1.1.7/Alpha              NNATNVVIKVCEFQFCNDPFLG...YHKNNKSWMESEFRVYSSANNCTFEYVSQPFLMDL
OU538849/B.1.1.7/Alpha              NNATNVVIKVCEFQFCNDPFLG...YHKNNKSWMESEFRVYSSANNCTFEYVSQPFLMDL
MW981442/B.1.351/Beta               NNATNVVIKVCEFQFCNDPFLGVY.YHKNNKSWMESEFRVYSSANNCTFEYVSQPFLMDL
MZ298839/B.1.351/Beta               NNATNVVIKVCEFQFCNDPFLGVY.YHKNNKSWMESEFRVYSSANNCTFEYVSQPFLMDL
MZ913100/B.1.351/Beta               NNATNVVIKVCEFQFCNYPFLGVY.YHKNNKSWMESEFRVYSSANNCTFEYVSQPFLMDL
MZ879365/B.1.351/Beta               NNATNVVIKVCEFQFCNDPFLGVY.YHKNNKSWMESEFRVYSSANNCTFEYVSQPFLMDL
OU539352/B.1.351/Beta               NNATNVVIKVCEFQFCNDPFLGVY.YHKNNKSWMESEFRVYSSANNCTFEYVSQPFLMDL
MZ202314/B.1.351/Beta               NNATNVVIKVCEFQFCNDPFLGVY.YHKNNKSWMESEFRVYSSANNCTFEYVSQPFLMDL
MZ298840/B.1.351/Beta               NNATNVVIKVCEFQFCNDPFLGVY.YHKNNKSWMESEFRVYSSANNCTFEYVSQPFLMDL
LC643036/B.1.617.2/Delta            NNATNVVIKVCEFQFCNDPFLDVY.YHKNNKSWMES...VYSSANNCTFEYVSQPFLMDL
LC643044/B.1.617.2/Delta            NNATNVVIKVCEFQFCNDPFLDVY.YHKNNKSWMES...VYSSANNCTFEYVSQPFLMDL
MZ853946/B.1.617.2/Delta            NNATNVVIKVCEFQFCNDPFLDVY.YHKNNKSWMES...VYSSANNCTFEYVSQPFLMDL
MZ854386/B.1.617.2/Delta            NNATNVVIKVCEFQFCNDPFLDVY.YHKNNKSWMESEG.VYSSANNCTFEYVSQPFLMDL
MZ888532/B.1.617.2/Delta            NNATNVVIKVCEFQFCNDPFLGVY.YHKNNKSWMES...VYSSANNCTFEYVSQPFLMDL
MZ888533/B.1.617.2/Delta            NNATNVVIKVCEFQFCNDPFLDVY.YHKNNKSWMES...VYSSANNCTFEYVSQPFLMDL
MZ896136/B.1.617.2/Delta            NNATNVVIKVCEFQFCNDPFLDVY.YHKNNKSWMES...VYSSANNCTFEYVSQPFLMDL
OU539261/B.1.617.2/Delta            NNATNVVIKVCEFQFCNDPFLGVY.YHKNNKSWMES...VYSSANNCTFEYVSQPFLMDL
MZ854389/B.1.617.2/Delta            NNATNVVIKVCEFQFCNDPFLDVY.YHKNNKSWMES...VYSSANNCTFEYVSQPFLMDL
OU539784/P.1/Gamma                  NNATNVVIKVCEFQFCNYPFLGVY.YHKNNKSWMESEFRVYSSANNCTFEYVSQPFLMDL
OU539830/P.1/Gamma                  NNATNVVIKVCEFQFCNYPFLGVY.YHKNNKSWMESEFRVYSSANNCTFEYVSQPFLMDL
OU539894/P.1/Gamma                  NNATNVVIKVCEFQFCNYPFLGVY.YHKNNKSWMESEFRVYSSANNCTFEYVSQPFLMDL
MZ477748/P.1/Gamma                  NNATNVVIKVCEFQFCNYPFLGVY.YHKNNKSWMESEFRVYSSANNCTFEYVSQPFLMDL
MZ477746/P.1/Gamma                  NNATNVVIKVCEFQFCNYPFLGVY.YHKNNKSWMESEFRVYSSANNCTFEYVSQPFLMDL
MW938104/P.1/Gamma                  NNATNVVIKVCEFQFCNYPFLGVY.YHKNNKSWMESEFRVYSSANNCTFEYVSQPFLMDL
MZ896179/P.1/Gamma                  NNATNVVIKVCEFQFCNYPFLGVY.YHKNNKSWMESEFRVYSSANNCTFEYVSQPFLMDL
MZ896359/P.1/Gamma                  NNATNVVIKVCEFQFCNYPFLGVY.YHKNNKSWMESEFRVYSSANNCTFEYVSQPFLMDL
OL677199/BA.1/Omicron               NNATNVVIKVCEFQFCNDPFL....HKNNKSWMESEFRVYSSANNCTFEYVSQPFLMDL
OL672836/BA.1/Omicron               NNATNVVIKVCEFQFCNDPFL....HKNNKSWMESEFRVYSSANNCTFEYVSQPFLMDL
OW714547.1/BA.1/Omicron             NNATNVVIKVCEFQFCNDPFLD...HKNNKSWMESEFRVYSSANNCTFEYVSQPFLMDL
OW714198.1/BA.1/Omicron             NNATNVVIKVCEFQFCNDPFLD...HKNNKSWMESEFRVYSSANNCTFEYVSQPFLMDL
OW303180.1/BA.1/Omicron             NNATNVVIKVCEFQFCNDPFLD...HKNNKSWMESEFRVYSSANNCTFEYVSQPFLMDL
EPI_ISL_7747552/BA.2/Omicron        NNATNVVIKVCEFQFCNDPFLDVY..HKNNKSWMESEFRVYSSANNCTFEYVSQPFLMDL
EPI_ISL_7747545/BA.2/Omicron        NNATNVVIKVCEFQFCNDPFLDVY..HKNNKSWMESEFRVYSSANNCTFEYVSQPFLMDL
EPI_ISL_7747536/BA.2/Omicron        NNATNVVIKVCEFQFCNDPFLDVY..HKNNKSWMESEFRVYSSANNCTFEYVSQPFLMDL
EPI_ISL_7747535/BA.2/Omicron        NNATNVVIKVCEFQFCNDPFLDVY..HKNNKSWMESEFRVYSSANNCTFEYVSQPFLMDL
EPI_ISL_6795834/BA.2/Omicron        NNATNVVIKVCEFQFCNDPFLDVY..HKNNKSWMESEFRVYSSANNCTFEYVSQPFLMDL
EPI_ISL_7718520/BA.2/Omicron        NNATNVVIKVCEFQFCNDPFLDVY..HKNNKSWMESEFRVYSSANNCTFEYVSQPFLMDL
EPI_ISL_7580387/BA.2/Omicron        NNATNVVIKVCEFQFCNDPFLDVY..HKNNKSWMESEFRVYSSANNCTFEYVSQPFLMDL
EPI_ISL_7701122/BA.2/Omicron        NNATNVVIKVCEFQFCNDPFLDVY..HKNNKSWMESEFRVYSSANNCTFEYVSQPFLMDL
EPI_ISL_7190366/BA.2/Omicron        NNATNVVIKVCEFQFCNDPFLDVY..HKNNKSWMESEFRVYSSANNCTFEYVSQPFLMDL
EPI_ISL_7644798/BA.2/Omicron        NNATNVVIKVCEFQFCNDPFLDVY..HKNNKSWMESEFRVYSSANNCTFEYVSQPFLMDL
EPI_ISL_7740766/BA.3/Omicron        NNATNVVIKVCEFQFCNDPFLD...HKNNKSWMESEFRVYSSANNCTFEYVSQPFLMDL
EPI_ISL_7605589/BA.3/Omicron        NNATNVVIKVCEFQFCNDPFLD...HKNNKSWMESEFRVYSSANNCTFEYVSQPFLMDL
ON296657.1/BA.4/Omicron             NNATNVVIKVCEFQFCNDPFLDVY..HKNNKSWMESEFRVYSSANNCTFEYVSQPFLMDL
ON331287.1/BA.4/Omicron             NNATNVVIKVCEFQFCNDPFLDVY..HKNNKSWMESEFRVYSSANNCTFEYVSQPFLMDL
ON321245.1/BA.4/Omicron             NNATNVVIKVCEFQFCNDPFLDVY..HKNNKSWMESEFRVYSSANNCTFEYVSQPFLMDL
ON337003.1/BA.4/Omicron             NNATNVVIKVCEFQFCNDPFLDVY..HKNNKSWMESEFRVYSSANNCTFEYVSQPFLMDL
ON270681.1/BA.4/Omicron             NNATNVVIKVCEFQFCNDPFLDVY..HKNNKSWMESEFRVYSSANNCTFEYVSQPFLMDL
ON373214.1/BA.4/Omicron             NNATNVVIKVCEFQFCNDPFLDVY..HKNNKSWMESEFRVYSSANNCTFEYVSQPFLMDL
ON393428.1/BA.5/Omicron             NNATNVVIKVCEFQFCNDPFLDVY..HKNNKSWMESEFRVYSSANNCTFEYVSQPFLMDL
ON393156.1/BA.5/Omicron             NNATNVVIKVCEFQFCNDPFLDVY..HKNNKSWMESEFRVYSSANNCTFEYVSQPFLMDL
ON348053.1/BA.5/Omicron             NNATNVVIKVCEFQFCNDPFLDVY..HKNNKSWMESEFRVYSSANNCTFEYVSQPFLMDL
ON323731.1/BA.5/Omicron             NNATNVVIKVCEFQFCNDPFLDVY..HKNNKSWMESEFRVYSSANNCTFEYVSQPFLMDL
ON251467.1/BA.5/Omicron             NNATNVVIKVCEFQFCNDPFLDVY..HKNNKSWMESEFRVYSSANNCTFEYVSQPFLMDL
ON250431.1/BA.5/Omicron             NNATNVVIKVCEFQFCNDPFLDVY..HKNNKSWMESEFRVYSSANNCTFEYVSQPFLMDL
MZ911221/B.1.427/Epsilon            NNATNVVIKVCEFQFCNDPFLGVY.YHKNNKSCMESEFRVYSSANNCTFEYVSQPFLMDL
MZ375858/B.1.427/Epsilon            NNATNVVIKVCEFQFCNDPFLGVY.YHKNNKSCMESEFRVYSSANNCTFEYVSQPFLMDL
MZ375854/B.1.427/Epsilon            NNATNVVIKVCEFQFCNDPFLGVY.YHKNNKSCMESEFRVYSSANNCTFEYVSQPFLMDL
MZ943834/B.1.429/Epsilon            NNATNVVIKVCEFQFCNDPFLGVY.YHKNNKSCMESEFRVYSSANNCTFEYVSQPFLMDL
MZ277390/B.1.429/Epsilon            NNATNVVIKVCEFQFCNDPFLGVY.YHKNNKSCMESEFRVYSSANNCTFEYVSQPFLMDL
OU282717/B.1.429/Epsilon            NNATNVVIKVCEFQFCNDPFLGVY.YHKNNKSCMESEFRVYSSANNCTFEYVSQPFLMDL
OU235436/B.1.429/Epsilon            NNATNVVIKVCEFQFCNDPFLGVY.YHKNNKSCMESEFRVYSSANNCTFEYVSQPFLMDL
MZ687447/B.1.429/Epsilon            NNATNVVIKVCEFQFCNDPFLGVY.YHKNNKSCMESEFRVYSSANNCTFEYVSQPFLMDL
MZ363839/B.1.617.1/Kappa            NNATNVVIKVCEFQFCNDPFLDVY.YHKNNKSWMESEFRVYSSANNCTFEYVSQPFLMDL
MZ538882/B.1.617.1/Kappa            NNATNVVIKVCEFQFCNDPFLDVY.YHKNNKSWMKSEFRVYSSANNCTFEYVSQPFLMDL
MZ724425/B.1.617.1/Kappa            NNATNVVIKVCEFQFCNDPFLDVY.YHKNNKSWMKSEFRVYSSANNCTFEYVSQPFLMDL
MZ332524/B.1.617.1/Kappa            NNATNVVIKVCEFQFCNDPFLDVY.YHKNNKSWMESEFRVYSSANNCTFEYVSQPFLMDL
MZ332527/B.1.617.1/Kappa            NNATNVVIKVCEFQFCNDPFLGVY.YHKNNKSWMESEFRVYSSANNCTFEYVSQPFLMDL
LC633761/B.1.617.1/Kappa            NNATNVVIKVCEFQFCNDPFLDVY.YHKNNKSWMKSEFRVYSSANNCTFEYVSQPFLMDL
MZ571142/B.1.617.1/Kappa            NNATNVVIKVCEFQFCNDPFLDVY.YHKNNKSWMKSEFRVYSSANNCTFEYVSQPFLMDL
MZ562746/B.1.617.1/Kappa            NNATNVVIKVCEFQFCNDPFLDVY.YHKNNKSWMKSEFRVYSSANNCTFEYVSQPFLMDL
OU053018/C.37/Lambda                NNATNVVIKVCEFQFCNDPFLGVY.YHKNNKSWMESEFRVYSSANNCTFEYVSQPFLMDL
OU268528/C.37/Lambda                NNATNVVIKVCEFQFCNDPFLGVY.YHKNNKSWMESEFRVYSSANNCTFEYVSQPFLMDL
OU100999/C.37/Lambda                NNATNVVIKVCEFQFCNDPFLGVY.YHKNNKSWMESEFRVYSSANNCTFEYVSQPFLMDL
MZ275295/C.37/Lambda                NNATNVVIKVCEFQFCNDPFLGVY.YHKNNKSWMESEFRVYSSANNCTFEYVSQPFLMDL
MZ275301/C.37/Lambda                NNATNVVIKVCEFQFCNDPFLGVY.YHKNNKSWMESEFRVYSSANNCTFEYVSQPFLMDL
MZ908711/C.37/Lambda                NNATNVVIKVCEFQFCNDPFLGVY.YHKNNKSWMESEFRVYSSANNCTFEYVSQPFLMDL
OU517038/C.37/Lambda                NNATNVVIKVCEFQFCNDPFLGVY.YHKNNKSWMESELRVYSSANNCTFEYVSQPFLMDL
MZ496613/C.37/Lambda                NNATNVVIKVCEFQFCNDPFLGVY.YHKNNKSWMESEFRVYSSANNCTFEYVSQPFLMDL
OU466149/B.1.621/Mu                 NNATNVVIKVCEFQFCNDPFLGVTSNHKNNKSWMESEFRVYSSANNCTFEYVSQPFLMDL
OU571573/B.1.621/Mu                 NNATNVVIKVCEFQFCNDPFLGVTSNHKNNKSWMESEFRVYSSANNCTFEYVSQPFLMDL
OK025270/B.1.621/Mu                 NNATNVVIKVCEFQFCNDPFLGVTSNHKNNKSWMESEFRVYSSANNCTFEYVSQPFLMDL
```

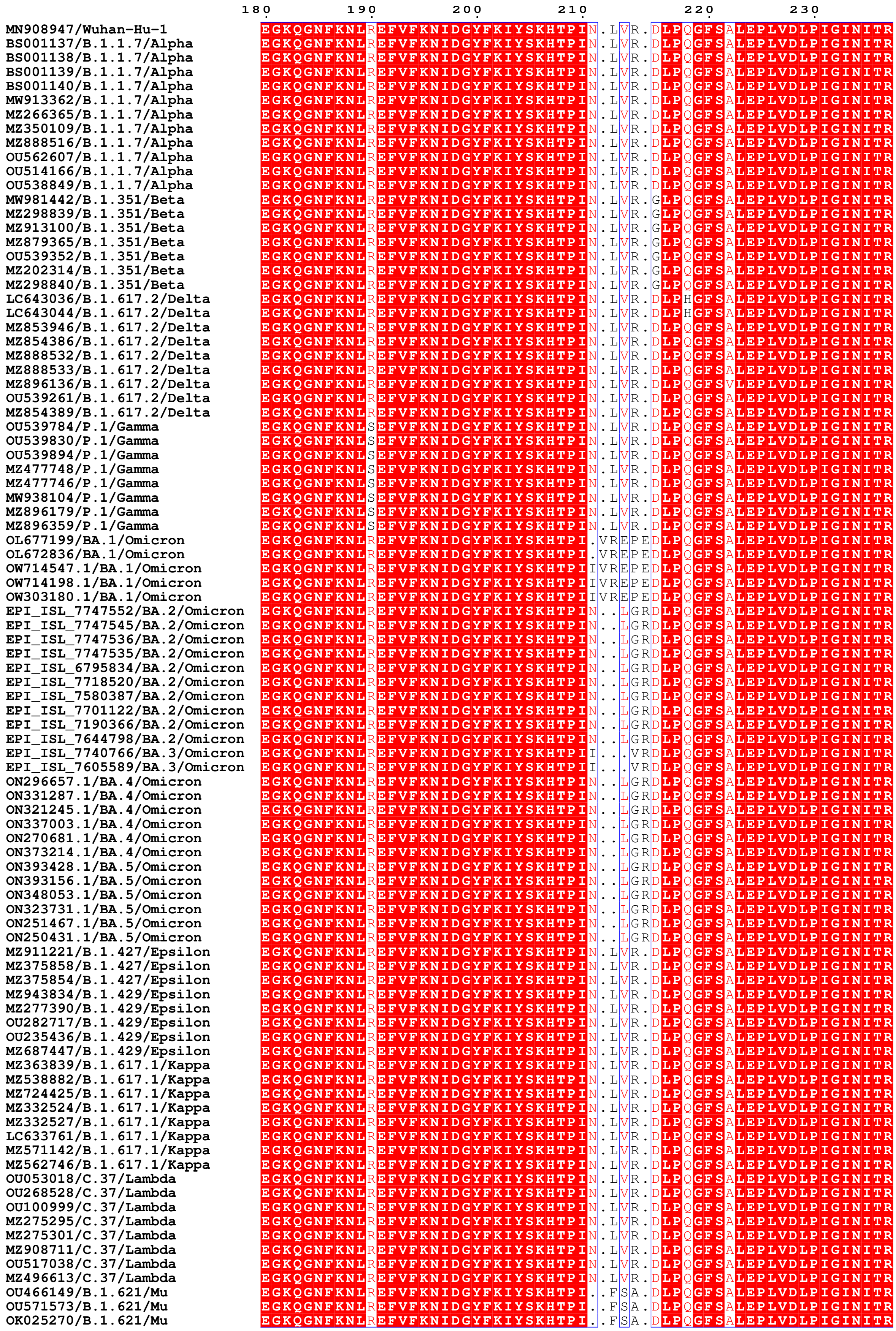
180 190 200 210 220 230
MN908947/Wuhan-Hu-1 EGKQGNFKNLREFVFKNIDGYFKIYSKHTPIN.LVR.DLPQGFSALEPLVDLPIGINITR
BS001137/B.1.1.7/Alpha EGKQGNFKNLREFVFKNIDGYFKIYSKHTPIN.LVR.DLPQGFSALEPLVDLPIGINITR
BS001138/B.1.1.7/Alpha EGKQGNFKNLREFVFKNIDGYFKIYSKHTPIN.LVR.DLPQGFSALEPLVDLPIGINITR
BS001139/B.1.1.7/Alpha EGKQGNFKNLREFVFKNIDGYFKIYSKHTPIN.LVR.DLPQGFSALEPLVDLPIGINITR
BS001140/B.1.1.7/Alpha EGKQGNFKNLREFVFKNIDGYFKIYSKHTPIN.LVR.DLPQGFSALEPLVDLPIGINITR
MW913362/B.1.1.7/Alpha EGKQGNFKNLREFVFKNIDGYFKIYSKHTPIN.LVR.DLPQGFSALEPLVDLPIGINITR
MZ266365/B.1.1.7/Alpha EGKQGNFKNLREFVFKNIDGYFKIYSKHTPIN.LVR.DLPQGFSALEPLVDLPIGINITR
MZ350109/B.1.1.7/Alpha EGKQGNFKNLREFVFKNIDGYFKIYSKHTPIN.LVR.DLPQGFSALEPLVDLPIGINITR
MZ888516/B.1.1.7/Alpha EGKQGNFKNLREFVFKNIDGYFKIYSKHTPIN.LVR.DLPQGFSALEPLVDLPIGINITR
OU562607/B.1.1.7/Alpha EGKQGNFKNLREFVFKNIDGYFKIYSKHTPIN.LVR.DLPQGFSALEPLVDLPIGINITR
OU514166/B.1.1.7/Alpha EGKQGNFKNLREFVFKNIDGYFKIYSKHTPIN.LVR.DLPQGFSALEPLVDLPIGINITR
OU538849/B.1.1.7/Alpha EGKQGNFKNLREFVFKNIDGYFKIYSKHTPIN.LVR.DLPQGFSALEPLVDLPIGINITR
MW981442/B.1.351/Beta EGKQGNFKNLREFVFKNIDGYFKIYSKHTPIN.LVR.GLPQGFSALEPLVDLPIGINITR
MZ298839/B.1.351/Beta EGKQGNFKNLREFVFKNIDGYFKIYSKHTPIN.LVR.GLPQGFSALEPLVDLPIGINITR
MZ913100/B.1.351/Beta EGKQGNFKNLREFVFKNIDGYFKIYSKHTPIN.LVR.GLPQGFSALEPLVDLPIGINITR
MZ879365/B.1.351/Beta EGKQGNFKNLREFVFKNIDGYFKIYSKHTPIN.LVR.GLPQGFSALEPLVDLPIGINITR
OU539352/B.1.351/Beta EGKQGNFKNLREFVFKNIDGYFKIYSKHTPIN.LVR.GLPQGFSALEPLVDLPIGINITR
MZ202314/B.1.351/Beta EGKQGNFKNLREFVFKNIDGYFKIYSKHTPIN.LVR.GLPQGFSALEPLVDLPIGINITR
MZ298840/B.1.351/Beta EGKQGNFKNLREFVFKNIDGYFKIYSKHTPIN.LVR.GLPQGFSALEPLVDLPIGINITR
LC643036/B.1.617.2/Delta EGKQGNFKNLREFVFKNIDGYFKIYSKHTPIN.LVR.DLPHGFSALEPLVDLPIGINITR
LC643044/B.1.617.2/Delta EGKQGNFKNLREFVFKNIDGYFKIYSKHTPIN.LVR.DLPHGFSALEPLVDLPIGINITR
MZ853946/B.1.617.2/Delta EGKQGNFKNLREFVFKNIDGYFKIYSKHTPIN.LVR.DLPQGFSALEPLVDLPIGINITR
MZ854386/B.1.617.2/Delta EGKQGNFKNLREFVFKNIDGYFKIYSKHTPIN.LVR.DLPQGFSALEPLVDLPIGINITR
MZ888532/B.1.617.2/Delta EGKQGNFKNLREFVFKNIDGYFKIYSKHTPIN.LVR.DLPQGFSALEPLVDLPIGINITR
MZ888533/B.1.617.2/Delta EGKQGNFKNLREFVFKNIDGYFKIYSKHTPIN.LVR.DLPQGFSALEPLVDLPIGINITR
MZ896136/B.1.617.2/Delta EGKQGNFKNLREFVFKNIDGYFKIYSKHTPIN.LVR.DLPQGFSVLEPLVDLPIGINITR
OU539261/B.1.617.2/Delta EGKQGNFKNLREFVFKNIDGYFKIYSKHTPIN.LVR.DLPQGFSALEPLVDLPIGINITR
MZ854389/B.1.617.2/Delta EGKQGNFKNLREFVFKNIDGYFKIYSKHTPIN.LVR.DLPQGFSALEPLVDLPIGINITR
OU539784/P.1/Gamma EGKQGNFKNLSEFVFKNIDGYFKIYSKHTPIN.LVR.DLPQGFSALEPLVDLPIGINITR
OU539830/P.1/Gamma EGKQGNFKNLSEFVFKNIDGYFKIYSKHTPIN.LVR.DLPQGFSALEPLVDLPIGINITR
OU539894/P.1/Gamma EGKQGNFKNLSEFVFKNIDGYFKIYSKHTPIN.LVR.DLPQGFSALEPLVDLPIGINITR
MZ477748/P.1/Gamma EGKQGNFKNLSEFVFKNIDGYFKIYSKHTPIN.LVR.DLPQGFSALEPLVDLPIGINITR
MZ477746/P.1/Gamma EGKQGNFKNLSEFVFKNIDGYFKIYSKHTPIN.LVR.DLPQGFSALEPLVDLPIGINITR
MW938104/P.1/Gamma EGKQGNFKNLSEFVFKNIDGYFKIYSKHTPIN.LVR.DLPQGFSALEPLVDLPIGINITR
MZ896179/P.1/Gamma EGKQGNFKNLSEFVFKNIDGYFKIYSKHTPIN.LVR.DLPQGFSALEPLVDLPIGINITR
MZ896359/P.1/Gamma EGKQGNFKNLSEFVFKNIDGYFKIYSKHTPIN.LVR.DLPQGFSALEPLVDLPIGINITR
OL677199/BA.1/Omicron EGKQGNFKNLREFVFKNIDGYFKIYSKHTPI.VREPEDLPQGFSALEPLVDLPIGINITR
OL672836/BA.1/Omicron EGKQGNFKNLREFVFKNIDGYFKIYSKHTPI.VREPEDLPQGFSALEPLVDLPIGINITR
OW714547.1/BA.1/Omicron EGKQGNFKNLREFVFKNIDGYFKIYSKHTPIIVREPEDLPQGFSALEPLVDLPIGINITR
OW714198.1/BA.1/Omicron EGKQGNFKNLREFVFKNIDGYFKIYSKHTPIIVREPEDLPQGFSALEPLVDLPIGINITR
OW303180.1/BA.1/Omicron EGKQGNFKNLREFVFKNIDGYFKIYSKHTPIIVREPEDLPQGFSALEPLVDLPIGINITR
EPI_ISL_7747552/BA.2/Omicron EGKQGNFKNLREFVFKNIDGYFKIYSKHTPIN..LGRDLPQGFSALEPLVDLPIGINITR
EPI_ISL_7747545/BA.2/Omicron EGKQGNFKNLREFVFKNIDGYFKIYSKHTPIN..LGRDLPQGFSALEPLVDLPIGINITR
EPI_ISL_7747536/BA.2/Omicron EGKQGNFKNLREFVFKNIDGYFKIYSKHTPIN..LGRDLPQGFSALEPLVDLPIGINITR
EPI_ISL_7747535/BA.2/Omicron EGKQGNFKNLREFVFKNIDGYFKIYSKHTPIN..LGRDLPQGFSALEPLVDLPIGINITR
EPI_ISL_6795834/BA.2/Omicron EGKQGNFKNLREFVFKNIDGYFKIYSKHTPIN..LGRDLPQGFSALEPLVDLPIGINITR
EPI_ISL_7718520/BA.2/Omicron EGKQGNFKNLREFVFKNIDGYFKIYSKHTPIN..LGRDLPQGFSALEPLVDLPIGINITR
EPI_ISL_7580387/BA.2/Omicron EGKQGNFKNLREFVFKNIDGYFKIYSKHTPIN..LGRDLPQGFSALEPLVDLPIGINITR
EPI_ISL_7701122/BA.2/Omicron EGKQGNFKNLREFVFKNIDGYFKIYSKHTPIN..LGRDLPQGFSALEPLVDLPIGINITR
EPI_ISL_7190366/BA.2/Omicron EGKQGNFKNLREFVFKNIDGYFKIYSKHTPIN..LGRDLPQGFSALEPLVDLPIGINITR
EPI_ISL_7644798/BA.2/Omicron EGKQGNFKNLREFVFKNIDGYFKIYSKHTPIN..LGRDLPQGFSALEPLVDLPIGINITR
EPI_ISL_7740766/BA.3/Omicron EGKQGNFKNLREFVFKNIDGYFKIYSKHTPII...VRDLPQGFSALEPLVDLPIGINITR
EPI_ISL_7605589/BA.3/Omicron EGKQGNFKNLREFVFKNIDGYFKIYSKHTPII...VRDLPQGFSALEPLVDLPIGINITR
ON296657.1/BA.4/Omicron EGKQGNFKNLREFVFKNIDGYFKIYSKHTPIN..LGRDLPQGFSALEPLVDLPIGINITR
ON331287.1/BA.4/Omicron EGKQGNFKNLREFVFKNIDGYFKIYSKHTPIN..LGRDLPQGFSALEPLVDLPIGINITR
ON321245.1/BA.4/Omicron EGKQGNFKNLREFVFKNIDGYFKIYSKHTPIN..LGRDLPQGFSALEPLVDLPIGINITR
ON337003.1/BA.4/Omicron EGKQGNFKNLREFVFKNIDGYFKIYSKHTPIN..LGRDLPQGFSALEPLVDLPIGINITR
ON270681.1/BA.4/Omicron EGKQGNFKNLREFVFKNIDGYFKIYSKHTPIN..LGRDLPQGFSALEPLVDLPIGINITR
ON373214.1/BA.4/Omicron EGKQGNFKNLREFVFKNIDGYFKIYSKHTPIN..LGRDLPQGFSALEPLVDLPIGINITR
ON393428.1/BA.5/Omicron EGKQGNFKNLREFVFKNIDGYFKIYSKHTPIN..LGRDLPQGFSALEPLVDLPIGINITR
ON393156.1/BA.5/Omicron EGKQGNFKNLREFVFKNIDGYFKIYSKHTPIN..LGRDLPQGFSALEPLVDLPIGINITR
ON348053.1/BA.5/Omicron EGKQGNFKNLREFVFKNIDGYFKIYSKHTPIN..LGRDLPQGFSALEPLVDLPIGINITR
ON323731.1/BA.5/Omicron EGKQGNFKNLREFVFKNIDGYFKIYSKHTPIN..LGRDLPQGFSALEPLVDLPIGINITR
ON251467.1/BA.5/Omicron EGKQGNFKNLREFVFKNIDGYFKIYSKHTPIN..LGRDLPQGFSALEPLVDLPIGINITR
ON250431.1/BA.5/Omicron EGKQGNFKNLREFVFKNIDGYFKIYSKHTPIN..LGRDLPQGFSALEPLVDLPIGINITR
MZ911221/B.1.427/Epsilon EGKQGNFKNLREFVFKNIDGYFKIYSKHTPIN.LVR.DLPQGFSALEPLVDLPIGINITR
MZ375858/B.1.427/Epsilon EGKQGNFKNLREFVFKNIDGYFKIYSKHTPIN.LVR.DLPQGFSALEPLVDLPIGINITR
MZ375854/B.1.427/Epsilon EGKQGNFKNLREFVFKNIDGYFKIYSKHTPIN.LVR.DLPQGFSALEPLVDLPIGINITR
MZ943834/B.1.429/Epsilon EGKQGNFKNLREFVFKNIDGYFKIYSKHTPIN.LVR.DLPQGFSALEPLVDLPIGINITR
MZ277390/B.1.429/Epsilon EGKQGNFKNLREFVFKNIDGYFKIYSKHTPIN.LVR.DLPQGFSALEPLVDLPIGINITR
OU282717/B.1.429/Epsilon EGKQGNFKNLREFVFKNIDGYFKIYSKHTPIN.LVR.DLPQGFSALEPLVDLPIGINITR
OU235436/B.1.429/Epsilon EGKQGNFKNLREFVFKNIDGYFKIYSKHTPIN.LVR.DLPQGFSALEPLVDLPIGINITR
MZ687447/B.1.429/Epsilon EGKQGNFKNLREFVFKNIDGYFKIYSKHTPIN.LVR.DLPQGFSALEPLVDLPIGINITR
MZ363839/B.1.617.1/Kappa EGKQGNFKNLREFVFKNIDGYFKIYSKHTPIN.LVR.DLPQGFSALEPLVDLPIGINITR
MZ538882/B.1.617.1/Kappa EGKQGNFKNLREFVFKNIDGYFKIYSKHTPIN.LVR.DLPQGFSALEPLVDLPIGINITR
MZ724425/B.1.617.1/Kappa EGKQGNFKNLREFVFKNIDGYFKIYSKHTPIN.LVR.DLPQGFSALEPLVDLPIGINITR
MZ332524/B.1.617.1/Kappa EGKQGNFKNLREFVFKNIDGYFKIYSKHTPIN.LVR.DLPQGFSALEPLVDLPIGINITR
MZ332527/B.1.617.1/Kappa EGKQGNFKNLREFVFKNIDGYFKIYSKHTPIN.LVR.DLPQGFSALEPLVDLPIGINITR
LC633761/B.1.617.1/Kappa EGKQGNFKNLREFVFKNIDGYFKIYSKHTPIN.LVR.DLPQGFSALEPLVDLPIGINITR
MZ571142/B.1.617.1/Kappa EGKQGNFKNLREFVFKNIDGYFKIYSKHTPIN.LVR.DLPQGFSALEPLVDLPIGINITR
MZ562746/B.1.617.1/Kappa EGKQGNFKNLREFVFKNIDGYFKIYSKHTPIN.LVR.DLPQGFSALEPLVDLPIGINITR
OU053018/C.37/Lambda EGKQGNFKNLREFVFKNIDGYFKIYSKHTPIN.LVR.DLPQGFSALEPLVDLPIGINITR
OU268528/C.37/Lambda EGKQGNFKNLREFVFKNIDGYFKIYSKHTPIN.LVR.DLPQGFSALEPLVDLPIGINITR
OU100999/C.37/Lambda EGKQGNFKNLREFVFKNIDGYFKIYSKHTPIN.LVR.DLPQGFSALEPLVDLPIGINITR
MZ275295/C.37/Lambda EGKQGNFKNLREFVFKNIDGYFKIYSKHTPIN.LVR.DLPQGFSALEPLVDLPIGINITR
MZ275301/C.37/Lambda EGKQGNFKNLREFVFKNIDGYFKIYSKHTPIN.LVR.DLPQGFSALEPLVDLPIGINITR
MZ908711/C.37/Lambda EGKQGNFKNLREFVFKNIDGYFKIYSKHTPIN.LVR.DLPQGFSALEPLVDLPIGINITR
OU517038/C.37/Lambda EGKQGNFKNLREFVFKNIDGYFKIYSKHTPIN.LVR.DLPQGFSALEPLVDLPIGINITR
MZ496613/C.37/Lambda EGKQGNFKNLREFVFKNIDGYFKIYSKHTPIN.LVR.DLPQGFSALEPLVDLPIGINITR
OU466149/B.1.621/Mu EGKQGNFKNLREFVFKNIDGYFKIYSKHTPI..FSA.DLPQGFSALEPLVDLPIGINITR
OU571573/B.1.621/Mu EGKQGNFKNLREFVFKNIDGYFKIYSKHTPI..FSA.DLPQGFSALEPLVDLPIGINITR
OK025270/B.1.621/Mu EGKQGNFKNLREFVFKNIDGYFKIYSKHTPI..FSA.DLPQGFSALEPLVDLPIGINITR

```
                                 240       250       260       270       280       290
                                   .         .         .         .         .         .
MN908947/Wuhan-Hu-1             FQTLLALHRSYLTPGDSSSGWTAGAAAYYVGYLQPRTFLLKYNENGTITDAVDCALDPLS
BS001137/B.1.1.7/Alpha          FQTLLALHRSYLTPGDSSSGWTAGAAAYYVGYLQPRTFLLKYNENGTITDAVDCALDPLS
BS001138/B.1.1.7/Alpha          FQTLLALHRSYLTPGDSSSGWTAGAAAYYVGYLQPRTFLLKYNENGTITDAVDCALDPLS
BS001139/B.1.1.7/Alpha          FQTLLALHRSYLTPGDSSSGWTAGAAAYYVGYLQPRTFLLKYNENGTITDAVDCALDPLS
BS001140/B.1.1.7/Alpha          FQTLLALHRSYLTPGDSSSGWTAGAAAYYVGYLQPRTFLLKYNENGTITDAVDCALDPLS
MW913362/B.1.1.7/Alpha          FQTLLALHRSYLTPGDSSSGWTAGAAAYYVGYLQPRTFLLKYNENGTITDAVDCALDPLS
MZ266365/B.1.1.7/Alpha          FQTLLALHRSYLTPGDSSSGWTAGAAAYYVGYLQPRTFLLKYNENGTITDAVDCALDPLS
MZ350109/B.1.1.7/Alpha          FQTLLALHRSYLTPGDSSSGWTAGAAAYYVGYLQPRTFLLKYNENGTITDAVDCALDPLS
MZ888516/B.1.1.7/Alpha          FQTLLALHRSYLTPGDSSSGWTAGAAAYYVGYLQPRTFLLKYNENGTITDAVDCALDPLS
OU562607/B.1.1.7/Alpha          FQTLLALHRSYLTPGDSSSGWTAGAAAYYVGYLQPRTFLLKYNENGTITDAVDCALDPLS
OU514166/B.1.1.7/Alpha          FQTLLALHRSYLTPGDSSSGWTAGAAAYYVGYLQPRTFLLKYNENGTITDAVDCALDPLS
OU538849/B.1.1.7/Alpha          FQTLLALHRSYLTPGDSSSGWTAGAAAYYVGYLQPRTFLLKYNENGTITDAVDCALDPLS
MW981442/B.1.351/Beta           FQT...LHRSYLTPGDSSSGWTAGAAAYYVGYLQPRTFLLKYNENGTITDAVDCALDPLS
MZ298839/B.1.351/Beta           FQT...LHRSYLTPGDSSSGWTAGAAAYYVGYLQPRTFLLKYNENGTITDAVDCALDPLS
MZ913100/B.1.351/Beta           FQT...LHRSYLTPGDSSSGWTAGAAAYYVGYLQPRTFLLKYNENGTITDAVDCALDPLS
MZ879365/B.1.351/Beta           FQT...LHRSYLTPGDSSSGWTAGAAAYYVGYLQPRTFLLKYNENGTITDAVDCALDPLS
OU539352/B.1.351/Beta           FQT...LHRSYLTPGDSSSGWTAGAAAYYVGYLQPRTFLLKYNENGTITDAVDCALDPLS
MZ202314/B.1.351/Beta           FQT...LHRSYLTPGDSSSGWTAGAAAYYVGYLQPRTFLLKYNENGTITDAVDCALDPLS
MZ298840/B.1.351/Beta           FQT...LHRSYLTPGDSSSGWTAGAAAYYVGYLQPRTFLLKYNENGTITDAVDCALDPLS
LC643036/B.1.617.2/Delta        FQTLLALHRSYLTPGDSSSGWTAGAAAYYVGYLQPRTFLLKYNENGTITDAVDCALDPLS
LC643044/B.1.617.2/Delta        FQTLLALHRSYLTPGDSSSGWTAGAAAYYVGYLQPRTFLLKYNENGTITDAVDCALDPLS
MZ853946/B.1.617.2/Delta        FQTLLALHRSYLTPGDSSSGWTAGAAAYYVGYLQPRTFLLKYNENGTITDAVDCALDPLS
MZ854386/B.1.617.2/Delta        FQTLLALHRSYLTPGDSSSGWTAGAAAYYVGYLQPRTFLLKYNENGTITDAVDCALDPLS
MZ888532/B.1.617.2/Delta        FQTLLALHRSYLTPGDSSSGWTAGAAAYYVGYLQPRTFLLKYNENGTITDAVDCALDPLS
MZ888533/B.1.617.2/Delta        FQTLLALHRSYLTPGDSSSGWTAGAAAYYVGYLQPRTFLLKYNENGTITDAVDCALDPLS
MZ896136/B.1.617.2/Delta        FQTLLALHRSYLTPGDSSSGWTAGAAAYYVGYLQPRTFLLKYNENGTITDAIDCALDPLS
OU539261/B.1.617.2/Delta        FQTLLALHRSYLTPGDSSSGWTAGAAAYYVGYLQPRTFLLKYNENGTITDAVDCALDPLS
MZ854389/B.1.617.2/Delta        FQTLLALHRSYLTPGDSSSGWTAGAAAYYVGYLQPRTFLLKYNENGTITDAVDCALDPLS
OU539784/P.1/Gamma              FQTLLALHRSYLTPGDSSSGWTAGAAAYYVGYLQPRTFLLKYNENGTITDAVDCALDPLS
OU539830/P.1/Gamma              FQTLLALHRSYLTPGDSSSGWTAGAAAYYVGYLQPRTFLLKYNENGTITDAVDCALDPLS
OU539894/P.1/Gamma              FQTLLALHRSYLTPGDSSSGWTAGAAAYYVGYLQPRTFLLKYNENGTITDAVDCALDPLS
MZ477748/P.1/Gamma              FQTLLALHRSYLTPGDSSSGWTASAAAYYVGYLQPRTFLLKYNENGTITDAVDCALDPLS
MZ477746/P.1/Gamma              FQTLLALHRSYLTPGDSSSGWTAGAAAYYVGYLQPRTFLLKYNENGTITDAVDCALDPLS
MW938104/P.1/Gamma              FQTLLALHRSYLTPGDSSSGWTAGAAAYYVGYLQPRTFLLKYNENGTITDAVDCALDPLS
MZ896179/P.1/Gamma              FQTLLALHRSYLTPGDSSSGWTAGAAAYYVGYLQPRTFLLKYNENGTITDAVDCALDPLS
MZ896359/P.1/Gamma              FQTLLALHRSYLTPGDSSSGWTAGAAAYYVGYLQPRTFLLKYNENGTITDAVDCALDPLS
OL677199/BA.1/Omicron           FQTLLALHRSYLTPGDSSSGWTAGAAAYYVGYLQPRTFLLKYNENGTITDAVDCALDPLS
OL672836/BA.1/Omicron           FQTLLALHRSYLTPGDSSSGWTAGAAAYYVGYLQPRTFLLKYNENGTITDAVDCALDPLS
OW714547.1/BA.1/Omicron         FQTLLALHRSYLTPGDSSSGWTAGAAAYYVGYLQPRTFLLKYNENGTITDAVDCALDPLS
OW714198.1/BA.1/Omicron         FQTLLALHRSYLTPGDSSSGWTAGAAAYYVGYLQPRTFLLKYNENGTITDAVDCALDPLS
OW303180.1/BA.1/Omicron         FQTLLALHRSYLTPGDSSSGWTAGAAAYYVGYLQPRTFLLKYNENGTITDAVDCALDPLS
EPI_ISL_7747552/BA.2/Omicron    FQTLLALHRSYLTPGDSSSGWTAGAAAYYVGYLQPRTFLLKYNENGTITDAVDCALDPLS
EPI_ISL_7747545/BA.2/Omicron    FQTLLALHRSYLTPGDSSSGWTAGAAAYYVGYLQPRTFLLKYNENGTITDAVDCALDPLS
EPI_ISL_7747536/BA.2/Omicron    FQTLLALHRSYLTPGDSSSGWTAGAAAYYVGYLQPRTFLLKYNENGTITDAVDCALDPLS
EPI_ISL_7747535/BA.2/Omicron    FQTLLALHRSYLTPGDSSSGWTAGAAAYYVGYLQPRTFLLKYNENGTITDAVDCALDPLS
EPI_ISL_6795834/BA.2/Omicron    FQTLLALHRSYLTPGDSSSGWTAGAAAYYVGYLQPRTFLLKYNENGTITDAVDCALDPLS
EPI_ISL_7718520/BA.2/Omicron    FQTLLALHRSYLTPGDSSSGWTAGAAAYYVGYLQPRTFLLKYNENGTITDAVDCALDPLS
EPI_ISL_7580387/BA.2/Omicron    FQTLLALHRSYLTPGDSSSGWTAGAAAYYVGYLQPRTFLLKYNENGTITDAVDCALDPLS
EPI_ISL_7701122/BA.2/Omicron    FQTLLALHRSYLTPGDSSSGWTAGAAAYYVGYLQPRTFLLKYNENGTITDAVDCALDPLS
EPI_ISL_7190366/BA.2/Omicron    FQTLLALHRSYLTPGDSSSGWTAGAAAYYVGYLQPRTFLLKYNENGTITDAVDCALDPLS
EPI_ISL_7644798/BA.2/Omicron    FQTLLALHRSYLTPGDSSSGWTAGAAAYYVGYLQPRTFLLKYNENGTITDAVDCALDPLS
EPI_ISL_7740766/BA.3/Omicron    FQTLLALHRSYLTPGDSSSGWTAGAAAYYVGYLQPRTFLLKYNENGTITDAVDCALDPLS
EPI_ISL_7605589/BA.3/Omicron    FQTLLALHRSYLTPGDSSSGWTAGAAAYYVGYLQPRTFLLKYNENGTITDAVDCALDPLS
ON296657.1/BA.4/Omicron         FQTLLALHRSYLTPGDSSSGWTAGAAAYYVGYLQPRTFLLKYNENGTITDAVDCALDPLS
ON331287.1/BA.4/Omicron         FQTLLALHRSYLTPGDSSSGWTAGAAAYYVGYLQPRTFLLKYNENGTITDAVDCALDPLS
ON321245.1/BA.4/Omicron         FQTLLALHRSYLTPGDSSSGWTAGAAAYYVGYLQPRTFLLKYNENGTITDAVDCALDPLS
ON337003.1/BA.4/Omicron         FQTLLALHRSYLTPGDSSSGWTAGAAAYYVGYLQPRTFLLKYNENGTITDAVDCALDPLS
ON270681.1/BA.4/Omicron         FQTLLALHRSYLTPGDSSSGWTAGAAAYYVGYLQPRTFLLKYNENGTITDAVDCALDPLS
ON373214.1/BA.4/Omicron         FQTLLALHRSYLTPGDSSSGWTAGAAAYYVGYLQPRTFLLKYNENGTITDAVDCALDPLS
ON393428.1/BA.5/Omicron         FQTLLALHRSYLTPGDSSSGWTAGAAAYYVGYLQPRTFLLKYNENGTITDAVDCALDPLS
ON393156.1/BA.5/Omicron         FQTLLALHRSYLTPGDSSSGWTAGAAAYYVGYLQPRTFLLKYNENGTITDAVDCALDPLS
ON348053.1/BA.5/Omicron         FQTLLALHRSYLTPGDSSSGWTAGAAAYYVGYLQPRTFLLKYNENGTITDAVDCALDPLS
ON323731.1/BA.5/Omicron         FQTLLALHRSYLTPGDSSSGWTAGAAAYYVGYLQPRTFLLKYNENGTITDAVDCALDPLS
ON251467.1/BA.5/Omicron         FQTLLALHRSYLTPGDSSSGWTAGAAAYYVGYLQPRTFLLKYNENGTITDAVDCALDPLS
ON250431.1/BA.5/Omicron         FQTLLALHRSYLTPGDSSSGWTAGAAAYYVGYLQPRTFLLKYNENGTITDAVDCALDPLS
MZ911221/B.1.427/Epsilon        FQTLLALHRSYLTPGDSSSGWTAGAAAYYVGYLQPRTFLLKYNENGTITDAVDCALDPLS
MZ375858/B.1.427/Epsilon        FQTLLALHRSYLTPGDSSSGLTAGAAAYYVGYLQPRTFLLKYNENGTITDAVDCALDPLS
MZ375854/B.1.427/Epsilon        FQTLLALHRSYLTPGDSSSGLTAGAAAYYVGYLQPRTFLLKYNENGTITDAVDCALDPLS
MZ943834/B.1.429/Epsilon        FQTLLALHRSYLTPGDSSSGWTAGAAAYYVGYLQPRTFLLKYNENGTITDAVDCALDPLS
MZ277390/B.1.429/Epsilon        FQTLLALHRSYLTPGDSSSGWTAGAAAYYVGYLQPRTFLLKYNENGTITDAVDCALDPLS
OU282717/B.1.429/Epsilon        FQTLLALHRSYLTPGDSSSGWTAGAAAYYVGYLQPRTFLLKYNENGTITDAVDCALDPLS
OU235436/B.1.429/Epsilon        FQTLLALHRSYLTPGDSSSGWTAGAAAYYVGYLQPRTFLLKYNENGTITDAVDCALDPLS
MZ687447/B.1.429/Epsilon        FQTLLALHRSYLTPGDSSSGWTAGAAAYYVGYLQPRTFLLKYNENGTITDAVDCALDPLS
MZ363839/B.1.617.1/Kappa        FQTLLALHRSYLTPGDSSSGWTAGAAAYYVGYLQPRTFLLKYNENGTITDAVDCALDPLS
MZ538882/B.1.617.1/Kappa        FQTLLALHRSYLTPGDSSSGWTAGAAAYYVGYLQPRTFLLKYNENGTITDAVDCALDPLS
MZ724425/B.1.617.1/Kappa        FQTLLALHRSYLTPGDSSSGWTAGAAAYYVGYLQPRTFLLKYNENGTITDAVDCALDPLS
MZ332524/B.1.617.1/Kappa        FQTLLALHRSYLTPGDSSSGWTAGAAAYYVGYLQPRTFLLKYNENGTITDAVDCALDPLS
MZ332527/B.1.617.1/Kappa        FQTLLALHRSYLTPGDSSSGWTAGAAAYYVGYLQPRTFLLKYNENGTITDAVDCALDPLS
LC633761/B.1.617.1/Kappa        FQTLLALHRSYLTPGDSSSGWTAGAAAYYVGYLQPRTFLLKYNENGTITDAVDCALDPLS
MZ571142/B.1.617.1/Kappa        FQTLLALHRSYLTPGDSSSGWTAGAAAYYVGYLQPRTFLLKYNENGTITDAVDCALDPLS
MZ562746/B.1.617.1/Kappa        FQTLLALHRSYLTPGDSSSGWTAGAAAYYVGYLQPRTFLLKYNENGTITDAVDCALDPLS
OU053018/C.37/Lambda            FQTLLALHN.......SSSGWTAGAAAYYVGYLQPRTFLLKYNENGTITDAVDCALDPLS
OU268528/C.37/Lambda            FQTLLALHN.......SSSGWTAGAAAYYVGYLQPRTFLLKYNENGTITDAVDCALDPLS
OU100999/C.37/Lambda            FQTLLALHN.......SSSGWTAGAAAYYVGYLQPRTFLLKYNENGTITDAVDCALDPLS
MZ275295/C.37/Lambda            FQTLLALHN.......SSSGWTAGAAAYYVGYLQPRTFLLKYNENGTITDAVDCALDPLS
MZ275301/C.37/Lambda            FQTLLALHN.......SSSGWTAGAAAYYVGYLQPRTFLLKYNENGTITDAVDCALDPLS
MZ908711/C.37/Lambda            FQTLLALHN.......SSSGWTAGAAAYYVGYLQPRTFLLKYNENGTITDAVDCALDPLS
OU517038/C.37/Lambda            FQTLLALHN.......SSSGWTAGAAAYYVGYLQPRTFLLKYNENGTITDAVDCALDPLS
MZ496613/C.37/Lambda            FQTLLALHN.......SSSGWTAGAAAYYVGYLQPRTFLLKYNENGTITDAVDCALDPLS
OU466149/B.1.621/Mu             FQTLLALHRSYLTPGDSSSGWTAGAAAYYVGYLQPRTFLLKYNENGTITDAVDCALDPLS
OU571573/B.1.621/Mu             FQTLLALHRSYLTPGDSSSGWTAGAAAYYVGYLQPRTFLLKYNENGTITDAVDCALDPLS
OK025270/B.1.621/Mu             FQTLLALHRSYLTPGDSSSGWTAGAAAYYVGYLQPRTFLLKYNENGTITDAVDCALDPLS
```

	300 . 310 . 320 . 330 . 340 . 350 .
MN908947/Wuhan-Hu-1	ETKCTLKSFTVEKGIYQTSNFRVQPTESIVRFPNITNLCPFGEVFNATRFASVYAWNRKR
BS001137/B.1.1.7/Alpha	ETKCTLKSFTVEKGIYQTSNFRVQPTESIVRFPNITNLCPFGEVFNATRFASVYAWNRKR
BS001138/B.1.1.7/Alpha	ETKCTLKSFTVEKGIYQTSNFRVQPTESIVRFPNITNLCPFGEVFNATRFASVYAWNRKR
BS001139/B.1.1.7/Alpha	ETKCTLKSFTVEKGIYQTSNFRVQPTESIVRFPNITNLCPFGEVFNATRFASVYAWNRKR
BS001140/B.1.1.7/Alpha	ETKCTLKSFTVEKGIYQTSNFRVQPTESIVRFPNITNLCPFGEVFNATRFASVYAWNRKR
MW913362/B.1.1.7/Alpha	ETKCTLKSFTVEKGIYQTSNFRVQPTESIVRFPNITNLCPFGEVFNATRFASVYAWNRKR
MZ266365/B.1.1.7/Alpha	ETKCTLKSFTVEKGIYQTSNFRVQPTESIVRFPNITNLCPFGEVFNATRFASVYAWNRKR
MZ350109/B.1.1.7/Alpha	ETKCTLKSFTVEKGIYQTSNFRVQPTESIVRFPNITNLCPFGEVFNATRFASVYAWNRKR
MZ888516/B.1.1.7/Alpha	ETKCTLKSFTVEKGIYQTSNFRVQPTESIVRFPNITNLCPFGEVFNATRFASVYAWNRKR
OU562607/B.1.1.7/Alpha	ETKCTLKSFTVEKGIYQTSNFRVQPTESIVRFPNITNLCPFGEVFNATRFASVYAWNRKR
OU514166/B.1.1.7/Alpha	ETKCTLKSFTVEKGIYQTSNFRVQPTESIVRFPNITNLCPFGEVFNATRFASVYAWNRKR
OU538849/B.1.1.7/Alpha	ETKCTLKSFTVEKGIYQTSNFRVQPTESIVRFPNITNLCPFGEVFNATRFASVYAWNRKR
MW981442/B.1.351/Beta	ETKCTLKSFTVEKGIYQTSNFRVQPTESIVRFPNITNLCPFGEVFNATRFASVYAWNRKR
MZ298839/B.1.351/Beta	ETKCTLKSFTVEKGIYQTSNFRVQPTESIVRFPNITNLCPFGEVFNATRFASVYAWNRKR
MZ913100/B.1.351/Beta	ETKCTLKSFTVEKGIYQTSNFRVQPTESIVRFPNITNLCPFGEVFNATRFASVYAWNRKR
MZ879365/B.1.351/Beta	ETKCTLKSFTVEKGIYQTSNFRVQPTESIVRFPNITNLCPFGEVFNATRFASVYAWNRKR
OU539352/B.1.351/Beta	ETKCTLKSFTVEKGIYQTSNFRVQPTESIVRFPNITNLCPFGEVFNATRFASVYAWNRKR
MZ202314/B.1.351/Beta	ETKCTLKSFTVEKGIYQTSNFRVQPTESIVRFPNITNLCPFGEVFNATRFASVYAWNRKR
MZ298840/B.1.351/Beta	ETKCTLKSFTVEKGIYQTSNFRVQPTESIVRFPNITNLCPFGEVFNATRFASVYAWNRKR
LC643036/B.1.617.2/Delta	ETKCTLKSFTVEKGIYQTSNFRVQPTESIVRFPNITNLCPFGEVFNATRFASVYAWNRKR
LC643044/B.1.617.2/Delta	ETKCTLKSFTVEKGIYQTSNFRVQPTESIVRFPNITNLCPFGEVFNATRFASVYAWNRKR
MZ853946/B.1.617.2/Delta	ETKCTLKSFTVEKGIYQTSNFRVQPTESIVRFPNITNLCPFGEVFNATRFASVYAWNRKR
MZ854386/B.1.617.2/Delta	ETKCTLKSFTVEKGIYQTSNFRVQPTESIVRFPNITNLCPFGEVFNATRFASVYAWNRKR
MZ888532/B.1.617.2/Delta	ETKCTLKSFTVEKGIYQTSNFRVQPTESIVRFPNITNLCPFGEVFNATRFASVYAWNRKR
MZ888533/B.1.617.2/Delta	ETKCTLKSFTVEKGIYQTSNFRVQPTESIVRFPNITNLCPFGEVFNATRFASVYAWNRKR
MZ896136/B.1.617.2/Delta	ETKCTLKSFTVEKGIYQTSNFRVQPTESIVRFPNITNLCPFGEVFNATRFASVYAWNRKR
OU539261/B.1.617.2/Delta	ETKCTLKSFTVEKGIYQTSNFRVQPTESIVRFPNITNLCPFGEVFNATRFASVYAWNRKR
MZ854389/B.1.617.2/Delta	ETKCTLKSFTVEKGIYQTSNFRVQPTESIVRFPNITNLCPFGEVFNATRFASVYAWNRKR
OU539784/P.1/Gamma	ETKCTLKSFTVEKGIYQTSNFRVQPTESIVRFPNITNLCPFGEVFNATRFASVYAWNRKR
OU539830/P.1/Gamma	ETKCTLKSFTVEKGIYQTSNFRVQPTESIVRFPNITNLCPFGEVFNATRFASVYAWNRKR
OU539894/P.1/Gamma	ETKCTLKSFTVEKGIYQTSNFRVQPTESIVRFPNITNLCPFGEVFNATRFASVYAWNRKR
MZ477748/P.1/Gamma	ETKCTLKSFTVEKGIYQTSNFRVQPTESIVRFPNITNLCPFGEVFNATRFASVYAWNRKR
MZ477746/P.1/Gamma	ETKCTLKSFTVEKGIYQTSNFRVQPTESIVRFPNITNLCPFGEVFNATRFASVYAWNRKR
MW938104/P.1/Gamma	ETKCTLKSFTVEKGIYQTSNFRVQPTESIVRFPNITNLCPFGEVFNATRFASVYAWNRKR
MZ896179/P.1/Gamma	ETKCTLKSFTVEKGIYQTSNFRVQPTESIVRFPNITNLCPFGEVFNATRFASVYAWNRKR
MZ896359/P.1/Gamma	ETKCTLKSFTVEKGIYQTSNFRVQPTESIVRFPNITNLCPFGEVFNATRFASVYAWNRKR
OL677199/BA.1/Omicron	ETKCTLKSFTVEKGIYQTSNFRVQPTESIVRFPNITNLCPFDEVFNATRFASVYAWNRKR
OL672836/BA.1/Omicron	ETKCTLKSFTVEKGIYQTSNFRVQPTESIVRFPNITNLCPFDEVFNATRFASVYAWNRKR
OW714547.1/BA.1/Omicron	ETKCTLKSFTVEKGIYQTSNFRVQPTESIVRFPNITNLCPFDEVFNATRFASVYAWNRKR
OW714198.1/BA.1/Omicron	ETKCTLKSFTVEKGIYQTSNFRVQPTESIVRFPNITNLCPFDEVFNATRFASVYAWNRKR
OW303180.1/BA.1/Omicron	ETKCTLKSFTVEKGIYQTSNFRVQPTESIVRFPNITNLCPFDEVFNATRFASVYAWNRKR
EPI_ISL_7747552/BA.2/Omicron	ETKCTLKSFTVEKGIYQTSNFRVQPTESIVRFPNITNLCPFDEVFNATRFASVYAWNRKR
EPI_ISL_7747545/BA.2/Omicron	ETKCTLKSFTVEKGIYQTSNFRVQPTESIVRFPNITNLCPFDEVFNATRFASVYAWNRKR
EPI_ISL_7747536/BA.2/Omicron	ETKCTLKSFTVEKGIYQTSNFRVQPTESIVRFPNITNLCPFDEVFNATRFASVYAWNRKR
EPI_ISL_7747535/BA.2/Omicron	ETKCTLKSFTVEKGIYQTSNFRVQPTESIVRFPNITNLCPFDEVFNATRFASVYAWNRKR
EPI_ISL_6795834/BA.2/Omicron	ETKCTLKSFTVEKGIYQTSNFRVQPTESIVRFPNITNLCPFDEVFNATRFASVYAWNRKR
EPI_ISL_7718520/BA.2/Omicron	ETKCTLKSFTVEKGIYQTSNFRVQPTESIVRFPNITNLCPFDEVFNATRFASVYAWNRKR
EPI_ISL_7580387/BA.2/Omicron	ETKCTLKSFTVEKGIYQTSNFRVQPTESIVRFPNITNLCPFDEVFNATRFASVYAWNRKR
EPI_ISL_7701122/BA.2/Omicron	ETKCTLKSFTVEKGIYQTSNFRVQPTESIVRFPNITNLCPFDEVFNATRFASVYAWNRKR
EPI_ISL_7190366/BA.2/Omicron	ETKCTLKSFTVEKGIYQTSNFRVQPTESIVRFPNITNLCPFDEVFNATRFASVYAWNRKR
EPI_ISL_7644798/BA.2/Omicron	ETKCTLKSFTVEKGIYQTSNFRVQPTESIVRFPNITNLCPFDEVFNATRFASVYAWNRKR
EPI_ISL_7740766/BA.3/Omicron	ETKCTLKSFTVEKGIYQTSNFRVQPTESIVRFPNITNLCPFDEVFNATRFASVYAWNRKR
EPI_ISL_7605589/BA.3/Omicron	ETKCTLKSFTVEKGIYQTSNFRVQPTESIVRFPNITNLCPFDEVFNATRFASVYAWNRKR
ON296657.1/BA.4/Omicron	ETKCTLKSFTVEKGIYQTSNFRVQPTESIVRFPNITNLCPFDEVFNATRFASVYAWNRKR
ON331287.1/BA.4/Omicron	ETKCTLKSFTVEKGIYQTSNFRVQPTESIVRFPNITNLCPFDEVFNATRFASVYAWNRKR
ON321245.1/BA.4/Omicron	ETKCTLKSFTVEKGIYQTSNFRVQPTESIVRFPNITNLCPFDEVFNATRFASVYAWNRKR
ON337003.1/BA.4/Omicron	ETKCTLKSFTVEKGIYQTSNFRVQPTESIVRFPNITNLCPFDEVFNATRFASVYAWNRKR
ON270681.1/BA.4/Omicron	ETKCTLKSFTVEKGIYQTSNFRVQPTESIVRFPNITNLCPFDEVFNATRFASVYAWNRKR
ON373214.1/BA.4/Omicron	ETKCTLKSFTVEKGIYQTSNFRVQPTESIVRFPNITNLCPFDEVFNATRFASVYAWNRKR
ON393428.1/BA.5/Omicron	ETKCTLKSFTVEKGIYQTSNFRVQPTESIVRFPNITNLCPFDEVFNATRFASVYAWNRKR
ON393156.1/BA.5/Omicron	ETKCTLKSFTVEKGIYQTSNFRVQPTESIVRFPNITNLCPFDEVFNATRFASVYAWNRKR
ON348053.1/BA.5/Omicron	ETKCTLKSFTVEKGIYQTSNFRVQPTESIVRFPNITNLCPFDEVFNATRFASVYAWNRKR
ON323731.1/BA.5/Omicron	ETKCTLKSFTVEKGIYQTSNFRVQPTESIVRFPNITNLCPFDEVFNATRFASVYAWNRKR
ON251467.1/BA.5/Omicron	ETKCTLKSFTVEKGIYQTSNFRVQPTESIVRFPNITNLCPFDEVFNATRFASVYAWNRKR
ON250431.1/BA.5/Omicron	ETKCTLKSFTVEKGIYQTSNFRVQPTESIVRFPNITNLCPFDEVFNATRFASVYAWNRKR
MZ911221/B.1.427/Epsilon	ETKCTLKSFTVEKGIYQTSNFRVQPTESIVRFPNITNLCPFGEVFNATRFASVYAWNRKR
MZ375858/B.1.427/Epsilon	ETKCTLKSFTVEKGIYQTSNFRVQPTESIVRFPNITNLCPFGEVFNATRFASVYAWNRKR
MZ375854/B.1.427/Epsilon	ETKCTLKSFTVEKGIYQTSNFRVQPTESIVRFPNITNLCPFGEVFNATRFASVYAWNRKR
MZ943834/B.1.429/Epsilon	ETKCTLKSFTVEKGIYQTSNFRVQPTESIVRFPNITNLCPFGEVFNATRFASVYAWNRKR
MZ277390/B.1.429/Epsilon	ETKCTLKSFTVEKGIYQTSNFRVQPTESIVRFPNITNLCPFGEVFNATRFASVYAWNRKR
OU282717/B.1.429/Epsilon	ETKCTLKSFTVEKGIYQTSNFRVQPTESIVRFPNITNLCPFGEVFNATRFASVYAWNRKR
OU235436/B.1.429/Epsilon	ETKCTLKSFTVEKGIYQTSNFRVQPTESIVRFPNITNLCPFGEVFNATRFASVYAWNRKR
MZ687447/B.1.429/Epsilon	ETKCTLKSFTVEKGIYQTSNFRVQPTESIVRFPNITNLCPFGEVFNATRFASVYAWNRKR
MZ363839/B.1.617.1/Kappa	ETKCTLKSFTVEKGIYQTSNFRVQPTESIVRFPNITNLCPFGEVFNATRFASVYAWNRKR
MZ538882/B.1.617.1/Kappa	ETKCTLKSFTVEKGIYQTSNFRVQPTESIVRFPNITNLCPFGEVFNATRFASVYAWNRKR
MZ724425/B.1.617.1/Kappa	ETKCTLKSFTVEKGIYQTSNFRVQPTESIVRFPNITNLCPFGEVFNATRFASVYAWNRKR
MZ332524/B.1.617.1/Kappa	ETKCTLKSFTVEKGIYQTSNFRVQPTESIVRFPNITNLCPFGEVFNATRFASVYAWNRKR
MZ332527/B.1.617.1/Kappa	ETKCTLKSFTVEKGIYQTSNFRVQPTESIVRFPNITNLCPFGEVFNATRFASVYAWNRKR
LC633761/B.1.617.1/Kappa	ETKCTLKSFTVEKGIYQTSNFRVQPTESIVRFPNITNLCPFGEVFNATRFASVYAWNRKR
MZ571142/B.1.617.1/Kappa	ETKCTLKSFTVEKGIYQTSNFRVQPTESIVRFPNITNLCPFGEVFNATRFASVYAWNRKR
MZ562746/B.1.617.1/Kappa	ETKCTLKSFTVEKGIYQTSNFRVQPTESIVRFPNITNLCPFGEVFNATRFASVYAWNRKR
OU053018/C.37/Lambda	ETKCTLKSFTVEKGIYQTSNFRVQPTESIVRFPNITNLCPFGEVFNATRFASVYAWNRKR
OU268528/C.37/Lambda	ETKCTLKSFTVEKGIYQTSNFRVQPTESIVRFPNITNLCPFGEVFNATRFASVYAWNRKR
OU100999/C.37/Lambda	ETKCTLKSFTVEKGIYQTSNFRVQPTESIVRFPNITNLCPFGEVFNATRFASVYAWNRKR
MZ275295/C.37/Lambda	ETKCTLKSFTVEKGIYQTSNFRVQPTESIVRFPNITNLCPFGEVFNATRFASVYAWNRKR
MZ275301/C.37/Lambda	ETKCTLKSFTVEKGIYQTSNFRVQPTESIVRFPNITNLCPFGEVFNATRFASVYAWNRKR
MZ908711/C.37/Lambda	ETKCTLKSFTVEKGIYQTSNFRVQPTESIVRFPNITNLCPFGEVFNATRFASVYAWNRKR
OU517038/C.37/Lambda	ETKCTLKSFTVEKGIYQTSNFRVQPTESIVRFPNITNLCPFGEVFNATRFASVYAWNRKR
MZ496613/C.37/Lambda	ETKCTLKSFTVEKGIYQTSNFRVQPTESIVRFPNITNLCPFGEVFNATRFASVYAWNRKR
OU466149/B.1.621/Mu	ETKCTLKSFTVEKGIYQTSNFRVQPTESIVRFPNITNLCPFGEVFNATKFASVYAWNRKR
OU571573/B.1.621/Mu	ETKCTLKSFTVEKGIYQTSNFRVQPTESIVRFPNITNLCPFGEVFNATKFASVYAWNRKR
OK025270/B.1.621/Mu	ETKCTLKSFTVEKGIYQTSNFRVQPTESIVRFPNITNLCPFGEVFNATKFASVYAWNRKR

```
                                 360       370       380       390       400       410
                                   .         .         .         .         .         .
MN908947/Wuhan-Hu-1              ISNCVADYSVLYNSASFSTFKCYGVSPTKLNDLCFTNVYADSFVIRGDEVRQIAPGQTGK
BS001137/B.1.1.7/Alpha           ISNCVADYSVLYNSASFSTFKCYGVSPTKLNDLCFTNVYADSFVIRGDEVRQIAPGQTGK
BS001138/B.1.1.7/Alpha           ISNCVADYSVLYNSASFSTFKCYGVSPTKLNDLCFTNVYADSFVIRGDEVRQIAPGQTGK
BS001139/B.1.1.7/Alpha           ISNCVADYSVLYNSASFSTFKCYGVSPTKLNDLCFTNVYADSFVIRGDEVRQIAPGQTGK
BS001140/B.1.1.7/Alpha           ISNCVADYSVLYNSASFSTFKCYGVSPTKLNDLCFTNVYADSFVIRGDEVRQIAPGQTGK
MW913362/B.1.1.7/Alpha           ISNCVADYSVLYNSASFSTFKCYGVSPTKLNDLCFTNVYADSFVIRGDEVRQIAPGQTGK
MZ266365/B.1.1.7/Alpha           ISNCVADYSVLYNSASFSTFKCYGVSPTKLNDLCFTNVYADSFVIRGDEVRQIAPGQTGK
MZ350109/B.1.1.7/Alpha           ISNCVADYSVLYNSASFSTFKCYGVSPTKLNDLCFTNVYADSFVIRGDEVRQIAPGQTGK
MZ888516/B.1.1.7/Alpha           ISNCVADYSVLYNSASFSTFKCYGVSPTKLNDLCFTNVYADSFVIRGDEVRQIAPGQTGK
OU562607/B.1.1.7/Alpha           ISNCVADYSVLYNSASFSTFKCYGVSPTKLNDLCFTNVYADSFVIRGDEVRQIAPGQTGK
OU514166/B.1.1.7/Alpha           ISNCVADYSVLYNSASFSTFKCYGVSPTKLNDLCFTNVYADSFVIRGDEVRQIAPGQTGK
OU538849/B.1.1.7/Alpha           ISNCVADYSVLYNSASFSTFKCYGVSPTKLNDLCFTNVYADSFVIRGDEVRQIAPGQTGK
MW981442/B.1.351/Beta            ISNCVADYSVLYNSASFSTFKCYGVSPTKLNDLCFTNVYADSFVIRGDEVRQIAPGQTGK
MZ298839/B.1.351/Beta            ISNCVADYSVLYNSASFSTFKCYGVSPTKLNDLCFTNVYADSFVIRGDEVRQIAPGQTGN
MZ913100/B.1.351/Beta            ISNCVADYSVLYNSASFSTFKCYGVSPTKLNDLCFTNVYADSFVIRGDEVRQIAPGQTGN
MZ879365/B.1.351/Beta            ISNCVADYSVLYNSASFSTFKCYGVSPTKLNDLCFTNVYADSFVIRGDEVRQIAPGQTGN
OU539352/B.1.351/Beta            ISNCVADYSVLYNSASFSTFKCYGVSPTKLNDLCFTNVYADSFVIRGDEVRQIAPGQTGN
MZ202314/B.1.351/Beta            ISNCVADYSVLYNSASFSTFKCYGVSPTKLNDLCFTNVYADSFVIRGDEVRQIAPGQTGN
MZ298840/B.1.351/Beta            ISNCVADYSVLYNSASFSTFKCYGVSPTKLNDLCFTNVYADSFVIRGDEVRQIAPGQTGN
LC643036/B.1.617.2/Delta         ISNCVADYSVLYNSASFSTFKCYGVSPTKLNDLCFTNVYADSFVIRGDEVRQIAPGQTGK
LC643044/B.1.617.2/Delta         ISNCVADYSVLYNSASFSTFKCYGVSPTKLNDLCFTNVYADSFVIRGDEVRQIAPGQTGK
MZ853946/B.1.617.2/Delta         ISNCVADYSVLYNSASFSTFKCYGVSPTKLNDLCFTNVYADSFVIRGDEVRQIAPGQTGK
MZ854386/B.1.617.2/Delta         ISNCVADYSVLYNSASFSTFKCYGVSPTKLNDLCFTNVYADSFVIRGDEVRQIAPGQTGK
MZ888532/B.1.617.2/Delta         ISNCVADYSVLYNSASFSTFKCYGVSPTKLNDLCFTNVYADSFVIRGDEVRQIAPGQTGK
MZ888533/B.1.617.2/Delta         ISNCVADYSVLYNSASFSTFKCYGVSPTKLNDLCFTNVYADSFVIRGDEVRQIAPGQTGK
MZ896136/B.1.617.2/Delta         ISNCVADYSVLYNSASFSTFKCYGVSPTKLNDLCFTNVYADSFVIRGDEVRQIAPGQTGK
OU539261/B.1.617.2/Delta         ISNCVADYSVLYNSASFSTFKCYGVSPTKLNDLCFTNVYADSFVIRGDEVRQIAPGQTGK
MZ854389/B.1.617.2/Delta         ISNCVADYSVLYNSASFSTFKCYGVSPTKLNDLCFTNVYADSFVIRGDEVRQIAPGQTGK
OU539784/P.1/Gamma               ISNCVADYSVLYNSASFSTFKCYGVSPTKLNDLCFTNVYADSFVIRGDEVRQIAPGQTGT
OU539830/P.1/Gamma               ISNCVADYSVLYNSASFSTFKCYGVSPTKLNDLCFTNVYADSFVIRGDEVRQIAPGQTGT
OU539894/P.1/Gamma               ISNCVADYSVLYNSASFSTFKCYGVSPTKLNDLCFTNVYADSFVIRGDEVRQIAPGQTGT
MZ477748/P.1/Gamma               ISNCVADYSVLYNSASFSTFKCYGVSPTKLNDLCFTNVYADSFVIRGDEVRQIAPGQTGT
MZ477746/P.1/Gamma               ISNCVADYSVLYNSASFSTFKCYGVSPTKLNDLCFTNVYADSFVIRGDEVRQIAPGQTGT
MW938104/P.1/Gamma               ISNCVADYSVLYNSASFSTFKCYGVSPTKLNDLCFTNVYADSFVIRGDEVRQIAPGQTGT
MZ896179/P.1/Gamma               ISNCVADYSVLYNSASFSTFKCYGVSPTKLNDLCFTNVYADSFVIRGDEVRQIAPGQTGT
MZ896359/P.1/Gamma               ISNCVADYSVLYNSASFSTFKCYGVSPTKLNDLCFTNVYADSFVIRGDEVRQIAPGQTGT
OL677199/BA.1/Omicron            ISNCVADYSVLYNLAPFFTFKCYGVSPTKLNDLCFTNVYADSFVIRGDEVRQIAPGQTGN
OL672836/BA.1/Omicron            ISNCVADYSVLYNLAPFFTFKCYGVSPTKLNDLCFTNVYADSFVIRGDEVRQIAPGQTGN
OW714547.1/BA.1/Omicron          ISNCVADYSVLYNLAPFFTFKCYGVSPTKLNDLCFTNVYADSFVIRGDEVRQIAPGQTG.
OW714198.1/BA.1/Omicron          ISNCVADYSVLYNLAPFFTFKCYGVSPTKLNDLCFTNVYADSFVIRGDEVRQIAPGQTGN
OW303180.1/BA.1/Omicron          ISNCVADYSVLYNLAPFFTFKCYGVSPTKLNDLCFTNVYADSFVIRGDEVRQIAPGQTGK
EPI_ISL_7747552/BA.2/Omicron     ISNCVADYSVLYNFAPFFAFKCYGVSPTKLNDLCFTNVYADSFVIRGNEVSQIAPGQTGN
EPI_ISL_7747545/BA.2/Omicron     ISNCVADYSVLYNFAPFFAFKCYGVSPTKLNDLCFTNVYADSFVIRGNEVSQIAPGQTGN
EPI_ISL_7747536/BA.2/Omicron     ISNCVADYSVLYNFAPFFAFKCYGVSPTKLNDLCFTNVYADSFVIRGNEVSQIAPGQTGN
EPI_ISL_7747535/BA.2/Omicron     ISNCVADYSVLYNFAPFFAFKCYGVSPTKLNDLCFTNVYADSFVIRGNEVSQIAPGQTGN
EPI_ISL_6795834/BA.2/Omicron     ISNCVADYSVLYNFAPFFAFKCYGVSPTKLNDLCFTNVYADSFVIRGNEVSQIAPGQTGN
EPI_ISL_7718520/BA.2/Omicron     ISNCVADYSVLYNFAPFFAFKCYGVSPTKLNDLCFTNVYADSFVIRGNEVSQIAPGQTGN
EPI_ISL_7580387/BA.2/Omicron     ISNCVADYSVLYNFAPFFAFKCYGVSPTKLNDLCFTNVYADSFVIRGNEVSQIAPGQTGN
EPI_ISL_7701122/BA.2/Omicron     ISNCVADYSVLYNFAPFFAFKCYGVSPTKLNDLCFTNVYADSFVIRGNEVSQIAPGQTGN
EPI_ISL_7190366/BA.2/Omicron     ISNCVADYSVLYNFAPFFAFKCYGVSPTKLNDLCFTNVYADSFVIRGNEVSQIAPGQTGN
EPI_ISL_7644798/BA.2/Omicron     ISNCVADYSVLYNFAPFFAFKCYGVSPTKLNDLCFTNVYADSFVIRGNEVSQIAPGQTGN
EPI_ISL_7740766/BA.3/Omicron     ISNCVADYSVLYNFAPFFTFKCYGVSPTKLNDLCFTNVYADSFVIRGNEVRQIAPGQTGN
EPI_ISL_7605589/BA.3/Omicron     ISNCVADYSVLYNFAPFFTFKCYGVSPTKLNDLCFTNVYADSFVIRGNEVRQIAPGQTGN
ON296657.1/BA.4/Omicron          ISNCVADYSVLYNFAPFFAFKCYGVSPTKLNDLCFTNVYADSFVIRGNEVSQIAPGQTGN
ON331287.1/BA.4/Omicron          ISNCVADYSVLYNFAPFFAFKCYGVSPTKLNDLCFTNVYADSFVIRGNEVSQIAPGQTGN
ON321245.1/BA.4/Omicron          ISNCVADYSVLYNFAPFFAFKCYGVSPTKLNDLCFTNVYADSFVIRGNEVSQIAPGQTGN
ON337003.1/BA.4/Omicron          ISNCVADYSVLYNFAPFFAFKCYGVSPTKLNDLCFTNVYADSFVIRGNEVSQIAPGQTGN
ON270681.1/BA.4/Omicron          ISNCVADYSVLYNFAPFFAFKCYGVSPTKLNDLCFTNVYADSFVIRGNEVSQIAPGQTGN
ON373214.1/BA.4/Omicron          ISNCVADYSVLYNFAPFFAFKCYGVSPTKLNDLCFTNVYADSFVIRGNEVSQIAPGQTGN
ON393428.1/BA.5/Omicron          ISNCVADYSVLYNFAPFFAFKCYGVSPTKLNDLCFTNVYADSFVIRGNEVSQIAPGQTGN
ON393156.1/BA.5/Omicron          ISNCVADYSVLYNFAPFFAFKCYGVSPTKLNDLCFTNVYADSFVIRGNEVSQIAPGQTGN
ON348053.1/BA.5/Omicron          ISNCVADYSVLYNFAPFFAFKCYGVSPTKLNDLCFTNVYADSFVIRGNEVSQIAPGQTGN
ON323731.1/BA.5/Omicron          ISNCVADYSVLYNFAPFFAFKCYGVSPTKLNDLCFTNVYADSFVIRGNEVSQIAPGQTGN
ON251467.1/BA.5/Omicron          ISNCVADYSVLYNFAPFFAFKCYGVSPTKLNDLCFTNVYADSFVIRGNEVSQIAPGQTGN
ON250431.1/BA.5/Omicron          ISNCVADYSVLYNFAPFFAFKCYGVSPTKLNDLCFTNVYADSFVIRGNEVSQIAPGQTGN
MZ911221/B.1.427/Epsilon         ISNCVADYSVLYNSASFSTFKCYGVSPTKLNDLCFTNVYADSFVIRGDEVRQIAPGQTGK
MZ375858/B.1.427/Epsilon         ISNCVADYSVLYNSASFSTFKCYGVSPTKLNDLCFTNVYADSFVIRGDEVRQIAPGQTGK
MZ375854/B.1.427/Epsilon         ISNCVADYSVLYNSASFSTFKCYGVSPTKLNDLCFTNVYADSFVIRGDEVRQIAPGQTGK
MZ943834/B.1.429/Epsilon         ISNCVADYSVLYNSASFSTFKCYGVSPTKLNDLCFTNVYADSFVIRGDEVRQIAPGQTGK
MZ277390/B.1.429/Epsilon         ISNCVADYSVLYNSASFSTFKCYGVSPTKLNDLCFTNVYADSFVIRGDEVRQIAPGQTGK
OU282717/B.1.429/Epsilon         ISNCVADYSVLYNSASFSTFKCYGVSPTKLNDLCFTNVYADSFVIRGDEVRQIAPGQTGK
OU235436/B.1.429/Epsilon         ISNCVADYSVLYNSASFSTFKCYGVSPTKLNDLCFTNVYADSFVIRGDEVRQIAPGQTGK
MZ687447/B.1.429/Epsilon         ISNCVADYSVLYNSASFSTFKCYGVSPTKLNDLCFTNVYADSFVIRGDEVRQIAPGQTGK
MZ363839/B.1.617.1/Kappa         ISNCVADYSVLYNSASFSTFKCYGVSPTKLNDLCFTNVYADSFVIRGDEVRQIAPGQTGK
MZ538882/B.1.617.1/Kappa         ISNCVADYSVLYNSASFSTFKCYGVSPTKLNDLCFTNVYADSFVIRGDEVRQIAPGQTGK
MZ724425/B.1.617.1/Kappa         ISNCVADYSVLYNSASFSTFKCYGVSPTKLNDLCFTNVYADSFVIRGDEVRQIAPGQTGK
MZ332524/B.1.617.1/Kappa         ISNCVADYSVLYNSASFSTFKCYGVSPTKLNDLCFTNVYADSFVIRGDEVRQIAPGQTGK
MZ332527/B.1.617.1/Kappa         ISNCVADYSVLYNSASFSTFKCYGVSPTKLNDLCFTNVYADSFVIRGDEVRQIAPGQTGK
LC633761/B.1.617.1/Kappa         ISNCVADYSVLYNSASFSTFKCYGVSPTKLNDLCFTNVYADSFVIRGDEVRQIAPGQTGK
MZ571142/B.1.617.1/Kappa         ISNCVADYSVLYNSASFSTFKCYGVSPTKLNDLCFTNVYADSFVIRGDEVRQIAPGQTGK
MZ562746/B.1.617.1/Kappa         ISNCVADYSVLYNSASFSTFKCYGVSPTKLNDLCFTNVYADSFVIRGDEVRQIAPGQTGK
OU053018/C.37/Lambda             ISNCVADYSVLYNSASFSTFKCYGVSPTKLNDLCFTNVYADSFVIRGDEVRQIAPGQTGK
OU268528/C.37/Lambda             ISNCVADYSVLYNSASFSTFKCYGVSPTKLNDLCFTNVYADSFVIRGDEVRQIAPGQTGK
OU100999/C.37/Lambda             ISNCVADYSVLYNSASFSTFKCYGVSPTKLNDLCFTNVYADSFVIRGDEVRQIAPGQTGK
MZ275295/C.37/Lambda             ISNCVADYSVLYNSASFSTFKCYGVSPTKLNDLCFTNVYADSFVIRGDEVRQIAPGQTGK
MZ275301/C.37/Lambda             ISNCVADYSVLYNSASFSTFKCYGVSPTKLNDLCFTNVYADSFVIRGDEVRQIAPGQTGK
MZ908711/C.37/Lambda             ISNCVADYSVLYNSASFSTFKCYGVSPTKLNDLCFTNVYADSFVIRGDEVRQIAPGQTGK
OU517038/C.37/Lambda             ISNCVADYSVLYNSASFSTFKCYGVSPTKLNDLCFTNVYADSFVIRGDEVRQIAPGQTGK
MZ496613/C.37/Lambda             ISNCVADYSVLYNSASFSTFKCYGVSPTKLNDLCFTNVYADSFVIRGDEVRQIAPGQTGK
OU466149/B.1.621/Mu              ISNCVADYSVLYNSASFSTFKCYGVSPTKLNDLCFTNVYADSFVIRGDEVRQIAPGQTGN
OU571573/B.1.621/Mu              ISNCVADYSVLYNSASFSTFKCYGVSPTKLNDLCFTNVYADSFVIRGDEVRQIAPGQTGK
OK025270/B.1.621/Mu              ISNCVADYSVLYNSASFSTFKCYGVSPTKLNDLCFTNVYADSFVIRGDEVRQIAPGQTGK
```

```
                                420       430       440       450       460       470
MN908947/Wuhan-Hu-1             IADYNYKLPDDFTGCVIAWNSNNLDSKVGGNYNYLYRLFRKSNLKPFERDISTEIYQAGS
BS001137/B.1.1.7/Alpha          IADYNYKLPDDFTGCVIAWNSNNLDSKVGGNYNYLYRLFRKSNLKPFERDISTEIYQAGS
BS001138/B.1.1.7/Alpha          IADYNYKLPDDFTGCVIAWNSNNLDSKVGGNYNYLYRLFRKSNLKPFERDISTEIYQAGS
BS001139/B.1.1.7/Alpha          IADYNYKLPDDFTGCVIAWNSNNLDSKVGGNYNYLYRLFRKSNLKPFERDISTEIYQAGS
BS001140/B.1.1.7/Alpha          IADYNYKLPDDFTGCVIAWNSNNLDSKVGGNYNYLYRLFRKSNLKPFERDISTEIYQAGS
MW913362/B.1.1.7/Alpha          IADYNYKLPDDFTGCVIAWNSNNLDSKVGGNYNYLYRLFRKSNLKPFERDISTEIYQAGS
MZ266365/B.1.1.7/Alpha          IADYNYKLPDDFTGCVIAWNSNNLDSKVGGNYNYLYRLFRKSNLKPFERDISTEIYQAGS
MZ350109/B.1.1.7/Alpha          IADYNYKLPDDFTGCVIAWNSNNLDSKVGGNYNYLYRLFRKSNLKPFERDISTEIYQAGS
MZ888516/B.1.1.7/Alpha          IADYNYKLPDDFTGCVIAWNSNNLDSKVGGNYNYLYRLFRKSNLKPFERDISTEIYQAGS
OU562607/B.1.1.7/Alpha          IADYNYKLPDDFTGCVIAWNSNNLDSKVGGNYNYLYRLFRKSNLKPFERDISTEIYQAGS
OU514166/B.1.1.7/Alpha          IADYNYKLPDDFTGCVIAWNSNNLDSKVGGNYNYLYRLFRKSNLKPFERDISTEIYQAGS
OU538849/B.1.1.7/Alpha          IADYNYKLPDDFTGCVIAWNSNNLDSKVGGNYNYLYRLFRKSNLKPFERDISTEIYQAGS
MW981442/B.1.351/Beta           IADYNYKLPDDFTGCVIAWNSNNLDSKVGGNYNYLYRLFRKSNLKPFERDISTEIYQAGS
MZ298839/B.1.351/Beta           IADYNYKLPDDFTGCVIAWNSNNLDSKVGGNYNYLYRLFRKSNLKPFERDISTEIYQAGS
MZ913100/B.1.351/Beta           IADYNYKLPDDFTGCVIAWNSNNLDSKVGGNYNYLYRLFRKSNLKPFERDISTEIYQAGS
MZ879365/B.1.351/Beta           IADYNYKLPDDFTGCVIAWNSNNLDSKVGGNYNYLYRLFRKSNLKPFERDISTEIYQAGS
OU539352/B.1.351/Beta           IADYNYKLPDDFTGCVIAWNSNNLDSKVGGNYNYLYRLFRKSNLKPFERDISTEIYQAGS
MZ202314/B.1.351/Beta           IADYNYKLPDDFTGCVIAWNSNNLDSKVGGNYNYLYRLFRKSNLKPFERDISTEIYQAGS
MZ298840/B.1.351/Beta           IADYNYKLPDDFTGCVIAWNSNNLDSKVGGNYNYLYRLFRKSNLKPFERDISTEIYQAGS
LC643036/B.1.617.2/Delta        IADYNYKLPDDFTGCVIAWNSNNLDSKVGGNYNYRYRLFRKSNLKPFERDISTEIYQAGS
LC643044/B.1.617.2/Delta        IADYNYKLPDDFTGCVIAWNSNNLDSKVGGNYNYRYRLFRKSNLKPFERDISTEIYQAGS
MZ853946/B.1.617.2/Delta        IADYNYKLPDDFTGCVIAWNSNNLDSKVGGNYNYRYRLFRKSNLKPFERDISTEIYQAGS
MZ854386/B.1.617.2/Delta        IADYNYKLPDDFTGCVIAWNSNNLDSKVVGNYNYRYRLFRKSNLKPFERDISTEIYQAGS
MZ888532/B.1.617.2/Delta        IADYNYKLPDDFTGCVIAWNSNNLDSKVGGNYNYRYRLFRKSNLKPFERDISTEIYQAGS
MZ888533/B.1.617.2/Delta        IADYNYKLPDDFTGCVIAWNSNNLDSKVGGNYNYRYRLFRKSNLKPFERDISTEIYQAGS
MZ896136/B.1.617.2/Delta        IADYNYKLPDDFTGCVIAWNSNNLDSKVGGNYNYRYRLFRKSNLKPFERDISTEIYQAGS
OU539261/B.1.617.2/Delta        IADYNYKLPDDFTGCVIAWNSNNLDSKVGGNYNYRYRLFRKSNLKPFERDISTEIYQAGS
MZ854389/B.1.617.2/Delta        IADYNYKLPDDFTGCVIAWNSNNLDSKVGGNYNYRYRLFRKSNLKPFERDISTEIYQAGS
OU539784/P.1/Gamma              IADYNYKLPDDFTGCVIAWNSNNLDSKVGGNYNYLYRLFRKSNLKPFERDISTEIYQAGS
OU539830/P.1/Gamma              IADYNYKLPDDFTGCVIAWNSNNLDSKVGGNYNYLYRLFRKSNLKPFERDISTEIYQAGS
OU539894/P.1/Gamma              IADYNYKLPDDFTGCVIAWNSNNLDSKVGGNYNYLYRLFRKSNLKPFERDISTEIYQAGS
MZ477748/P.1/Gamma              IADYNYKLPDDFTGCVIAWNSNNLDSKVGGNYNYLYRLFRKSNLKPFERDISTEIYQAGS
MZ477746/P.1/Gamma              IADYNYKLPDDFTGCVIAWNSNNLDSKVGGNYNYLYRLFRKSNLKPFERDISTEIYQAGS
MW938104/P.1/Gamma              IADYNYKLPDDFTGCVIAWNSNNLDSKVGGNYNYLYRLFRKSNLKPFERDISTEIYQAGS
MZ896179/P.1/Gamma              IADYNYKLPNDFTGCVIAWNSNNLDSKVGGNYNYLYRLFRKSNLKPFERDISTEIYQAGS
MZ896359/P.1/Gamma              IADYNYKLPDDFTGCVIAWNSNNLDSKVGGNYNYLYRLFRKSNLKPFERDISTEIYQAGS
OL677199/BA.1/Omicron           IADYNYKLPDDFTGCVIAWNSNKLDSKVSGNYNYLYRLFRKSNLKPFERDISTEIYQAGN
OL672836/BA.1/Omicron           IADYNYKLPDDFTGCVIAWNSNKLDSKVSGNYNYLYRLFRKSNLKPFERDISTEIYQAGN
OW714547.1/BA.1/Omicron         IADYNYKLPDDFTGCVIAWNSNKLDSKVSGNYNYLYRLFRKSNLKPFERDISTEIYQAGN
OW714198.1/BA.1/Omicron         IADYNYKLPDDFTGCVIAWNSNKLDSKVSGNYNYLYRLFRKSNLKPFERDISTEIYQAGN
OW303180.1/BA.1/Omicron         IADYNYKLPDDFTGCVIAWNSNKLDSKVSGNYNYLYRLFRKSNLKPFERDISTEIYQAGN
EPI_ISL_7747552/BA.2/Omicron    IADYNYKLPDDFTGCVIAWNSNKLDSKVGGNYNYLYRLFRKSNLKPFERDISTEIYQAGN
EPI_ISL_7747545/BA.2/Omicron    IADYNYKLPDDFTGCVIAWNSNKLDSKVGGNYNYLYRLFRKSNLKPFERDISTEIYQAGN
EPI_ISL_7747536/BA.2/Omicron    IADYNYKLPDDFTGCVIAWNSNKLDSKVGGNYNYLYRLFRKSNLKPFERDISTEIYQAGN
EPI_ISL_7747535/BA.2/Omicron    IADYNYKLPDDFTGCVIAWNSNKLDSKVGGNYNYLYRLFRKSNLKPFERDISTEIYQAGN
EPI_ISL_6795834/BA.2/Omicron    IADYNYKLPDDFTGCVIAWNSNKLDSKVGGNYNYLYRLFRKSNLKPFERDISTEIYQAGN
EPI_ISL_7718520/BA.2/Omicron    IADYNYKLPDDFTGCVIAWNSNKLDSKVGGNYNYLYRLFRKSNLKPFERDISTEIYQAGN
EPI_ISL_7580387/BA.2/Omicron    IADYNYKLPDDFTGCVIAWNSNKLDSKVGGNYNYLYRLFRKSNLKPFERDISTEIYQAGN
EPI_ISL_7701122/BA.2/Omicron    IADYNYKLPDDFTGCVIAWNSNKLDSKVGGNYNYLYRLFRKSNLKPFERDISTEIYQAGN
EPI_ISL_7190366/BA.2/Omicron    IADYNYKLPDDFTGCVIAWNSNKLDSKVGGNYNYLYRLFRKSNLKPFERDISTEIYQAGN
EPI_ISL_7644798/BA.2/Omicron    IADYNYKLPDDFTGCVIAWNSNKLDSKVGGNYNYLYRLFRKSNLKPFERDISTEIYQAGN
EPI_ISL_7740766/BA.3/Omicron    IADYNYKLPDDFTGCVIAWNSNKLDSKVSGNYNYLYRLFRKSNLKPFERDISTEIYQAGN
EPI_ISL_7605589/BA.3/Omicron    IADYNYKLPDDFTGCVIAWNSNKLDSKVSGNYNYLYRLFRKSNLKPFERDISTEIYQAGN
ON296657.1/BA.4/Omicron         IADYNYKLPDDFTGCVIAWNSNKLDSKVGGNYNYRYRLFRKSNLKPFERDISTEIYQAGN
ON331287.1/BA.4/Omicron         IADYNYKLPDDFTGCVIAWNSNKLDSKVGGNYNYRYRLFRKSNLKPFERDISTEIYQAGN
ON321245.1/BA.4/Omicron         IADYNYKLPDDFTGCVIAWNSNKLDSKVGGNYNYRYRLFRKSNLKPFERDISTEIYQAGN
ON337003.1/BA.4/Omicron         IADYNYKLPDDFTGCVIAWNSNKLDSKVGGNYNYRYRLFRKSNLKPFERDISTEIYQAGN
ON270681.1/BA.4/Omicron         IADYNYKLPDDFTGCVIAWNSNKLDSKVGGNYNYRYRLFRKSNLKPFERDISTEIYQAGN
ON373214.1/BA.4/Omicron         IADYNYKLPDDFTGCVIAWNSNKLDSKVGGNYNYRYRLFRKSNLKPFERDISTEIYQAGN
ON393428.1/BA.5/Omicron         IADYNYKLPDDFTGCVIAWNSNKLDSKVGGNYNYRYRLFRKSNLKPFERDISTEIYQAGN
ON393156.1/BA.5/Omicron         IADYNYKLPDDFTGCVIAWNSNKLDSKVGGNYNYRYRLFRKSNLKPFERDISTEIYQAGN
ON348053.1/BA.5/Omicron         IADYNYKLPDDFTGCVIAWNSNKLDSKVGGNYNYRYRLFRKSNLKPFERDISTEIYQAGN
ON323731.1/BA.5/Omicron         IADYNYKLPDDFTGCVIAWNSNKLDSKVGGNYNYRYRLFRKSNLKPFERDISTEIYQAGN
ON251467.1/BA.5/Omicron         IADYNYKLPDDFTGCVIAWNSNKLDSKVGGNYNYRYRLFRKSNLKPFERDISTEIYQAGN
ON250431.1/BA.5/Omicron         IADYNYKLPDDFTGCVIAWNSNKLDSKVGGNYNYRYRLFRKSNLKPFERDISTEIYQAGN
MZ911221/B.1.427/Epsilon        IADYNYKLPDDFTGCVIAWNSNNLDSKVGGNYNYRYRLFRKSNLKPFERDISTEIYQAGS
MZ375858/B.1.427/Epsilon        IADYNYKLPDDFTGCVIAWNSNNLDSKVGGNYNYRYRLFRKSNLKPFERDISTEIYQAGS
MZ375854/B.1.427/Epsilon        IADYNYKLPDDFTGCVIAWNSNNLDSKVGGNYNYRYRLFRKSNLKPFERDISTEIYQAGS
MZ943834/B.1.429/Epsilon        IADYNYKLPDDFTGCVIAWNSNNLDSKVGGNYNYRYRLFRKSNLKPFERDISTEIYQAGS
MZ277390/B.1.429/Epsilon        IADYNYKLPDDFTGCVIAWNSNNLDSKVGGNYNYRYRLFRKSNLKPFERDISTEIYQAGS
OU282717/B.1.429/Epsilon        IADYNYKLPDDFTGCVIAWNSNNLDSKVGGNYNYRYRLFRKSNLKPFERDISTEIYQAGS
OU235436/B.1.429/Epsilon        IADYNYKLPDDFTGCVIAWNSNNLDSKVGGNYNYRYRLFRKSNLKPFERDISTEIYQAGS
MZ687447/B.1.429/Epsilon        IADYNYKLPDDFTGCVIAWNSNNLDSKVGGNYNYRYRLFRKSNLKPFERDISTEIYQAGS
MZ363839/B.1.617.1/Kappa        IADYNYKLPDDFTGCVIAWNSNNLDSKVGGNYNYRYRLFRKSNLKPFERDISTEIYQAGS
MZ538882/B.1.617.1/Kappa        IADYNYKLPDDFTGCVIAWNSNNLDSKVGGNYNYRYRLFRKSNLKPFERDISTEIYQAGS
MZ724425/B.1.617.1/Kappa        IADYNYKLPDDFTGCVIAWNSNNLDSKVGGNYNYRYRLFRKSNLKPFERDISTEIYQAGS
MZ332524/B.1.617.1/Kappa        IADYNYKLPDDFTGCVIAWNSNNLDSKVGGNYNYRYRLFRKSNLKPFERDISTEIYQAGS
MZ332527/B.1.617.1/Kappa        IADYNYKLPDDFTGCVIAWNSNNLDSKVGGNYNYRYRLFRKSNLKPFERDISTEIYQAGS
LC633761/B.1.617.1/Kappa        IADYNYKLPDDFTGCVIAWNSNNLDSKVGGNYNYRYRLFRKSNLKPFERDISTEIYQAGS
MZ571142/B.1.617.1/Kappa        IADYNYKLPDDFTGCVIAWNSNNLDSKVGGNYNYRYRLFRKSNLKPFERDISTEIYQAGS
MZ562746/B.1.617.1/Kappa        IADYNYKLPDDFTGCVIAWNSNNLDSKVGGNYNYRYRLFRKSNLKPFERDISTEIYQAGS
OU053018/C.37/Lambda            IADYNYKLPDDFTGCVIAWNSNNLDSKVGGNYNYQYRLFRKSNLKPFERDISTEIYQAGS
OU268528/C.37/Lambda            IADYNYKLPDDFTGCVIAWNSNNLDSKVGGNYNYQYRLFRKSNLKPFERDISTEIYQAGS
OU100999/C.37/Lambda            IADYNYKLPDDFTGCVIAWNSNNLDSKVGGNYNYQYRLFRKSNLKPFERDISTEIYQAGS
MZ275295/C.37/Lambda            IADYNYKLPDDFTGCVIAWNSNNLDSKVGGNYNYQYRLFRKSNLKPFERDISTEIYQAGS
MZ275301/C.37/Lambda            IADYNYKLPDDFTGCVIAWNSNNLDSKVGGNYNYQYRLFRKSNLKPFERDISTEIYQAGS
MZ908711/C.37/Lambda            IADYNYKLPDDFTGCVIAWNSNNLDSKVGGNYNYQYRLFRKSNLKPFERDISTEIYQAGS
OU517038/C.37/Lambda            IADYNYKLPDDFTGCVIAWNSNNLDSKVGGNYNYQYRLFRKSNLKPFERDISTEIYQAGS
MZ496613/C.37/Lambda            IADYNYKLPDDFTGCVIAWNSNNLDSKVGGNYNYQYRLFRKSNLKPFERDISTEIYQAGS
OU466149/B.1.621/Mu             IADYNYKLPDDFTGCVIAWNSNNLDSKVGGNYNYLYRLFRKSNLKPFERDISTEIYQAGS
OU571573/B.1.621/Mu             IADYNYKLPDDFTGCVIAWNSNNLDSKVGGNYNYLYRLFRKSNLKPFERDISTEIYQAGS
OK025270/B.1.621/Mu             IADYNYKLPDDFTGCVIAWNSNNLDSKVGGNYNYLYRLFRKSNLKPFERDISTEIYQAGS
```

```
                                    480       490       500       510       520       530
MN908947/Wuhan-Hu-1                TPCNGVEGFNCYFPLQSYGFQPTNGVGYQPYRVVVLSFELLHAPATVCGPKKSTNLVKNK
BS001137/B.1.1.7/Alpha             TPCNGVEGFNCYFPLQSYGFQPTYGVGYQPYRVVVLSFELLHAPATVCGPKKSTNLVKNK
BS001138/B.1.1.7/Alpha             TPCNGVEGFNCYFPLQSYGFQPTYGVGYQPYRVVVLSFELLHAPATVCGPKKSTNLVKNK
BS001139/B.1.1.7/Alpha             TPCNGVEGFNCYFPLQSYGFQPTYGVGYQPYRVVVLSFELLHAPATVCGPKKSTNLVKNK
BS001140/B.1.1.7/Alpha             TPCNGVEGFNCYFPLQSYGFQPTYGVGYQPYRVVVLSFELLHAPATVCGPKKSTNLVKNK
MW913362/B.1.1.7/Alpha             TPCNGVEGFNCYFPLQSYGFQPTYGVGYQPYRVVVLSFELLHAPATVCGPKKSTNLVKNK
MZ266365/B.1.1.7/Alpha             TPCNGVEGFNCYFPLQSYGFQPTYGVGYQPYRVVVLSFELLHAPATVCGPKKSTNLVKNK
MZ350109/B.1.1.7/Alpha             TPCNGVEGFNCYFPLQSYGFQPTYGVGYQPYRVVVLSFELLHAPATVCGPKKSTNLVKNK
MZ888516/B.1.1.7/Alpha             TPCNGVEGFNCYFPLQSYGFQPTYGVGYQPYRVVVLSFELLHAPATVCGPKKSTNLVKNK
OU562607/B.1.1.7/Alpha             TPCNGVEGFNCYFPLQSYGFQPTYGVGYQPYRVVVLSFELLHAPATVCGPKKSTNLVKNK
OU514166/B.1.1.7/Alpha             TPCNGVEGFNCYFPLQSYGFQPTYGVGYQPYRVVVLSFELLHAPATVCGPKKSTNLVKNK
OU538849/B.1.1.7/Alpha             TPCNGVEGFNCYFPLQSYGFQPTYGVGYQPYRVVVLSFELLHAPATVCGPKKSTNLVKNK
MW981442/B.1.351/Beta              TPCNGVEGFNCYFPLQSYGFQPTYGVGYQPYRVVVLSFELLHAPATVCGPKKSTNLVKNK
MZ298839/B.1.351/Beta              TPCNGVKGFNCYFPLQSYGFQPTYGVGYQPYRVVVLSFELLHAPATVCGPKKSTNLVKNK
MZ913100/B.1.351/Beta              TPCNGVKGFNCYFPLQSYGFQPTYGVGYQPYRVVVLSFELLHAPATVCGPKKSTNLVKNK
MZ879365/B.1.351/Beta              TPCNGVKGFNCYFPLQSYGFQPTYGVGYQPYRVVVLSFELLHAPATVCGPKKSTNLVKNK
OU539352/B.1.351/Beta              TPCNGVKGFNCYFPLQSYGFQPTYGVGYQPYRVVVLSFELLHAPATVCGPKKSTNLVKNK
MZ202314/B.1.351/Beta              TPCNGVKGFNCYFPLQSYGFQPTYGVGYQPYRVVVLSFELLHAPATVCGPKKSTNLVKNK
MZ298840/B.1.351/Beta              TPCNGVKGFNCYFPLQSYGFQPTYGVGYQPYRVVVLSFELLHAPATVCGPKKSTNLVKNK
LC643036/B.1.617.2/Delta           KPCNGVEGFNCYFPLQSYGFQPTNGVGYQPYRVVVLSFELLHAPATVCGPKKSTNLVKNK
LC643044/B.1.617.2/Delta           KPCNGVEGFNCYFPLQSYGFQPTNGVGYQPYRVVVLSFELLHAPATVCGPKKSTNLVKNK
MZ853946/B.1.617.2/Delta           KPCNGVEGFNCYFPLQSYGFQPTNGVGYQPYRVVVLSFELLHAPATVCGPKKSTNLVKNK
MZ854386/B.1.617.2/Delta           KPCNGVEGFNCYFPLQSYGFQPTNGVGYQPYRVVVLSFELLHAPATVCGPKKSTNLVKNK
MZ888532/B.1.617.2/Delta           KPCNGVEGFNCYFPLQSYGFQPTNGVGYQPYRVVVLSFELLHAPATVCGPKKSTNLVKNK
MZ888533/B.1.617.2/Delta           KPCNGVEGFNCYFPLQSYGFQPTNGVGYQPYRVVVLSFELLHAPATVCGPKKSTNLVKNK
MZ896136/B.1.617.2/Delta           KPCNGVEGFNCYFPLQSYGFQPTNGVGYQPYRVVVLSFELLHAPATVCGPKKSTNLVKNK
OU539261/B.1.617.2/Delta           KPCNGVEGFNCYFPLQSYGFQPTNGVGYQPYRVVVLSFELLHAPATVCGPKKSTNLVKNK
MZ854389/B.1.617.2/Delta           KPCNGVEGFNCYFPLQSYGFQPTNGVGYQPYRVVVLSFELLHAPATVCGPKKSTNLVKNK
OU539784/P.1/Gamma                 TPCNGVKGFNCYFPLQSYGFQPTYGVGYQPYRVVVLSFELLHAPATVCGPKKSTNLVKNK
OU539830/P.1/Gamma                 TPCNGVKGFNCYFPLQSYGFQPTYGVGYQPYRVVVLSFELLHAPATVCGPKKSTNLVKNK
OU539894/P.1/Gamma                 TPCNGVKGFNCYFPLQSYGFQPTYGVGYQPYRVVVLSFELLHAPATVCGPKKSTNLVKNK
MZ477748/P.1/Gamma                 TPCNGVKGFNCYFPLQSYGFQPTYGVGYQPYRVVVLSFELLHAPATVCGPKKSTNLVKNK
MZ477746/P.1/Gamma                 TPCNGVKGFNCYFPLQSYGFQPTYGVGYQPYRVVVLSFELLHAPATVCGPKKSTNLVKNK
MW938104/P.1/Gamma                 TPCNGVKGFNCYFPLQSYGFQPTYGVGYQPYRVVVLSFELLHAPATVCGPKKSTNLVKNK
MZ896179/P.1/Gamma                 TPCNGVKGFNCYFPLQSYGFQPTYGVGYQPYRVVVLSFELLHAPATVCGPKKSTNLVKNK
MZ896359/P.1/Gamma                 TPCNGVKGFNCYFPLQSYGFQPTYGVGYQPYRVVVLSFELLHAPATVCGPKKSTNLVKNK
OL677199/BA.1/Omicron              KPCNGVAGFNCYFPLRSYSFRPTYGVGHQPYRVVVLSFELLHAPATVCGPKKSTNLVKNK
OL672836/BA.1/Omicron              KPCNGVAGFNCYFPLRSYSFRPTYGVGHQPYRVVVLSFELLHAPATVCGPKKSTNLVKNK
OW714547.1/BA.1/Omicron            KPCNGVAGFNCYFPLRSYSFRPTYGVGHQPYRVVVLSFELLHAPATVCGPKKSTNLVKNK
OW714198.1/BA.1/Omicron            KPCNGVAGFNCYFPLRSYSFRPTYGVGHQPYRVVVLSFELLHAPATVCGPKKSTNLVKNK
OW303180.1/BA.1/Omicron            KPCNGVAGFNCYFPLRSYSFRPTYGVGHQPYRVVVLSFELLHAPATVCGPKKSTNLVKNK
EPI_ISL_7747552/BA.2/Omicron       KPCNGVAGFNCYFPLRSYGFRPTYGVGHQPYRVVVLSFELLHAPATVCGPKKSTNLVKNK
EPI_ISL_7747545/BA.2/Omicron       KPCNGVAGFNCYFPLRSYGFRPTYGVGHQPYRVVVLSFELLHAPATVCGPKKSTNLVKNK
EPI_ISL_7747536/BA.2/Omicron       KPCNGVAGFNCYFPLRSYGFRPTYGVGHQPYRVVVLSFELLHAPATVCGPKKSTNLVKNK
EPI_ISL_7747535/BA.2/Omicron       KPCNGVAGFNCYFPLRSYGFRPTYGVGHQPYRVVVLSFELLHAPATVCGPKKSTNLVKNK
EPI_ISL_6795834/BA.2/Omicron       KPCNGVAGFNCYFPLRSYGFRPTYGVGHQPYRVVVLSFELLHAPATVCGPKKSTNLVKNK
EPI_ISL_7718520/BA.2/Omicron       KPCNGVAGFNCYFPLRSYGFRPTYGVGHQPYRVVVLSFELLHAPATVCGPKKSTNLVKNK
EPI_ISL_7580387/BA.2/Omicron       KPCNGVAGFNCYFPLRSYGFRPTYGVGHQPYRVVVLSFELLHAPATVCGPKKSTNLVKNK
EPI_ISL_7701122/BA.2/Omicron       KPCNGVAGFNCYFPLRSYGFRPTYGVGYQPYRVVVLSFELLHAPATVCGPKKSTNLVKNK
EPI_ISL_7190366/BA.2/Omicron       KPCNGVAGFNCYFPLRSYGFRPTYGVGHQPYRVVVLSFELLHAPATVCGPKKSTNLVKNK
EPI_ISL_7644798/BA.2/Omicron       KPCNGVAGFNCYFPLRSYGFRPTYGVGHQPYRVVVLSFELLHAPATVCGPKKSTNLVKNK
EPI_ISL_7740766/BA.3/Omicron       KPCNGVAGFNCYFPLRSYGFRPTYGVGHQPYRVVVLSFELLHAPATVCGPKKSTNLVKNK
EPI_ISL_7605589/BA.3/Omicron       KPCNGVAGFNCYFPLRSYGFRPTYGVGHQPYRVVVLSFELLHAPATVCGPKKSTNLVKNK
ON296657.1/BA.4/Omicron            KPCNGVAGVNCYFPLQSYGFRPTYGVGHQPYRVVVLSFELLHAPATVCGPKKSTNLVKNK
ON331287.1/BA.4/Omicron            KPCNGVAGVNCYFPLQSYGFRPTYGVGHQPYRVVVLSFELLHAPATVCGPKKSTNLVKNK
ON321245.1/BA.4/Omicron            KPCNGVAGVNCYFPLQSYGFRPTYGVGHQPYRVVVLSFELLHAPATVCGPKKSTNLVKNK
ON337003.1/BA.4/Omicron            KPCNGVAGVNCYFPLQSYGFRPTYGVGHQPYRVVVLSFELLHAPATVCGPKKSTNLVKNK
ON270681.1/BA.4/Omicron            KPCNGVAGVNCYFPLQSYGFRPTYGVGHQPYRVVVLSFELLHAPATVCGPKKSTNLVKNK
ON373214.1/BA.4/Omicron            KPCNGVAGVNCYFPLQSYGFRPTYGVGHQPYRVVVLSFELLHAPATVCGPKKSTNLVKNK
ON393428.1/BA.5/Omicron            KPCNGVAGVNCYFPLQSYGFRPTYGVGHQPYRVVVLSFELLHAPATVCGPKKSTNLVKNK
ON393156.1/BA.5/Omicron            KPCNGVAGVNCYFPLQSYGFRPTYGVGHQPYRVVVLSFELLHAPATVCGPKKSTNLVKNK
ON348053.1/BA.5/Omicron            KPCNGVAGVNCYFPLQSYGFRPTYGVGHQPYRVVVLSFELLHAPATVCGPKKSTNLVKNK
ON323731.1/BA.5/Omicron            KPCNGVAGVNCYFPLQSYGFRPTYGVGHQPYRVVVLSFELLHAPATVCGPKKSTNLVKNK
ON251467.1/BA.5/Omicron            KPCNGVAGVNCYFPLQSYGFRPTYGVGHQPYRVVVLSFELLHAPATVCGPKKSTNLVKNK
ON250431.1/BA.5/Omicron            KPCNGVAGVNCYFPLQSYGFRPTYGVGHQPYRVVVLSFELLHAPATVCGPKKSTNLVKNK
MZ911221/B.1.427/Epsilon           TPCNGVEGFNCYFPLQSYGFQPTNGVGYQPYRVVVLSFELLHAPATVCGPKKSTNLVKNK
MZ375858/B.1.427/Epsilon           TPCNGVEGFNCYFPLQSYGFQPTNGVGYQPYRVVVLSFELLHAPATVCGPKKSTNLVKNK
MZ375854/B.1.427/Epsilon           TPCNGVEGFNCYFPLQSYGFQPTNGVGYQPYRVVVLSFELLHAPATVCGPKKSTNLVKNK
MZ943834/B.1.429/Epsilon           TPCNGVEGFNCYFPLQSYGFQPTNGVGYQPYRVVVLSFELLHAPATVCGPKKSTNLVKNK
MZ277390/B.1.429/Epsilon           TPCNGVEGFNCYFPLQSYGFQPTNGVGYQPYRVVVLSFELLHAPATVCGPKKSTNLVKNK
OU282717/B.1.429/Epsilon           TPCNGVEGFNCYFPLQSYGFQPTNGVGYQPYRVVVLSFELLHAPATVCGPKKSTNLVKNK
OU235436/B.1.429/Epsilon           TPCNGVEGFNCYFPLQSYGFQPTNGVGYQPYRVVVLSFELLHAPATVCGPKKSTNLVKNK
MZ687447/B.1.429/Epsilon           TPCNGVEGFNCYFPLQSYGFQPTNGVGYQPYRVVVLSFELLHAPATVCGPKKSTNLVKNK
MZ363839/B.1.617.1/Kappa           TPCNGVQGFNCYFPLQSYGFQPTNGVGYQPYRVVVLSFELLHAPATVCGPKKSTNLVKNK
MZ538882/B.1.617.1/Kappa           TPCNGVQGFNCYFPLQSYGFQPTNGVGYQPYRVVVLSFELLHAPATVCGPKKSTNLVKNK
MZ724425/B.1.617.1/Kappa           TPCNGVQGFNCYFPLQSYGFQPTNGVGYQPYRVVVLSFELLHAPATVCGPKKSTNLVKNK
MZ332524/B.1.617.1/Kappa           TPCNGVQGFNCYFPLQSYGFQPTNGVGYQPYRVVVLSFELLHAPATVCGPKKSTNLVKNK
MZ332527/B.1.617.1/Kappa           TPCNGVQGFNCYFPLQSYGFQPTNGVGYQPYRVVVLSFELLHAPATVCGPKKSTNLVKNK
LC633761/B.1.617.1/Kappa           TPCNGVQGFNCYFPLQSYGFQPTNGVGYQPYRVVVLSFELLHAPATVCGPKKSTNLVKNK
MZ571142/B.1.617.1/Kappa           TPCNGVQGFNCYFPLQSYGFQPTNGVGYQPYRVVVLSFELLHAPATVCGPKKSTNLVKNK
MZ562746/B.1.617.1/Kappa           TPCNGVQGFNCYFPLQSYGFQPTNGVGYQPYRVVVLSFELLHAPVTVCGPKKSTNLVKNK
OU053018/C.37/Lambda               TPCNGVEGFNCYSPLQSYGFQPTNGVGYQPYRVVVLSFELLHAPATVCGPKKSTNLVKNK
OU268528/C.37/Lambda               TPCNGVEGFNCYSPLQSYGFQPTNGVGYQPYRVVVLSFELLHAPATVCGPKKSTNLVKNK
OU100999/C.37/Lambda               TPCNGVEGFNCYSPLQSYGFQPTNGVGYQPYRVVVLSFELLHAPATVCGPKKSTNLVKNK
MZ275295/C.37/Lambda               TPCNGVEGFNCYSPLQSYGFQPTNGVGYQPYRVVVLSFELLHAPATVCGPKKSTNLVKNK
MZ275301/C.37/Lambda               TPCNGVEGFNCYSPLQSYGFQPTNGVGYQPYRVVVLSFELLHAPATVCGPKKSTNLVKNK
MZ908711/C.37/Lambda               TPCNGVEGFNCYSPLQSYGFQPTNGVGYQPYRVVVLSFELLHAPATVCGPKKSTNLVKNK
OU517038/C.37/Lambda               TPCNGVEGFNCYSPLQSYGFQPTNGVGYQPYRVVVLSFELLHAPATVCGPKKSTNLVKNK
MZ496613/C.37/Lambda               TPCNGVEGFNCYSPLQSYGFQPTNGVGYQPYRVVVLSFELLHAPATVCGPKKSTNLVKNK
OU466149/B.1.621/Mu                TPCNGVKGFNCYFPLQSYGFQPTYGVGYQPYRVVVLSFELLHAPATVCGPKKSTNLVKNK
OU571573/B.1.621/Mu                TPCNGVKGFNCYFPLQSYGFQPTYGVGYQPYRVVVLSFELLHAPATVCGPKKSTNLVKNK
OK025270/B.1.621/Mu                TPCNGVKGFNCYFPLQSYGFQPTYGVGYQPYRVVVLSFELLHAPATVCGPKKSTNLVKNK
```

```
                                     540       550       560       570       580       590
                                       .         .         .         .         .         .
MN908947/Wuhan-Hu-1                  CVNFNFNGLTGTGVLTESNKKFLPFQQFGRDIADTTDAVRDPQTLEILDITPCSFGGVSV
BS001137/B.1.1.7/Alpha               CVNFNFNGLTGTGVLTESNKKFLPFQQFGRDIDDTTDAVRDPQTLEILDITPCSFGGVSV
BS001138/B.1.1.7/Alpha               CVNFNFNGLTGTGVLTESNKKFLPFQQFGRDIDDTTDAVRDPQTLEILDITPCSFGGVSV
BS001139/B.1.1.7/Alpha               CVNFNFNGLTGTGVLTESNKKFLPFQQFGRDIDDTTDAVRDPQTLEILDITPCSFGGVSV
BS001140/B.1.1.7/Alpha               CVNFNFNGLTGTGVLTESNKKFLPFQQFGRDIDDTTDAVRDPQTLEILDITPCSFGGVSV
MW913362/B.1.1.7/Alpha               CVNFNFNGLTGTGVLTESNKKFLPFQQFGRDIDDTTDAVRDPQTLEILDITPCSFGGVSV
MZ266365/B.1.1.7/Alpha               CVNFNFNGLTGTGVLTESNKKFLPFQQFGRDIDDTTDAVRDPQTLEILDITPCSFGGVSV
MZ350109/B.1.1.7/Alpha               CVNFNFNGLTGTGVLTESNKKFLPFQQFGRDIDDTTDAVRDPQTLEILDITPCSFGGVSV
MZ888516/B.1.1.7/Alpha               CVNFNFNGLTGTGVLTESNKKFLPFQQFGRDIDDTTDAVRDPQTLEILDITPCSFGGVSV
OU562607/B.1.1.7/Alpha               CVNFNFNGLTGTGVLTESNKKFLPFQQFGRDIDDTTDAVRDPQTLEILDITPCSFGGVSV
OU514166/B.1.1.7/Alpha               CVNFNFNGLTGTGVLTESNKKFLPFQQFGRDIDDTTDAVRDPQTLEILDITPCSFGGVSV
OU538849/B.1.1.7/Alpha               CVNFNFNGLTGTGVLTESNKKFLPFQQFGRDIDDTTDAVRDPQTLEILDITPCSFGGVSV
MW981442/B.1.351/Beta                CVNFNFNGLTGTGVLTESNKKFLPFQQFGRDIADTTDAVRDPQTLEILDITPCSFGGVSV
MZ298839/B.1.351/Beta                CVNFNFNGLTGTGVLTESNKKFLPFQQFGRDIADTTDAVRDPQTLEILDITPCSFGGVSV
MZ913100/B.1.351/Beta                CVNFNFNGLTGTGVLTESNKKFLPFQQFGRDIADTTDAVRDPQTLEILDITPCSFGGVSV
MZ879365/B.1.351/Beta                CVNFNFNGLTGTGVLTESNKKFLPFQQFGRDIADTTDAVRDPQTLEILDITPCSFGGVSV
OU539352/B.1.351/Beta                CVNFNFNGLTGTGVLTESNKKFLPFQQFGRDIADTTDAVRDPQTLEILDITPCSFGGVSV
MZ202314/B.1.351/Beta                CVNFNFNGLTGTGVLTESNKKFLPFQQFGRDIADTTDAVRDPQTLEILDITPCSFGGVSV
MZ298840/B.1.351/Beta                CVNFNFNGLTGTGVLTESNKKFLPFQQFGRDIADTTDAVRDPQTLEILDITPCSFGGVSV
LC643036/B.1.617.2/Delta             CVNFNFNGLTGTGVLTESNKKFLPFQQFGRDIADTTDAVRDPQTLEILDITPCSFGGVSV
LC643044/B.1.617.2/Delta             CVNFNFNGLTGTGVLTESNKKFLPFQQFGRDIADTTDAVRDPQTLEILDITPCSFGGVSV
MZ853946/B.1.617.2/Delta             CVNFNFNGLTGTGVLTESNKKFLPFQQFGRDIADTTDAVRDPQTLEILDITPCSFGGVSV
MZ854386/B.1.617.2/Delta             CVNFNFNGLTGTGVLTESNKKFLPFQQFGRDIADTTDAVRDPQTLEILDITPCSFGGVSV
MZ888532/B.1.617.2/Delta             CVNFNFNGLTGTGVLTESNKKFLPFQQFGRDIADTTDAVRDPQTLEILDITPCSFGGVSV
MZ888533/B.1.617.2/Delta             CVNFNFNGLTGTGVLTESNKKFLPFQQFGRDIADTTDAVRDPQTLEILDITPCSFGGVSV
MZ896136/B.1.617.2/Delta             CVNFNFNGLTGTGVLTESNKKFLPFQQFGRDIADTTDAVRDPQTLEILDITPCSFGGVSV
OU539261/B.1.617.2/Delta             CVNFNFNGLTGTGVLTESNKKFLPFQQFGRDIADTTDAVRDPQTLEILDITPCSFGGVSV
MZ854389/B.1.617.2/Delta             CVNFNFNGLTGTGVLTESNKKFLPFQQFGRDIADTTDAVRDPQTLEILDITPCSFGGVSV
OU539784/P.1/Gamma                   CVNFNFNGLTGTGVLTESNKKFLPFQQFGRDIADTTDAVRDPQTLEILDITPCSFGGVSV
OU539830/P.1/Gamma                   CVNFNFNGLTGTGVLTESNKKFLPFQQFGRDIADTTDAVRDPQTLEILDITPCSFGGVSV
OU539894/P.1/Gamma                   CVNFNFNGLTGTGVLTESNKKFLPFQQFGRDIADTTDAVRDPQTLEILDITPCSFGGVSV
MZ477748/P.1/Gamma                   CVNFNFNGLTGTGVLTESNKKFLPFQQFGRDIADTTDAVRDPQTLEILDITPCSFGGVSV
MZ477746/P.1/Gamma                   CVNFNFNGLTGTGVLTESNKKFLPFQQFGRDIADTTDAVRDPQTLEILDITPCSFGGVSV
MW938104/P.1/Gamma                   CVNFNFNGLTGTGVLTESNKKFLPFQQFGRDIADTTDAVRDPQTLEILDITPCSFGGVSV
MZ896179/P.1/Gamma                   CVNFNFNGLTGTGVLTESNKKFLPFQQFGRDIADTTDAVRDPQTLEILDITPCSFGGVSV
MZ896359/P.1/Gamma                   CVNFNFNGLTGTGVLTESNKKFLPFQQFGRDIADTTDAVRDPQTLEILDITPCSFGGVSV
OL677199/BA.1/Omicron                CVNFNFNGLKGTGVLTESNKKFLPFQQFGRDIADTTDAVRDPQTLEILDITPCSFGGVSV
OL672836/BA.1/Omicron                CVNFNFNGLKGTGVLTESNKKFLPFQQFGRDIADTTDAVRDPQTLEILDITPCSFGGVSV
OW714547.1/BA.1/Omicron              CVNFNFNGLKGTGVLTESNKKFLPFQQFGRDIADTTDAVRDPQTLEILDITPCSFGGVSV
OW714198.1/BA.1/Omicron              CVNFNFNGLKGTGVLTESNKKFLPFQQFGRDIADTTDAVRDPQTLEILDITPCSFGGVSV
OW303180.1/BA.1/Omicron              CVNFNFNGLKGTGVLTESNKKFLPFQQFGRDIADTTDAVRDPQTLEILDITPCSFGGVSV
EPI_ISL_7747552/BA.2/Omicron         CVNFNFNGLTGTGVLTESNKKFLPFQQFGRDIADTTDAVRDPQTLEILDITPCSFGGVSV
EPI_ISL_7747545/BA.2/Omicron         CVNFNFNGLTGTGVLTESNKKFLPFQQFGRDIADTTDAVRDPQTLEILDITPCSFGGVSV
EPI_ISL_7747536/BA.2/Omicron         CVNFNFNGLTGTGVLTESNKKFLPFQQFGRDIADTTDAVRDPQTLEILDITPCSFGGVSV
EPI_ISL_7747535/BA.2/Omicron         CVNFNFNGLTGTGVLTESNKKFLPFQQFGRDIADTTDAVRDPQTLEILDITPCSFGGVSV
EPI_ISL_6795834/BA.2/Omicron         CVNFNFNGLTGTGVLTESNKKFLPFQQFGRDIADTTDAVRDPQTLEILDITPCSFGGVSV
EPI_ISL_7718520/BA.2/Omicron         CVNFNFNGLTGTGVLTESNKKFLPFQQFGRDIADTTDAVRDPQTLEILDITPCSFGGVSV
EPI_ISL_7580387/BA.2/Omicron         CVNFNFNGLTGTGVLTESNKKFLPFQQFGRDIADTTDAVRDPQTLEILDITPCSFGGVSV
EPI_ISL_7701122/BA.2/Omicron         CVNFNFNGLTGTGVLTESNKKFLPFQQFGRDIADTTDAVRDPQTLEILDITPCSFGGVSV
EPI_ISL_7190366/BA.2/Omicron         CVNFNFNGLTGTGVLTESNKKFLPFQQFGRDIADTTDAVRDPQTLEILDITPCSFGGVSV
EPI_ISL_7644798/BA.2/Omicron         CVNFNFNGLTGTGVLTESNKKFLPFQQFGRDIADTTDAVRDPQTLEILDITPCSFGGVSV
EPI_ISL_7740766/BA.3/Omicron         CVNFNFNGLTGTGVLTESNKKFLPFQQFGRDIADTTDAVRDPQTLEILDITPCSFGGVSV
EPI_ISL_7605589/BA.3/Omicron         CVNFNFNGLTGTGVLTESNKKFLPFQQFGRDIADTTDAVRDPQTLEILDITPCSFGGVSV
ON296657.1/BA.4/Omicron              CVNFNFNGLTGTGVLTESNKKFLPFQQFGRDIADTTDAVRDPQTLEILDITPCSFGGVSV
ON331287.1/BA.4/Omicron              CVNFNFNGLTGTGVLTESNKKFLPFQQFGRDIADTTDAVRDPQTLEILDITPCSFGGVSV
ON321245.1/BA.4/Omicron              CVNFNFNGLTGTGVLTESNKKFLPFQQFGRDIADTTDAVRDPQTLEILDITPCSFGGVSV
ON337003.1/BA.4/Omicron              CVNFNFNGLTGTGVLTESNKKFLPFQQFGRDIADTTDAVRDPQTLEILDITPCSFGGVSV
ON270681.1/BA.4/Omicron              CVNFNFNGLTGTGVLTESNKKFLPFQQFGRDIADTTDAVRDPQTLEILDITPCSFGGVSV
ON373214.1/BA.4/Omicron              CVNFNFNGLTGTGVLTESNKKFLPFQQFGRDIADTTDAVRDPQTLEILDITPCSFGGVSV
ON393428.1/BA.5/Omicron              CVNFNFNGLTGTGVLTESNKKFLPFQQFGRDIADTTDAVRDPQTLEILDITPCSFGGVSV
ON393156.1/BA.5/Omicron              CVNFNFNGLTGTGVLTESNKKFLPFQQFGRDIADTTDAVRDPQTLEILDITPCSFGGVSV
ON348053.1/BA.5/Omicron              CVNFNFNGLTGTGVLTESNKKFLPFQQFGRDIADTTDAVRDPQTLEILDITPCSFGGVSV
ON323731.1/BA.5/Omicron              CVNFNFNGLTGTGVLTESNKKFLPFQQFGRDIADTTDAVRDPQTLEILDITPCSFGGVSV
ON251467.1/BA.5/Omicron              CVNFNFNGLTGTGVLTESNKKFLPFQQFGRDIADTTDAVRDPQTLEILDITPCSFGGVSV
ON250431.1/BA.5/Omicron              CVNFNFNGLTGTGVLTESNKKFLPFQQFGRDIADTTDAVRDPQTLEILDITPCSFGGVSV
MZ911221/B.1.427/Epsilon             CVNFNFNGLTGTGVLTESNKKFLPFQQFGRDIADTTDAVRDPQTLEILDITPCSFGGVSV
MZ375858/B.1.427/Epsilon             CVNFNFNGLTGTGVLTESNKKFLPFQQFGRDIADTTDAVRDPQTLEILDITPCSFGGVSV
MZ375854/B.1.427/Epsilon             CVNFNFNGLTGTGVLTESNKKFLPFQQFGRDIADTTDAVRDPQTLEILDITPCSFGGVSV
MZ943834/B.1.429/Epsilon             CVNFNFNGLTGTGVLTESNKKFLPFQQFGRDIADTTDAVRDPQTLEILDITPCSFGGVSV
MZ277390/B.1.429/Epsilon             CVNFNFNGLTGTGVLTESNKKFLPFQQFGRDIADTTDAVRDPQTLEILDITPCSFGGVSV
OU282717/B.1.429/Epsilon             CVNFNFNGLTGTGVLTESNKKFLPFQQFGRDIADTTDAVRDPQTLEILDITPCSFGGVSV
OU235436/B.1.429/Epsilon             CVNFNFNGLTGTGVLTESNKKFLPFQQFGRDIADTTDAVRDPQTLEILDITPCSFGGVSV
MZ687447/B.1.429/Epsilon             CVNFNFNGLTGTGVLTESNKKFLPFQQFGRDIADTTDAVRDPQTLEILDITPCSFGGVSV
MZ363839/B.1.617.1/Kappa             CVNFNFNGLTGTGVLTESNKKFLPFQQFGRDIADTTDAVRDPQTLEILDITPCSFGGVSV
MZ538882/B.1.617.1/Kappa             CVNFNFNGLTGTGVLTESNKKFLPFQQFGRDIADTTDAVRDPQTLEILDITPCSFGGVSV
MZ724425/B.1.617.1/Kappa             CVNFNFNGLTGTGVLTESNKKFLPFQQFGRDIADTTDAVRDPQTLEILDITPCSFGGVSV
MZ332524/B.1.617.1/Kappa             CVNFNFNGLTGTGVLTESNKKFLPFQQFGRDIADTTDAVRDPQTLEILDITPCSFGGVSV
MZ332527/B.1.617.1/Kappa             CVNFNFNGLTGTGVLTESNKKFLPFQQFGRDIADTTDAVRDPQTLEILDITPCSFGGVSV
LC633761/B.1.617.1/Kappa             CVNFNFNGLTGTGVLTESNKKFLPFQQFGRDIADTTDAVRDPQTLEILDITPCSFGGVSV
MZ571142/B.1.617.1/Kappa             CVNFNFNGLTGTGVLTESNKKFLPFQQFGRDIADTTDAVRDPQTLEILDITPCSFGGVSV
MZ562746/B.1.617.1/Kappa             CVNFNFNGLTGTGVLTESNKKFLPFQQFGRDIADTTDAVRDPQTLEILDITPCSFGGVSV
OU053018/C.37/Lambda                 CVNFNFNGLTGTGVLTESNKKFLPFQQFGRDIADTTDAVRDPQTLEILDITPCSFGGVSV
OU268528/C.37/Lambda                 CVNFNFNGLTGTGVLTESNKKFLPFQQFGRDIADTTDAVRDPQTLEILDITPCSFGGVSV
OU100999/C.37/Lambda                 CVNFNFNGLTGTGVLTESNKKFLPFQQFGRDIADTTDAVRDPQTLEILDITPCSFGGVSV
MZ275295/C.37/Lambda                 CVNFNFNGLTGTGVLTESNKKFLPFQQFGRDIADTTDAVRDPQTLEILDITPCSFGGVSV
MZ275301/C.37/Lambda                 CVNFNFNGLTGTGVLTESNKKFLPFQQFGRDIADTTDAVRDPQTLEILDITPCSFGGVSV
MZ908711/C.37/Lambda                 CVNFNFNGLTGTGVLTESNKKFLPFQQFGRDIADTTDAVRDPQTLEILDITPCSFGGVSV
OU517038/C.37/Lambda                 CVNFNFNGLTGTGVLTESNKKFLPFQQFGRDIADTTDAVRDPQTLEILDITPCSFGGVSV
MZ496613/C.37/Lambda                 CVNFNFNGLTGTGVLTESNKKFLPFQQFGRDIADTTDAVRDPQTLEILDITPCSFGGVSV
OU466149/B.1.621/Mu                  CVNFNFNGLTGTGVLTESNKKFLPFQQFGRDIADTTDAVRDPQTLEILDITPCSFGGVSV
OU571573/B.1.621/Mu                  CVNFNFNGLTGTGVLTESNKKFLPFQQFGRDIADTTDAVRDPQTLEILDITPCSFGGVSV
OK025270/B.1.621/Mu                  CVNFNFNGLTGTGVLTESNKKFLPFQQFGRDIADTTDAVRDPQTLEILDITPCSFGGVSV
```

```
                                    600       610       620       630       640       650
                                      .         .         .         .         .         .
MN908947/Wuhan-Hu-1               ITPGTNTSNQVAVLYQDVNCTEVPVAIHADQLTPTWRVYSTGSNVFQTRAGCLIGAEHVN
BS001137/B.1.1.7/Alpha            ITPGTNTSNQVAVLYQGVNCTEVPVAIHADQLTPTWRVYSTGSNVFQTRAGCLIGAEHVN
BS001138/B.1.1.7/Alpha            ITPGTNTSNQVAVLYQGVNCTEVPVAIHADQLTPTWRVYSTGSNVFQTRAGCLIGAEHVN
BS001139/B.1.1.7/Alpha            ITPGTNTSNQVAVLYQGVNCTEVPVAIHADQLTPTWRVYSTGSNVFQTRAGCLIGAEHVN
BS001140/B.1.1.7/Alpha            ITPGTNTSNQVAVLYQGVNCTEVPVAIHADQLTPTWRVYSTGSNVFQTRAGCLIGAEHVN
MW913362/B.1.1.7/Alpha            ITPGTNTSNQVAVLYQGVNCTEVPVAIHADQLTPTWRVYSTGSNVFQTRAGCLIGAEHVN
MZ266365/B.1.1.7/Alpha            ITPGTNTSNQVAVLYQGVNCTEVPVAIHADQLTPTWRVYSTGSNVFQTRAGCLIGAEHVN
MZ350109/B.1.1.7/Alpha            ITPGTNTSNQVAVLYQGVNCTEVPVAIHADQLTPTWRVYSTGSNVFQTRAGCLIGAEHVN
MZ888516/B.1.1.7/Alpha            ITPGTNTSNQVAVLYQGVNCTEVPVAIHADQLTPTWRVYSTGSNVFQTRAGCLIGAEHVN
OU562607/B.1.1.7/Alpha            ITPGTNTSNQVAVLYQGVNCTEVPVAIHADQLTPTWRVYSTGSNVFQTRAGCLIGAEHVN
OU514166/B.1.1.7/Alpha            ITPGTNTSNQVAVLYQGVNCTEVPVAIHADQLTPTWRVYSTGSNVFQTRAGCLIGAEHVN
OU538849/B.1.1.7/Alpha            ITPGTNTSNQVAVLYQGVNCTEVPVAIHADQLTPTWRVYSTGSNVFQTRAGCLIGAEHVN
MW981442/B.1.351/Beta             ITPGTNTSNQVAVLYQGVNCTEVPVAIHADQLTPTWRVYSTGSNVFQTRAGCLIGAEHVN
MZ298839/B.1.351/Beta             ITPGTNTSNQVAVLYQGVNCTEVPVAIHADQLTPTWRVYSTGSNVFQTRAGCLIGAEHVN
MZ913100/B.1.351/Beta             ITPGTNTSNQVAVLYQGVNCTEVPVAIHADQLTPTWRVYSTGSNVFQTRAGCLIGAEHVN
MZ879365/B.1.351/Beta             ITPGTNTSNQVAVLYQGVNCTEVPVAIHADQLTPTWRVYSTGSNVFQTRAGCLIGAEHVN
OU539352/B.1.351/Beta             ITPGTNTSNQVAVLYQGVNCTEVPVAIHADQLTPTWRVYSTGSNVFQTRAGCLIGAEHVN
MZ202314/B.1.351/Beta             ITPGTNTSNQVAVLYQGVNCTEVPVAIHADQLTPTWRVYSTGSNVFQTRAGCLIGAEHVN
MZ298840/B.1.351/Beta             ITPGTNTSNQVAVLYQGVNCTEVPVAIHADQLTPTWRVYSTGSNVFQTRAGCLIGAEHVN
LC643036/B.1.617.2/Delta          ITPGTNTSNQVAVLYQGVNCTEVPVAIHADQLTPTWRVYSTGSNVFQTRAGCLIGAEHVN
LC643044/B.1.617.2/Delta          ITPGTNTSNQVAVLYQGVNCTEVPVAIHADQLTPTWRVYSTGSNVFQTRAGCLIGAEHVN
MZ853946/B.1.617.2/Delta          ITPGTNTSNQVAVLYQGVNCTEVPVAIHADQLTPTWRVYSTGSNVFQTRAGCLIGAEHVN
MZ854386/B.1.617.2/Delta          ITPGTNTSNQVAVLYQGVNCTEVPVAIHADQLTPTWRVYSTGSNVFQTRAGCLIGAEHVN
MZ888532/B.1.617.2/Delta          ITPGTNTSNQVAVLYQGVNCTEVPVAIHADQLTPTWRVYSTGSNVFQTRAGCLIGAEHVN
MZ888533/B.1.617.2/Delta          ITPGTNTSNQVAVLYQGVNCTEVPVAIHADQLTPTWRVYSTGSNVFQTRAGCLIGAEHVN
MZ896136/B.1.617.2/Delta          ITPGTNTSNQVAVLYQGVNCTEVPVAIHADQLTPTWRVYSTGSNVFQTRAGCLIGAEHVN
OU539261/B.1.617.2/Delta          ITPGTNTSNQVAVLYQGVNCTEVPVAIHADQLTPTWRVYSTGSNVFQTRAGCLIGAEHVN
MZ854389/B.1.617.2/Delta          ITPGTNTSNQVAVLYQGVNCTEVPVAIHADQLTPTWRVYSTGSNVFQTRAGCLIGAEHVN
OU539784/P.1/Gamma                ITPGTNTSNQVAVLYQGVNCTEVPVAIHADQLTPTWRVYSTGSNVFQTRAGCLIGAEYVN
OU539830/P.1/Gamma                ITPGTNTSNQVAVLYQGVNCTEVPVAIHADQLTPTWRVYSTGSNVFQTRAGCLIGAEYVN
OU539894/P.1/Gamma                ITPGTNTSNQVAVLYQGVNCTEVPVAIHADQLTPTWRVYSTGSNVFQTRAGCLIGAEYVN
MZ477748/P.1/Gamma                ITPGTNTSNQVAVLYQGVNCTEVPVAIHADQLTPTWRVYSTGSNVFQTRAGCLIGAEYVN
MZ477746/P.1/Gamma                ITPGTNTSNQVAVLYQGVNCTEVPVAIHADQLTPTWRVYSTGSNVFQTRAGCLIGAEYVN
MW938104/P.1/Gamma                ITPGTNTSNQVAVLYQGVNCTEVPVAIHADQLTPTWRVYSTGSNVFQTRAGCLIGAEYVN
MZ896179/P.1/Gamma                ITPGTNTSNQVAVLYQGVNCTEVPVAIHADQLTPTWRVYSTGSNVFQTRAGCLIGAEYVN
MZ896359/P.1/Gamma                ITPGTNTSNQVAVLYQGVNCTEVPVAIHADQLTPTWRVYSTGSNVFQTRAGCLIGAEYVN
OL677199/BA.1/Omicron             ITPGTNTSNQVAVLYQGVNCTEVPVAIHADQLTPTWRVYSTGSNVFQTRAGCLIGAEYVN
OL672836/BA.1/Omicron             ITPGTNTSNQVAVLYQGVNCTEVPVAIHADQLTPTWRVYSTGSNVFQTRAGCLIGAEYVN
OW714547.1/BA.1/Omicron           ITPGTNTSNQVAVLYQGVNCTEVPVAIHADQLTPTWRVYSTGSNVFQTRAGCLIGAEYVN
OW714198.1/BA.1/Omicron           ITPGTNTSNQVAVLYQGVNCTEVPVAIHADQLTPTWRVYSTGSNVFQTRAGCLIGAEYVN
OW303180.1/BA.1/Omicron           ITPGTNTSNQVAVLYQGVNCTEVPVAIHADQLTPTWRVYSTGSNVFQTRAGCLIGAEYVN
EPI_ISL_7747552/BA.2/Omicron      ITPGTNTSNQVAVLYQGVNCTEVPVAIHADQLTPTWRVYSTGSNVFQTRAGCLIGAEYVN
EPI_ISL_7747545/BA.2/Omicron      ITPGTNTSNQVAVLYQGVNCTEVPVAIHADQLTPTWRVYSTGSNVFQTRAGCLIGAEYVN
EPI_ISL_7747536/BA.2/Omicron      ITPGTNTSNQVAVLYQGVNCTEVPVAIHADQLTPTWRVYSTGSNVFQTRAGCLIGAEYVN
EPI_ISL_7747535/BA.2/Omicron      ITPGTNTSNQVAVLYQGVNCTEVPVAIHADQLTPTWRVYSTGSNVFQTRAGCLIGAEYVN
EPI_ISL_6795834/BA.2/Omicron      ITPGTNTSNQVAVLYQGVNCTEVPVAIHADQLTPTWRVYSTGSNVFQTRAGCLIGAEYVN
EPI_ISL_7718520/BA.2/Omicron      ITPGTNTSNQVAVLYQGVNCTEVPVAIHADQLTPTWRVYSTGSNVFQTRAGCLIGAEYVN
EPI_ISL_7580387/BA.2/Omicron      ITPGTNTSNQVAVLYQGVNCTEVPVAIHADQLTPTWRVYSTGSNVFQTRAGCLIGAEYVN
EPI_ISL_7701122/BA.2/Omicron      ITPGTNTSNQVAVLYQGVNCTEVPVAIHADQLTPTWRVYSTGSNVFQTRAGCLIGAEYVN
EPI_ISL_7190366/BA.2/Omicron      ITPGTNTSNQVAVLYQGVNCTEVPVAIHADQLTPTWRVYSTGSNVFQTRAGCLIGAEYVN
EPI_ISL_7644798/BA.2/Omicron      ITPGTNTSNQVAVLYQGVNCTEVPVAIHADQLTPTWRVYSTGSNVFQTRAGCLIGAEYVN
EPI_ISL_7740766/BA.3/Omicron      ITPGTNTSNQVAVLYQGVNCTEVPVAIHADQLTPTWRVYSTGSNVFQTRAGCLIGAEYVN
EPI_ISL_7605589/BA.3/Omicron      ITPGTNTSNQVAVLYQGVNCTEVPVAIHADQLTPTWRVYSTGSNVFQTRAGCLIGAEYVN
ON296657.1/BA.4/Omicron           ITPGTNTSNQVAVLYQGVNCTEVPVAIHADQLTPTWRVYSTGSNVFQTRAGCLIGAEYVN
ON331287.1/BA.4/Omicron           ITPGTNTSNQVAVLYQGVNCTEVPVAIHADQLTPTWRVYSTGSNVFQTRAGCLIGAEYVN
ON321245.1/BA.4/Omicron           ITPGTNTSNQVAVLYQGVNCTEVPVAIHADQLTPTWRVYSTGSNVFQTRAGCLIGAEYVN
ON337003.1/BA.4/Omicron           ITPGTNTSNQVAVLYQGVNCTEVPVAIHADQLTPTWRVYSTGSNVFQTRAGCLIGAEYVN
ON270681.1/BA.4/Omicron           ITPGTNTSNQVAVLYQGVNCTEVPVAIHADQLTPTWRVYSTGSNVFQTRAGCLIGAEYVN
ON373214.1/BA.4/Omicron           ITPGTNTSNQVAVLYQGVNCTEVPVAIHADQLTPTWRVYSTGSNVFQTRAGCLIGAEYVN
ON393428.1/BA.5/Omicron           ITPGTNTSNQVAVLYQGVNCTEVPVAIHADQLTPTWRVYSTGSNVFQTRAGCLIGAEYVN
ON393156.1/BA.5/Omicron           ITPGTNTSNQVAVLYQGVNCTEVPVAIHADQLTPTWRVYSTGSNVFQTRAGCLIGAEYVN
ON348053.1/BA.5/Omicron           ITPGTNTSNQVAVLYQGVNCTEVPVAIHADQLTPTWRVYSTGSNVFQTRAGCLIGAEYVN
ON323731.1/BA.5/Omicron           ITPGTNTSNQVAVLYQGVNCTEVPVAIHADQLTPTWRVYSTGSNVFQTRAGCLIGAEYVN
ON251467.1/BA.5/Omicron           ITPGTNTSNQVAVLYQGVNCTEVPVAIHADQLTPTWRVYSTGSNVFQTRAGCLIGAEYVN
ON250431.1/BA.5/Omicron           ITPGTNTSNQVAVLYQGVNCTEVPVAIHADQLTPTWRVYSTGSNVFQTRAGCLIGAEYVN
MZ911221/B.1.427/Epsilon          ITPGTNTSNQVAVLYQGVNCTEVPVAIHADQLTPTWRVYSTGSNVFQTRAGCLIGAEHVN
MZ375858/B.1.427/Epsilon          ITPGTNTSNQVAVLYQGVNCTEVPVAIHADQLTPTWRVYSTGSNVFQTRAGCLIGAEHVN
MZ375854/B.1.427/Epsilon          ITPGTNTSNQVAVLYQGVNCTEVPVAIHADQLTPTWRVYSTGSNVFQTRAGCLIGAEHVN
MZ943834/B.1.429/Epsilon          ITPGTNTSNQVAVLYQGVNCTEVPVAIHADQLTPTWRVYSTGSNVFQTRAGCLIGAEHVN
MZ277390/B.1.429/Epsilon          ITPGTNTSNQVAVLYQGVNCTEVPVAIHADQLTPTWRVYSTGSNVFQTRAGCLIGAEHVN
OU282717/B.1.429/Epsilon          ITPGTNTSNQVAVLYQGVNCTEVPVAIHADQLTPTWRVYSTGSNVFQTRAGCLIGAEHVN
OU235436/B.1.429/Epsilon          ITPGTNTSNQVAVLYQGVNCTEVPVAIHADQLTPTWRVYSTGSNVFQTRAGCLIGAEHVN
MZ687447/B.1.429/Epsilon          ITPGTNTSNQVAVLYQGVNCTEVPVAIHADQLTPTWRVYSTGSNVFQTRAGCLIGAEHVN
MZ363839/B.1.617.1/Kappa          ITPGTNTSNQVAVLYQGVNCTEVPVAIHADQLTPTWRVYSTGSNVFQTRAGCLIGAEHVN
MZ538882/B.1.617.1/Kappa          ITPGTNTSNQVAVLYQGVNCTEVPVAIHADQLTPTWRVYSTGSNVFQTRAGCLIGAEHVN
MZ724425/B.1.617.1/Kappa          ITPGTNTSNQVAVLYQGVNCTEVPVAIHADQLTPTWRVYSTGSNVFQTRAGCLIGAEHVN
MZ332524/B.1.617.1/Kappa          ITPGTNTSNQVAVLYQGVNCTEVPVAIHADQLTPTWRVYSTGSNVFQTRAGCLIGAEHVN
MZ332527/B.1.617.1/Kappa          ITPGTNTSNQVAVLYQGVNCTEVPVAIHADQLTPTWRVYSTGSNVFQTRAGCLIGAEHVN
LC633761/B.1.617.1/Kappa          ITPGTNTSNQVAVLYQGVNCTEVPVAIHADQLTPTWRVYSTGSNVFQTRAGCLIGAEHVN
MZ571142/B.1.617.1/Kappa          ITPGTNTSNQVAVLYQGVNCTEVPVAIHADQLTPTWRVYSTGSNVFQTRAGCLIGAEHVN
MZ562746/B.1.617.1/Kappa          ITPGTNTSNQVAVLYQGVNCTEVPVAIHADQLTPTWRVYSTGSNVFQTRAGCLIGAEHVN
OU053018/C.37/Lambda              ITPGTNTSNQVAVLYQGVNCTEVPVAIHADQLTPTWRVYSTGSNVFQTRAGCLIGAEHVN
OU268528/C.37/Lambda              ITPGTNTSNQVAVLYQGVNCTEVPVAIHADQLTPTWRVYSTGFNVFQTRAGCLIGAEHVN
OU100999/C.37/Lambda              ITPGTNTSNQVAVLYQGVNCTEVPVAIHADQLTPTWRVYSTGSNVFQTRAGCLIGAEHVN
MZ275295/C.37/Lambda              ITPGTNTSNQVAVLYQGVNCTEVPVAIHADQLTPTWRVYSTGSNVFQTRAGCLIGAEHVN
MZ275301/C.37/Lambda              ITPGTNTSNQVAVLYQGVNCTEVPVAIHADQLTPTWRVYSTGSNVFQTRAGCLIGAEHVN
MZ908711/C.37/Lambda              ITPGTNTSNQVAVLYQGVNCTEVPVAIHADQLTPTWRVYSTGSNVFQTRAGCLIGAEHVN
OU517038/C.37/Lambda              ITPGTNTSNQVAVLYQGVNCTEVPVAIHADQLTPTWRVYSTGSNVFQTRAGCLIGAEHVN
MZ496613/C.37/Lambda              ITPGTNTSNQVAVLYQGVNCTEVPVAIHADQLTPTWRVYSTGSNVFQTRAGCLIGAEHVN
OU466149/B.1.621/Mu               ITPGTNTSNQVAVLYQGVNCTEVPVAIHADQLTPTWRVYSTGSNVFQTRAGCLIGAEHVN
OU571573/B.1.621/Mu               ITPGTNTSNQVAVLYQGVNCTEVPVAIHADQLTPTWRVYSTGSNVFQTRAGCLIGAEHVN
OK025270/B.1.621/Mu               ITPGTNTSNQVAVLYQGVNCTEVPVAIHADQLTPTWRVYSTGSNVFQTRAGCLIGAEHVN
```

```
                                     660       670       680       690       700       710
MN908947/Wuhan-Hu-1                NSYECDIPIGAGICASYQTQTNSPRRARSVASQSIIAYTMSLGAENSVAYSNNSIAIPTN
BS001137/B.1.1.7/Alpha             NSYECDIPIGAGICASYQTQTNSHRRARSVASQSIIAYTMSLGAENSVAYSNNSIAIPIN
BS001138/B.1.1.7/Alpha             NSYECDIPIGAGICASYQTQTNSHRRARSVASQSIIAYTMSLGAENSVAYSNNSIAIPIN
BS001139/B.1.1.7/Alpha             NSYECDIPIGAGICASYQTQTNSHRRARSVASQSIIAYTMSLGAENSVAYSNNSIAIPIN
BS001140/B.1.1.7/Alpha             NSYECDIPIGAGICASYQTQTNSHRRARSVASQSIIAYTMSLGAENSVAYSNNSIAIPIN
MW913362/B.1.1.7/Alpha             NSYECDIPIGAGICASYQTQTNSHRRARSVASQSIIAYTMSLGAENSVAYSNNSIAIPIN
MZ266365/B.1.1.7/Alpha             NSYECDIPIGAGICASYQTQTNSHRRARSVASQSIIAYTMSLGAENSVAYSNNSIAIPIN
MZ350109/B.1.1.7/Alpha             NSYECDIPIGAGICASYQTQTNSHRRARSVASQSIIAYTMSLGAENSVAYSNNSIAIPIN
MZ888516/B.1.1.7/Alpha             NSYECDIPIGAGICASYQTQTNSHRRARSVASQSIIAYTMSLGAENSVAYSNNSIAIPIN
OU562607/B.1.1.7/Alpha             NSYECDIPIGAGICASYQTQTNSHRRARSVASQSIIAYTMSLGAENSVAYSNNSIAIPIN
OU514166/B.1.1.7/Alpha             NSYECDIPIGAGICASYQTQTNSHRRARSVASQSIIAYTMSLGAENSVAYSNNSIAIPIN
OU538849/B.1.1.7/Alpha             NSYECDIPIGAGICASYQTQTNSHRRARSVASQSIIAYTMSLGAENSVAYSNNSIAIPIN
MW981442/B.1.351/Beta              NSYECDIPIGAGICASYQTHTNSPWRARSVASQSIIAYTMSLGVENSVAYSNNSIAIPTN
MZ298839/B.1.351/Beta              NSYECDIPIGAGICASYQTQTNSPRRARSVASQSIIAYTMSLGVENSVAYSNNSIAIPTN
MZ913100/B.1.351/Beta              NSYECDIPIGAGICASYQTQTNSPRRARSVASQSIIAYTMSLGVENSVAYSNNSIAIPTN
MZ879365/B.1.351/Beta              NSYECDIPIGAGICASYQTQTNSPRRARSVASQSIIAYTMSLGVENSVAYSNNSIAIPTN
OU539352/B.1.351/Beta              NSYECDIPIGAGICASYQTQTNSPRRARSVASQSIIAYTMSLGVENSVAYSNNSIAIPTN
MZ202314/B.1.351/Beta              NSYECDIPIGAGICASYQTHTNSPWRARSVASQSIIAYTMSLGVENSVAYSNNSIAIPTN
MZ298840/B.1.351/Beta              NSYECDIPIGAGICASYQTQTNSPRRARSVASQSIIAYTMSLGVENSVAYSNNSIAIPTN
LC643036/B.1.617.2/Delta           NSYECDIPIGAGICASYQTQTNSRRRARSVASQSIIAYTMSLGAENSVAYSNNSIAIPTN
LC643044/B.1.617.2/Delta           NSYECDIPIGAGICASYQTQTNSRRRARSVASQSIIAYTMSLGAENSVAYSNNSIAIPTN
MZ853946/B.1.617.2/Delta           NSYECDIPIGAGICASYQTQTNSRRRARSVASQSIIAYTMSLGAENSVAYSNNSIAIPTN
MZ854386/B.1.617.2/Delta           NSYECDIPIGAGICASYQTQTNSRRRARSVASQSIIAYTMSLGAENSVAYSNNSIAIPTN
MZ888532/B.1.617.2/Delta           NSYECDIPIGAGICASYQTQTNSRRRARSVASQSIIAYTMSLGAENSVAYSNNSIAIPTN
MZ888533/B.1.617.2/Delta           NSYECDIPIGAGICASYQTQTNSRRRARSVASQSIIAYTMSLGAENSVAYSNNSIAIPTN
MZ896136/B.1.617.2/Delta           NSYECDIPIGAGICASYQTQTNSRRRARSVASQSIIAYTMSLGAENSVAYSNNSIAIPTN
OU539261/B.1.617.2/Delta           NSYECDIPIGAGICASYQTQTNSRRRARSVASQSIIAYTMSLGAENSVAYSNNSIAIPTN
MZ854389/B.1.617.2/Delta           NSYECDIPIGAGICASYQTQTNSRRRARSVASQSIIAYTMSLGAENSVAYSNNSIAIPTN
OU539784/P.1/Gamma                 NSYECDIPIGAGICASYQTQTNSPRRARSVASQSIIAYTMSLGAENSVAYSNNSIAIPTN
OU539830/P.1/Gamma                 NSYECDIPIGAGICASYQTQTNSPRRARSVASQSIIAYTMSLGAENSVAYSNNSIAIPTN
OU539894/P.1/Gamma                 NSYECDIPIGAGICASYQTQTNSPRRARSVASQSIIAYTMSLGAENSVAYSNNSIAIPTN
MZ477748/P.1/Gamma                 NSYECDIPIGAGICASYQTQTNSPRRARSVASQSIIAYTMSLGAENSVAYSNNSIAIPTN
MZ477746/P.1/Gamma                 NSYECDIPIGAGICASYQTQTNSPRRARSVASQSIIAYTMSLGAENSVAYSNNSIAIPTN
MW938104/P.1/Gamma                 NSYECDIPIGAGICASYQTQTNSPRRARSVASQSIIAYTMSLGAENSVAYSNNSIAIPTN
MZ896179/P.1/Gamma                 NSYECDIPIGAGICASYQTQTNSPRRARSVASQSIIAYTMSLGAENSVAYSNNSIAIPTN
MZ896359/P.1/Gamma                 NSYECDIPIGAGICASYQTQTNSPRRARSVASQSIIAYTMSLGAENSVAYSNNSIAIPTN
OL677199/BA.1/Omicron              NSYECDIPIGAGICASYQTQTKSHRRARSVASQSIIAYTMSLGAENSVAYSNNSIAIPTN
OL672836/BA.1/Omicron              NSYECDIPIGAGICASYQTQTKSHRRARSVASQSIIAYTMSLGAENSVAYSNNSIAIPTN
OW714547.1/BA.1/Omicron            NSYECDIPIGAGICASYQTQTKSHRRARSVASQSIIAYTMSLGAENSVAYSNNSIAIPTN
OW714198.1/BA.1/Omicron            NSYECDIPIGAGICASYQTQTKSHRRARSVASQSIIAYTMSLGAENSVAYSNNSIAIPTN
OW303180.1/BA.1/Omicron            NSYECDIPIGAGICASYQTQTKSHRRARSVASQSIIAYTMSLGAENSVAYSNNSIAIPTN
EPI_ISL_7747552/BA.2/Omicron       NSYECDIPIGAGICASYQTQTKSHRRARSVASQSIIAYTMSLGAENSVAYSNNSIAIPTN
EPI_ISL_7747545/BA.2/Omicron       NSYECDIPIGAGICASYQTQTKSHRRARSVASQSIIAYTMSLGAENSVAYSNNSIAIPTN
EPI_ISL_7747536/BA.2/Omicron       NSYECDIPIGAGICASYQTQTKSHRRARSVASQSIIAYTMSLGAENSVAYSNNSIAIPTN
EPI_ISL_7747535/BA.2/Omicron       NSYECDIPIGAGICASYQTQTKSHRRARSVASQSIIAYTMSLGAENSVAYSNNSIAIPTN
EPI_ISL_6795834/BA.2/Omicron       NSYECDIPIGAGICASYQTQTKSHRRARSVASQSIIAYTMSLGAENSVAYSNNSIAIPTN
EPI_ISL_7718520/BA.2/Omicron       NSYECDIPIGAGICASYQTQTKSHRRARSVASQSIIAYTMSLGAENSVAYSNNSIAIPTN
EPI_ISL_7580387/BA.2/Omicron       NSYECDIPIGAGICASYQTQTKSHRRARSVASQSIIAYTMSLGAENSVAYSNNSIAIPTN
EPI_ISL_7701122/BA.2/Omicron       NSYECDIPIGAGICASYQTQTKSHRRARSVASQSIIAYTMSLGAENSVAYSNNSIAIPTN
EPI_ISL_7190366/BA.2/Omicron       NSYECDIPIGAGICASYQTQTKSHRRARSVASQSIIAYTMSLGAENSVAYSNNSIAIPTN
EPI_ISL_7644798/BA.2/Omicron       NSYECDIPIGAGICASYQTQTKSHRRARSVASQSIIAYTMSLGAENSVAYSNNSIAIPTN
EPI_ISL_7740766/BA.3/Omicron       NSYECDIPIGAGICASYQTQTKSHRRARSVASQSIIAYTMSLGAENSVAYSNNSIAIPTN
EPI_ISL_7605589/BA.3/Omicron       NSYECDIPIGAGICASYQTQTKSHRRARSVASQSIIAYTMSLGAENSVAYSNNSIAIPTN
ON296657.1/BA.4/Omicron            NSYECDIPIGAGICASYQTQTKSHRRARSVASQSIIAYTMSLGAENSVAYSNNSIAIPTN
ON331287.1/BA.4/Omicron            NSYECDIPIGAGICASYQTQTKSHRRARSVASQSIIAYTMSLGAENSVAYSNNSIAIPTN
ON321245.1/BA.4/Omicron            NSYECDIPIGAGVCASYQTQTKSHRRARSVASQSIIAYTMSLGAENSVAYSNNSIAIPTN
ON337003.1/BA.4/Omicron            NSYECDIPIGAGICASYQTQTKSHRRARSVASQSIIAYTMSLGAENSVAYSNNSIAIPTN
ON270681.1/BA.4/Omicron            NSYECDIPIGAGICASYQTQTKSHRRARSVASQSIIAYTMSLGAENSVAYSNNSIAIPTN
ON373214.1/BA.4/Omicron            NSYECDIPIGAGICASYQTQTKSHRRARSVASQSIIAYTMSLGAENSVAYSNNSIAIPTN
ON393428.1/BA.5/Omicron            NSYECDIPIGAGICASYQTQTKSHRRARSVASQSIIAYTMSLGAENSVAYSNNSIAIPTN
ON393156.1/BA.5/Omicron            NSYECDIPIGAGICASYQTQTKSHRRARSVASQSIIAYTMSLGAENSVAYSNNSIAIPTN
ON348053.1/BA.5/Omicron            NSYECDIPIGAGICASYQTQTKSHRRARSVASQSIIAYTMSLGAENSVAYSNNSIAIPTN
ON323731.1/BA.5/Omicron            NSYECDIPIGAGICASYQTQTKSHRRARSVASQSIIAYTMSLGAENSVAYSNNSIAIPTN
ON251467.1/BA.5/Omicron            NSYECDIPIGAGICASYQTQTKSHRRARSVASQSIIAYTMSLGAENSVAYSNNSIAIPTN
ON250431.1/BA.5/Omicron            NSYECDIPIGAGICASYQTQTKSHRRARSVASQSIIAYTMSLGAENSVAYSNNSIAIPTN
MZ911221/B.1.427/Epsilon           NSYECDIPIGAGICASYQTQTNSPRRARSVASQSIIAYTMSLGAENSVAYSNNSIAIPTN
MZ375858/B.1.427/Epsilon           NSYECDIPIGAGICASYQTQTNSHRRARSVASQSIIAYTMSLGAENSVAYSNNSIAIPTN
MZ375854/B.1.427/Epsilon           NSYECDIPIGAGICASYQTQTNSPRRARSVASQSIIAYTMSLGAENSVAYSNNSIAIPTN
MZ943834/B.1.429/Epsilon           NSYECDIPIGAGICASYQTQTNSPRRARSVASQSIIAYTMSLGAENSVAYSNNSIAIPTN
MZ277390/B.1.429/Epsilon           NSYECDIPIGAGICASYQTQTNSPRRARSVASQSIIAYTMSLGAENSVAYSNNSIAIPTN
OU282717/B.1.429/Epsilon           NSYECDIPIGAGICASYQTQTNSPRRARSVASQSIIAYTMSLGAENSVAYSNNSIAIPTN
OU235436/B.1.429/Epsilon           NSYECDIPIGAGICASYQTQTNSPRRARSVASQSIIAYTMSLGAENSVAYSNNSIAIPTN
MZ687447/B.1.429/Epsilon           NSYECDIPIGAGICASYQTQTNSPRRARSVASQSIIAYTMSLGAENSVAYSNNSIAIPTN
MZ363839/B.1.617.1/Kappa           NSYECDIPIGAGICASYQTQTNSRRRARSVASQSIIAYTMSLGAENSVAYSNNSIAIPTN
MZ538882/B.1.617.1/Kappa           NSYECDIPIGAGICASYQTQTNSRRRARSVASQSIIAYTMSLGAENSVAYSNNSIAIPTN
MZ724425/B.1.617.1/Kappa           NSYECDIPIGAGICASYQTQTNSRRRARSVASQSIIAYTMSLGAENSVAYSNNSIAIPTN
MZ332524/B.1.617.1/Kappa           NSYECDIPIGAGICASYQTQTNSRRRARSVASQSIIAYTMSLGAENSVAYSNNSIAIPTN
MZ332527/B.1.617.1/Kappa           NSYECDIPIGAGICASYQTQTNSRRRARSVASQSIIAYTMSLGAENSVAYSNNSIAIPTN
LC633761/B.1.617.1/Kappa           NSYECDIPIGAGICASYQTQTNSRRRARSVASQSIIAYTMSLGAENSVAYSNNSIAIPTN
MZ571142/B.1.617.1/Kappa           NSYECDIPIGAGICASYQTQTNSRRRARSVASQSIIAYTMSLGAENSVAYSNNSIAIPTN
MZ562746/B.1.617.1/Kappa           NSYECDIPIGAGICASYQTQTNSRRRARSVASQSIIAYTMSLGAENSVAYSNNSIAIPTN
OU053018/C.37/Lambda               NSYECDIPIGAGICASYQTQTNSPRRARSVASQSIIAYTMSLGAENSVAYSNNSIAIPTN
OU268528/C.37/Lambda               NSYECDIPIGAGICASYQTQTNSPRRARSVASQSIIAYTMSLGAENSVAYSNNSIAIPTN
OU100999/C.37/Lambda               NSYECDIPIGAGICASYQTQTNSPRRARSVASQSIIAYTMSLGAENSVAYSNNSIAVPTN
MZ275295/C.37/Lambda               NSYECDIPIGAGICASYQTQTNSPRRARSVASQSIIAYTMSLGAENSVAYSNNSIAIPTN
MZ275301/C.37/Lambda               NSYECDIPIGAGICASYQTQTNSPRRARSVASQSIIAYTMSLGAENSVAYSNNSIAIPTN
MZ908711/C.37/Lambda               NSYECDIPIGAGICASYQTQTNSPRRARSVASQSIIAYTMSLGAENSVAYSNNSIAIPTN
OU517038/C.37/Lambda               NSYECDIPIGAGICASYQTQTNSPRRARSVASQSIIAYTMSLGAENSVAYSNNSIAIPTN
MZ496613/C.37/Lambda               NSYECDIPIGAGICASYQTQTNSPRRARSVASQSIIAYTMSLGAENSVAYSNNSIAIPTN
OU466149/B.1.621/Mu                NSYECDIPIGAGICASYQTQTNSHRRARSVASQSIIAYTMSLGAENSVAYSNNSIAIPTN
OU571573/B.1.621/Mu                NSYECDIPIGAGICASYQTQTNSHRRARSVASQSIIAYTMSLGAENSVAYSNNSIAIPTN
OK025270/B.1.621/Mu                NSYECDIPIGAGICASYQTQTNSHRRARSVASQSIIAYTMSLGAENSVAYSNNSIAIPTN
```

```
                                720       730       740       750       760       770
MN908947/Wuhan-Hu-1             FTISVTTEILPVSMTKTSVDCTMYICGDSTECSNLLLQYGSFCTQLNRALTGIAVEQDKN
BS001137/B.1.1.7/Alpha          FTISVTTEILPVSMTKTSVDCTMYICGDSTECSNLLLQYGSFCTQLNRALTGIAVEQDKN
BS001138/B.1.1.7/Alpha          FTISVTTEILPVSMTKTSVDCTMYICGDSTECSNLLLQYGSFCTQLNRALTGIAVEQDKN
BS001139/B.1.1.7/Alpha          FTISVTTEILPVSMTKTSVDCTMYICGDSTECSNLLLQYGSFCTQLNRALTGIAVEQDKN
BS001140/B.1.1.7/Alpha          FTISVTTEILPVSMTKTSVDCTMYICGDSTECSNLLLQYGSFCTQLNRALTGIAVEQDKN
MW913362/B.1.1.7/Alpha          FTISVTTEILPVSMTKTSVDCTMYICGDSTECSNLLLQYGSFCTQLNRALTGIAVEQDKN
MZ266365/B.1.1.7/Alpha          FTISVTTEILPVSMTKTSVDCTMYICGDSTECSNLLLQYGSFCTQLNRALTGIAVEQDKN
MZ350109/B.1.1.7/Alpha          FTISVTTEILPVSMTKTSVDCTMYICGDSTECSNLLLQYGSFCTQLNRALTGIAVEQDKN
MZ888516/B.1.1.7/Alpha          FTISVTTEILPVSMTKTSVDCTMYICGDSTECSNLLLQYGSFCTQLNRALTGIAVEQDKN
OU562607/B.1.1.7/Alpha          FTISVTTEILPVSMTKTSVDCTMYICGDSTECSNLLLQYGSFCTQLNRALTGIAVEQDKN
OU514166/B.1.1.7/Alpha          FTISVTTEILPVSMTKTSVDCTMYICGDSTECSNLLLQYGSFCTQLNRALTGIAVEQDKN
OU538849/B.1.1.7/Alpha          FTISVTTEILPVSMTKTSVDCTMYICGDSTECSNLLLQYGSFCTQLNRALTGIAVEQDKN
MW981442/B.1.351/Beta           FTISVTTEILPVSMTKTSVDCTMYICGDSTECSNLLLQYGSFCTQLNRALTGIAVEQDKN
MZ298839/B.1.351/Beta           FTISVTTEILPVSMTKTSVDCTMYICGDSTECSNLLLQYGSFCTQLNRALTGIAVEQDKN
MZ913100/B.1.351/Beta           FTISVTTEILPVSMTKTSVDCTMYICGDSTECSNLLLQYGSFCTQLNRALTGIAVEQDKN
MZ879365/B.1.351/Beta           FTISVTTEILPVSMTKTSVDCTMYICGDSTECSNLLLQYGSFCTQLNRALTGIAVEQDKN
OU539352/B.1.351/Beta           FTISVTTEILPVSMTKTSVDCTMYICGDSTECSNLLLQYGSFCTQLNRALTGIAVEQDKN
MZ202314/B.1.351/Beta           FTISVTTEILPVSMTKTSVDCTMYICGDSTECSNLLLQYGSFCTQLNRALTGIAVEQDKN
MZ298840/B.1.351/Beta           FTISVTTEILPVSMTKTSVDCTMYICGDSTECSNLLLQYGSFCTQLNRALTGIAVEQDKN
LC643036/B.1.617.2/Delta        FTISVTTEILPVSMTKTSVDCTMYICGDSTECSNLLLQYGSFCTQLNRALTGIAVEQDKN
LC643044/B.1.617.2/Delta        FTISVTTEILPVSMTKTSVDCTMYICGDSTECSNLLLQYGSFCTQLNRALTGIAVEQDKN
MZ853946/B.1.617.2/Delta        FTISVTTEILPVSMTKTSVDCTMYICGDSTECSNLLLQYGSFCTQLNRALTGIAVEQDKN
MZ854386/B.1.617.2/Delta        FTISVTTEILPVSMTKTSVDCTMYICGDSTECSNLLLQYGSFCTQLNRALTGIAVEQDKN
MZ888532/B.1.617.2/Delta        FTISVTTEILPVSMTKTSVDCTMYICGDSTECSNLLLQYGSFCTQLNRALTGIAVEQDKN
MZ888533/B.1.617.2/Delta        FTISVTTEILPVSMTKTSVDCTMYICGDSTECSNLLLQYGSFCTQLNRALTGIAVEQDKN
MZ896136/B.1.617.2/Delta        FTISVTTEILPVSMTKTSVDCTMYICGDSTECSNLLLQYGSFCTQLNRALTGIAVEQDKN
OU539261/B.1.617.2/Delta        FTISVTTEILPVSMTKTSVDCTMYICGDSTECSNLLLQYGSFCTQLNRALTGIAVEQDKN
MZ854389/B.1.617.2/Delta        FTISVTTEILPVSMTKTSVDCTMYICGDSTECSNLLLQYGSFCTQLNRALTGIAVEQDKN
OU539784/P.1/Gamma              FTISVTTEILPVSMTKTSVDCTMYICGDSTECSNLLLQYGSFCTQLNRALTGIAVEQDKN
OU539830/P.1/Gamma              FTISVTTEILPVSMTKTSVDCTMYICGDSTECSNLLLQYGSFCTQLNRALTGIAVEQDKN
OU539894/P.1/Gamma              FTISVTTEILPVSMTKTSVDCTMYICGDSTECSNLLLQYGSFCTQLNRALTGIAVEQDKN
MZ477748/P.1/Gamma              FTISVTTEILPVSMTKTSVDCTMYICGDSTECSNLLLQYGSFCTQLNRALTGIAVEQDKN
MZ477746/P.1/Gamma              FTISVTTEILPVSMTKTSVDCTMYICGDSTECSNLLLQYGSFCTQLNRALTGIAVEQDKN
MW938104/P.1/Gamma              FTISVTTEILPVSMTKTSVDCTMYICGDSTECSNLLLQYGSFCTQLNRALTGIAVEQDKN
MZ896179/P.1/Gamma              FTISVTTEILPVSMTKTSVDCTMYICGDSTECSNLLLQYGSFCTQLNRALTGIAVEQDKN
MZ896359/P.1/Gamma              FTISVTTEILPVSMTKTSVDCTMYICGDSTECSNLLLQYGSFCTQLNRALTGIAVEQDKN
OL677199/BA.1/Omicron           FTISVTTEILPVSMTKTSVDCTMYICGDSTECSNLLLQYGSFCTQLKRALTGIAVEQDKN
OL672836/BA.1/Omicron           FTISVTTEILPVSMTKTSVDCTMYICGDSTECSNLLLQYGSFCTQLKRALTGIAVEQDKN
OW714547.1/BA.1/Omicron         FTISVTTEILPVSMTKTSVDCTMYICGDSTECSNLLLQYGSFCTQLKRALTGIAVEQDKN
OW714198.1/BA.1/Omicron         FTISVTTEILPVSMTKTSVDCTMYICGDSTECSNLLLQYGSFCTQLKRALTGIAVEQDKN
OW303180.1/BA.1/Omicron         FTISVTTEILPVSMTKTSVDCTMYICGDSTECSNLLLQYGSFCTQL.RALTGIAVEQDKN
EPI_ISL_7747552/BA.2/Omicron    FTISVTTEILPVSMTKTSVDCTMYICGDSTECSNLLLQYGSFCTQLKRALTGIAVEQDKN
EPI_ISL_7747545/BA.2/Omicron    FTISVTTEILPVSMTKTSVDCTMYICGDSTECSNLLLQYGSFCTQLKRALTGIAVEQDKN
EPI_ISL_7747536/BA.2/Omicron    FTISVTTEILPVSMTKTSVDCTMYICGDSTECSNLLLQYGSFCTQLKRALTGIAVEQDKN
EPI_ISL_7747535/BA.2/Omicron    FTISVTTEILPVSMTKTSVDCTMYICGDSTECSNLLLQYGSFCTQLKRALTGIAVEQDKN
EPI_ISL_6795834/BA.2/Omicron    FTISVTTEILPVSMTKTSVDCTMYICGDSTECSNLLLQYGSFCTQLKRALTGIAVEQDKN
EPI_ISL_7718520/BA.2/Omicron    FTISVTTEILPVSMTKTSVDCTMYICGDSTECSNLLLQYGSFCTQLKRALTGIAVEQDKN
EPI_ISL_7580387/BA.2/Omicron    FTISVTTEILPVSMTKTSVDCTMYICGDSTECSNLLLQYGSFCTQLKRALTGIAVEQDKN
EPI_ISL_7701122/BA.2/Omicron    FTISVTTEILPVSMTKTSVDCTMYICGDSTECSNLLLQYGSFCTQLKRALTGIAVEQDKN
EPI_ISL_7190366/BA.2/Omicron    FTISVTTEILPVSMTKTSVDCTMYICGDSTECSNLLLQYGSFCTQLKRALTGIAVEQDKN
EPI_ISL_7644798/BA.2/Omicron    FTISVTTEILPVSMTKTSVDCTMYICGDSTECSNLLLQYGSFCTQLKRALTGIAVEQDKN
EPI_ISL_7740766/BA.3/Omicron    FTISVTTEILPVSMTKTSVDCTMYICGDSTECSNLLLQYGSFCTQLKRALTGIAVEQDKN
EPI_ISL_7605589/BA.3/Omicron    FTISVTTEILPVSMTKTSVDCTMYICGDSTECSNLLLQYGSFCTQLKRALTGIAVEQDKN
ON296657.1/BA.4/Omicron         FTISVTTEILPVSMTKTSVDCTMYICGDSTECSNLLLQYGSFCTQLKRALTGIAVEQDKN
ON331287.1/BA.4/Omicron         FTISVTTEILPVSMTKTSVDCTMYICGDSTECSNLLLQYGSFCTQLKRALTGIAVEQDKN
ON321245.1/BA.4/Omicron         FTISVTTEILPVSMTKTSVDCTMYICGDSTECSNLLLQYGSFCTQLKRALTGIAVEQDKN
ON337003.1/BA.4/Omicron         FTISVTTEILPVSMTKTSVDCTMYICGDSTECSNLLLQYGSFCTQLKRALTGIAVEQDKN
ON270681.1/BA.4/Omicron         FTISVTTEILPVSMTKTSVDCTMYICGDSTECSNLLLQYGSFCTQLKRALTGIAVEQDKN
ON373214.1/BA.4/Omicron         FTISVTTEILPVSMTKTSVDCTMYICGDSTECSNLLLQYGSFCTQLKRALTGIAVEQDKN
ON393428.1/BA.5/Omicron         FTISVTTEILPVSMTKTSVDCTMYICGDSTECSNLLLQYGSFCTQLKRALTGIAVEQDKN
ON393156.1/BA.5/Omicron         FTISVTTEILPVSMTKTSVDCTMYICGDSTECSNLLLQYGSFCTQLKRALTGIAVEQDKN
ON348053.1/BA.5/Omicron         FTISVTTEILPVSMTKTSVDCTMYICGDSTECSNLLLQYGSFCTQLKRALTGIAVEQDKN
ON323731.1/BA.5/Omicron         FTISVTTEILPVSMTKTSVDCTMYICGDSTECSNLLLQYGSFCTQLKRALTGIAVEQDKN
ON251467.1/BA.5/Omicron         FTISVTTEILPVSMTKTSVDCTMYICGDSTECSNLLLQYGSFCTQLKRALTGIAVEQDKN
ON250431.1/BA.5/Omicron         FTISVTTEILPVSMTKTSVDCTMYICGDSTECSNLLLQYGSFCTQLKRALTGIAVEQDKN
MZ911221/B.1.427/Epsilon        FTISVTTEILPVSMTKTSVDCTMYICGDSTECSNLLLQYGSFCTQLNRALTGIAVEQDKN
MZ375858/B.1.427/Epsilon        FTISVTTEILPVSMTKTSVDCTMYICGDSTECSNLLLQYGSFCTQLNRALTGIAVEQDKN
MZ375854/B.1.427/Epsilon        FTISVTTEILPVSMTKTSVDCTMYICGDSTECSNLLLQYGSFCTQLNRALTGIAVEQDKN
MZ943834/B.1.429/Epsilon        FTISVTTEILPVSMTKTSVDCTMYICGDSTECSNLLLQYGSFCTQLNRALTGIAVEQDKN
MZ277390/B.1.429/Epsilon        FTISVTTEILPVSMTKTSVDCTMYICGDSTECSNLLLQYGSFCTQLNRALTGIAVEQDKN
OU282717/B.1.429/Epsilon        FTISVTTEILPVSMTKTSVDCTMYICGDSTECSNLLLQYGSFCTQLNRALTGIAVEQDKN
OU235436/B.1.429/Epsilon        FTISVTTEILPVSMTKTSVDCTMYICGDSTECSNLLLQYGSFCTQLNRALTGIAVEQDKN
MZ687447/B.1.429/Epsilon        FTISVTTEILPVSMTKTSVDCTMYICGDSTECSNLLLQYGSFCTQLNRALTGIAVEQDKN
MZ363839/B.1.617.1/Kappa        FTISVTTEILPVSMTKTSVDCTMYICGDSTECSNLLLQYGSFCTQLNRALTGIAVEQDKN
MZ538882/B.1.617.1/Kappa        FTISVTTEILPVSMTKTSVDCTMYICGDSTECSNLLLQYGSFCTQLNRALTGIAVEQDKN
MZ724425/B.1.617.1/Kappa        FTISVTTEILPVSMTKTSVDCTMYICGDSTECSNLLLQYGSFCTQLNRALTGIAVEQDKN
MZ332524/B.1.617.1/Kappa        FTISVTTEILPVSMTKTSVDCTMYICGDSTECSNLLLQYGSFCTQLNRALTGIAVEQDKN
MZ332527/B.1.617.1/Kappa        FTISVTTEILPVSMTKTSVDCTMYICGDSTECSNLLLQYGSFCTQLNRALTGIAVEQDKN
LC633761/B.1.617.1/Kappa        FTISVTTEILPVSMTKTSVDCTMYICGDSTECSNLLLQYGSFCTQLNRALTGIAVEQDKN
MZ571142/B.1.617.1/Kappa        FTISVTTEILPVSMTKTSVDCTMYICGDSTECSNLLLQYGSFCTQLNRALTGIAVEQDKN
MZ562746/B.1.617.1/Kappa        FTISVTTEILPVSMTKTSVDCTMYICGDSTECSNLLLQYGSFCTQLNRALTGIAVEQDKN
OU053018/C.37/Lambda            FTISVTTEILPVSMTKTSVDCTMYICGDSTECSNLLLQYGSFCTQLNRALTGIAVEQDKN
OU268528/C.37/Lambda            FTISVTTEILPVSMTKTSVDCTMYICGDSTECSNLLLQYGSFCTQLNRALTGIAVEQDKN
OU100999/C.37/Lambda            FTISVTTEILPVSMTKTSVDCTMYICGDSTECSNLLLQYGSFCTQLNRALTGIAVEQDKN
MZ275295/C.37/Lambda            FTISVTTEILPVSMTKTSVDCTMYICGDSTECSNLLLQYGSFCTQLNRALTGIAVEQDKN
MZ275301/C.37/Lambda            FTISVTTEILPVSMTKTSVDCTMYICGDSTECSNLLLQYGSFCTQLNRALTGIAVEQDKN
MZ908711/C.37/Lambda            FTISVTTEILPVSMTKTSVDCTMYICGDSTECSNLLLQYGSFCTQLNRALTGIAVEQDKN
OU517038/C.37/Lambda            FTISVTTEILPVSMTKTSVDCTMYICGDSTECSNLLLQYGSFCTQLNRALTGIAVEQDKN
MZ496613/C.37/Lambda            FTISVTTEILPVSMTKTSVDCTMYICGDSTECSNLLLQYGSFCTQLNRALTGIAVEQDKN
OU466149/B.1.621/Mu             FTISVTTEILPVSMTKTSVDCTMYICGDSTECSNLLLQYGSFCTQLNRALTGIAVEQDKN
OU571573/B.1.621/Mu             FTISVTTEILPVSMTKTSVDCTMYICGDSTECSNLLLQYGSFCTQLNRALTGIAVEQDKN
OK025270/B.1.621/Mu             FTISVTTEILPVSMTKTSVDCTMYICGDSTECSNLLLQYGSFCTQLNRALTGIAVEQDKN
```

	780 . 790 . 800 . 810 . 820 . 830 .
MN908947/Wuhan-Hu-1	TQEVFAQVKQIYKTPPIKDFGGFNFSQILPDPSKPSKRSFIEDLLFNKVTLADAGFIKQY
BS001137/B.1.1.7/Alpha	TQEVFAQVKQIYKTPPIKDFGGFNFSQILPDPSKPSKRSFIEDLLFNKVTLADAGFIKQY
BS001138/B.1.1.7/Alpha	TQEVFAQVKQIYKTPPIKDFGGFNFSQILPDPSKPSKRSFIEDLLFNKVTLADAGFIKQY
BS001139/B.1.1.7/Alpha	TQEVFAQVKQIYKTPPIKDFGGFNFSQILPDPSKPSKRSFIEDLLFNKVTLADAGFIKQY
BS001140/B.1.1.7/Alpha	TQEVFAQVKQIYKTPPIKDFGGFNFSQILPDPSKPSKRSFIEDLLFNKVTLADAGFIKQY
MW913362/B.1.1.7/Alpha	TQEVFAQVKQIYKTPPIKDFGGFNFSQILPDPSKPSKRSFIEDLLFNKVTLADAGFIKQY
MZ266365/B.1.1.7/Alpha	TQEVFAQVKQIYKTPPIKDFGGFNFSQILPDPSKPSKRSFIEDLLFNKVTLADAGFIKQY
MZ350109/B.1.1.7/Alpha	TQEVFAQVKQIYKTPPIKDFGGFNFSQILPDPSKPSKRSFIEDLLFNKVTLADAGFIKQY
MZ888516/B.1.1.7/Alpha	TQEVFAQVKQIYKTPPIKDFGGFNFSQILPDPSKPSKRSFIEDLLFNKVTLADAGFIKQY
OU562607/B.1.1.7/Alpha	TQEVFAQVKQIYKTPPIKDFGGFNFSQILPDPSKPSKRSFIEDLLFNKVTLADAGFIKQY
OU514166/B.1.1.7/Alpha	TQEVFAQVKQIYKTPPIKDFGGFNFSQILPDPSKPSKRSFIEDLLFNKVTLADAGFIKQY
OU538849/B.1.1.7/Alpha	TQEVFAQVKQIYKTPPIKDFGGFNFSQILPDPSKPSKRSFIEDLLFNKVTLADAGFIKQY
MW981442/B.1.351/Beta	TQEVFAQVKQIYKTPPIKDFGGFNFSQILPDPSKPSKRSFIEDLLFNKVTLADAGFIKQY
MZ298839/B.1.351/Beta	TQEVFAQVKQIYKTPPIKDFGGFNFSQILPDPSKPSKRSFIEDLLFNKVTLADAGFIKQY
MZ913100/B.1.351/Beta	TQEVFAQVKQIYKTPPIKDFGGFNFSQILPDPSKPSKRSFIEDLLFNKVTLADAGFIKQY
MZ879365/B.1.351/Beta	TQEVFAQVKQIYKTPPIKDFGGFNFSQILPDPSKPSKRSFIEDLLFNKVTLADAGFIKQY
OU539352/B.1.351/Beta	TQEVFAQVKQIYKTPPIKDFGGFNFSQILPDPSKPSKRSFIEDLLFNKVTLADAGFIKQY
MZ202314/B.1.351/Beta	TQEVFAQVKQIYKTPPIKDFGGFNFSQILPDPSKPSKRSFIEDLLFNKVTLADAGFIKQY
MZ298840/B.1.351/Beta	TQEVFAQVKQIYKTPPIKDFGGFNFSQILPDPSKPSKRSFIEDLLFNKVTLADAGFIKQY
LC643036/B.1.617.2/Delta	TQEVFAQVKQIYKTPPIKDFGGFNFSQILPDPSKPSKRSFIEDLLFNKVTLADAGFIKQY
LC643044/B.1.617.2/Delta	TQEVFAQVKQIYKTPPIKDFGGFNFSQILPDPSKPSKRSFIEDLLFNKVTLADAGFIKQY
MZ853946/B.1.617.2/Delta	TQEVFAQVKQIYKTPPIKDFGGFNFSQILPDPSKPSKRSFIEDLLFNKVTLADAGFIKQY
MZ854386/B.1.617.2/Delta	TQEVFAQVKQIYKTPPIKDFGGFNFSQILPDPSKPSKRSFIEDLLFNKVTLADAGFIKQY
MZ888532/B.1.617.2/Delta	TQEVFAQVKQIYKTPPIKDFGGFNFSQILPDPSKPSKRSFIEDLLFNKVTLADAGFIKQY
MZ888533/B.1.617.2/Delta	TQEVFAQVKQIYKTPPIKDFGGFNFSQILPDPSKPSKRSFIEDLLFNKVTLADAGFIKQY
MZ896136/B.1.617.2/Delta	TQEVFAQVKQIYKTPPIKDFGGFNFSQILPDPSKPSKRSFIEDLLFNKVTLADAGFIKQY
OU539261/B.1.617.2/Delta	TQEVFAQVKQIYKTPPIKDFGGFNFSQILPDPSKPSKRSFIEDLLFNKVTLADAGFIKQY
MZ854389/B.1.617.2/Delta	TQEVFAQVKQIYKTPPIKDFGGFNFSQILPDPSKPSKRSFIEDLLFNKVTLADAGFIKQY
OU539784/P.1/Gamma	TQEVFAQVKQIYKTPPIKDFGGFNFSQILPDPSKPSKRSFIEDLLFNKVTLADAGFIKQY
OU539830/P.1/Gamma	TQEVFAQVKQIYKTPPIKDFGGFNFSQILPDPSKPSKRSFIEDLLFNKVTLADAGFIKQY
OU539894/P.1/Gamma	TQEVFAQVKQIYKTPPIKDFGGFNFSQILPDPSKPSKRSFIEDLLFNKVTLADAGFIKQY
MZ477748/P.1/Gamma	TQEVFAQVKQIYKTPPIKDFGGFNFSQILPDPSKPSKRSFIEDLLFNKVTLADAGFIKQY
MZ477746/P.1/Gamma	TQEVFAQVKQIYKTPPIKDFGGFNFSQILPDPSKPSKRSFIEDLLFNKVTLADAGFIKQY
MW938104/P.1/Gamma	TQEVFAQVKQIYKTPPIKDFGGFNFSQILPDPSKPSKRSFIEDLLFNKVTLADAGFIKQY
MZ896179/P.1/Gamma	TQEVFAQVKQIYKTPPIKDFGGFNFSQILPDPSKPSKRSFIEDLLFNKVTLADAGFIKQY
MZ896359/P.1/Gamma	TQEVFAQVKQIYKTPPIKDFGGFNFSQILPDPSKPSKRSFIEDLLFNKVTLADAGFIKQY
OL677199/BA.1/Omicron	TQEVFAQVKQIYKTPPIKYFGGFNFSQILPDPSKPSKRSFIEDLLFNKVTLADAGFIKQY
OL672836/BA.1/Omicron	TQEVFAQVKQIYKTPPIKYFGGFNFSQILPDPSKPSKRSFIEDLLFNKVTLADAGFIKQY
OW714547.1/BA.1/Omicron	TQEVFAQVKQIYKTPPIKYFGGFNFSQILPDPSKPSKRSFIEDLLFNKVTLADAGFIKQY
OW714198.1/BA.1/Omicron	TQEVFAQVKQIYKTPPIKYFGGFNFSQILPDPSKPSKRSFIEDLLFNKVTLADAGFIKQY
OW303180.1/BA.1/Omicron	TQEVFAQVKQIYKTPPIKYFGGFNFSQILPDPSKPSKRSFIEDLLFNKVTLADAGFIKQY
EPI_ISL_7747552/BA.2/Omicron	TQEVFAQVKQIYKTPPIKYFGGFNFSQILPDPSKPSKRSFIEDLLFNKVTLADAGFIKQY
EPI_ISL_7747545/BA.2/Omicron	TQEVFAQVKQIYKTPPIKYFGGFNFSQILPDPSKPSKRSFIEDLLFNKVTLADAGFIKQY
EPI_ISL_7747536/BA.2/Omicron	TQEVFAQVKQIYKTPPIKYFGGFNFSQILPDPSKPSKRSFIEDLLFNKVTLADAGFIKQY
EPI_ISL_7747535/BA.2/Omicron	TQEVFAQVKQIYKTPPIKYFGGFNFSQILPDPSKPSKRSFIEDLLFNKVTLADAGFIKQY
EPI_ISL_6795834/BA.2/Omicron	TQEVFAQVKQIYKTPPIKYFGGFNFSQILPDPSKPSKRSFIEDLLFNKVTLADAGFIKQY
EPI_ISL_7718520/BA.2/Omicron	TQEVFAQVKQIYKTPPIKYFGGFNFSQILPDPSKPSKRSFIEDLLFNKVTLADAGFIKQY
EPI_ISL_7580387/BA.2/Omicron	TQEVFAQVKQIYKTPPIKYFGGFNFSQILPDPSKPSKRSFIEDLLFNKVTLADAGFIKQY
EPI_ISL_7701122/BA.2/Omicron	TQEVFAQVKQIYKTPPIKYFGGFNFSQILPDPSKPSKRSFIEDLLFNKVTLADAGFIKQY
EPI_ISL_7190366/BA.2/Omicron	TQEVFAQVKQIYKTPPIKYFGGFNFSQILPDPSKPSKRSFIEDLLFNKVTLADAGFIKQY
EPI_ISL_7644798/BA.2/Omicron	TQEVFAQVKQIYKTPPIKYFGGFNFSQILPDPSKPSKRSFIEDLLFNKVTLADAGFIKQY
EPI_ISL_7740766/BA.3/Omicron	TQEVFAQVKQIYKTPPIKYFGGFNFSQILPDPSKPSKRSFIEDLLFNKVTLADAGFIKQY
EPI_ISL_7605589/BA.3/Omicron	TQEVFAQVKQIYKTPPIKYFGGFNFSQILPDPSKPSKRSFIEDLLFNKVTLADAGFIKQY
ON296657.1/BA.4/Omicron	TQEVFAQVKQIYKTPPIKYFGGFNFSQILPDPSKPSKRSFIEDLLFNKVTLADAGFIKQY
ON331287.1/BA.4/Omicron	TQEVFAQVKQIYKTPPIKYFGGFNFSQILPDPSKPSKRSFIEDLLFNKVTLADAGFIKQY
ON321245.1/BA.4/Omicron	TQEVFAQVKQIYKTPPIKYFGGFNFSQILPDPSKPSKRSFIEDLLFNKVTLADAGFIKQY
ON337003.1/BA.4/Omicron	TQEVFAQVKQIYKTPPIKYFGGFNFSQILPDPSKPSKRSFIEDLLFNKVTLADAGFIKQY
ON270681.1/BA.4/Omicron	TQEVFAQVKQIYKTPPIKYFGGFNFSQILPDPSKPSKRSFIEDLLFNKVTLADAGFIKQY
ON373214.1/BA.4/Omicron	TQEVFAQVKQIYKTPPIKYFGGFNFSQILPDPSKPSKRSFIEDLLFNKVTLADAGFIKQY
ON393428.1/BA.5/Omicron	TQEVFAQVKQIYKTPPIKYFGGFNFSQILPDPSKPSKRSFIEDLLFNKVTLADAGFIKQY
ON393156.1/BA.5/Omicron	TQEVFAQVKQIYKTPPIKYFGGFNFSQILPDPSKPSKRSFIEDLLFNKVTLADAGFIKQY
ON348053.1/BA.5/Omicron	TQEVFAQVKQIYKTPPIKYFGGFNFSQILPDPSKPSKRSFIEDLLFNKVTLADAGFIKQY
ON323731.1/BA.5/Omicron	TQEVFAQVKQIYKTPPIKYFGGFNFSQILPDPSKPSKRSFIEDLLFNKVTLADAGFIKQY
ON251467.1/BA.5/Omicron	TQEVFAQVKQIYKTPPIKYFGGFNFSQILPDPSKPSKRSFIEDLLFNKVTLADAGFIKQY
ON250431.1/BA.5/Omicron	TQEVFAQVKQIYKTPPIKYFGGFNFSQILPDPSKPSKRSFIEDLLFNKVTLADAGFIKQY
MZ911221/B.1.427/Epsilon	TQEVFAQVKQIYKTPPIKDFGGFNFSQILPDPSKPSKRSFIEDLLFNKVTLADAGFIKQY
MZ375858/B.1.427/Epsilon	TQEVFAQVKQIYKTPPIKDFGGFNFSQILPDPSKPSKRSFIEDLLFNKVTLADAGFIKQY
MZ375854/B.1.427/Epsilon	TQEVFAQVKQIYKTPPIKDFGGFNFSQILPDPSKPSKRSFIEDLLFNKVTLADAGFIKQY
MZ943834/B.1.429/Epsilon	TQEVFAQVKQIYKTPPIKDFGGFNFSQILPDPSKPSKRSFIEDLLFNKVTLADAGFIKQY
MZ277390/B.1.429/Epsilon	TQEVFAQVKQIYKTPPIKDFGGFNFSQILPDPSKPSKRSFIEDLLFNKVTLADAGFIKQY
OU282717/B.1.429/Epsilon	TQEVFAQVKQIYKTPPIKNFGGFNFSQILPDPSKPSKRSFIEDLLFNKVTLADAGFIKQY
OU235436/B.1.429/Epsilon	TQEVFAQVKQIYKTPPIKDFGGFNFSQILPDPSKPSKRSFIEDLLFNKVTLADAGFIKQY
MZ687447/B.1.429/Epsilon	TQEVFAQVKQIYKTPPIKDFGGFNFSQILPDPSKPSKRSFIEDLLFNKVTLADAGFIKQY
MZ363839/B.1.617.1/Kappa	TQEVFAQVKQIYKTPPIKDFGGFNFSQILPDPSKPSKRSFIEDLLFNKVTLADAGFIKQY
MZ538882/B.1.617.1/Kappa	TQEVFAQVKQIYKTPPIKDFGGFNFSQILPDPSKPSKRSFIEDLLFNKVTLADAGFIKQY
MZ724425/B.1.617.1/Kappa	TQEVFAQVKQIYKTPPIKDFGGFNFSQILPDPSKPSKRSFIEDLLFNKVTLADAGFIKQY
MZ332524/B.1.617.1/Kappa	TQEVFAQVKQIYKTPPIKDFGGFNFSQILPDPSKPSKRSFIEDLLFNKVTLADAGFIKQY
MZ332527/B.1.617.1/Kappa	TQEVFAQVKQIYKTPPIKDFGGFNFSQILPDPSKPSKRSFIEDLLFNKVTLADAGFIKQY
LC633761/B.1.617.1/Kappa	TQEVFAQVKQIYKTPPIKDFGGFNFSQILPDPSKPSKRSFIEDLLFNKVTLADAGFIKQY
MZ571142/B.1.617.1/Kappa	TQEVFAQVKQIYKTPPIKDFGGFNFSQILPDPSKPSKRSFIEDLLFNKVTLADAGFIKQY
MZ562746/B.1.617.1/Kappa	TQEVFAQVKQIYKTPPIKDFGGFNFSQILPDPSKPSKRSFIEDLLFNKVTLADAGFIKQY
OU053018/C.37/Lambda	TQEVFAQVKQIYKTPPIKDFGGFNFSQILPDPSKPSKRSFIEDLLFNKVTLADAGFIKQY
OU268528/C.37/Lambda	TQEVFAQVKQIYKTPPIKDFGGFNFSQILPDPSKPSKRSFIEDLLFNKVTLADAGFIKQY
OU100999/C.37/Lambda	TQEVFAQVKQIYKTPPIKDFGGFNFSQILPDPSKPSKRSFIEDLLFNKVTLADAGFIKQY
MZ275295/C.37/Lambda	TQEVFAQVKQIYKTPPIKDFGGFNFSQILPDPSKPSKRSFIEDLLFNKVTLADAGFIKQY
MZ275301/C.37/Lambda	TQEVFAQVKQIYKTPPIKDFGGFNFSQILPDPSKPSKRSFIEDLLFNKVTLADAGFIKQY
MZ908711/C.37/Lambda	TQEVFAQVKQIYKTPPIKDFGGFNFSQILPDPSKPSKRSFIEDLLFNKVTLADAGFIKQY
OU517038/C.37/Lambda	TQEVFAQVKQIYKTPPIKDFGGFNFSQILPDPSKPSKRSFIEDLLFNKVTLADAGFIKQY
MZ496613/C.37/Lambda	TQEVFAQVKQIYKTPPIKDFGGFNFSQILPDPSKPSKRSFIEDLLFNKVTLADAGFIKQY
OU466149/B.1.621/Mu	TQEVFAQVKQIYKTPPIKDFGGFNFSQILPDPSKPSKRSFIEDLLFNKVTLADAGFIKQY
OU571573/B.1.621/Mu	TQEVFAQVKQIYKTPPIKDFGGFNFSQILPDPSKPSKRSFIEDLLFNKVTLADAGFIKQY
OK025270/B.1.621/Mu	TQEVFAQVKQIYKTPPIKDFGGFNFSQILPDPSKPSKRSFIEDLLFNKVTLADAGFIKQY

```
                                  840       850       860       870       880       890
MN908947/Wuhan-Hu-1               GDCLGDIAARDLICAQKFNGLTVLPPLLTDEMIAQYTSALLAGTITSGWTFGAGAALQIP
BS001137/B.1.1.7/Alpha            GDCLGDIAARDLICAQKFNGLTVLPPLLTDEMIAQYTSALLAGTITSGWTFGAGAALQIP
BS001138/B.1.1.7/Alpha            GDCLGDIAARDLICAQKFNGLTVLPPLLTDEMIAQYTSALLAGTITSGWTFGAGAALQIP
BS001139/B.1.1.7/Alpha            GDCLGDIAARDLICAQKFNGLTVLPPLLTDEMIAQYTSALLAGTITSGWTFGAGAALQIP
BS001140/B.1.1.7/Alpha            GDCLGDIAARDLICAQKFNGLTVLPPLLTDEMIAQYTSALLAGTITSGWTFGAGAALQIP
MW913362/B.1.1.7/Alpha            GDCLGDIAARDLICAQKFNGLTVLPPLLTDEMIAQYTSALLAGTITSGWTFGAGAALQIP
MZ266365/B.1.1.7/Alpha            GDCLGDIAARDLICAQKFNGLTVLPPLLTDEMIAQYTSALLAGTITSGWTFGAGAALQIP
MZ350109/B.1.1.7/Alpha            GDCLGDIAARDLICAQKFNGLTVLPPLLTDEMIAQYTSALLAGTITSGWTFGAGAALQIP
MZ888516/B.1.1.7/Alpha            GDCLGDIAARDLICAQKFNGLTVLPPLLTDEMIAQYTSALLAGTITSGWTFGAGAALQIP
OU562607/B.1.1.7/Alpha            GDCLGDIAARDLICAQKFNGLTVLPPLLTDEMIAQYTSALLAGTITSGWTFGAGAALQIP
OU514166/B.1.1.7/Alpha            GDCLGDIAARDLICAQKFNGLTVLPPLLTDEMIAQYTSALLAGTITSGWTFGAGAALQIP
OU538849/B.1.1.7/Alpha            GDCLGDIAARDLICAQKFNGLTVLPPLLTDEMIAQYTSALLAGTITSGWTFGAGAALQIP
MW981442/B.1.351/Beta             GDCLGDIAARDLICAQKFNGLTVLPPLLTDEMIAQYTSALLAGTITSGWTFGAGAALQIP
MZ298839/B.1.351/Beta             GDCLGDIAARDLICAQKFNGLTVLPPLLTDEMIAQYTSALLAGTITSGWTFGAGAALQIP
MZ913100/B.1.351/Beta             GDCLGDIAARDLICAQKFNGLTVLPPLLTDEMIAQYTSALLAGTITSGWTFGAGAALQIP
MZ879365/B.1.351/Beta             GDCLGDIAARDLICAQKFNGLTVLPPLLTDEMIAQYTSALLAGTITSGWTFGAGAALQIP
OU539352/B.1.351/Beta             GDCLGDIAARDLICAQKFNGLTVLPPLLTDEMIAQYTSALLAGTITSGWTFGAGAALQIP
MZ202314/B.1.351/Beta             GDCLGDIAARDLICAQKFNGLTVLPPLLTDEMIAQYTSALLAGTITSGWTFGAGAALQIP
MZ298840/B.1.351/Beta             GDCLGDIAARDLICAQKFNGLTVLPPLLTDEMIAQYTSALLAGTITSGWTFGAGAALQIP
LC643036/B.1.617.2/Delta          GDCLGDIAARDLICAQKFNGLTVLPPLLTDEMIAQYTSALLAGTITSGWTFGAGAALQIP
LC643044/B.1.617.2/Delta          GDCLGDIAARDLICAQKFNGLTVLPPLLTDEMIAQYTSALLAGTITSGWTFGAGAALQIP
MZ853946/B.1.617.2/Delta          GDCLGDIAARDLICAQKFNGLTVLPPLLTDEMIAQYTSALLAGTITSGWTFGAGAALQIP
MZ854386/B.1.617.2/Delta          GDCLGDIAARDLICAQKFNGLTVLPPLLTDEMIAQYTSALLAGTITSGWTFGAGAALQIP
MZ888532/B.1.617.2/Delta          GDCLGDIAARDLICAQKFNGLTVLPPLLTDEMIAQYTSALLAGTITSGWTFGAGAALQIP
MZ888533/B.1.617.2/Delta          GDCLGDIAARDLICAQKFNGLTVLPPLLTDEMIAQYTSALLAGTITSGWTFGAGAALQIP
MZ896136/B.1.617.2/Delta          GDCLGDIAARDLICAQKFNGLTVLPPLLTDEMIAQYTSALLAGTITSGWTFGAGAALQIP
OU539261/B.1.617.2/Delta          GDCLGDIAARDLICAQKFNGLTVLPPLLTDEMIAQYTSALLAGTITSGWTFGAGAALQIP
MZ854389/B.1.617.2/Delta          GDCLGDIAARDLICAQKFNGLTVLPPLLTDEMIAQYTSALLAGTITSGWTFGAGAALQIP
OU539784/P.1/Gamma                GDCLGDIAARDLICAQKFNGLTVLPPLLTDEMIAQYTSALLAGTITSGWTFGAGAALQIP
OU539830/P.1/Gamma                GDCLGDIAARDLICAQKFNGLTVLPPLLTDEMIAQYTSALLAGTITSGWTFGAGAALQIP
OU539894/P.1/Gamma                GDCLGDIAARDLICAQKFNGLTVLPPLLTDEMIAQYTSALLAGTITSGWTFGAGAALQIP
MZ477748/P.1/Gamma                GDCLGDIAARDLICAQKFNGLTVLPPLLTDEMIAQYTSALLAGTITSGWTFGAGAALQIP
MZ477746/P.1/Gamma                GDCLGDIAARDLICAQKFNGLTVLPPLLTDEMIAQYTSALLAGTITSGWTFGAGAALQIP
MW938104/P.1/Gamma                GDCLGDIAARDLICAQKFNGLTVLPPLLTDEMIAQYTSALLAGTITSGWTFGAGAALQIP
MZ896179/P.1/Gamma                GDCLGDIAARDLICAQKFNGLTVLPPLLTDEMIAQYTSALLAGTITSGWTFGAGAALQIP
MZ896359/P.1/Gamma                GDCLGDIAARDLICAQKFNGLTVLPPLLTDEMIAQYTSALLAGTITSGWTFGAGAALQIP
OL677199/BA.1/Omicron             GDCLGDIAARDLICAQKFKGLTVLPPLLTDEMIAQYTSALLAGTITSGWTFGAGAALQIP
OL672836/BA.1/Omicron             GDCLGDIAARDLICAQKFKGLTVLPPLLTDEMIAQYTSALLAGTITSGWTFGAGAALQIP
OW714547.1/BA.1/Omicron           GDCLGDIAARDLICAQKFKGLTVLPPLLTDEMIAQYTSALLAGTITSGWTFGAGAALQIP
OW714198.1/BA.1/Omicron           GDCLGDIAARDLICAQKFKGLTVLPPLLTDEMIAQYTSALLAGTITSGWTFGAGAALQIP
OW303180.1/BA.1/Omicron           GDCLGDIAARDLICAQKFKGLTVLPPLLTDEMIAQYTSALLAGTITSGWTFGAGAALQIP
EPI_ISL_7747552/BA.2/Omicron      GDCLGDIAARDLICAQKFNGLTVLPPLLTDEMIAQYTSALLAGTITSGWTFGAGAALQIP
EPI_ISL_7747545/BA.2/Omicron      GDCLGDIAARDLICAQKFNGLTVLPPLLTDEMIAQYTSALLAGTITSGWTFGAGAALQIP
EPI_ISL_7747536/BA.2/Omicron      GDCLGDIAARDLICAQKFNGLTVLPPLLTDEMIAQYTSALLAGTITSGWTFGAGAALQIP
EPI_ISL_7747535/BA.2/Omicron      GDCLGDIAARDLICAQKFNGLTVLPPLLTDEMIAQYTSALLAGTITSGWTFGAGAALQIP
EPI_ISL_6795834/BA.2/Omicron      GDCLGDIAARDLICAQKFNGLTVLPPLLTDEMIAQYTSALLAGTITSGWTFGAGAALQIP
EPI_ISL_7718520/BA.2/Omicron      GDCLGDIAARDLICAQKFNGLTVLPPLLTDEMIAQYTSALLAGTITSGWTFGAGAALQIP
EPI_ISL_7580387/BA.2/Omicron      GDCLGDIAARDLICAQKFNGLTVLPPLLTDEMIAQYTSALLAGTITSGWTFGAGAALQIP
EPI_ISL_7701122/BA.2/Omicron      GDCLGDIAARDLICAQKFNGLTVLPPLLTDEMIAQYTSALLAGTITSGWTFGAGAALQIP
EPI_ISL_7190366/BA.2/Omicron      GDCLGDIAARDLICAQKFNGLTVLPPLLTDEMIAQYTSALLAGTITSGWTFGAGAALQIP
EPI_ISL_7644798/BA.2/Omicron      GDCLGDIAARDLICAQKFNGLTVLPPLLTDEMIAQYTSALLAGTITSGWTFGAGAALQIP
EPI_ISL_7740766/BA.3/Omicron      GDCLGDIAARDLICAQKFNGLTVLPPLLTDEMIAQYTSALLAGTITSGWTFGAGAALQIP
EPI_ISL_7605589/BA.3/Omicron      GDCLGDIAARDLICAQKFNGLTVLPPLLTDEMIAQYTSALLAGTITSGWTFGAGAALQIP
ON296657.1/BA.4/Omicron           GDCLGDIAARDLICAQKFNGLTVLPPLLTDEMIAQYTSALLAGTITSGWTFGAGAALQIP
ON331287.1/BA.4/Omicron           GDCLGDIAARDLICAQKFNGLTVLPPLLTDEMIAQYTSALLAGTITSGWTFGAGAALQIP
ON321245.1/BA.4/Omicron           GDCLGDIAARDLICAQKFNGLTVLPPLLTDEMIAQYTSALLAGTITSGWTFGAGAALQIP
ON337003.1/BA.4/Omicron           GDCLGDIAARDLICAQKFNGLTVLPPLLTDEMIAQYTSALLAGTITSGWTFGAGAALQIP
ON270681.1/BA.4/Omicron           GDCLGDIAARDLICAQKFNGLTVLPPLLTDEMIAQYTSALLAGTITSGWTFGAGAALQIP
ON373214.1/BA.4/Omicron           GDCLGDIAARDLICAQKFNGLTVLPPLLTDEMIAQYTSALLAGTITSGWTFGAGAALQIP
ON393428.1/BA.5/Omicron           GDCLGDIAARDLICAQKFNGLTVLPPLLTDEMIAQYTSALLAGTITSGWTFGAGAALQIP
ON393156.1/BA.5/Omicron           GDCLGDIAARDLICAQKFNGLTVLPPLLTDEMIAQYTSALLAGTITSGWTFGAGAALQIP
ON348053.1/BA.5/Omicron           GDCLGDIAARDLICAQKFNGLTVLPPLLTDEMIAQYTSALLAGTITSGWTFGAGAALQIP
ON323731.1/BA.5/Omicron           GDCLGDIAARDLICAQKFNGLTVLPPLLTDEMIAQYTSALLAGTITSGWTFGAGAALQIP
ON251467.1/BA.5/Omicron           GDCLGDIAARDLICAQKFNGLTVLPPLLTDEMIAQYTSALLAGTITSGWTFGAGAALQIP
ON250431.1/BA.5/Omicron           GDCLGDIAARDLICAQKFNGLTVLPPLLTDEMIAQYTSALLAGTITSGWTFGAGAALQIP
MZ911221/B.1.427/Epsilon          GDCLGDIAARDLICAQKFNGLTVLPPLLTDEMIAQYTSALLAGTITSGWTFGAGAALQIP
MZ375858/B.1.427/Epsilon          GDCLGDIAARDLICAQKFNGLTVLPPLLTDEMIAQYTSALLAGTITSGWTFGAGAALQIP
MZ375854/B.1.427/Epsilon          GDCLGDIAARDLICAQKFNGLTVLPPLLTDEMIAQYTSALLAGTITSGWTFGAGAALQIP
MZ943834/B.1.429/Epsilon          GDCLGDIAARDLICAQKFNGLTVLPPLLTDEMIAQYTSALLAGTITSGWTFGAGAALQIP
MZ277390/B.1.429/Epsilon          GDCLGDIAARDLICAQKFNGLTVLPPLLTDEMIAQYTSALLAGTITSGWTFGAGAALQIP
OU282717/B.1.429/Epsilon          GDCLGDIAARDLICAQKFNGLTVLPPLLTDEMIAQYTSALLAGTITSGWTFGAGAALQIP
OU235436/B.1.429/Epsilon          GDCLGDIAARDLICAQKFNGLTVLPPLLTDEMIAQYTSALLAGTITSGWTFGAGAALQIP
MZ687447/B.1.429/Epsilon          GDCLGDIAARDLICAQKFNGLTVLPPLLTDEMIAQYTSALLAGTITSGWTFGAGAALQIP
MZ363839/B.1.617.1/Kappa          GDCLGDIAARDLICAQKFNGLTVLPPLLTDEMIAQYTSALLAGTITSGWTFGAGAALQIP
MZ538882/B.1.617.1/Kappa          GDCLGDIAARDLICAQKFNGLTVLPPLLTDEMIAQYTSALLAGTITSGWTFGAGAALQIP
MZ724425/B.1.617.1/Kappa          GDCLGDIAARDLICAQKFNGLTVLPPLLTDEMIAQYTSALLAGTITSGWTFGAGAALQIP
MZ332524/B.1.617.1/Kappa          GDCLGDIAARDLICAQKFNGLTVLPPLLTDEMIAQYTSALLAGTITSGWTFGAGAALQIP
MZ332527/B.1.617.1/Kappa          GDCLGDIAARDLICAQKFNGLTVLPPLLTDEMIAQYTSALLAGTITSGWTFGAGAALQIP
LC633761/B.1.617.1/Kappa          GDCLGDIAARDLICAQKFNGLTVLPPLLTDEMIAQYTSALLAGTITSGWTFGAGAALQIP
MZ571142/B.1.617.1/Kappa          GDCLGDIAARDLICAQKFNGLTVLPPLLTDEMIAQYTSALLAGTITSGWTFGAGAALQIP
MZ562746/B.1.617.1/Kappa          GDCLGDIAARDLICAQKFNGLTVLPPLLTDEMIAQYTSALLAGTITSGWTFGAGAALQIP
OU053018/C.37/Lambda              GDCLGDIAARDLICAQKFNGLNVLPPLLTDEMIAQYTSALLAGTITSGWTFGAGAALQIP
OU268528/C.37/Lambda              GDCLGDIAARDLICAQKFNGLNVLPPLLTDEMIAQYTSALLAGTITSGWTFGAGAALQIP
OU100999/C.37/Lambda              GDCLGDIAARDLICAQKFNGLNVLPPLLTDEMIAQYTSALLAGTITSGWTFGAGAALQIP
MZ275295/C.37/Lambda              GDCLADIAARDLICAQKFNGLNVLPPLLTDEMIAQYTSALLAGTITSGWTFGAGAALQIP
MZ275301/C.37/Lambda              GDCLGDIAARDLICAQKFNGLNVLPPLLTDEMIAQYTSALLAGTITSGWTFGAGAALQIP
MZ908711/C.37/Lambda              GDCLGDIAARDLICAQKFNGLNVLPPLLTDEMIAQYTSALLAGTITSGWTFGAGAALQIP
OU517038/C.37/Lambda              GDCLGDIAARDLICAQKFNGLNVLPPLLTDEMIAQYTSALLAGTITSGWTFGAGAALQIP
MZ496613/C.37/Lambda              GDCLGDIAARDLICAQKFNGLNVLPPLLTDEMIAQYTSALLAGTITSGWTFGAGAALQIP
OU466149/B.1.621/Mu               GDCLGDIAARDLICAQKFNGLTVLPPLLTDEMIAQYTSALLAGTITSGWTFGAGAALQIP
OU571573/B.1.621/Mu               GDCLGDIAARDLICAQKFNGLTVLPPLLTDEMIAQYTSALLAGTITSGWTFGAGAALQIP
OK025270/B.1.621/Mu               GDCLGDIAARDLICAQKFNGLTVLPPLLTDEMIAQYTSALLAGTITSGWTFGAGAALQIP
```

	900 910 920 930 940 950
MN908947/Wuhan-Hu-1	FAMQMAYRFNGIGVTQNVLYENQKLIANQFNSAIGKIQDSLSSTASALGKLQDVVNQNAQ
BS001137/B.1.1.7/Alpha	FAMQMAYRFNGIGVTQNVLYENQKLIANQFNSAIGKIQDSLSSTASALGKLQDVVNQNAQ
BS001138/B.1.1.7/Alpha	FAMQMAYRFNGIGVTQNVLYENQKLIANQFNSAIGKIQDSLSSTASALGKLQDVVNQNAQ
BS001139/B.1.1.7/Alpha	FAMQMAYRFNGIGVTQNVLYENQKLIANQFNSAIGKIQDSLSSTASALGKLQDVVNQNAQ
BS001140/B.1.1.7/Alpha	FAMQMAYRFNGIGVTQNVLYENQKLIANQFNSAIGKIQDSLSSTASALGKLQDVVNQNAQ
MW913362/B.1.1.7/Alpha	FAMQMAYRFNGIGVTQNVLYENQKLIANQFNSAIGKIQDSLSSTASALGKLQDVVNQNAQ
MZ266365/B.1.1.7/Alpha	FAMQMAYRFNGIGVTQNVLYENQKLIANQFNSAIGKIQDSLSSTASALGKLQDVVNQNAQ
MZ350109/B.1.1.7/Alpha	FAMQMAYRFNGIGVTQNVLYENQKLIANQFNSAIGKIQDSLSSTASALGKLQDVVNQNAQ
MZ888516/B.1.1.7/Alpha	FAMQMAYRFNGIGVTQNVLYENQKLIANQFNSAIGKIQDSLSSTASALGKLQDVVNQNAQ
OU562607/B.1.1.7/Alpha	FAMQMAYRFNGIGVTQNVLYENQKLIANQFNSAIGKIQDSLSSTASALGKLQDVVNQNAQ
OU514166/B.1.1.7/Alpha	FAMQMAYRFNGIGVTQNVLYENQKLIANQFNSAIGKIQDSLSSTASALGKLQDVVNQNAQ
OU538849/B.1.1.7/Alpha	FAMQMAYRFNGIGVTQNVLYENQKLIANQFNSAIGKIQDSLSSTASALGKLQDVVNQNAQ
MW981442/B.1.351/Beta	FAMQMAYRFNGIGVTQNVLYENQKLIANQFNSAIGKIQDSLSSTASALGKLQDVVNQNAQ
MZ298839/B.1.351/Beta	FAMQMAYRFNGIGVTQNVLYENQKLIANQFNSAIGKIQDSLSSTASALGKLQDVVNQNAQ
MZ913100/B.1.351/Beta	FAMQMAYRFNGIGVTQNVLYENQKLIANQFNSAIGKIQDSLSSTASALGKLQDVVNQNAQ
MZ879365/B.1.351/Beta	FAMQMAYRFNGIGVTQNVLYENQKLIANQFNSAIGKIQDSLSSTASALGKLQDVVNQNAQ
OU539352/B.1.351/Beta	FAMQMAYRFNGIGVTQNVLYENQKLIANQFNSAIGKIQDSLSSTASALGKLQDVVNQNAQ
MZ202314/B.1.351/Beta	FAMQMAYRFNGIGVTQNVLYENQKLIANQFNSAIGKIQDSLSSTASALGKLQDVVNQNAQ
MZ298840/B.1.351/Beta	FAMQMAYRFNGIGVTQNVLYENQKLIANQFNSAIGKIQDSLSSTASALGKLQDVVNQNAQ
LC643036/B.1.617.2/Delta	FAMQMAYRFNGIGVTQNVLYENQKLIANQFNSAIGKIQDSLSSTASALGKLQNVVNQNAQ
LC643044/B.1.617.2/Delta	FAMQMAYRFNGIGVTQNVLYENQKLIANQFNSAIGKIQDSLSSTASALGKLQNVVNQNAQ
MZ853946/B.1.617.2/Delta	FAMQMAYRFNGIGVTQNVLYENQKLIANQFNSAIGKIQDSLSSTASALGKLQNVVNQNAQ
MZ854386/B.1.617.2/Delta	FAMQMAYRFNGIGVTQNVLYENQKLIANQFNSAIGKIQDSLSSTASALGKLQNVVNQNAQ
MZ888532/B.1.617.2/Delta	FAMQMAYRFNGIGVTQNVLYENQKLIANQFNSAIGKIQDSLSSTASALGKLQNVVNQNAQ
MZ888533/B.1.617.2/Delta	FAMQMAYRFNGIGVTQNVLYENQKLIANQFNSAIGKIQDSLSSTASALGKLQNVVNQNAQ
MZ896136/B.1.617.2/Delta	FAMQMAYRFNGIGVTQNVLYENQKLIANQFNSAIGKIQDSLSSTASALGKLQNVVNQNAQ
OU539261/B.1.617.2/Delta	FAMQMAYRFNGIGVTQNVLYENQKLIANQFNSAIGKIQDSLSSTASALGKLQNVVNQNAQ
MZ854389/B.1.617.2/Delta	FAMQMAYRFNGIGVTQNVLYENQKLIANQFNSAIGKIQDSLSSTASALGKLQNVVNQNAQ
OU539784/P.1/Gamma	FAMQMAYRFNGIGVTQNVLYENQKLIANQFNSAIGKIQDSLSSTASALGKLQDVVNQNAQ
OU539830/P.1/Gamma	FAMQMAYRFNGIGVTQNVLYENQKLIANQFNSAIGKIQDSLSSTASALGKLQDVVNQNAQ
OU539894/P.1/Gamma	FAMQMAYRFNGIGVTQNVLYENQKLIANQFNSAIGKIQDSLSSTASALGKLQDVVNQNAQ
MZ477748/P.1/Gamma	FAMQMAYRFNGIGVTQNVLYENQKLIANQFNSAIGKIQDSLSSTASALGKLQDVVNQNAQ
MZ477746/P.1/Gamma	FAMQMAYRFNGIGVTQNVLYENQKLIANQFNSAIGKIQDSLSSTASALGKLQDVVNQNAQ
MW938104/P.1/Gamma	FAMQMAYRFNGIGVTQNVLYENQKLIANQFNSAIGKIQDSLSSTASALGKLQDVVNQNAQ
MZ896179/P.1/Gamma	FAMQMAYRFNGIGVTQNVLYENQKLIANQFNSAIGKIQDSLSSTASALRKLQDVVNQNAQ
MZ896359/P.1/Gamma	FAMQMAYRFNGIGVTQNVLYENQKLIANQFNSAIGKIQDSLSSTASALGKLQDVVNQNAQ
OL677199/BA.1/Omicron	FAMQMAYRFNGIGVTQNVLYENQKLIANQFNSAIGKIQDSLSSTASALGKLQDVVNHNAQ
OL672836/BA.1/Omicron	FAMQMAYRFNGIGVTQNVLYENQKLIANQFNSAIGKIQDSLSSTASALGKLQDVVNHNAQ
OW714547.1/BA.1/Omicron	FAMQMAYRFNGIGVTQNVLYENQKLIANQFNSAIGKIQDSLSSTASALGKLQDVVNHNAQ
OW714198.1/BA.1/Omicron	FAMQMAYRFNGIGVTQNVLYENQKLIANQFNSAIGKIQDSLSSTASALGKLQDVVNHNAQ
OW303180.1/BA.1/Omicron	FAMQMAYRFNGIGVTQNVLYENQKLIANQFNSAIGKIQDSLSSTASALGKLQDVVNHNAQ
EPI_ISL_7747552/BA.2/Omicron	FAMQMAYRFNGIGVTQNVLYENQKLIANQFNSAIGKIQDSLSSTASALGKLQDVVNHNAQ
EPI_ISL_7747545/BA.2/Omicron	FAMQMAYRFNGIGVTQNVLYENQKLIANQFNSAIGKIQDSLSSTASALGKLQDVVNHNAQ
EPI_ISL_7747536/BA.2/Omicron	FAMQMAYRFNGIGVTQNVLYENQKLIANQFNSAIGKIQDSLSSTASALGKLQDVVNHNAQ
EPI_ISL_7747535/BA.2/Omicron	FAMQMAYRFNGIGVTQNVLYENQKLIANQFNSAIGKIQDSLSSTASALGKLQDVVNHNAQ
EPI_ISL_6795834/BA.2/Omicron	FAMQMAYRFNGIGVTQNVLYENQKLIANQFNSAIGKIQDSLSSTASALGKLQDVVNHNAQ
EPI_ISL_7718520/BA.2/Omicron	FAMQMAYRFNGIGVTQNVLYENQKLIANQFNSAIGKIQDSLSSTASALGKLQDVVNHNAQ
EPI_ISL_7580387/BA.2/Omicron	FAMQMAYRFNGIGVTQNVLYENQKLIANQFNSAIGKIQDSLSSTASALGKLQDVVNHNAQ
EPI_ISL_7701122/BA.2/Omicron	FAMQMAYRFNGIGVTQNVLYENQKLIANQFNSAIGKIQDSLSSTASALGKLQDVVNHNAQ
EPI_ISL_7190366/BA.2/Omicron	FAMQMAYRFNGIGVTQNVLYENQKLIANQFNSAIGKIQDSLSSTASALGKLQDVVNHNAQ
EPI_ISL_7644798/BA.2/Omicron	FAMQMAYRFNGIGVTQNVLYENQKLIANQFNSAIGKIQDSLSSTASALGKLQDVVNHNAQ
EPI_ISL_7740766/BA.3/Omicron	FAMQMAYRFNGIGVTQNVLYENQKLIANQFNSAIGKIQDSLSSTASALGKLQDVVNHNAQ
EPI_ISL_7605589/BA.3/Omicron	FAMQMAYRFNGIGVTQNVLYENQKLIANQFNSAIGKIQDSLSSTASALGKLQDVVNHNAQ
ON296657.1/BA.4/Omicron	FAMQMAYRFNGIGVTQNVLYENQKLIANQFNSAIGKIQDSLSSTASALGKLQDVVNHNAQ
ON331287.1/BA.4/Omicron	FAMQMAYRFNGIGVTQNVLYENQKLIANQFNSAIGKIQDSLSSTASALGKLQDVVNHNAQ
ON321245.1/BA.4/Omicron	FAMQMAYRFNGIGVTQNVLYENQKLIANQFNSAIGKIQDSLSSTASALGKLQDVVNHNAQ
ON337003.1/BA.4/Omicron	FAMQMAYRFNGIGVTQNVLYENQKLIANQFNSAIGKIQDSLSSTASALGKLQDVVNHNAQ
ON270681.1/BA.4/Omicron	FAMQMAYRFNGIGVTQNVLYENQKLIANQFNSAIGKIQDSLSSTASALGKLQDVVNHNAQ
ON373214.1/BA.4/Omicron	FAMQMAYRFNGIGVTQNVLYENQKLIANQFNSAIGKIQDSLSSTASALGKLQDVVNHNAQ
ON393428.1/BA.5/Omicron	FAMQMAYRFNGIGVTQNVLYENQKLIANQFNSAIGKIQDSLSSTASALGKLQDVVNHNAQ
ON393156.1/BA.5/Omicron	FAMQMAYRFNGIGVTQNVLYENQKLIANQFNSAIGKIQDSLSSTASALGKLQDVVNHNAQ
ON348053.1/BA.5/Omicron	FAMQMAYRFNGIGVTQNVLYENQKLIANQFNSAIGKIQDSLSSTASALGKLQDVVNHNAQ
ON323731.1/BA.5/Omicron	FAMQMAYRFNGIGVTQNVLYENQKLIANQFNSAIGKIQDSLSSTASALGKLQDVVNHNAQ
ON251467.1/BA.5/Omicron	FAMQMAYRFNGIGVTQNVLYENQKLIANQFNSAIGKIQDSLSSTASALGKLQDVVNHNAQ
ON250431.1/BA.5/Omicron	FAMQMAYRFNGIGVTQNVLYENQKLIANQFNSAIGKIQDSLSSTASALGKLQDVVNHNAQ
MZ911221/B.1.427/Epsilon	FAMQMAYRFNGIGVTQNVLYENQKLIANQFNSAIGKIQDSLSSTASALGKLQDVVNQNAQ
MZ375858/B.1.427/Epsilon	FAMQMAYRFNGIGVTQNVLYENQKLIANQFNSAIGKIQDSLSSTASALGKLQDVVNQNAQ
MZ375854/B.1.427/Epsilon	FAMQMAYRFNGIGVTQNVLYENQKLIANQFNSAIGKIQDSLSSTASALGKLQDVVNQNAQ
MZ943834/B.1.429/Epsilon	FAMQMAYRFNGIGVTQNVLYENQKLIANQFNSAIGKIQDSLSSTASALGKLQDVVNQNAQ
MZ277390/B.1.429/Epsilon	FAMQMAYRFNGIGVTQNVLYENQKLIANQFNSAIGKIQDSLSSTASALGKLQDVVNQNAQ
OU282717/B.1.429/Epsilon	FAMQMAYRFNGIGVTQNVLYENQKLIANQFNSAIGKIQDSLSSTASALGKLQDVVNQNAQ
OU235436/B.1.429/Epsilon	FAMQMAYRFNGIGVTQNVLYENQKLIANQFNSAIGKIQDSLSSTASALGKLQDVVNQNAQ
MZ687447/B.1.429/Epsilon	FAMQMAYRFNGIGVTQNVLYENQKLIANQFNSAIGKIQDSLSSTASALGKLQDVVNQNAQ
MZ363839/B.1.617.1/Kappa	FAMQMAYRFNGIGVTQNVLYENQKLIANQFNSAIGKIQDSLSSTASALGKLQDVVNQNAQ
MZ538882/B.1.617.1/Kappa	FAMQMAYRFNGIGVTQNVLYENQKLIANQFNSAIGKIQDSLSSTASALGKLQDVVNQNAQ
MZ724425/B.1.617.1/Kappa	FAMQMAYRFNGIGVTQNVLYENQKLIANQFNSAIGKIQDSLSSTASALGKLQDVVNQNAQ
MZ332524/B.1.617.1/Kappa	FAMQMAYRFNGIGVTQNVLYENQKLIANQFNSAIGKIQDSLSSTASALGKLQDVVNQNAQ
MZ332527/B.1.617.1/Kappa	FAMQMAYRFNGIGVTQNVLYENQKLIANQFNSAIGKIQDSLSSTASALGKLQDVVNQNAQ
LC633761/B.1.617.1/Kappa	FAMQMAYRFNGIGVTQNVLYENQKLIANQFNSAIGKIQDSLSSTASALGKLQDVVNQNAQ
MZ571142/B.1.617.1/Kappa	FAMQMAYRFNGIGVTQNVLYENQKLIANQFNSAIGKIQDSLSSTASALGKLQDVVNQNAQ
MZ562746/B.1.617.1/Kappa	FAMQMAYRFNGIGVTQNVLYENQKLIANQFNSAIGKIQDSLSSTASALGKLQDVVNQNAQ
OU053018/C.37/Lambda	FAMQMAYRFNGIGVTQNVLYENQKLIANQFNSAIGKIQDSLSSTASALGKLQDVVNQNAQ
OU268528/C.37/Lambda	FAMQMAYRFNGIGVTQNVLYENQKLIANQFNSAIGKIQDSLSSTASALGKLQDVVNQNAQ
OU100999/C.37/Lambda	FAMQMAYRFNGIGVTQNVLYENQKLIANQFNSAIGKIQDSLSSTASALGKLQDVVNQNAQ
MZ275295/C.37/Lambda	FAMQMAYRFNGIGVTQNVLYENQKLIANQFNSAIGKIQDSLSSTASALGKLQDVVNQNAQ
MZ275301/C.37/Lambda	FAMQMAYRFNGIGVTQNVLYENQKLIANQFNSAIGKIQDSLSSTASALGKLQDVVNQNAQ
MZ908711/C.37/Lambda	FAMQMAYRFNGIGVTQNVLYENQKLIANQFNSAIGKIQDSLSSTASALGKLQDVVNQNAQ
OU517038/C.37/Lambda	FAMQMAYRFNGIGVTQNVLYENQKLIANQFNSAIGKIQDSLSSTASALGKLQDVVNQNAQ
MZ496613/C.37/Lambda	FAMQMAYRFNGIGVTQNVLYENQKLIANQFNSAIGKIQDSLSSTASALGKLQDVVNQNAQ
OU466149/B.1.621/Mu	FAMQMAYRFNGIGVTQNVLYENQKLIANQFNSAIGKIQDSLSSTASALGKLQNVVNQNAQ
OU571573/B.1.621/Mu	FAMQMAYRFNGIGVTQNVLYENQKLIANQFNSAIGKIQDSLSSTASALGKLQNVVNQNAQ
OK025270/B.1.621/Mu	FAMQMAYRFNGIGVTQNVLYENQKLIANQFNSAIGKIQDSLSSTASALGKLQNVVNQNAQ

```
                                      960       970       980       990       1000      1010
MN908947/Wuhan-Hu-1                ALNTLVKQLSSNFGAISSVLNDILSRLDKVEAEVQIDRLITGRLQSLQTYVTQQLIRAAE
BS001137/B.1.1.7/Alpha             ALNTLVKQLSSNFGAISSVLNDILARLDKVEAEVQIDRLITGRLQSLQTYVTQQLIRAAE
BS001138/B.1.1.7/Alpha             ALNTLVKQLSSNFGAISSVLNDILARLDKVEAEVQIDRLITGRLQSLQTYVTQQLIRAAE
BS001139/B.1.1.7/Alpha             ALNTLVKQLSSNFGAISSVLNDILARLDKVEAEVQIDRLITGRLQSLQTYVTQQLIRAAE
BS001140/B.1.1.7/Alpha             ALNTLVKQLSSNFGAISSVLNDILARLDKVEAEVQIDRLITGRLQSLQTYVTQQLIRAAE
MW913362/B.1.1.7/Alpha             ALNTLVKQLSSNFGAISSVLNDILARLDKVEAEVQIDRLITGRLQSLQTYVTQQLIRAAE
MZ266365/B.1.1.7/Alpha             ALNTLVKQLSSNFGAISSVLNDILARLDKVEAEVQIDRLITGRLQSLQTYVTQQLIRAAE
MZ350109/B.1.1.7/Alpha             ALNTLVKQLSSNFGAISSVLNDILARLDKVEAEVQIDRLITGRLQSLQTYVTQQLIRAAE
MZ888516/B.1.1.7/Alpha             ALNTLVKQLSSNFGAISSVLNDILARLDKVEAEVQIDRLITGRLQSLQTYVTQQLIRAAE
OU562607/B.1.1.7/Alpha             ALNTLVKQLSSNFGAISSVLNDILARLDKVEAEVQIDRLITGRLQSLQTYVTQQLIRAAE
OU514166/B.1.1.7/Alpha             ALNTLVKQLSSNFGAISSVLNDILARLDKVEAEVQIDRLITGRLQSLQTYVTQQLIRAAE
OU538849/B.1.1.7/Alpha             ALNTLVKQLSSNFGAISSVLNDILARLDKVEAEVQIDRLITGRLQSLQTYVTQQLIRAAE
MW981442/B.1.351/Beta              ALNTLVKQLSSNFGAISSVLNDILSRLDKVEAEVQIDRLITGRLQSLQTYVTQQLIRAAE
MZ298839/B.1.351/Beta              ALNTLVKQLSSNFGAISSVLNDILSRLDKVEAEVQIDRLITGRLQSLQTYVTQQLIRAAE
MZ913100/B.1.351/Beta              ALNTLVKQLSSNFGAISSVLNDILSRLDKVEAEVQIDRLITGRLQSLQTYVTQQLIRAAE
MZ879365/B.1.351/Beta              ALNTLVKQLSSNFGAISSVLNDILSRLDKVEAEVQIDRLITGRLQSLQTYVTQQLIRAAE
OU539352/B.1.351/Beta              ALNTLVKQLSSNFGAISSVLNDILSRLDKVEAEVQIDRLITGRLQSLQTYVTQQLIRAAE
MZ202314/B.1.351/Beta              ALNTLVKQLSSNFGAISSVLNDILSRLDKVEAEVQIDRLITGRLQSLQTYVTQQLIRAAE
MZ298840/B.1.351/Beta              ALNTLVKQLSSNFGAISSVLNDILSRLDKVEAEVQIDRLITGRLQSLQTYVTQQLIRAAE
LC643036/B.1.617.2/Delta           ALNTLVKQLSSNFGAISSVLNDILSRLDKVEAEVQIDRLITGRLQSLQTYVTQQLIRAAE
LC643044/B.1.617.2/Delta           ALNTLVKQLSSNFGAISSVLNDILSRLDKVEAEVQIDRLITGRLQSLQTYVTQQLIRAAE
MZ853946/B.1.617.2/Delta           ALNTLVKQLSSNFGAISSVLNDILSRLDKVEAEVQIDRLITGRLQSLQTYVTQQLIRAAE
MZ854386/B.1.617.2/Delta           ALNTLVKQLSSNFGAISSVLNDILSRLDKVEAEVQIDRLITGRLQSLQTYVTQQLIRAAE
MZ888532/B.1.617.2/Delta           ALNTLVKQLSSNFGAISSVLNDILSRLDKVEAEVQIDRLITGRLQSLQTYVTQQLIRAAE
MZ888533/B.1.617.2/Delta           ALNTLVKQLSSNFGAISSVLNDILSRLDKVEAEVQIDRLITGRLQSLQTYVTQQLIRAAE
MZ896136/B.1.617.2/Delta           ALNTLVKQLSSNFGAISSVLNDILSRLDKVEAEVQIDRLITGRLQSLQTYVTQQLIRAAE
OU539261/B.1.617.2/Delta           ALNTLVKQLSSNFGAISSVLNDILSRLDKVEAEVQIDRLITGRLQSLQTYVTQQLIRAAE
MZ854389/B.1.617.2/Delta           ALNTLVKQLSSNFGAISSVLNDILSRLDKVEAEVQIDRLITGRLQSLQTYVTQQLIRAAE
OU539784/P.1/Gamma                 ALNTLVKQLSSNFGAISSVLNDILSRLDKVEAEVQIDRLITGRLQSLQTYVTQQLIRAAE
OU539830/P.1/Gamma                 ALNTLVKQLSSNFGAISSVLNDILSRLDKVEAEVQIDRLITGRLQSLQTYVTQQLIRAAE
OU539894/P.1/Gamma                 ALNTLVKQLSSNFGAISSVLNDILSRLDKVEAEVQIDRLITGRLQSLQTYVTQQLIRAAE
MZ477748/P.1/Gamma                 ALNTLVKQLSSNFGAISSVLNDILSRLDKVEAEVQIDRLITGRLQSLQTYVTQQLIRAAE
MZ477746/P.1/Gamma                 ALNTLVKQLSSNFGAISSVLNDILSRLDKVEAEVQIDRLITGRLQSLQTYVTQQLIRAAE
MW938104/P.1/Gamma                 ALNTLVKQLSSNFGAISSVLNDILSRLDKVEAEVQIDRLITGRLQSLQTYVTQQLIRAAE
MZ896179/P.1/Gamma                 ALNTLVKQLSSNFGAISSVLNDILSRLDKVEAEVQIDRLITGRLQSLQTYVTQQLIRAAE
MZ896359/P.1/Gamma                 ALNTLVKQLSSNFGAISSVLNDILSRLDKVEAEVQIDRLITGRLQSLQTYVTQQLIRAAE
OL677199/BA.1/Omicron              ALNTLVKQLSSKFGAISSVLNDIFSRLDKVEAEVQIDRLITGRLQSLQTYVTQQLIRAAE
OL672836/BA.1/Omicron              ALNTLVKQLSSKFGAISSVLNDIFSRLDKVEAEVQIDRLITGRLQSLQTYVTQQLIRAAE
OW714547.1/BA.1/Omicron            ALNTLVKQLSSKFGAISSVLNDIFSRLDKVEAEVQIDRLITGRLQSLQTYVTQQLIRAAE
OW714198.1/BA.1/Omicron            ALNTLVKQLSSKFGAISSVLNDIFSRLDKVEAEVQIDRLITGRLQSLQTYVTQQLIRAAE
OW303180.1/BA.1/Omicron            ALNTLVKQLSSKFGAISSVLNDIFSRLDKVEAEVQIDRLITGRLQSLQTYVTQQLIRAAE
EPI_ISL_7747552/BA.2/Omicron       ALNTLVKQLSSKFGAISSVLNDILSRLDKVEAEVQIDRLITGRLQSLQTYVTQQLIRAAE
EPI_ISL_7747545/BA.2/Omicron       ALNTLVKQLSSKFGAISSVLNDILSRLDKVEAEVQIDRLITGRLQSLQTYVTQQLIRAAE
EPI_ISL_7747536/BA.2/Omicron       ALNTLVKQLSSKFGAISSVLNDILSRLDKVEAEVQIDRLITGRLQSLQTYVTQQLIRAAE
EPI_ISL_7747535/BA.2/Omicron       ALNTLVKQLSSKFGAISSVLNDILSRLDKVEAEVQIDRLITGRLQSLQTYVTQQLIRAAE
EPI_ISL_6795834/BA.2/Omicron       ALNTLVKQLSSKFGAISSVLNDILSRLDKVEAEVQIDRLITGRLQSLQTYVTQQLIRAAE
EPI_ISL_7718520/BA.2/Omicron       ALNTLVKQLSSKFGAISSVLNDILSRLDKVEAEVQIDRLITGRLQSLQTYVTQQLIRAAE
EPI_ISL_7580387/BA.2/Omicron       ALNTLVKQLSSKFGAISSVLNDILSRLDKVEAEVQIDRLITGRLQSLQTYVTQQLIRAAE
EPI_ISL_7701122/BA.2/Omicron       ALNTLVKQLSSKFGAISSVLNDILSRLDKVEAEVQIDRLITGRLQSLQTYVTQQLIRAAE
EPI_ISL_7190366/BA.2/Omicron       ALNTLVKQLSSKFGAISSVLNDILSRLDKVEAEVQIDRLITGRLQSLQTYVTQQLIRAAE
EPI_ISL_7644798/BA.2/Omicron       ALNTLVKQLSSKFGAISSVLNDILSRLDKVEAEVQIDRLITGRLQSLQTYVTQQLIRAAE
EPI_ISL_7740766/BA.3/Omicron       ALNTLVKQLSSKFGAISSVLNDILSRLDKVEAEVQIDRLITGRLQSLQTYVTQQLIRAAE
EPI_ISL_7605589/BA.3/Omicron       ALNTLVKQLSSKFGAISSVLNDILSRLDKVEAEVQIDRLITGRLQSLQTYVTQQLIRAAE
ON296657.1/BA.4/Omicron            ALNTLVKQLSSKFGAISSVLNDILSRLDKVEAEVQIDRLITGRLQSLQTYVTQQLIRAAE
ON331287.1/BA.4/Omicron            ALNTLVKQLSSKFGAISSVLNDILSRLDKVEAEVQIDRLITGRLQSLQTYVTQQLIRAAE
ON321245.1/BA.4/Omicron            ALNTLVKQLSSKFGAISSVLNDILSRLDKVEAEVQIDRLITGRLQSLQTYVTQQLIRAAE
ON337003.1/BA.4/Omicron            ALNTLVKQLSSKFGAISSVLNDILSRLDKVEAEVQIDRLITGRLQSLQTYVTQQLIRAAE
ON270681.1/BA.4/Omicron            ALNTLVKQLSSKFGAISSVLNDILSRLDKVEAEVQIDRLITGRLQSLQTYVTQQLIRAAE
ON373214.1/BA.4/Omicron            ALNTLVKQLSSKFGAISSVLNDILSRLDKVEAEVQIDRLITGRLQSLQTYVTQQLIRAAE
ON393428.1/BA.5/Omicron            ALNTLVKQLSSKFGAISSVLNDILSRLDKVEAEVQIDRLITGRLQSLQTYVTQQLIRAAE
ON393156.1/BA.5/Omicron            ALNTLVKQLSSKFGAISSVLNDILSRLDKVEAEVQIDRLITGRLQSLQTYVTQQLIRAAE
ON348053.1/BA.5/Omicron            ALNTLVKQLSSKFGAISSVLNDILSRLDKVEAEVQIDRLITGRLQSLQTYVTQQLIRAAE
ON323731.1/BA.5/Omicron            ALNTLVKQLSSKFGAISSVLNDILSRLDKVEAEVQIDRLITGRLQSLQTYVTQQLIRAAE
ON251467.1/BA.5/Omicron            ALNTLVKQLSSKFGAISSVLNDILSRLDKVEAEVQIDRLITGRLQSLQTYVTQQLIRAAE
ON250431.1/BA.5/Omicron            ALNTLVKQLSSKFGAISSVLNDILSRLDKVEAEVQIDRLITGRLQSLQTYVTQQLIRAAE
MZ911221/B.1.427/Epsilon           ALNTLVKQLSSNFGAISSVLNDILSRLDKVEAEVQIDRLITGRLQSLQTYVTQQLIRAAE
MZ375858/B.1.427/Epsilon           ALNTLVKQLSSNFGAISSVLNDILSRLDKVEAEVQIDRLITGRLQSLQTYVTQQLIRAAE
MZ375854/B.1.427/Epsilon           ALNTLVKQLSSNFGAISSVLNDILSRLDKVEAEVQIDRLITGRLQSLQTYVTQQLIRAAE
MZ943834/B.1.429/Epsilon           ALNTLVKQLSSNFGAISSVLNDILSRLDKVEAEVQIDRLITGRLQSLQTYVTQQLIRAAE
MZ277390/B.1.429/Epsilon           ALNTLVKQLSSNFGAISSVLNDILSRLDKVEAEVQIDRLITGRLQSLQTYVTQQLIRAAE
OU282717/B.1.429/Epsilon           ALNTLVKQLSSNFGAISSVLNDILSRLDKVEAEVQIDRLITGRLQSLQTYVTQQLIRAAE
OU235436/B.1.429/Epsilon           ALNTLVKQLSSNFGAISSVLNDILSRLDKVEAEVQIDRLITGRLQSLQTYVTQQLIRAAE
MZ687447/B.1.429/Epsilon           ALNTLVKQLSSNFGAISSVLNDILSRLDKVEAEVQIDRLITGRLQSLQTYVTQQLIRAAE
MZ363839/B.1.617.1/Kappa           ALNTLVKQLSSNFGAISSVLNDILSRLDKVEAEVQIDRLITGRLQSLQTYVTQQLIRAAE
MZ538882/B.1.617.1/Kappa           ALNTLVKQLSSNFGAISSVLNDILSRLDKVEAEVQIDRLITGRLQSLQTYVTQQLIRAAE
MZ724425/B.1.617.1/Kappa           ALNTLVKQLSSNFGAISSVLNDILSRLDKVEAEVQIDRLITGRLQSLQTYVTQQLIRAAE
MZ332524/B.1.617.1/Kappa           ALNTLVKQLSSNFGAISSVLNDILSRLDKVEAEVQIDRLITGRLQSLQTYVTQQLIRAAE
MZ332527/B.1.617.1/Kappa           ALNTLVKQLSSNFGAISSVLNDILSRLDKVEAEVQIDRLITGRLQSLQTYVTQQLIRAAE
LC633761/B.1.617.1/Kappa           ALNTLVKQLSSNFGAISSVLNDILSRLDKVEAEVQIDRLITGRLQSLQTYVTQQLIRAAE
MZ571142/B.1.617.1/Kappa           ALNTLVKQLSSNFGAISSVLNDILSRLDKVEAEVQIDRLITGRLQSLQTYVTQQLIRAAE
MZ562746/B.1.617.1/Kappa           ALNTLVKQLSSNFGAISSVLNDILSRLDKVEAEVQIDRLITGRLQSLQTYVTQQLIRAAE
OU053018/C.37/Lambda               ALNTLVKQLSSNFGAISSVLNDILSRLDKVEAEVQIDRLITGRLQSLQTYVTQQLIRAAE
OU268528/C.37/Lambda               ALNTLVKQLSSNFGAISSVLNDILSRLDKVEAEVQIDRLITGRLQSLQTYVTQQLIRAAE
OU100999/C.37/Lambda               ALNTLVKQLSSNFGAISSVLNDILSRLDKVEAEVQIDRLITGRLQSLQTYVTQQLIRAAE
MZ275295/C.37/Lambda               ALNTLVKQLSSNFGAISSVLNDILSRLDKVEAEVQIDRLITGRLQSLQTYVTQQLIRAAE
MZ275301/C.37/Lambda               ALNTLVKQLSSNFGAISSVLNDILSRLDKVEAEVQIDRLITGRLQSLQTYVTQQLIRAAE
MZ908711/C.37/Lambda               ALNTLVKQLSSNFGAISSVLNDILSRLDKVEAEVQIDRLITGRLQSLQTYVTQQLIRAAE
OU517038/C.37/Lambda               ALNTLVKQLSSNFGAISSVLNDILSRLDKVEAEVQIDRLITGRLQSLQTYVTQQLIRAAE
MZ496613/C.37/Lambda               ALNTLVKQLSSNFGAISSVLNDILSRLDKVEAEVQIDRLITGRLQSLQTYVTQQLIRAAE
OU466149/B.1.621/Mu                ALNTLVKQLSSNFGAISSVLNDILSRLDKVEAEVQIDRLITGRLQSLQTYVTQQLIRAAE
OU571573/B.1.621/Mu                ALNTLVKQLSSNFGAISSVLNDILSRLDKVEAEVQIDRLITGRLQSLQTYVTQQLIRAAE
OK025270/B.1.621/Mu                ALNTLVKQLSSNFGAISSVLNDILSRLDKVEAEVQIDRLITGRLQSLQTYVTQQLIRAAE
```

```
                                 1020      1030      1040      1050      1060      1070
MN908947/Wuhan-Hu-1              IRASANLAATKMSECVLGQSKRVDFCGKGYHLMSFPQSAPHGVVFLHVTYVPAQEKNFTT
BS001137/B.1.1.7/Alpha           IRASANLAATKMSECVLGQSKRVDFCGKGYHLMSFPQSAPHGVVFLHVTYVPAQEKNFTT
BS001138/B.1.1.7/Alpha           IRASANLAATKMSECVLGQSKRVDFCGKGYHLMSFPQSAPHGVVFLHVTYVPAQEKNFTT
BS001139/B.1.1.7/Alpha           IRASANLAATKMSECVLGQSKRVDFCGKGYHLMSFPQSAPHGVVFLHVTYVPAQEKNFTT
BS001140/B.1.1.7/Alpha           IRASANLAATKMSECVLGQSKRVDFCGKGYHLMSFPQSAPHGVVFLHVTYVPAQEKNFTT
MW913362/B.1.1.7/Alpha           IRASANLAATKMSECVLGQSKRVDFCGKGYHLMSFPQSAPHGVVFLHVTYVPAQEKNFTT
MZ266365/B.1.1.7/Alpha           IRASANLAATKMSECVLGQSKRVDFCGKGYHLMSFPQSAPHGVVFLHVTYVPAQEKNFTT
MZ350109/B.1.1.7/Alpha           IRASANLAATKMSECVLGQSKRVDFCGKGYHLMSFPQSAPHGVVFLHVTYVPAQEKNFTT
MZ888516/B.1.1.7/Alpha           IRASANLAATKMSECVLGQSKRVDFCGKGYHLMSFPQSAPHGVVFLHVTYVPAQEKNFTT
OU562607/B.1.1.7/Alpha           IRASANLAATKMSECVLGQSKRVDFCGKGYHLMSFPQSAPHGVVFLHVTYVPAQEKNFTT
OU514166/B.1.1.7/Alpha           IRSSANLAATKMSECVLGQSKRVDFCGKGYHLMSFPQSAPHGVVFLHVTYVPAQEKNFTT
OU538849/B.1.1.7/Alpha           IRASANLAATKMSECVLGQSKRVDFCGKGYHLMSFPQSAPHGVVFLHVTYVPAQEKNFTT
MW981442/B.1.351/Beta            IRASANLAATKMSECVLGQSKRVDFCGKGYHLMSFPQSAPHGVVFLHVTYVPAQEKNFTT
MZ298839/B.1.351/Beta            IRASANLAATKMSECVLGQSKRVDFCGKGYHLMSFPQSAPHGVVFLHVTYVPAQEKNFTT
MZ913100/B.1.351/Beta            IRASANLAATKMSECVLGQSKRVDFCGKGYHLMSFPQSAPHGVVFLHVTYVPAQEKNFTT
MZ879365/B.1.351/Beta            IRASANLAATKMSECVLGQSKRVDFCGKGYHLMSFPQSAPHGVVFLHVTYVPAQEKNFTT
OU539352/B.1.351/Beta            IRASANLAATKMSECVLGQSKRVDFCGKGYHLMSFPQSAPHGVVFLHVTYVPAQEKNFTT
MZ202314/B.1.351/Beta            IRASANLAATKMSECVLGQSKRVDFCGKGYHLMSFPQSAPHGVVFLHVTYVPAQEKNFTT
MZ298840/B.1.351/Beta            IRASANLAATKMSECVLGQSKRVDFCGKGYHLMSFPQSAPHGVVFLHVTYVPAQEKNFTT
LC643036/B.1.617.2/Delta         IRASANLAATKMSECVLGQSKRVDFCGKGYHLMSFPQSAPHGVVFLHVTYVPAQEKNFTT
LC643044/B.1.617.2/Delta         IRASANLAATKMSECVLGQSKRVDFCGKGYHLMSFPQSAPHGVVFLHVTYVPAQEKNFTT
MZ853946/B.1.617.2/Delta         IRASANLAATKMSECVLGQSKRVDFCGKGYHLMSFPQSAPHGVVFLHVTYVPAQEKNFTT
MZ854386/B.1.617.2/Delta         IRASANLAATKMSECVLGQSKRVDFCGKGYHLMSFPQSAPHGVVFLHVTYVPAQEKNFTT
MZ888532/B.1.617.2/Delta         IRASANLAATKMSECVLGQSKRVDFCGKGYHLMSFPQSAPHGVVFLHVTYVPAQEKNFTT
MZ888533/B.1.617.2/Delta         IRASANLAATKMSECVLGQSKRVDFCGKGYHLMSFPQSAPHGVVFLHVTYVPAQEKNFTT
MZ896136/B.1.617.2/Delta         IRASANLAATKMSECVLGQSKRVDFCGKGYHLMSFPQSAPHGVVFLHVTYVPAQEKNFTT
OU539261/B.1.617.2/Delta         IRASANLAATKMSECVLGQSKRVDFCGKGYHLMSFPQSAPHGVVFLHVTYVPAQEKNFTT
MZ854389/B.1.617.2/Delta         IRASANLAATKMSECVLGQSKRVDFCGKGYHLMSFPQSAPHGVVFLHVTYVPAQEKNFTT
OU539784/P.1/Gamma               IRASANLAAIKMSECVLGQSKRVDFCGKGYHLMSFPQSAPHGVVFLHVTYVPAQEKNFTT
OU539830/P.1/Gamma               IRASANLAAIKMSECVLGQSKRVDFCGKGYHLMSFPQSAPHGVVFLHVTYVPAQEKNFTT
OU539894/P.1/Gamma               IRASANLAAIKMSECVLGQSKRVDFCGKGYHLMSFPQSAPHGVVFLHVTYVPAQEKNFTT
MZ477748/P.1/Gamma               IRASANLAAIKMSECVLGQSKRVDFCGKGYHLMSFPQSAPHGVVFLHVTYVPAQEKNFTT
MZ477746/P.1/Gamma               IRASANLAAIKMSECVLGQSKRVDFCGKGYHLMSFPQSAPHGVVFLHVTYVPAQEKNFTT
MW938104/P.1/Gamma               IRASANLAAIKMSECVLGQSKRVDFCGKGYHLMSFPQSAPHGVVFLHVTYVPAQEKNFTT
MZ896179/P.1/Gamma               IRASANLAAIKMSECVLGQSKRVDFCGKGYHLMSFPQSAPHGVVFLHVTYVPAQEKNFTT
MZ896359/P.1/Gamma               IRASANLAAIKMSECVLGQSKRVDFCGKGYHLMSFPQSAPHGVVFLHVTYVPAQEKNFTT
OL677199/BA.1/Omicron            IRASANLAATKMSECVLGQSKRVDFCGKGYHLMSFPQSAPHGVVFLHVTYVPAQEKNFTT
OL672836/BA.1/Omicron            IRASANLAATKMSECVLGQSKRVDFCGKGYHLMSFPQSAPHGVVFLHVTYVPAQEKNFTT
OW714547.1/BA.1/Omicron          IRASANLAATKMSECVLGQSKRVDFCGKGYHLMSFPQSAPHGVVFLHVTYVPAQEKNFTT
OW714198.1/BA.1/Omicron          IRASANLAATKMSECVLGQSKRVDFCGKGYHLMSFPQSAPHGVVFLHVTYVPAQEKNFTT
OW303180.1/BA.1/Omicron          IRASANLAATKMSECVLGQSKRVDFCGKGYHLMSFPQSAPHGVVFLHVTYVPAQEKNFTT
EPI_ISL_7747552/BA.2/Omicron     IRASANLAATKMSECVLGQSKRVDFCGKGYHLMSFPQSAPHGVVFLHVTYVPAQEKNFTT
EPI_ISL_7747545/BA.2/Omicron     IRASANLAATKMSECVLGQSKRVDFCGKGYHLMSFPQSAPHGVVFLHVTYVPAQEKNFTT
EPI_ISL_7747536/BA.2/Omicron     IRASANLAATKMSECVLGQSKRVDFCGKGYHLMSFPQSAPHGVVFLHVTYVPAQEKNFTT
EPI_ISL_7747535/BA.2/Omicron     IRASANLAATKMSECVLGQSKRVDFCGKGYHLMSFPQSAPHGVVFLHVTYVPAQEKNFTT
EPI_ISL_6795834/BA.2/Omicron     IRASANLAATKMSECVLGQSKRVDFCGKGYHLMSFPQSAPHGVVFLHVTYVPAQEKNFTT
EPI_ISL_7718520/BA.2/Omicron     IRASANLAATKMSECVLGQSKRVDFCGKGYHLMSFPQSAPHGVVFLHVTYVPAQEKNFTT
EPI_ISL_7580387/BA.2/Omicron     IRASANLAATKMSECVLGQSKRVDFCGKGYHLMSFPQSAPHGVVFLHVTYVPAQEKNFTT
EPI_ISL_7701122/BA.2/Omicron     IRASANLAATKMSECVLGQSKRVDFCGKGYHLMSFPQSAPHGVVFLHVTYVPAQEKNFTT
EPI_ISL_7190366/BA.2/Omicron     IRASANLAATKMSECVLGQSKRVDFCGKGYHLMSFPQSAPHGVVFLHVTYVPAQEKNFTT
EPI_ISL_7644798/BA.2/Omicron     IRASANLAATKMSECVLGQSKRVDFCGKGYHLMSFPQSAPHGVVFLHVTYVPAQEKNFTT
EPI_ISL_7740766/BA.3/Omicron     IRASANLAATKMSECVLGQSKRVDFCGKGYHLMSFPQSAPHGVVFLHVTYVPAQEKNFTT
EPI_ISL_7605589/BA.3/Omicron     IRASANLAATKMSECVLGQSKRVDFCGKGYHLMSFPQSAPHGVVFLHVTYVPAQEKNFTT
ON296657.1/BA.4/Omicron          IRASANLAATKMSECVLGQSKRVDFCGKGYHLMSFPQSAPHGVVFLHVTYVPAQEKNFTT
ON331287.1/BA.4/Omicron          IRASANLAATKMSECVLGQSKRVDFCGKGYHLMSFPQSAPHGVVFLHVTYVPAQEKNFTT
ON321245.1/BA.4/Omicron          IRASANLAATKMSECVLGQSKRVDFCGKGYHLMSFPQSAPHGVVFLHVTYVPAQEKNFTT
ON337003.1/BA.4/Omicron          IRASANLAATKMSECVLGQSKRVDFCGKGYHLMSFPQSAPHGVVFLHVTYVPAQEKNFTT
ON270681.1/BA.4/Omicron          IRASANLAATKMSECVLGQSKRVDFCGKGYHLMSFPQSAPHGVVFLHVTYVPAQEKNFTT
ON373214.1/BA.4/Omicron          IRASANLAATKMSECVLGQSKRVDFCGKGYHLMSFPQSAPHGVVFLHVTYVPAQEKNFTT
ON393428.1/BA.5/Omicron          IRASANLAATKMSECVLGQSKRVDFCGKGYHLMSFPQSAPHGVVFLHVTYVPAQEKNFTT
ON393156.1/BA.5/Omicron          IRASANLAATKMSECVLGQSKRVDFCGKGYHLMSFPQSAPHGVVFLHVTYVPAQEKNFTT
ON348053.1/BA.5/Omicron          IRASANLAATKMSECVLGQSKRVDFCGKGYHLMSFPQSAPHGVVFLHVTYVPAQEKNFTT
ON323731.1/BA.5/Omicron          IRASANLAATKMSECVLGQSKRVDFCGKGYHLMSFPQSAPHGVVFLHVTYVPAQEKNFTT
ON251467.1/BA.5/Omicron          IRASANLAATKMSECVLGQSKRVDFCGKGYHLMSFPQSAPHGVVFLHVTYVPAQEKNFTT
ON250431.1/BA.5/Omicron          IRASANLAATKMSECVLGQSKRVDFCGKGYHLMSFPQSAPHGVVFLHVTYVPAQEKNFTT
MZ911221/B.1.427/Epsilon         IRASANLAATKMSECVLGQSKRVDFCGKGYHLMSFPQSAPHGVVFLHVTYVPAQEKNFTT
MZ375858/B.1.427/Epsilon         IRASANLAATKMSECVLGQSKRVDFCGKGYHLMSFPQSAPHGVVFLHVTYVPAQEKNFTT
MZ375854/B.1.427/Epsilon         IRASANLAATKMSECVLGQSKRVDFCGKGYHLMSFPQSAPHGVVFLHVTYVPAQEKNFTT
MZ943834/B.1.429/Epsilon         IRASANLAATKMSECVLGQSKRVDFCGKGYHLMSFPQSAPHGVVFLHVTYVPAQEKNFTT
MZ277390/B.1.429/Epsilon         IRASANLAATKMSECVLGQSKRVDFCGKGYHLMSFPQSAPHGVVFLHVTYVPAQEKNFTT
OU282717/B.1.429/Epsilon         IRASANLAATKMSECVLGQSKRVDFCGKGYHLMSFPQSAPHGVVFLHVTYVPAQEKNFTT
OU235436/B.1.429/Epsilon         IRASANLAATKMSECVLGQSKRVDFCGKGYHLMSFPQSAPHGVVFLHVTYVPAQEKNFTT
MZ687447/B.1.429/Epsilon         IRASANLAATKMSECVLGQSKRVDFCGKGYHLMSFPQSAPHGVVFLHVTYVPAQEKNFTT
MZ363839/B.1.617.1/Kappa         IRASANLAATKMSECVLGQSKRVDFCGKGYHLMSFPQSAPHGVVFLHVTYVPAHEKNFTT
MZ538882/B.1.617.1/Kappa         IRASANLAATKMSECVLGQSKRVDFCGKGYHLMSFPQSAPHGVVFLHVTYVPAHEKNFTT
MZ724425/B.1.617.1/Kappa         IRASANLAATKMSECVLGQSKRVDFCGKGYHLMSFPQSAPHGVVFLHVTYVPAHEKNFTT
MZ332524/B.1.617.1/Kappa         IRASANLAATKMSECVLGQSKRVDFCGKGYHLMSFPQSAPHGVVFLHVTYVPAHEKNFTT
MZ332527/B.1.617.1/Kappa         IRASANLAATKMSECVLGQSKRVDFCGKGYHLMSFPQSAPHGVVFLHVTYVPAHEKNFTT
LC633761/B.1.617.1/Kappa         IRASANLAATKMSECVLGQSKRVDFCGKGYHLMSFPQSAPHGVVFLHVTYVPAHEKNFTT
MZ571142/B.1.617.1/Kappa         IRASANLAATKMSECVLGQSKRVDFCGKGYHLMSFPQSAPHGVVFLHVTYVPAHEKNFTT
MZ562746/B.1.617.1/Kappa         IRASANLAATKMSECVLGQSKRVDFCGKGYHLMSFPQSAPHGVVFLHVTYVPAHEKNFTT
OU053018/C.37/Lambda             IRASANLAATKMSECVLGQSKRVDFCGKGYHLMSFPQSAPHGVVFLHVTYVPAQEKNFTT
OU268528/C.37/Lambda             IRASANLAATKMSECVLGQSKRVDFCGKGYHLMSFPQSAPHGVVFLHVTYVPAQEKNFTT
OU100999/C.37/Lambda             IRASANLAATKMSECVLGQSKRVDFCGKGYHLMSFPQSAPHGVVFLHVTYVPAQEKNFTT
MZ275295/C.37/Lambda             IRASANLAATKMSECVLGQSKRVDFCGKGYHLMSFPQSAPHGVVFLHVTYVPAQEKNFTT
MZ275301/C.37/Lambda             IRASANLAATKMSECVLGQSKRVDFCGKGYHLMSFPQSAPHGVVFLHVTYVPAQEKNFTT
MZ908711/C.37/Lambda             IRASANLAATKMSECVLGQSKRVDFCGKGYHLMSFPQSAPHGVVFLHVTYVPAQEKNFTT
OU517038/C.37/Lambda             IRASANLAATKMSECVLGQSKRVDFCGKGYHLMSFPQSAPHGVVFLHVTYVPAQEKNFTT
MZ496613/C.37/Lambda             IRASANLAATKMSECVLGQSKRVDFCGKGYHLMSFPQSAPHGVVFLHVTYVPAQEKNFTT
OU466149/B.1.621/Mu              IRASANLAATKMSECVLGQSKRVDFCGKGYHLMSFPQSAPHGVVFLHVTYVPAQEKNFTT
OU571573/B.1.621/Mu              IRASANLAATKMSECVLGQSKRVDFCGKGYHLMSFPQSAPHGVVFLHVTYVPAQEKNFTT
OK025270/B.1.621/Mu              IRASANLAATKMSECVLGQSKRVDFCGKGYHLMSFPQSAPHGVVFLHVTYVPAQEKNFTT
```

```
                                   1080      1090      1100      1110      1120      1130
MN908947/Wuhan-Hu-1                APAICHDGKAHFPREGVFVSNGTHWFVTQRNFYEPQIITTDNTFVSGNCDVVIGIVNNTV
BS001137/B.1.1.7/Alpha             APAICHDGKAHFPREGVFVSNGTHWFVTQRNFYEPQIITTHNTFVSGNCDVVIGIVNNTV
BS001138/B.1.1.7/Alpha             APAICHDGKAHFPREGVFVSNGTHWFVTQRNFYEPQIITTHNTFVSGNCDVVIGIVNNTV
BS001139/B.1.1.7/Alpha             APAICHDGKAHFPREGVFVSNGTHWFVTQRNFYEPQIITTHNTFVSGNCDVVIGIVNNTV
BS001140/B.1.1.7/Alpha             APAICHDGKAHFPREGVFVSNGTHWFVTQRNFYEPQIITTHNTFVSGNCDVVIGIVNNTV
MW913362/B.1.1.7/Alpha             APAICHDGKAHFPREGVFVSNGTHWFVTQRNFYEPQIITTHNTFVSGNCDVVIGIVNNTV
MZ266365/B.1.1.7/Alpha             APAICHDGKAHFPREGVFVSNGTHWFVTQRNFYEPQIITTHNTFVSGNCDVVIGIVNNTV
MZ350109/B.1.1.7/Alpha             APAICHDGKAHFPREGVFVSNGTHWFVTQRNFYEPQIITTHNTFVSGNCDVVIGIVNNTV
MZ888516/B.1.1.7/Alpha             APAICHDGKAHFPREGVFVSNGTHWFVTQRNFYEPQIITTHNTFVSGNCDVVIGIVNNTV
OU562607/B.1.1.7/Alpha             APAICHDGKAHFPREGVFVSNGTHWFVTQRNFYEPQIITTHNTFVSGNCDVVIGIVNNTV
OU514166/B.1.1.7/Alpha             APAICHDGKAHFPREGVFVSNGTHWFVTQRNFYEPQIITTHNTFVSGNCDVVIGIVNNTV
OU538849/B.1.1.7/Alpha             APAICHDGKAHFPREGVFVSNGTHWFVTQRNFYEPQIITTHNTFVSGNCDVVIGIVNNTV
MW981442/B.1.351/Beta              APAICHDGKAHFPREGVFVSNGTHWFVTQRNFYEPQIITTDNTFVSGNCDVVIGIVNNTV
MZ298839/B.1.351/Beta              APAICHDGKAHFPREGVFVSNGTHWFVTQRNFYEPQIITTDNTFVSCNCDVVIGIVNNTV
MZ913100/B.1.351/Beta              APAICHDGKAHFPREGVFVSNGTHWFVTQRNFYEPQIITTDNTFVSGNCDVVIGIVNNTV
MZ879365/B.1.351/Beta              APAICHDGKAHFPREGVFVSNGTHWFVTQRNFYEPQIITTDNTFVSGNCDVVIGIVNNTV
OU539352/B.1.351/Beta              APAICHDGKAHFPREGVFVSNGTHWFVTQRNFYEPQIITTDNTFVSGNCDVVIGIVNNTV
MZ202314/B.1.351/Beta              APAICHDGKAHFPREGVFVSNGTHWFVTQRNFYEPQIITTDNTFVSGNCDVVIGIVNNTV
MZ298840/B.1.351/Beta              APAICHDGKAHFPREGVFVSNGTHWFVTQRNFYEPQIITTDNTFVSCNCDVVIGIVNNTV
LC643036/B.1.617.2/Delta           APAICHDGKAHFPREGVFVSNGTHWFLTQRNFYEPQIITTDNTFVSGNCDVVIGIVNNTV
LC643044/B.1.617.2/Delta           APAICHDGKAHFPREGVFVSNGTHWFLTQRNFYEPQIITTDNTFVSGNCDVVIGIVNNTV
MZ853946/B.1.617.2/Delta           APAICHDGKAHFPREGVFVSNGTHWFVTQRNFYEPQIITTDNTFVSGNCDVVIGIVNNTV
MZ854386/B.1.617.2/Delta           APAICHDGKAHFPREGVFVSNGTHWFVTQRNFYEPQIITTDNTFVSGNCDVVIGIVNNTV
MZ888532/B.1.617.2/Delta           APAICHDGKAHFPREGVFVSNGTHWFVTQRNFYEPQIITTDNTFVSGNCDVVIGIVNNTV
MZ888533/B.1.617.2/Delta           APAICHDGKAHFPREGVFVSNGTHWFVTQRNFYEPQIITTDNTFVSGNCDVVIGIVNNTV
MZ896136/B.1.617.2/Delta           APAICHDGKAHFPREGVFVSNGTHWFVTQRNFYEPQIITTDNTFVSGNCDVVIGIVNNTV
OU539261/B.1.617.2/Delta           APAICHDGKAHFPREGVFVSNGTHWFVTQRNFYEPQIITTDNTFVSGNCDVVIGIVNNTV
MZ854389/B.1.617.2/Delta           APAICHDGKAHFPREGVFVSNGTHWFVTQRNFYEPQIITTDNTFVSGNCDVVIGIVNNTV
OU539784/P.1/Gamma                 APAICHDGKAHFPREGVFVSNGTHWFVTQRNFYEPQIITTDNTFVSGNCDVVIGIVNNTV
OU539830/P.1/Gamma                 APAICHDGKAHFPREGVFVSNGTHWFVTQRNFYEPQIITTDNTFVSGNCDVVIGIVNNTV
OU539894/P.1/Gamma                 APAICHDGKAHFPREGVFVSNGTHWFVTQRNFYEPQIITTDNTFVSGNCDVVIGIVNNTV
MZ477748/P.1/Gamma                 APAICHDGKAHFPREGVFVSNGTHWFVTQRNFYEPQIITTDNTFVSGNCDVVIGIVNNTV
MZ477746/P.1/Gamma                 APAICHDGKAHFPREGVFVSNGTHWFVTQRNFYEPQIITTDNTFVSGNCDVVIGIVNNTV
MW938104/P.1/Gamma                 APAICHDGKAHFPREGVFVSNGTHWFVTQRNFYEPQIITTDNTFVSGNCDVVIGIVNNTV
MZ896179/P.1/Gamma                 APAICHDGKAHFPREGVFVSNGTHWFVTQRNFYEPQIITTDNTFVSGNCDVVIGIVNNTV
MZ896359/P.1/Gamma                 APAICHDGKAHFPREGVFVSNGTHWFVTQRNFYEPQIITTDNTFVSGNCDVVIGIVNNTV
OL677199/BA.1/Omicron              APAICHDGKAHFPREGVFVSNGTHWFVTQRNFYEPQIITTDNTFVSGNCDVVIGIVNNTV
OL672836/BA.1/Omicron              APAICHDGKAHFPREGVFVSNGTHWFVTQRNFYEPQIITTDNTFVSGNCDVVIGIVNNTV
OW714547.1/BA.1/Omicron            APAICHDGKAHFPREGVFVSNGTHWFVTQRNFYEPQIITTDNTFVSGNCDVVIGIVNNTV
OW714198.1/BA.1/Omicron            APAICHDGKAHFPREGVFVSNGTHWFVTQRNFYEPQIITTDNTFVSGNCDVVIGIVNNTV
OW303180.1/BA.1/Omicron            APAICHDGKAHFPREGVFVSNGTHWFVTQRNFYEPQIITTDNTFVSGNCDVVIGIVNNTV
EPI_ISL_7747552/BA.2/Omicron       APAICHDGKAHFPREGVFVSNGTHWFVTQRNFYEPQIITTDNTFVSGNCDVVIGIVNNTV
EPI_ISL_7747545/BA.2/Omicron       APAICHDGKAHFPREGVFVSNGTHWFVTQRNFYEPQIITTDNTFVSGNCDVVIGIVNNTV
EPI_ISL_7747536/BA.2/Omicron       APAICHDGKAHFPREGVFVSNGTHWFVTQRNFYEPQIITTDNTFVSGNCDVVIGIVNNTV
EPI_ISL_7747535/BA.2/Omicron       APAICHDGKAHFPREGVFVSNGTHWFVTQRNFYEPQIITTDNTFVSGNCDVVIGIVNNTV
EPI_ISL_6795834/BA.2/Omicron       APAICHDGKAHFPREGVFVSNGTHWFVTQRNFYEPQIITTDNTFVSGNCDVVIGIVNNTV
EPI_ISL_7718520/BA.2/Omicron       APAICHDGKAHFPREGVFVSNGTHWFVTQRNFYEPQIITTDNTFVSGNCDVVIGIVNNTV
EPI_ISL_7580387/BA.2/Omicron       APAICHDGKAHFPREGVFVSNGTHWFVTQRNFYEPQIITTDNTFVSGNCDVVIGIVNNTV
EPI_ISL_7701122/BA.2/Omicron       APAICHDGKAHFPREGVFVSNGTHWFVTQRNFYEPQIITTDNTFVSGNCDVVIGIVNNTV
EPI_ISL_7190366/BA.2/Omicron       APAICHDGKAHFPREGVFVSNGTHWFVTQRNFYEPQIITTDNTFVSGNCDVVIGIVNNTV
EPI_ISL_7644798/BA.2/Omicron       APAICHDGKAHFPREGVFVSNGTHWFVTQRNFYEPQIITTDNTFVSGNCDVVIGIVNNTV
EPI_ISL_7740766/BA.3/Omicron       APAICHDGKAHFPREGVFVSNGTHWFVTQRNFYEPQIITTDNTFVSGNCDVVIGIVNNTV
EPI_ISL_7605589/BA.3/Omicron       APAICHDGKAHFPREGVFVSNGTHWFVTQRNFYEPQIITTDNTFVSGNCDVVIGIVNNTV
ON296657.1/BA.4/Omicron            APAICHDGKAHFPREGVFVSNGTHWFVTQRNFYEPQIITTDNTFVSGNCDVVIGIVNNTV
ON331287.1/BA.4/Omicron            APAICHDGKAHFPREGVFVSNGTHWFVTQRNFYEPQIITTDNTFVSGNCDVVIGIVNNTV
ON321245.1/BA.4/Omicron            APAICHDGKAHFPREGVFVSNGTHWFVTQRNFYEPQIITTDNTFVSGNCDVVIGIVNNTV
ON337003.1/BA.4/Omicron            APAICHDGKAHFPREGVFVSNGTHWFVTQRNFYEPQIITTDNTFVSGNCDVVIGIVNNTV
ON270681.1/BA.4/Omicron            APAICHDGKAHFPREGVFVSNGTHWFVTQRNFYEPQIITTDNTFVSGNCDVVIGIVNNTV
ON373214.1/BA.4/Omicron            APAICHDGKAHFPREGVFVSNGTHWFVTQRNFYEPQIITTDNTFVSGNCDVVIGIVNNTV
ON393428.1/BA.5/Omicron            APAICHDGKAHFPREGVFVSNGTHWFVTQRNFYEPQIITTDNTFVSGNCDVVIGIVNNTV
ON393156.1/BA.5/Omicron            APAICHDGKAHFPREGVFVSNGTHWFVTQRNFYEPQIITTDNTFVSGNCDVVIGIVNNTV
ON348053.1/BA.5/Omicron            APAICHDGKAHFPREGVFVSNGTHWFVTQRNFYEPQIITTDNTFVSGNCDVVIGIVNNTV
ON323731.1/BA.5/Omicron            APAICHDGKAHFPREGVFVSNGTHWFVTQRNFYEPQIITTDNTFVSGNCDVVIGIVNNTV
ON251467.1/BA.5/Omicron            APAICHDGKAHFPREGVFVSNGTHWFVTQRNFYEPQIITTDNTFVSGNCDVVIGIVNNTV
ON250431.1/BA.5/Omicron            APAICHDGKAHFPREGVFVSNGTHWFVTQRNFYEPQIITTDNTFVSGNCDVVIGIVNNTV
MZ911221/B.1.427/Epsilon           APAICHDGKAHFPREGVFVSNGTHWFVTQRNFYEPQIITTDNTFVSGNCDVVIGIVNNTV
MZ375858/B.1.427/Epsilon           APAICHDGKAHFPREGVFVSNGTHWFVTQRNFYEPQIITTDNTFVSGNCDVVIGIVNNTV
MZ375854/B.1.427/Epsilon           APAICHDGKAHFPREGVFVSNGTHWFVTQRNFYEPQIITTDNTFVSGNCDVVIGIVNNTV
MZ943834/B.1.429/Epsilon           APAICHDGKAHFPREGVFVSNGTHWFVTQRNFYEPQIITTDNTFVSGNCDVVIGIVNNTV
MZ277390/B.1.429/Epsilon           APAICHDGKAHFPREGVFVSNGTHWFVTQRNFYEPQIITTDNTFVSGNCDVVIGIVNNTV
OU282717/B.1.429/Epsilon           APAICHDGKAHFPREGVFVSNGTHWFVTQRNFYEPQIITTDNTFVSGNCDVVIGIVNNTV
OU235436/B.1.429/Epsilon           APAICHDGKAHFPREGVFVSNGTHWFVTQRNFYEPQIITTDNTFVSGNCDVVIGIVNNTV
MZ687447/B.1.429/Epsilon           APAICHDGKAHFPREGVFVSNGTHWFVTQRNFYEPQIITTDNTFVSGNCDVVIGIVNNTV
MZ363839/B.1.617.1/Kappa           APAICHDGKAHFPREGVFVSNGTHWFVTQRNFYEPQIITTDNTFVSGNCDVVIGIVNNTV
MZ538882/B.1.617.1/Kappa           APAICHDGKAHFPREGVFVSNGTHWFVTQRNFYEPQIITTDNTFVSGNCDVVIGIVNNTV
MZ724425/B.1.617.1/Kappa           APAICHDGKAHFPREGVFVSNGTHWFVTQRNFYEPQIITTDNTFVSGNCDVVIGIVNNTV
MZ332524/B.1.617.1/Kappa           APAICHDGKAHFPREGVFVSNGTHWFVTQRNFYEPQIITTDNTFVSGNCDVVIGIVNNTV
MZ332527/B.1.617.1/Kappa           APAICHDGKAHFPREGVFVSNGTHWFVTQRNFYEPQIITTDNTFVSGNCDVVIGIVNNTV
LC633761/B.1.617.1/Kappa           APAICHDGKAHFPREGVFVSNGTHWFVTQRNFYEPQIITTDNTFVSGNCDVVIGIVNNTV
MZ571142/B.1.617.1/Kappa           APAICHDGKAHFPREGVFVSNGTHWFVTQRNFYEPQIITTDNTFVSGNCDVVIGIVNNTV
MZ562746/B.1.617.1/Kappa           APAICHDGKAHFPREGVFVSNGTHWFVTQRNFYEPQIITTDNTFVSGNCDVVIGIVNNTV
OU053018/C.37/Lambda               APAICHDGKAHFPREGVFVSNGTHWFVTQRNFYEPQIITTDNTFVSGNCDVVIGIVNNTV
OU268528/C.37/Lambda               APAICHDGKAHFPREGVFVSNGTHWFVTQRNFYEPQIITTDNTFVSGNCDVVIGIVNNTV
OU100999/C.37/Lambda               APAICHDGKAHFPREGVFVSNGTHWFVTQRNFYEPQIITTDNTFVSGNCDVVIGIVNNTV
MZ275295/C.37/Lambda               APAICHDGKAHFPREGVFVSNGTHWFVTQRNFYEPQIITTDNTFVSGNCDVVIGIVNNTV
MZ275301/C.37/Lambda               APAICHDGKAHFPREGVFVSNGTHWFVTQRNFYEPQIITTDNTFVSGNCDVVIGIVNNTV
MZ908711/C.37/Lambda               APAICHDGKAHFPREGVFVSNGTHWFVTQRNFYEPQIITTDNTFVSGNCDVVIGIVNNTV
OU517038/C.37/Lambda               APAICHDGKAHFPREGVFVSNGTHWFVTQRNFYEPQIITTDNTFVSGNCDVVIGIVNNTV
MZ496613/C.37/Lambda               APAICHDGKAHFPREGVFVSNGTHWFVTQRNFYEPQIITTDNTFVSGNCDVVIGIVNNTV
OU466149/B.1.621/Mu                APAICHDGKAHFPREGVFVSNGTHWFVTQRNFYEPQIITTDNTFVSGNCDVVIGIVNNTV
OU571573/B.1.621/Mu                APAICHDGKAHFPREGVFVSNGTHWFVTQRNFYEPQIITTDNTFVSGNCDVVIGIVNNTV
OK025270/B.1.621/Mu                APAICHDGKAHFPREGVFVSNGTHWFVTQRNFYEPQIITTDNTFVSGNCDVVIGIVNNTV
```

```
                                      1140      1150      1160      1170      1180      1190
                                         .         .         .         .         .         .
MN908947/Wuhan-Hu-1                 YDPLQPELDSFKEELDKYFKNHTSPDVDLGDISGINASVVNIQKEIDRLNEVAKNLNESL
BS001137/B.1.1.7/Alpha              YDPLQPELDSFKEELDKYFKNHTSPDVDLGDISGINASVVNIQKEIDRLNEVAKNLNESL
BS001138/B.1.1.7/Alpha              YDPLQPELDSFKEELDKYFKNHTSPDVDLGDISGINASVVNIQKEIDRLNEVAKNLNESL
BS001139/B.1.1.7/Alpha              YDPLQPELDSFKEELDKYFKNHTSPDVDLGDISGINASVVNIQKEIDRLNEVAKNLNESL
BS001140/B.1.1.7/Alpha              YDPLQPELDSFKEELDKYFKNHTSPDVDLGDISGINASVVNIQKEIDRLNEVAKNLNESL
MW913362/B.1.1.7/Alpha              YDPLQPELDSFKEELDKYFKNHTSPDVDLGDISGINASVVNIQKEIDRLNEVAKNLNESL
MZ266365/B.1.1.7/Alpha              YDPLQPELDSFKEELDKYFKNHTSPDVDLGDISGINASVVNIQKEIDRLNEVAKNLNESL
MZ350109/B.1.1.7/Alpha              YDPLQPELDSFKEELDKYFKNHTSPDVDLGDISGINASVVNIQKEIDRLNEVAKNLNESL
MZ888516/B.1.1.7/Alpha              YDPLQPELDSFKEELDKYFKNHTSPDVDLGDISGINASVVNIQKEIDRLNEVAKNLNESL
OU562607/B.1.1.7/Alpha              YDPLQPELDSFKEELDKYFKNHTSPDVDLGDISGINASVVNIQKEIDRLNEVAKNLNESL
OU514166/B.1.1.7/Alpha              YDPLQPELDSFKEELDKYFKNHTSPDVDLGDISGINASVVNIQKEIDRLNEVAKNLNESL
OU538849/B.1.1.7/Alpha              YDPLQPELDSFKEELDKYFKNHTSPDVDLGDISGINASVVNIQKEIDRLNEVAKNLNESL
MW981442/B.1.351/Beta               YDPLQPELDSFKEELDKYFKNHTSPDVDLGDISGINASVVNIQKEIDRLNEVAKNLNESL
MZ298839/B.1.351/Beta               YDPLQPELDSFKEELDKYFKNHTSPDVDLGDISGINASVVNIQKEIDRLNEVAKNLNESL
MZ913100/B.1.351/Beta               YDPLQPELDSFKEELDKYFKNHTSPDVDLGDISGINASVVNIQKEIDRLNEVAKNLNESL
MZ879365/B.1.351/Beta               YDPLQPELDSFKEELDKYFKNHTSPDVDLGDISGINASVVNIQKEIDRLNEVAKNLNESL
OU539352/B.1.351/Beta               YDPLQPELDSFKEELDKYFKNHTSPDVDLGDISGINASVVNIQKEIDRLNEVAKNLNESL
MZ202314/B.1.351/Beta               YDPLQPELDSFKEELDKYFKNHTSPDVDLGDISGINASVVNIQKEIDRLNEVAKNLNESL
MZ298840/B.1.351/Beta               YDPLQPELDSFKEELDKYFKNHTSPDVDLGDISGINASVVNIQKEIDRLNEVAKNLNESL
LC643036/B.1.617.2/Delta            YDPLQPELDSFKEELDKYFKNHTSPDVDLGDISGINASVVNIQKEIDRLNEVAKNLNESL
LC643044/B.1.617.2/Delta            YDPLQPELDSFKEELDKYFKNHTSPDVDLGDISGINASVVNIQKEIDRLNEVAKNLNESL
MZ853946/B.1.617.2/Delta            YDPWQPELDSFKEELDKYFKNHTSPDVDLGDISGINASVVNIQKEIDRLNEVAKNLNESL
MZ854386/B.1.617.2/Delta            YDPLQPELDSFKEELDKYFKNHTSPDVDLGDISGINASVVNIQKEIDRLNEVAKNLNESL
MZ888532/B.1.617.2/Delta            YDPLQPELDSFKEELDKYFKNHTSPDVDLGDISGINASVVNIQKEIDRLNEVAKNLNESL
MZ888533/B.1.617.2/Delta            YDPLQPELDSFKEELDKYFKNHTSPDVDLGDISGINASVVNIQKEIDRLNEVAKNLNESL
MZ896136/B.1.617.2/Delta            YDPLQPELDSFKEELDKYFKNHTSPDVDLGDISGINASVVNIQKEIDRLNEVAKNLNESL
OU539261/B.1.617.2/Delta            YDPLQPELDSFKEELDKYFKNHTSPDVDLGDISGINASVVNIQKEIDRLNEVAKNLNESL
MZ854389/B.1.617.2/Delta            YDPLQPELDSFKEELDKYFKNHTSPDVDLGDISGINASVVNIQKEIDRLNEVAKNLNESL
OU539784/P.1/Gamma                  YDPLQPELDSFKEELDKYFKNHTSPDVDLGDISGINASFVNIQKEIDRLNEVAKNLNESL
OU539830/P.1/Gamma                  YDPLQPELDSFKEELDKYFKNHTSPDVDLGDISGINASFVNIQKEIDRLNEVAKNLNESL
OU539894/P.1/Gamma                  YDPLQPELDSFKEELDKYFKNHTSPDVDLGDISGINASFVNIQKEIDRLNEVAKNLNESL
MZ477748/P.1/Gamma                  YDPLQPELDSFKEELDKYFKNHTSPDVDLGDISGINASFVNIQKEIDRLNEVAKNLNESL
MZ477746/P.1/Gamma                  YDPLQPELDSFKEELDKYFKNHTSPDVDLGDISGINASFVNIQKEIDRLNEVAKNLNESL
MW938104/P.1/Gamma                  YDPLQPELDSFKEELDKYFKNHTSPDVDLGDISGINASFVNIQKEIDRLNEVAKNLNESL
MZ896179/P.1/Gamma                  YDPLQPELDSFKEELDKYFKNHTSPDVDLGDISGINASFVNIQKEIDRLNEVAKNLNESL
MZ896359/P.1/Gamma                  YDPLQPELDSFKEELDKYFKNHTSPDVDLGDISGINASFVNIQKEIDRLNEVAKNLNESL
OL677199/BA.1/Omicron               YDPLQPELDSFKEELDKYFKNHTSPDVDLGDISGINASVVNIQKEIDRLNEVAKNLNESL
OL672836/BA.1/Omicron               YDPLQPELDSFKEELDKYFKNHTSPDVDLGDISGINASVVNIQKEIDRLNEVAKNLNESL
OW714547.1/BA.1/Omicron             YDPLQPELDSFKEELDKYFKNHTSPDVDLGDISGINASVVNIQKEIDRLNEVAKNLNESL
OW714198.1/BA.1/Omicron             YDPLQPELDSFKEELDKYFKNHTSPDVDLGDISGINASVVNIQKEIDRLNEVAKNLNESL
OW303180.1/BA.1/Omicron             YDPLQPELDSFKEELDKYFKNHTSPDVDLGDISGINASVVNIQKEIDRLNEVAKNLNESL
EPI_ISL_7747552/BA.2/Omicron        YDPLQPELDSFKEELDKYFKNHTSPDVDLGDISGINASVVNIQKEIDRLNEVAKNLNESL
EPI_ISL_7747545/BA.2/Omicron        YDPLQPELDSFKEELDKYFKNHTSPDVDLGDISGINASVVNIQKEIDRLNEVAKNLNESL
EPI_ISL_7747536/BA.2/Omicron        YDPLQPELDSFKEELDKYFKNHTSPDVDLGDISGINASVVNIQKEIDRLNEVAKNLNESL
EPI_ISL_7747535/BA.2/Omicron        YDPLQPELDSFKEELDKYFKNHTSPDVDLGDISGINASVVNIQKEIDRLNEVAKNLNESL
EPI_ISL_6795834/BA.2/Omicron        YDPLQPELDSFKEELDKYFKNHTSPDVDLGDISGINASVVNIQKEIDRLNEVAKNLNESL
EPI_ISL_7718520/BA.2/Omicron        YDPLQPELDSFKEELDKYFKNHTSPDVDLGDISGINASVVNIQKEIDRLNEVAKNLNESL
EPI_ISL_7580387/BA.2/Omicron        YDPLQPELDSFKEELDKYFKNHTSPDVDLGDISGINASVVNIQKEIDRLNEVAKNLNESL
EPI_ISL_7701122/BA.2/Omicron        YDPLQPELDSFKEELDKYFKNHTSPDVDLGDISGINASVVNIQKEIDRLNEVAKNLNESL
EPI_ISL_7190366/BA.2/Omicron        YDPLQPELDSFKEELDKYFKNHTSPDVDLGDISGINASVVNIQKEIDRLNEVAKNLNESL
EPI_ISL_7644798/BA.2/Omicron        YDPLQPELDSFKEELDKYFKNHTSPDVDLGDISGINASVVNIQKEIDRLNEVAKNLNESL
EPI_ISL_7740766/BA.3/Omicron        YDPLQPELDSFKEELDKYFKNHTSPDVDLGDISGINASVVNIQKEIDRLNEVAKNLNESL
EPI_ISL_7605589/BA.3/Omicron        YDPLQPELDSFKEELDKYFKNHTSPDVDLGDISGINASVVNIQKEIDRLNEVAKNLNESL
ON296657.1/BA.4/Omicron             YDPLQPELDSFKEELDKYFKNHTSPDVDLGDISGINASVVNIQKEIDRLNEVAKNLNESL
ON331287.1/BA.4/Omicron             YDPLQPELDSFKEELDKYFKNHTSPDVDLGDISGINASVVNIQKEIDRLNEVAKNLNESL
ON321245.1/BA.4/Omicron             YDPLQPELDSFKEELDKYFKNHTSPDVDLGDISGINASVVNIQKEIDRLNEVAKNLNESL
ON337003.1/BA.4/Omicron             YDPLQPELDSFKEELDKYFKNHTSPDVDLGDISGINASVVNIQKEIDRLNEVAKNLNESL
ON270681.1/BA.4/Omicron             YDPLQPELDSFKEELDKYFKNHTSPDVDLGDISGINASVVNIQKEIDRLNEVAKNLNESL
ON373214.1/BA.4/Omicron             YDPLQPELDSFKEELDKYFKNHTSPDVDLGDISGINASVVNIQKEIDRLNEVAKNLNESL
ON393428.1/BA.5/Omicron             YDPLQPELDSFKEELDKYFKNHTSPDVDLGDISGINASVVNIQKEIDRLNEVAKNLNESL
ON393156.1/BA.5/Omicron             YDPLQPELDSFKEELDKYFKNHTSPDVDLGDISGINASVVNIQKEIDRLNEVAKNLNESL
ON348053.1/BA.5/Omicron             YDPLQPELDSFKEELDKYFKNHTSPDVDLGDISGINASVVNIQKEIDRLNEVAKNLNESL
ON323731.1/BA.5/Omicron             YDPLQPELDSFKEELDKYFKNHTSPDVDLGDISGINASVVNIQKEIDRLNEVAKNLNESL
ON251467.1/BA.5/Omicron             YDPLQPELDSFKEELDKYFKNHTSPDVDLGDISGINASVVNIQKEIDRLNEVAKNLNESL
ON250431.1/BA.5/Omicron             YDPLQPELDSFKEELDKYFKNHTSPDVDLGDISGINASVVNIQKEIDRLNEVAKNLNESL
MZ911221/B.1.427/Epsilon            YDPLQPELDSFKEELDKYFKNHTSPDVDLGDISGINASVVNIQKEIDRLNEVAKNLNESL
MZ375858/B.1.427/Epsilon            YDPLQPELDSFKEELDKYFKNHTSPDVDLGDISGINASVVNIQKEIDRLNEVAKNLNESL
MZ375854/B.1.427/Epsilon            YDPLQPELDSFKEELDKYFKNHTSPDVDLGDISGINASVVNIQKEIDRLNEVAKNLNESL
MZ943834/B.1.429/Epsilon            YDPLQPELDSFKEELDKYFKNHTSPDVDLGDISGINASVVNIQKEIDRLNEVAKNLNESL
MZ277390/B.1.429/Epsilon            YDPLQPELDSFKEELDKYFKNHTSPDVDLGDISGINASVVNIQKEIDRLNEVAKNLNESL
OU282717/B.1.429/Epsilon            YDPLQPELDSFKEELDKYFKNHTSPDVDLGDISGINASVVNIQKEIDRLNEVAKNLNESL
OU235436/B.1.429/Epsilon            YDPLQPELDSFKEELDKYFKNHTSPDVDLGDISGINASVVNIQKEIDRLNEVAKNLNESL
MZ687447/B.1.429/Epsilon            YDPLQPELDSFKEELDKYFKNHTSPDVDLGDISGINASVVNIQKEIDRLNEVAKNLNESL
MZ363839/B.1.617.1/Kappa            YDPLQPELDSFKEELDKYFKNHTSPDVDLGDISGINASVVNIQKEIDRLNEVAKNLNESL
MZ538882/B.1.617.1/Kappa            YDPLQPELDSFKEELDKYFKNHTSPDVDLGDISGINASVVNIQKEIDRLNEVAKNLNESL
MZ724425/B.1.617.1/Kappa            YDPLQPELDSFKEELDKYFKNHTSPDVDLGDISGINASVVNIQKEIDRLNEVAKNLNESL
MZ332524/B.1.617.1/Kappa            YDPLQPELDSFKEELDKYFKNHTSPDVDLGDISGINASVVNIQKEIDRLNEVAKNLNESL
MZ332527/B.1.617.1/Kappa            YDPLQPELDSFKEELDKYFKNHTSPDVDLGDISGINASVVNIQKEIDRLNEVAKNLNESL
LC633761/B.1.617.1/Kappa            YDPLQPELDSFKEELDKYFKNHTSPDVDLGDISGINASVVNIQKEIDRLNEVAKNLNESL
MZ571142/B.1.617.1/Kappa            YDPLQPELDSFKEELDKYFKNHTSPDVDLGDISGINASVVNIQKEIDRLNEVAKNLNESL
MZ562746/B.1.617.1/Kappa            YDPLQPELDSFKEELDKYFKNHTSPDVDLGDISGINASVVNIQKEIDRLNEVAKNLNESL
OU053018/C.37/Lambda                YDPLQPELDSFKEELDKYFKNHTSPDVDLGDISGINASVVNIQKEIDRLNEVAKNLNESL
OU268528/C.37/Lambda                YDPLQPELDSFKEELDKYFKNHTSPDVDLGDISGINASVVNIQKEIDRLNEVAKNLNESL
OU100999/C.37/Lambda                YDPLQPELDSFKEELDKYFKNHTSPDVDLGDISGINASVVNIQKEIDRLNEVAKNLNESL
MZ275295/C.37/Lambda                YDPLQPELDSFKEELDKYFKNHTSPDVDLGDISGINASVVNIQKEIDRLNEVAKNLNESL
MZ275301/C.37/Lambda                YDPLQPELDSFKEELDKYFKNHTSPDVDLGDISGINASVVNIQKEIDRLNEVAKNLNESL
MZ908711/C.37/Lambda                YDPLQPELDSFKEELDKYFKNHTSPDVDLGDISGINASVVNIQKEIDRLNEVAKNLNESL
OU517038/C.37/Lambda                YDPLQPELDSFKEELDKYFKNHTSPDVDLGDISGINASVVNIQKEIDRLNEVAKNLNESL
MZ496613/C.37/Lambda                YDPLQPELDSFKEELDKYFKNHTSPDVDLGDISGINASVVNIQKEIDRLNEVAKNLNESL
OU466149/B.1.621/Mu                 YDPLQPELDSFKEELDKYFKNHTSPDVDLGDISGINASVVNIQKEIDRLNEVAKNLNESL
OU571573/B.1.621/Mu                 YDPLQPELDSFKEELDKYFKNHTSPDVDLGDISGINASVVNIQKEIDRLNEVAKNLNESL
OK025270/B.1.621/Mu                 YDPLQPELDSFKEELDKYFKNHTSPDVDLGDISGINASVVNIQKEIDRLNEVAKNLNESL
```

```
                                 1200      1210      1220      1230      1240      1250
MN908947/Wuhan-Hu-1              IDLQELGKYEQYIKWPWYIWLGFIAGLIAIVMVTIMLCCMTSCCSCLKGCCSCGSCCKFD
BS001137/B.1.1.7/Alpha           IDLQELGKYEQYIKWPWYIWLGFIAGLIAIVMVTIMLCCMTSCCSCLKGCCSCGSCCKFD
BS001138/B.1.1.7/Alpha           IDLQELGKYEQYIKWPWYIWLGFIAGLIAIVMVTIMLCCMTSCCSCLKGCCSCGSCCKFD
BS001139/B.1.1.7/Alpha           IDLQELGKYEQYIKWPWYIWLGFIAGLIAIVMVTIMLCCMTSCCSCLKGCCSCGSCCKFD
BS001140/B.1.1.7/Alpha           IDLQELGKYEQYIKWPWYIWLGFIAGLIAIVMVTIMLCCMTSCCSCLKGCCSCGSCCKFD
MW913362/B.1.1.7/Alpha           IDLQELGKYEQYIKWPWYIWLGFIAGLIAIVMVTIMLCCMTSCCSCLKGCCSCGSCCKFD
MZ266365/B.1.1.7/Alpha           IDLQELGKYEQYIKWPWYIWLGFIAGLIAIVMVTIMLCCMTSCCSCLKGCCSCGSCCKFD
MZ350109/B.1.1.7/Alpha           IDLQELGKYEQYIKWPWYIWLGFIAGLIAIVMVTIMLCCMTSCCSCLKGCCSCGSCCKFD
MZ888516/B.1.1.7/Alpha           IDLQELGKYEQYIKWPWYIWLGFIAGLIAIVMVTIMLCCMTSCCSCLKGCCSCGSCCKFD
OU562607/B.1.1.7/Alpha           IDLQELGKYEQYIKWPWYIWLGFIAGLIAIVMVTIMLCCMTSCCSCLKGCCSCGSCCKFD
OU514166/B.1.1.7/Alpha           IDLQELGKYEQYIKWPWYIWLGFIAGLIAIVMVTIMLCCMTSCCSCLKGCCSCGSCCKFD
OU538849/B.1.1.7/Alpha           IDLQELGKYEQYIKWPWYIWLGFIAGLIAIVMVTIMLCCMTSCCSCLKGCCSCGSCCKFD
MW981442/B.1.351/Beta            IDLQELGKYEQYIKWPWYIWLGFIAGLIAIVMVTIMLCCMTSCCSCLKGCCSCGSCCKFD
MZ298839/B.1.351/Beta            IDLQELGKYEQYIKWPWYIWLGFIAGLIAIVMVTIMLCCMTSCCSCLKGCCSCGSCCKFD
MZ913100/B.1.351/Beta            IDLQELGKYEQYIKWPWYIWLGFIAGLIAIVMVTIMLCCMTSCCSCLKGCCSCGSCCKFD
MZ879365/B.1.351/Beta            IDLQELGKYEQYIKWPWYIWLGFIAGLIAIVMVTIMLCCMTSCCSCLKGCCSCGSCCKFD
OU539352/B.1.351/Beta            IDLQELGKYEQYIKWPWYIWLGFIAGLIAIVIVTIMLCCMTSCCSCLKGCCSCGSCCKFD
MZ202314/B.1.351/Beta            IDLQELGKYEQYIKWPWYIWLGFIAGLIAIVMVTIMLCCMTSCCSCLKGCCSCGSCCKFD
MZ298840/B.1.351/Beta            IDLQELGKYEQYIKWPWYIWLGFIAGLIAIVMVTIMLCCMTSCCSCLKGCCSCGSCCKFD
LC643036/B.1.617.2/Delta         IDLQELGKYEQYIKWPWYIWLGFIAGLIAIVMVTIMLCCMTSCCSCLKGCCSCGSCCKFD
LC643044/B.1.617.2/Delta         IDLQELGKYEQYIKWPWYIWLGFIAGLIAIVMVTIMLCCMTSCCSCLKGCCSCGSCCKFD
MZ853946/B.1.617.2/Delta         IDLQELGKYEQYIKWPWYIWLGFIAGLIAIVMVTIMLCCMTSCCSCLKGCCSCGSCCKFD
MZ854386/B.1.617.2/Delta         IDLQELGKYEQYIKWPWYIWLGFIAGLIAIVMVTIMLCCMTSCCSCLKGCCSCGSCCKFD
MZ888532/B.1.617.2/Delta         IDLQELGKYEQYIKWPWYIWLGFIAGLIAIVMVTIMLCCMTSCCSCLKGCCSCGSCCKFD
MZ888533/B.1.617.2/Delta         IDLQELGKYEQYIKWPWYIWLGFIAGLIAIVMVTIMLCCMTSCCSCLKGCCSCGSCCKFD
MZ896136/B.1.617.2/Delta         IDLQELGKYEQYIKWPWYIWLGFIAGLIAIVMVTIMLCCMTSCCSCLKGCCSCGSCCKFD
OU539261/B.1.617.2/Delta         IDLQELGKYEQYIKWPWYIWLGFIAGLIAIVMVTIMLCCMTSCCSCLKGCCSCGSCCKFD
MZ854389/B.1.617.2/Delta         IDLQELGKYEQYIKWPWYIWLGFIAGLIAIVMVTIMLCCMTSCCSCLKGCCSCGSCCKFD
OU539784/P.1/Gamma               IDLQELGKYEQYIKWPWYIWLGFIAGLIAIVMVTIMLCCMTSCCSCLKGCCSCGSCCKFD
OU539830/P.1/Gamma               IDLQELGKYEQYIKWPWYIWLGFIAGLIAIVMVTIMLCCMTSCCSCLKGCCSCGSCCKFD
OU539894/P.1/Gamma               IDLQELGKYEQYIKWPWYIWLGFIAGLIAIVMVTIMLCCMTSCCSCLKGCCSCGSCCKFD
MZ477748/P.1/Gamma               IDLQELGKYEQYIKWPWYIWLGFIAGLIAIVMVTIMLCCMTSCCSCLKGCCSCGSCCKFD
MZ477746/P.1/Gamma               IDLQELGKYEQYIKWPWYIWLGFIAGLIAIVMVTIMLCCMTSCCSCLKGCCSCGSCCKFD
MW938104/P.1/Gamma               IDLQELGKYEQYIKWPWYIWLGFIAGLIAIVMVTIMLCCMTSCCSCLKGCCSCGSCCKFD
MZ896179/P.1/Gamma               IDLQELGKYEQYIKWPWYIWLGFIAGLIAIVMVTIMLCCMTSCCSCLKGCCSCGSCCKFD
MZ896359/P.1/Gamma               IDLQELGKYEQYIKWPWYIWLGFIAGLIAIVMVTIMLCCMTSCCSCLKGCCSCGSCCKFD
OL677199/BA.1/Omicron            IDLQELGKYEQYIKWPWYIWLGFIAGLIAIVMVTIMLCCMTSCCSCLKGCCSCGSCCKFD
OL672836/BA.1/Omicron            IDLQELGKYEQYIKWPWYIWLGFIAGLIAIVMVTIMLCCMTSCCSCLKGCCSCGSCCKFD
OW714547.1/BA.1/Omicron          IDLQELGKYEQYIKWPWYIWLGFIAGLIAIVMVTIMLCCMTSCCSCLKGCCSCGSCCKFD
OW714198.1/BA.1/Omicron          IDLQELGKYEQYIKWPWYIWLGFIAGLIAIVMVTIMLCCMTSCCSCLKGCCSCGSCCKFD
OW303180.1/BA.1/Omicron          IDLQELGKYEQYIKWPWYIWLGFIAGLIAIVMVTIMLCCMTSCCSCLKGCCSCGSCCKFD
EPI_ISL_7747552/BA.2/Omicron     IDLQELGKYEQYIKWPWYIWLGFIAGLIAIVMVTIMLCCMTSCCSCLKGCCSCGSCCKFD
EPI_ISL_7747545/BA.2/Omicron     IDLQELGKYEQYIKWPWYIWLGFIAGLIAIVMVTIMLCCMTSCCSCLKGCCSCGSCCKFD
EPI_ISL_7747536/BA.2/Omicron     IDLQELGKYEQYIKWPWYIWLGFIAGLIAIVMVTIMLCCMTSCCSCLKGCCSCGSCCKFD
EPI_ISL_7747535/BA.2/Omicron     IDLQELGKYEQYIKWPWYIWLGFIAGLIAIVMVTIMLCCMTSCCSCLKGCCSCGSCCKFD
EPI_ISL_6795834/BA.2/Omicron     IDLQELGKYEQYIKWPWYIWLGFIAGLIAIVMVTIMLCCMTSCCSCLKGCCSCGSCCKFD
EPI_ISL_7718520/BA.2/Omicron     IDLQELGKYEQYIKWPWYIWLGFIAGLIAIVMVTIMLCCMTSCCSCLKGCCSCGSCCKFD
EPI_ISL_7580387/BA.2/Omicron     IDLQELGKYEQYIKWPWYIWLGFIAGLIAIVMVTIMLCCMTSCCSCLKGCCSCGSCCKFD
EPI_ISL_7701122/BA.2/Omicron     IDLQELGKYEQYIKWPWYIWLGFIAGLIAIVMVTIMLCCMTSCCSCLKGCCSCGSCCKFD
EPI_ISL_7190366/BA.2/Omicron     IDLQELGKYEQYIKWPWYIWLGFIAGLIAIVMVTIMLCCMTSCCSCLKGCCSCGSCCKFD
EPI_ISL_7644798/BA.2/Omicron     IDLQELGKYEQYIKWPWYIWLGFIAGLIAIVMVTIMLCCMTSCCSCLKGCCSCGSCCKFD
EPI_ISL_7740766/BA.3/Omicron     IDLQELGKYEQYIKWPWYIWLGFIAGLIAIVMVTIMLCCMTSCCSCLKGCCSCGSCCKFD
EPI_ISL_7605589/BA.3/Omicron     IDLQELGKYEQYIKWPWYIWLGFIAGLIAIVMVTIMLCCMTSCCSCLKGCCSCGSCCKFD
ON296657.1/BA.4/Omicron          IDLQELGKYEQYIKWPWYIWLGFIAGLIAIVMVTIMLCCMTSCCSCLKGCCSCGSCCKFD
ON331287.1/BA.4/Omicron          IDLQELGKYEQYIKWPWYIWLGFIAGLIAIVMVTIMLCCMTSCCSCLKGCCSCGSCCKFD
ON321245.1/BA.4/Omicron          IDLQELGKYEQYIKWPWYIWLGFIAGLIAIVMVTIMLCCMTSCCSCLKGCCSCGSCCKFD
ON337003.1/BA.4/Omicron          IDLQELGKYEQYIKWPWYIWLGFIAGLIAIVMVTIMLCCMTSCCSCLKGCCSCGSCCKFD
ON270681.1/BA.4/Omicron          IDLQELGKYEQYIKWPWYIWLGFIAGLIAIVMVTIMLCCMTSCCSCLKGCCSCGSCCKFD
ON373214.1/BA.4/Omicron          IDLQELGKYEQYIKWPWYIWLGFIAGLIAIVMVTIMLCCMTSCCSCLKGCCSCGSCCKFD
ON393428.1/BA.5/Omicron          IDLQELGKYEQYIKWPWYIWLGFIAGLIAIVMVTIMLCCMTSCCSCLKGCCSCGSCCKFD
ON393156.1/BA.5/Omicron          IDLQELGKYEQYIKWPWYIWLGFIAGLIAIVMVTIMLCCMTSCCSCLKGCCSCGSCCKFD
ON348053.1/BA.5/Omicron          IDLQELGKYEQYIKWPWYIWLGFIAGLIAIVMVTIMLCCMTSCCSCLKGCCSCGSCCKFD
ON323731.1/BA.5/Omicron          IDLQELGKYEQYIKWPWYIWLGFIAGLIAIVMVTIMLCCMTSCCSCLKGCCSCGSCCKFD
ON251467.1/BA.5/Omicron          IDLQELGKYEQYIKWPWYIWLGFIAGLIAIVMVTIMLCCMTSCCSCLKGCCSCGSCCKFD
ON250431.1/BA.5/Omicron          IDLQELGKYEQYIKWPWYIWLGFIAGLIAIVMVTIMLCCMTSCCSCLKGCCSCGSCCKFD
MZ911221/B.1.427/Epsilon         IDLQELGKYEQYIKWPWYIWLGFIAGLIAIVMVTIMLCCMTSCCSCLKGCCSCGSCCKFD
MZ375858/B.1.427/Epsilon         IDLQELGKYEQYIKWPWYIWLGFIAGLIAIVMVTIMLCCMTSCCSCLKGCCSCGSCCKFD
MZ375854/B.1.427/Epsilon         IDLQELGKYEQYIKWPWYIWLGFIAGLIAIVMVTIMLCCMTSCCSCLKGCCSCGSCCKFD
MZ943834/B.1.429/Epsilon         IDLQELGKYEQYIKWPWYIWLGFIAGLIAIVMVTIMLCCMTSCCSCLKGCCSCGSCCKFD
MZ277390/B.1.429/Epsilon         IDLQELGKYEQYIKWPWYIWLGFIAGLIAIVMVTIMLCCMTSCCSCLKGCCSCGSCCKFD
OU282717/B.1.429/Epsilon         IDLQELGKYEQYIKWPWYIWLGFIAGLIAIVMVTIMLCCMTSCCSCLKGCCSCGSCCKFD
OU235436/B.1.429/Epsilon         IDLQELGKYEQYIKWPWYIWLGFIAGLIAIVMVTIMLCCMTSCCSCLKGCCSCGSCCKFD
MZ687447/B.1.429/Epsilon         IDLQELGKYEQYIKWPWYIWLGFIAGLIAIVMVTIMLCCMTSCCSCLKGCCSCGSCCKFD
MZ363839/B.1.617.1/Kappa         IDLQELGKYEQYIKWPWYIWLGFIAGLIAIVMVTIMLCCMTSCCSCLKGCCSCGSCCKFD
MZ538882/B.1.617.1/Kappa         IDLQELGKYEQYIKWPWYIWLGFIAGLIAIVMVTIMLCCMTSCCSCLKGCCSCGSCCKFD
MZ724425/B.1.617.1/Kappa         IDLQELGKYEQYIKWPWYIWLGFIAGLIAIVMVTIMLCCMTSCCSCLKGCCSCGSCCKFD
MZ332524/B.1.617.1/Kappa         IDLQELGKYEQYIKWPWYIWLGFIAGLIAIVMVTIMLCCMTSCCSCLKGCCSCGSCCKFD
MZ332527/B.1.617.1/Kappa         IDLQELGKYEQYIKWPWYIWLGFIAGLIAIVMVTIMLCCMTSCCSCLKGCCSCGSCCKFD
LC633761/B.1.617.1/Kappa         IDLQELGKYEQYIKWPWYIWLGFIAGLIAIVMVTIMLCCMTSCCSCLKGCCSCGSCCKFD
MZ571142/B.1.617.1/Kappa         IDLQELGKYEQYIKWPWYIWLGFIAGLIAIVMVTIMLCCMTSCCSCLKGCCSCGSCCKFD
MZ562746/B.1.617.1/Kappa         IDLQELGKYEQYIKWPWYIWLGFIAGLIAIVMVTIMLCCMTSCCSCLKGCCSCGSCCKFD
OU053018/C.37/Lambda             IDLQELGKYEQYIKWPWYIWLGFIAGLIAIVMVTIMLCCMTSCCSCLKGCCSCGSCCKFD
OU268528/C.37/Lambda             IDLQELGKYEQYIKWPWYIWLGFIAGLIAIVMVTIMLCCMTSCCSCLKGCCSCGSCCKFD
OU100999/C.37/Lambda             IDLQELGKYEQYIKWPWYIWLGFIAGLIAIVMVTIMLCCMTSCCSCLKGCCSCGSCCKFD
MZ275295/C.37/Lambda             IDLQELGKYEQYIKWPWYIWLGFIAGLIAIVMVTIMLCCMTSCCSCLKGCCSCGSCCKFD
MZ275301/C.37/Lambda             IDLQELGKYEQYIKWPWYIWLGFIAGLIAIVMVTIMLCCMTSCCSCLKGCCSCGSCCKFD
MZ908711/C.37/Lambda             IDLQELGKYEQYIKWPWYIWLGFIAGLIAIVMVTIMLCCMTSCCSCLKGCCSCGSCCKFD
OU517038/C.37/Lambda             IDLQELGKYEQYIKWPWYIWLGFIAGLIAIVMVTIMLCCMTSCCSCLKGCCSCGSCCKFD
MZ496613/C.37/Lambda             IDLQELGKYEQYIKWPWYIWLGFIAGLIAIVMVTIMLCCMTSCCSCLKGCCSCGSCCKFD
OU466149/B.1.621/Mu              IDLQELGKYEQYIKWPWYIWLGFIAGLIAIVMVTIMLCCMTSCCSCLKGCCSCGSCCKFD
OU571573/B.1.621/Mu              IDLQELGKYEQYIKWPWYIWLGFIAGLIAIVIVTIMLCCMTSCCSCLKGCCSCGSCCKFD
OK025270/B.1.621/Mu              IDLQELGKYEQYIKWPWYIWLGFIAGLIAIVMVTIMLCCMTSCCSCLKGCCSCGSCCKFD
```

```
                                  1260      1270
                                     .         .
MN908947/Wuhan-Hu-1              EDDSEPVLKGVKLHYT
BS001137/B.1.1.7/Alpha           EDDSEPVLKGVKLHYT
BS001138/B.1.1.7/Alpha           EDDSEPVLKGVKLHYT
BS001139/B.1.1.7/Alpha           EDDSEPVLKGVKLHYT
BS001140/B.1.1.7/Alpha           EDDSEPVLKGVKLHYT
MW913362/B.1.1.7/Alpha           EDDSEPVLKGVKLHYT
MZ266365/B.1.1.7/Alpha           EDDSEPVLKGVKLHYT
MZ350109/B.1.1.7/Alpha           EDDSEPVLKGVKLHYT
MZ888516/B.1.1.7/Alpha           EDDSEPVLKGVKLHYT
OU562607/B.1.1.7/Alpha           EDDSEPVLKGVKLHYT
OU514166/B.1.1.7/Alpha           EDDSEPVLKGVKLHYT
OU538849/B.1.1.7/Alpha           EDDSEPVLKGVKLHYT
MW981442/B.1.351/Beta            EDDSEPVLKGVKLHYT
MZ298839/B.1.351/Beta            EDDSEPVLKGVKLHYT
MZ913100/B.1.351/Beta            EDDSEPVLKGVKLHYT
MZ879365/B.1.351/Beta            EDDSEPVLKGVKLHYT
OU539352/B.1.351/Beta            EDDSEPVLKGVKLHYT
MZ202314/B.1.351/Beta            EDDSEPVLKGVKLHYT
MZ298840/B.1.351/Beta            EDDSEPVLKGVKLHYT
LC643036/B.1.617.2/Delta         EDDSEPVLKGVKLHYT
LC643044/B.1.617.2/Delta         EDDSEPVLKGVKLHYT
MZ853946/B.1.617.2/Delta         EDDSEPVLKGVKLHYT
MZ854386/B.1.617.2/Delta         EDDSEPVLKGVKLHYT
MZ888532/B.1.617.2/Delta         EDDSEPVLKGVKLHYT
MZ888533/B.1.617.2/Delta         EDDSEPVLKGVKLHYT
MZ896136/B.1.617.2/Delta         EDDSEPVLKGVKLHYT
OU539261/B.1.617.2/Delta         EDDSEPVLKGVKLHYT
MZ854389/B.1.617.2/Delta         EDDSEPVLKGVKLHYT
OU539784/P.1/Gamma               EDDSEPVLKGVKLHYT
OU539830/P.1/Gamma               EDDSEPVLKGVKLHYT
OU539894/P.1/Gamma               EDDSEPVLKGVKLHYT
MZ477748/P.1/Gamma               EDDSEPVLKGVKLHYT
MZ477746/P.1/Gamma               EDDSEPVLKGVKLHYT
MW938104/P.1/Gamma               EDDSEPVLKGVKLHYT
MZ896179/P.1/Gamma               EDDSEPVLKGVKLHYT
MZ896359/P.1/Gamma               EDDSEPVLKGVKLHYT
OL677199/BA.1/Omicron            EDDSEPVLKGVKLHYT
OL672836/BA.1/Omicron            EDDSEPVLKGVKLHYT
OW714547.1/BA.1/Omicron          EDDSEPVLKGVKLHYT
OW714198.1/BA.1/Omicron          EDDSEPVLKGVKLHYT
OW303180.1/BA.1/Omicron          EDDSEPVLKGVKLHYT
EPI_ISL_7747552/BA.2/Omicron     EDDSEPVLKGVKLHYT
EPI_ISL_7747545/BA.2/Omicron     EDDSEPVLKGVKLHYT
EPI_ISL_7747536/BA.2/Omicron     EDDSEPVLKGVKLHYT
EPI_ISL_7747535/BA.2/Omicron     EDDSEPVLKGVKLHYT
EPI_ISL_6795834/BA.2/Omicron     EDDSEPVLKGVKLHYT
EPI_ISL_7718520/BA.2/Omicron     EDDSEPVLKGVKLHYT
EPI_ISL_7580387/BA.2/Omicron     EDDSEPVLKGVKLHYT
EPI_ISL_7701122/BA.2/Omicron     EDDSEPVLKGVKLHYT
EPI_ISL_7190366/BA.2/Omicron     EDDSEPVLKGVKLHYT
EPI_ISL_7644798/BA.2/Omicron     EDDSEPVLKGVKLHYT
EPI_ISL_7740766/BA.3/Omicron     EDDSEPVLKGVKLHYT
EPI_ISL_7605589/BA.3/Omicron     EDDSEPVLKGVKLHYT
ON296657.1/BA.4/Omicron          EDDSEPVLKGVKLHYT
ON331287.1/BA.4/Omicron          EDDSEPVLKGVKLHYT
ON321245.1/BA.4/Omicron          EDDSEPVLKGVKLHYT
ON337003.1/BA.4/Omicron          EDDSEPVLKGVKLHYT
ON270681.1/BA.4/Omicron          EDDSEPVLKGVKLHYT
ON373214.1/BA.4/Omicron          EDDSEPVLKGVKLHYT
ON393428.1/BA.5/Omicron          EDDSEPVLKGVKLHYT
ON393156.1/BA.5/Omicron          EDDSEPVLKGVKLHYT
ON348053.1/BA.5/Omicron          EDDSEPVLKGVKLHYT
ON323731.1/BA.5/Omicron          EDDSEPVLKGVKLHYT
ON251467.1/BA.5/Omicron          EDDSEPVLKGVKLHYT
ON250431.1/BA.5/Omicron          EDDSEPVLKGVKLHYT
MZ911221/B.1.427/Epsilon         EDDSEPVLKGVKLHYT
MZ375858/B.1.427/Epsilon         EDDSEPVLKGVKLHYT
MZ375854/B.1.427/Epsilon         EDDSEPVLKGVKLHYT
MZ943834/B.1.429/Epsilon         EDDSEPVLKGVKLHYT
MZ277390/B.1.429/Epsilon         EDDSEPVLKGVKLHYT
OU282717/B.1.429/Epsilon         EDDSEPVLKGVKLHYT
OU235436/B.1.429/Epsilon         EDDSEPVLKGVKLHYT
MZ687447/B.1.429/Epsilon         EDDSEPVLKGVKLHYT
MZ363839/B.1.617.1/Kappa         EDDSEPVLKGVKLHYT
MZ538882/B.1.617.1/Kappa         EDDSEPVLKGVKLHYT
MZ724425/B.1.617.1/Kappa         EDDSEPVLKGVKLHYT
MZ332524/B.1.617.1/Kappa         EDDSEPVLKGVKLHYT
MZ332527/B.1.617.1/Kappa         EDDSEPVLKGVKLHYT
LC633761/B.1.617.1/Kappa         EDDSEPVLKGVKLHYT
MZ571142/B.1.617.1/Kappa         EDDSEPVLKGVKLHYT
MZ562746/B.1.617.1/Kappa         EDDSEPVLKGVKLHYT
OU053018/C.37/Lambda             EDDSEPVLKGVKLHYT
OU268528/C.37/Lambda             EDDSEPVLKGVKLHYT
OU100999/C.37/Lambda             EDDSEPVLKGVKLHYT
MZ275295/C.37/Lambda             EDDSEPVLKGVKLHYT
MZ275301/C.37/Lambda             EDDSEPVLKGVKLHYT
MZ908711/C.37/Lambda             EDDSEPVLKGVKLHYT
OU517038/C.37/Lambda             EDDSEPVLKGVKLHYT
MZ496613/C.37/Lambda             EDDSEPVLKGVKLHYT
OU466149/B.1.621/Mu              EDDSEPVLKGVKLHYT
OU571573/B.1.621/Mu              EDDSEPVLKGVKLHYT
OK025270/B.1.621/Mu              EDDSEPVLKGVKLHYT
```

Supplemental Figure S3

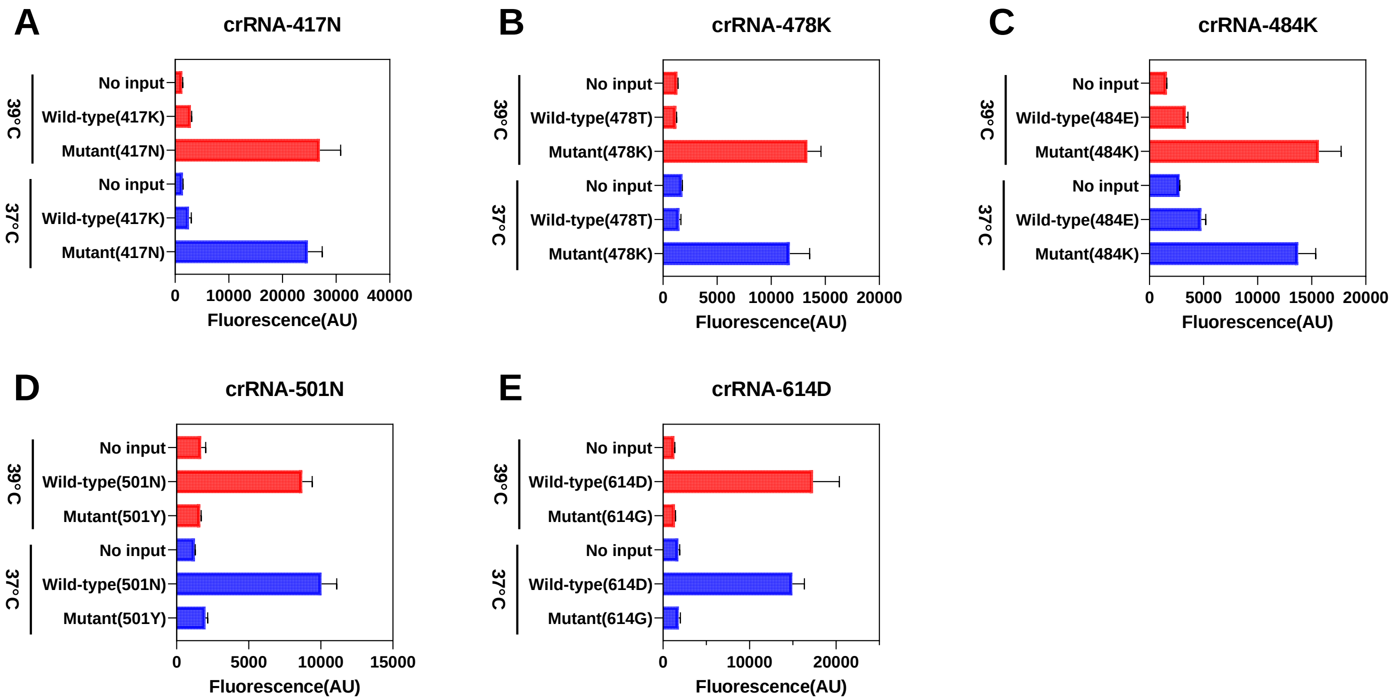

Supplemental Figure S4

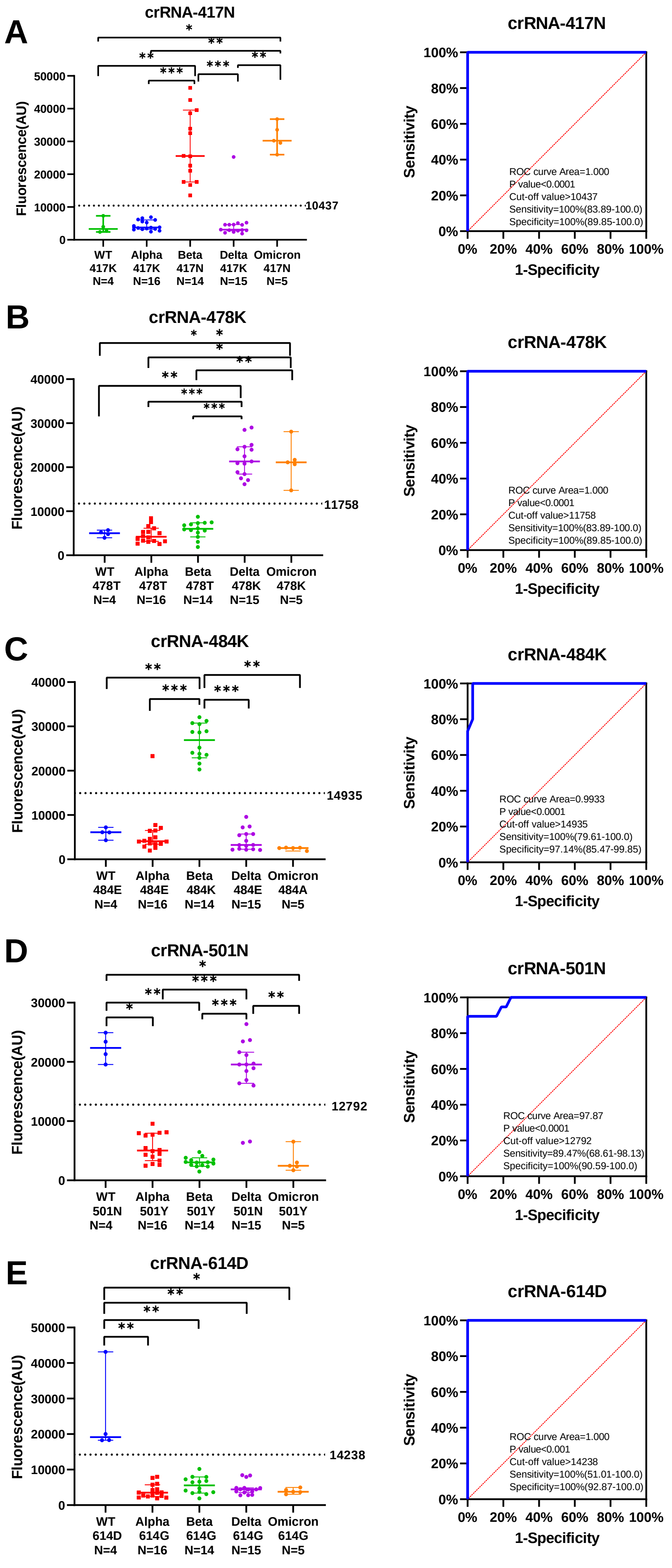

Supplemental Figure S5

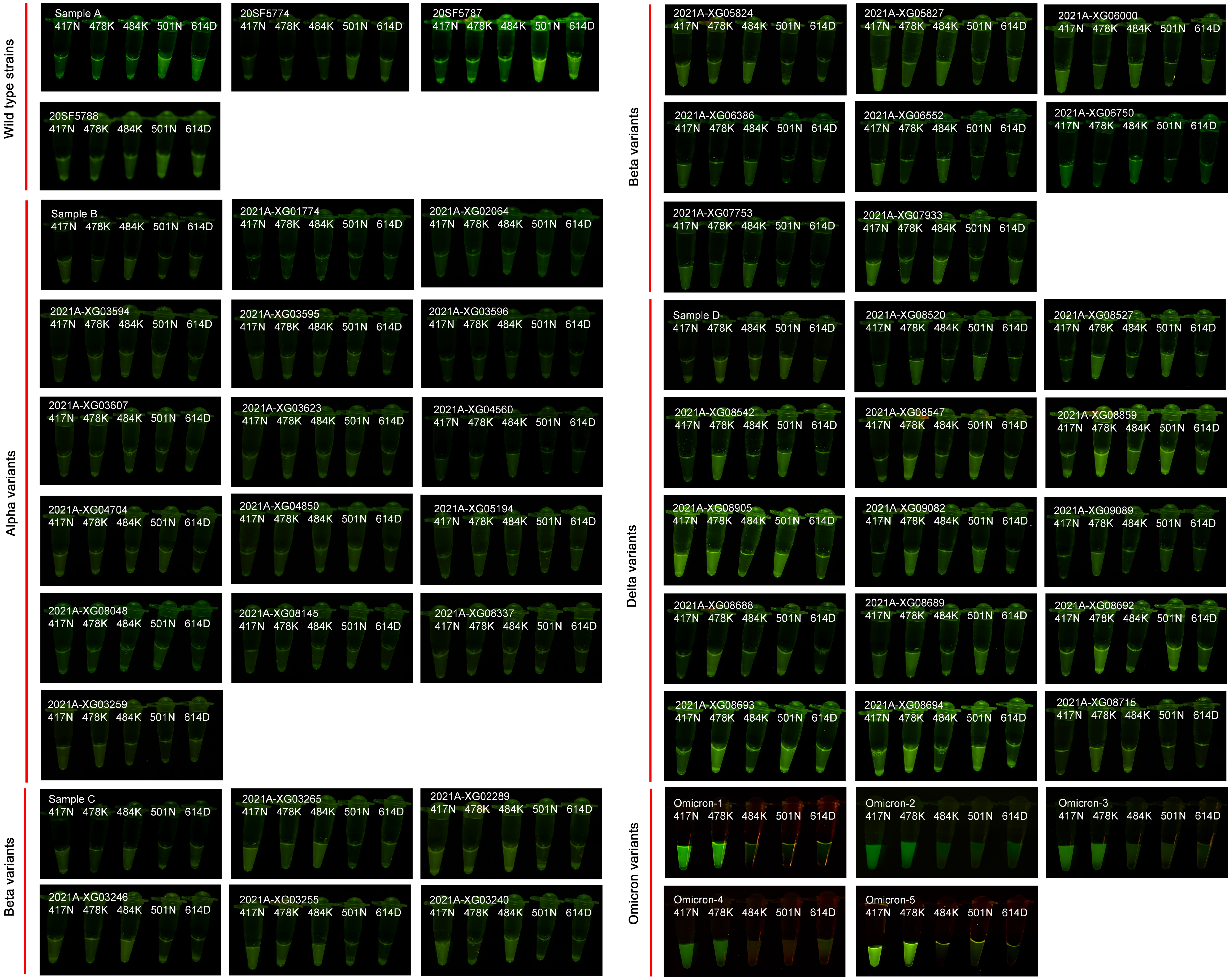

Supplement: Supplementary file 1 [file Data_Sheet_1.PDF]
